# Exploring fishing threat at fleet segment and subregional scale: Least expert knowledge and a resilience versus disturbance‐based approach as conservation's tools for cartilaginous fish

**DOI:** 10.1002/ece3.9881

**Published:** 2023-03-19

**Authors:** Umberto Scacco, Simone Di Crescenzo, Alice Sbrana

**Affiliations:** ^1^ National Centre of Laboratories‐Biology Italian Institute for Environmental Protection and Research (ISPRA) Rome Italy; ^2^ Department of Bio Ecological Sciences University of Tuscia Viterbo Italy; ^3^ Department of Life and Environmental Sciences University of Cagliari Cagliari Italy; ^4^ Department of Biology University of Rome Tor Vergata Rome Italy; ^5^ PhD program in Evolutionary Biology and Ecology University of Rome Tor Vergata Rome Italy

**Keywords:** elasmobranchs, fishing fleet's segments, IUCN red lists, priority species, risk assessment

## Abstract

Based on an explorative but rigorous elicitation framework, we obtained the bycatch fishing probability at the fishing fleet segment level using expert estimates. Based on the knowledge of three scientific experts, we developed a new and creative structured method for smart and fast fishery‐related risk assessments for species of high conservation concern. In order to test the method here propose, we applied it to 76 cartilaginous fish species (included in the IUCN Red Lists) and on five different fishing segments at both Italian and Mediterranean scale. The method produced qualitative results specific to the threat posed by fishing for each species and each segment with information between and within the segments. Based on the interpretation of resilience–disturbance interactions developed for ecological systems, the quantitative results provided reliable cumulative metrics, measuring the extinction risk due to fishing and the response to overfishing for the species considered. Additionally, the results highlight that the method perform best on a small geographic scale. Therefore, the application of this new method on other subregional or local scales where very few data are available (e.g., fishing effort) could be a valuable tool for the preliminary assessment for species of conservation concern. In fact, despite the absence of detailed catch data at local geographic scales, the flexibility of this method could help to highlight potential fishery‐related conservation problems and thus redirect conservation strategies for threatened marine species such as many sharks and rays species.

## Graphical abstract

Based on a simplified and attempt, yet rigorous, elicitation framework, we obtained expert's estimations of a species' probability of being caught (fatally), at fishing fleet segment scale. Based on these estimates, we developed a creative method, structured to assess the fishing‐related risks for species of conservation concern. Indeed, even in the absence of detailed catch data at the local geographic scale, the flexibility of this method could help highlight potential fishery‐related conservation problems and thus help reorient cartilaginous species conservation strategies accordingly.

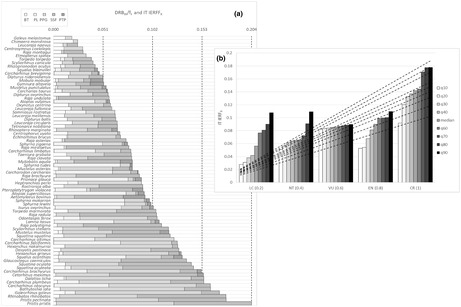

## 1 INTRODUCTION

Targeted and untargeted fisheries have negatively influenced cartilaginous fish populations over the past five decades (Pacoureau et al., 2021); the latter is considered the main culprit by the IUCN (Heppell et al., 1999; Musick et al., 2000). Additionally, habitat degradation and climate change are playing a key role in the rapid decline of elasmobranch populations (Delaval et al., 2021; Espinoza et al., 2020; Swift & Portnoy, 2021). This has led to repeated reports of an alarming global decline in cartilaginous fish populations at various scales (Baum et al., 2003; Davidson et al., 2016; Dulvy et al., 2014; Ferretti et al., 2008, 2010; Myers et al., 2007; Pacoureau et al., 2021).

Fishing mortality varies widely among cartilaginous fish species depending to gear and metier varieties. For example, pelagic longlines pose the main threat to sharks and pelagic rays in the world's oceans (Beerkircher et al., 2002; Gallagher et al., 2014; Kroodsma et al., 2018, Queiroz et al., 2019), including areas of the Mediterranean Sea (Ferretti et al., 2008; Megalofonou et al., 2005; Moro et al., 2019). Set gillnets and trammel nets tend to catch large numbers of sharks and rays (Benjamins et al., 2010; Coelho et al., 2005; Perez & Wahrlich, 2005; Scacco et al., 2012; Thorpe & Frierson, 2009; Valenzuela et al., 2008), as do the drift nets (McKinnell & Seki, 1998; Tudela et al., 2005). Small‐scale fisheries severely affect coastal‐dependent Chondrichthyes due to the use of passive fishing gears in coastal waters (Lloret et al., 2019; Roff et al., 2018; Smith & Basurto, 2019; Temple et al., 2018). Low‐selective active and semi‐active gears (FAO, 2016; Ferretti, 1981), such as bottom trawls (demersal and pelagic) and purse seines, have high fishing mortality for a mixture of demersal and pelagic species in the world's oceans and the Mediterranean Sea (Amande et al., 2008; Biton Porsmoguer & Lloret, 2020; Carbonell et al., 2003; Carpentieri et al., 2021; Fortuna et al., 2010; Psomadakis et al., 2008; Scacco et al., 2002, 2009, 2023; Stobutzki et al., 2002).

Despite the link between the species caught and the fishing gear used, quantitative empirical data can have high uncertainty, as the density and distribution of cartilaginous fish species in overlapping fisheries can vary widely as a function of spatiotemporal variables (Kroodsma et al., 2018; Murua et al., 2021; Queiroz et al., 2019; Williamson et al., 2020). This is particularly true in the Mediterranean Sea, where Chondrichthyes have rather unpredictable distributions and home ranges due to their natural low density, occasional vagrant or endemic‐stenoid status (Tortonese, 1956) in this basin.

The high level of uncertainty about the extinction risk of fisheries of many species belonging to this group is also related to the difficulty of assessing the contributing role of different fisheries to the overall extinction risk. This role is primarily related to the fishing selectivity of individual gears used within each fishing fleet segment toward individual species. Although it is a measurable variable (Millar & Fryer, 1999; Stewart, 2002), gear selectivity can vary widely even within the same gear, depending on local fishing techniques and habits (FAO, 2016; Graham et al., 2007). Moreover, exhaustive information on the variability of species‐specific gear interactions is rarely available (Young et al., 2019). Therefore, catch and distribution data for these species are sparse and/or insignificant and likely suffer from underestimation due to the difficulty of obtaining them empirically.

At the same time, several elasmobranch populations suffer dramatically from anthropogenic impacts (Walls & Dulvy, 2020), in particular fishing pressure. This generated and risen up the paradoxical situation suffered by several species: the urgent need for an assessment of the risk of extinction, despite being deficient in the data needed for a correct assessment. As proof of this, many data‐deficient (DD) species observed at subregional IUCN Red List (i.e., Mediterranean or Italian scale) are generally higher in number when compared to a larger scale (i.e., Global or European scale), and, probably, they are underestimated as far as extinction risk at smaller scales.

The present work aims to fill such an information gap by a twofold approach combining the expert knowledge of a minimum number of experts with an easy framework for species risk assessment to fishing by different fishing segments,

On the one hand, the formal elicitation of scientific expert knowledge can help provide the uncertain quantities targeted. One of the definitions of knowledge is the fact or condition of knowing something with familiarity gained through experience or association (Merriam‐Webster Dictionary, 2021). Scientific expert knowledge about a specific subject is therefore a combined output of individual knowledge, up‐to‐date literature, and scientific experience (Humphreys et al., 2009; Johnson & Gillingham, 2004). Inherent variability in experts' judgments (Cox et al., 2014; Morgan & Henrion, 1990) can be useful for ecological assessments (O'Hagan, 2019). Considering the case of a triangular distribution (Kotz & Van Dorp, 2004), three evaluation points (experts) are often sufficient to generate a valid estimate; experts are generally able to provide the minimum, or possibly maximum, values that the measure can take (Morgan & Henrion, 1990). There is an extensive literature on the formal elicitation of expert knowledge and some leading protocols were developed (Burgman, 2015; Cooke, 1991; Oakley & O'Hagan, 2016; Rowe & Wright, 1999). Despite this, these frameworks are structured along serial and complex procedures that slow the entire flow and can introduce significant biases in the final assessment. Therefore, we aimed first at reducing all elicitation steps at the minimum through proposing an innovative assumption for developing a multivariate method for least expert knowledge elicitation.

On the other hand, the scarcity of empirical baseline catch data for many species has led researchers to develop alternative techniques to establish reliable species risk assessments, such as fuzzy logic analysis (Cheung et al., 2005, 2007), Bayesian models (Punt & Hilborn, 1997), extinction risk to global anthropogenic pressures (Dulvy et al., 2014), productivity–susceptibility‐based assessments (Astles et al., 2006, 2009; Cortés et al., 2009, 2015; Osio et al., 2015), and the “Robin Hood” approach (Punt et al., 2011). Despite the scientific value of the information provided, they appear very technical and complex to be replicated on a reduced scale. Recent developments in data‐gathering techniques (Azzurro & Cerri, 2021) suggest that species risk assessments for fishing could be more useful through a localized approach. Indeed, facing conservation issues on a reduced scale would ensure a more precise identification of actual and potential species refuges (Lauria et al., 2015).

The basic concepts of species resilience and resistance to a given disturbance, fishing pressure included, offer the chance for a most user‐friendly approach to fishing risk assessment. These two species‐specific attributes can complexly interact and cooperate with each other, when extending to the species the concepts elaborated for the resistance–resilience traits of ecological systems (Walker et al., 2004). However, it appears that species resistance is somehow inversely related to resilience, as postulated in the classical interpretation (Grimm & Wissel, 1997; Holling, 1996). In the fishing context, the resilience acts primarily at the population level and represents the ability of a species to reestablish a condition of demographic balance when steady fishing mortality has significantly altered it within the population (Froese et al., 2017), given the fishing gear used. Elasmobranchs are generally low‐resilience species, given their peculiar life‐history traits such as slow growth, delayed maturity, and low fecundity rates, all of which make them particularly vulnerable to overfishing (Cortés, 1998, 2002).

Differently, resistance is the average individual response of a species to capture by a given fishing gear (Froese et al., 2017; Holling, 1996). It can be measured by the average probability that an individual is alive or dead following the capture. In contrast to low resilience, cartilaginous fish show high resistance (Chin et al., 2015; Scacco et al., 2023) instead, common trait of K‐selected compared to r‐selected species (Pianka, 1970). Synoptically, species respond to a disturbance as a function of how it develops (Holling, 1996). Pulse and more intense disturbances trigger more rapid impacts on species resistance compared with steady disturbances that tend to trigger resilience dynamics (Holling, 1996). Based on these concepts, the second aim of this work was to develop metrics to separate species based on their resilience and resistance potentials to fishing pressure by different fishing segments.

Overall, the main aim of this work was to provide an easy replicable tool for a rapid risk assessment to fishing of elasmobranch species, given the different fishing efforts displayed by fishing segments on a given spatial and temporal scale.

## 2 METHODS

### 2.1 The elicitation experiment

We first conducted an expert knowledge elicitation experiment to obtain quantitative data as Estimated mean individual disaggregated Probability of fatal Catch (EPC_*IX*_) for *X* ***=*** 76 cartilaginous species (Appendices A1–A2). and for *I* = 5 selected fleet segments (FFL, see 2.3; S1), recorded on the Italian and Mediterranean scale. We developed the preliminary steps of the protocol based on Burgman (2015), but we used a lower number of randomly selected experts (3) to demonstrate that it was sufficient for the wisdom of the crowd effect compared with that usually recommended (Morgan, 2014). The three experts were randomly selected from 10 shark experts profiled by scientific and field experience in fishing operations in the Mediterranean Sea (Appendix A1). We structured the elicitation question (Appendix A2) to elicit multiple dependent quantities for a set of uncertain proportions that must sum to 1 (O'Hagan, 2019) in one round‐assessment for each expert. Prior to estimation, we supplied experts with a simple guide (S1) that outlined the fishing gear to be considered within each FFL. We asked them to provide the best single estimates without a confidence interval for each species when assessing percentages within a segment, and so on for all fleet segments considered.

Based on a generalization of the uniqueness quantification theory (Andrews, 2002; Kleene, 1952), we assumed that the assertion “exactly k (5) objects exist such that their sum is simultaneously one” could fit a simultaneous assessment of five dependent quantities that an expert is asked to provide such that they add up to one (Appendix A3). Therefore, we considered the estimates of each expert per species within a segment as five singletons EPC_*IX*_, that is, experts provided quantities that were inherently the best estimates, as each measure delimited the other. To consider the uncertainty around the best estimates, we first define the “mother distributions” as the beta distributions that match the species' estimates provided by the expert for each of the segments and then extrapolate the four quartiles accordingly (Appendix A3). Based on these, we assign the best estimate for each individual species to the interval it belonged to, consequently obtaining an upper and lower bound per species and segment (Appendix A3). Prior beta distributions were used to fit the resulting intervals for each species within each segment and to obtain associated quartiles. In doing this task, we used the roulette method (3 grids x 20 bins, 3 betting coins) through the online elicitation tool MATCH (Morris et al., 2014). Betting approaches might diminish overconfidence (Ferretti et al., 2016) or experts' underestimation of sampling variability (Tversky & Kahneman, 1974). Additionally, limiting the number of betting coins minimizes the bias in beta distribution fitting associated with the different coins' disposition patterns an expert may assign to bins when using the recommended number (about 20, O'Hagan, 2019) of coins (Stefan et al., 2020). We repeated this routine for each of the experts. The prior beta distributions were then mathematically aggregated by a linear combination, which is well‐suited for idempotency. As opposed to the classical model, averaging probability densities (Cooke & Goossens, 2008), we averaged quartiles (first, second, and third quantiles) (Lichtendahl et al., 2013) as the resulting distribution is more concentrated around the median, thus reducing the suspected problem of wide uncertainty distributions in combined assessments (Lichtendahl et al., 2013). Therefore, we used the combined interquartile for each species within a segment to fit the marginal beta distributions for species and segments, and then to compare them to an adjusted Dirichlet distribution whose marginal beta distributions were as close as possible to the elicited distributions (O'Hagan, 2019). To do this task, we performed the analyses with the online elicitation tool available at https://jeremy‐oakley.shinyapps.io/SHELF‐Dirichlet/ (Zapata‐Vazquez et al., 2014). The analyses were run on percentages rescaled in the 0–1 interval. The species values of 0 for all quartiles (i.e., species estimated not to be caught within a given segment) were arbitrarily set at 0.0001, 0.0002, and 0.0003 for the first, second, and third quartiles, respectively.

We checked for the overall level of agreement between the experts' estimates by Friedman Anova, separately applied to species grouped in two broad morpho‐ecological categories (A: Hexanchiformes, Lamniformes, Carcharhiniformes, and Squaliformes; B: Squatiniformes, Rajiformes, Rhinopristiformes and Chimaeriformes) within each FFL for the sake of data homogeneity.

### 2.2 Fishing risk charts of species by segments

The fitted intervals of EPC_*IX*_ were coupled with the corresponding values of intrinsic vulnerability to fishing IVF_*X*_ (Cheung et al., 2005) to provide a graphical representation of qualitative assessment of species risk to fishing taking care to use IVF_*X*_ data as coeval as possible (FishBase, 2016, 2021) with those used in subsequent quantitative analyses. For each FFL and species group, we measured EPC_*IX*_ on the vertical (y) axis (gradient of disturbance ranging from 0 to max EPC_*IX*_ within that segment) and the corresponding IVF_*X*_ on the horizontal (x) axis (gradient of species resilience ranging from min to max IVF_*X*_ within that segment), thus obtaining 10 Cartesian planes. Halving the vertical and horizontal axes in each plane, we obtained two reference values (OB) and (AB) and four quadrants accounting for four levels of risk (disturbance vs response; Cox et al., 2014). To compensate for the interdependence between EPC_*IX*_ across FFLs, we normalized data choosing OB and AB always as the half of 0‐max range of EPC_*IX*_ and min–max of IVF_*X*_ at FFL and species' group levels, thus obtaining the four areas correspondingly. Heuristic criteria ruled the association of species' intervals to one or other quadrants (Table 1). According to the quadrant association, we parametrize the risk level of species (*X*) by FFL (*I*) correspondingly by assigning a QS_*IX*_ quantitative score for each FFL and species (Table 1). We used chi‐squared tests to search for differences in the breakdown of threats (the number of species per quadrant) fishing poses to the species (simple chi‐squared tests on the two species groups by FFL, separately) and between FFLs (a 5X4 contingency table with five FFLs as rows and four levels of risk as columns for aggregated species groups).

**TABLE 1:** Heuristic rules used to assign the 76 cartilaginous species (X), reported at the Italian and Mediterranean scale, to quadrants (L—lower left, LI—upper left, IH—lower right, and H—upper right) that graphically represent the risk posed from fishing related activities for each of the five representative fishing fleet segments (I), based on expert knowledge (EPC_IX_) and vulnerability to fishing (IVF_X_).

Median location	Quartiles (in)equality	Vulnerability location (^a^IVF_X_)		
		IVF_X_ < ^b^AB_I_	IVF_X_ = AB_I_	IVV_X_ > AB_I_
MEDIAN ^c^EPC_IX_ < ^d^OB_I_	|^c^Q_75_ EPC_IX_ −O_B_| ≤ |^c^Q_25_ EPC_IX_ −O_B_|	^e^L (0.25)	^e^L‐LI (0.375)	^e^IH (0.75)
	|Q_75_ EPC_IX_ −O_B_| > |Q_25_ EPC_IX_ −O_B_|	^e^LI (0.50)	^e^LI‐H (0.75)	^e^H (1)
MEDIAN EPC_IX_ = OB_I_	|Q_75_ EPC_IX_ −O_B_| < |Q_25_ EPC_IX_ −O_B_|	L (0.25)	L‐LI (0.375)	IH (0.75)
	|Q_75_ EPC_IX_ −O_B_| > |Q_25_ EPC_IX_ −O_B_|	LI (0.50)	LI‐H (0.75)	H (1)
	|Q_75_ EPC_IX_ −O_B_| = |Q_25_ EPC_IX_ −O_B_|	L‐LI (0.375)	^e^L‐LI‐IH‐H (0.625)	^e^IH‐H (0.875)
MEDIAN EPC_IX_ > OB_I_	|Q_75_ EPC_IX_ −O_B_| < |Q_25_ EPC_IX_ −O_B_|	L (0.25)	L‐IH (0.5)	IH (0.75)
	|Q_75_ EPC_IX_ −O_B_| ≥ |Q_25_ EPC_IX_ −O_B_|	LI (0.5)	LI‐H (0.75)	H (1)

^a^ IVF_IX_ is intrinsic vulnerability to fishing of species (Cheung et al., 2005) targeted according to fishing fleet segment I.

^b^ AB_I_ is abscissa benchmark according to fishing fleet segment I.

^c^ EPC_IX_ (%) (percentage, as median, lower and upper quartiles) is Estimated Probability of fatal Catch, based on estimates of three independent, profiled and selected experts.

^d^ OB_I_ is ordinate benchmark according to fishing fleet segment I.

^e^ L, LI, IH and H are Low, Low to Intermediate, Intermediate to High and High qualitative levels of fishing threat, respectively, and all their possible combinations, for the considered species. Numbers in brackets are the corresponding four basic qualitative scores (L = 0.25; LI = 0.50; IH = 0.75; H = 1) and different combined scores (the mean) for data shared between two and among four quadrant types.

### 2.3 Fishing effort data: Extraction and grouping criteria

The fishing effort data by vessel were extracted from the JRC database (JRC, 2020) referring to a four‐year period (2015–2018). The database contains records at the scale of a single vessel, together with some fishery information, provided according to the official standard codification (JRC, 2020). The data considered for analysis were those referred to vessels censed in Mediterranean countries, intended as data on member state fishing activities carried out in Mediterranean and Black Sea waters. Records selected for the analysis were those having the following information at least: (1) macro‐ and subareas, (2) vessel belonging country, (3) fishing effort by year (expressed as gross tonnage × fishing days by year), (4) vessel length (expressed as categories based on length size ranges), (5) category of fishing gears yearly used and expressed as official identification alphanumerical code (FAO, 2016), and (6) a valid fishing effort data entry. Based on database information, we extracted and grouped data according to the three criteria in the opinion of the authors:

1. Ensuring a more focused representation of the threat at the species level extracting effort data for fishing gear that potentially poses a threat to cartilaginous species ► selecting fishing gear.
2. Providing a better representation of fishing effort in the two main fishing areas (i.e., inshore and offshore) ► grouping effort data by vessel length.
3. Better representation of the selectivity of the fishing gear toward the considered species, as an “average” selectivity at the level of the fishing segment, ► grouping gear effort data by representative fishing segment.

As a result, we obtained five FFLs: (1) bottom trawlers (BT: 2737 and 1138 valid records at Mediterranean and Italian scales, respectively) that aggregated vessels having a length over 12 m and allowed to fish in offshore waters beyond 3 Nm or below 50 m depth using different types of bottom‐demersal trawl nets (Council Regulation, 2006; FAO, 1972, 1985, 1995, 2016); (2) pelagic longline (PL: 937 and 299 records) that grouped those boats having a length over 12 m and using passive gears such as pelagic hooks and lines, generally fishing in offshore waters (Council Regulation, 2006; FAO, 1972, 1985, 1995, 2016); (3) purse seines and pelagic trawlers (PPT: 1774 and 763 records) accounted for boats larger than 12 m as vessel length, generally fishing actively or semi‐actively in the water column of offshore waters (Council Regulation, 2006; FAO, 1972, 1985, 1995, 2016); (4) passive polyvalent gears (PPG: 2459 and 652 records) referred to those boats larger than 12 m, using a wide range of passive fishing gears, such as bottom longlines, gill and trammel or combined nets, fishing generally in offshore waters but also inshore depending on size (Council Regulation, 2006; FAO, 1972, 1985, 1995, 2016); and (5) small‐scale fishery (SSF: 7190 and 1431 records) individuated small vessels up to 12 m in vessel length, generally using artisanal or semi‐industrial passive fishing gears such as nets and hooks (pots were excluded from analyses) and, occasionally, also low effort active and semi‐active fishing gears, both within inshore waters (Council Regulation, 2006; FAO, 1972, 1985, 1995, 2016). The fleet segmentation used for the analyses was very close to that officially in force by the CFP in EU waters 4 (JRC, 2020). Finally, mean absolute values of fishing effort by corresponding FFLs were expressed as relative measures (percentages) of their total cumulative value (Table 2), at Italian and Mediterranean scales (IT or MED fI, respectively). We furtherly used such percentages to obtain cumulative metrics calculated both at Italian and at Mediterranean scales.

**TABLE 2:** Fishing effort data disaggregated by fishing segment, expressed as percentages of the total, and measured as mean gross tonnage × fishing days at the Italian and Mediterranean scales.

Relative fishing effort by fishing fleet segment (% mean of gross tonnage × fishing days)	BT	PL	PPG	SSF	PTP
IT	60.26	11.49	3.46	6.75	18.04
MED	56.14	8.22	11.17	4.32	20.15

### 2.4 Fishing risk metrics of species by segments

For a quantitative representation of species risk to fishing, we extended the concept of resilience (Walker et al., 2004) and disturbance (Grimm & Wissel, 1997; Holling, 1996) developed for ecological systems to species studied. Therefore, we modeled an intrinsic metric defined as disturbance–resilience balance by species (*X*) and FLL (*I*):

(1) $${\mathrm{DRB}}_{\mathrm{IX}}=\left(\left(\text{mean}\;{\mathrm{EPC}}_{\mathrm{IX}}\right)/{\mathrm{IVF}}_{X}\right))*{\mathrm{QS}}_{\mathrm{IX}}$$

Mean EPC_IX_ was calculated upon median, first, and third quartiles fitted from expert estimates. To deal with the actual level of fishing pressure, we combined DRB_IX_ with fishing effort data from a 4‐year period for the FFLs considered in this analysis (see chapter 2.2; S1), obtaining two different cumulative metrics depending on how the fishing effort corresponded to weight DRB_IX_:

Index of Fishing Response.

(2) $${\mathrm{IFR}}_{X}=\sum_{I}\;\left({{\mathrm{RRB}}_{\mathrm{IX}}}^{*}f_{I}\right)$$

by species, which represented the sum of products between individual FFL contribution by species (DRB_IX_) and the corresponding fishing efforts (f_I_),

and

Index of Overfishing Response.

(3) $${\mathrm{IOR}}_{X}{=}^{’}\left({\mathrm{IFR}}_{X}\right)$$

which represented the first derivative of IFR_X_ along IUCN ranked categories in metrics' verification framework.

Index of Extinction Risk to Fishing.

(4) $${\text{IERF}}_{X}=\sum_{I}\;\left({\mathrm{RRB}}_{\mathrm{IX}}/f_{I}\right)$$

by species, which was the sum of rates between the individual contribution of FFL by species (DRB_*IX*_) and the corresponding fishing efforts (f_*I*_).

By analogy, we elaborated the sum of fishing effort‐weighted,

divided.

(5) $${\mathrm{SW}}_{\left(/\right)}{\mathrm{QS}}_{X}=\sum_{I}\left({\mathrm{QS}}_{\mathrm{IX}}/f_{I}\right)$$

or multiplied,

(6) $${\mathrm{SW}}_{\left(*\right)}{\mathrm{QS}}_{X}=\sum_{I}\left({{\mathrm{QS}}_{\mathrm{IX}}}^{*}f_{I}\right)$$

qualitative scores for a back‐verification of the method. Acronyms, formulas and meaning of the metrics are summarized in Table 3.

**TABLE 3:** List of the metrics developed for the risk assessment method, with related acronym, meaning, and mathematical formula.

Variable acronym	Formula	Meaning
DRB_*IX*_: Disturbance Resilience Balance for the X species within the I segment	DRB_*IX*_ = ((mean EPC_*IX*_/IVF_*X*_))*QS_*IX*_	It measures the intrinsic response of a species resilience to fishing disturbance by a given segment
IFR_*X*_: Index of Fishing Response by species	IFR_*X*_ = ∑_I_ (DRB_IX_*f_*I*_)	It measures the response of a species resilience to actual fishing effort by all cumulated segments
IOR_*X*_: Index of Overfishing Response by species	IOR_*X*_ = (IFR_*X*_)	It is the first derivative of IFR_X_. It measures the status of a species with respect to overfishing by all cumulated segments
IERF_*X*_: Index of estimated Extinction Risk to Fishing by species	IERF_*X*_ = ∑_*I*_ (RRB_*IX*_/f_*I*_)	It measures the response of a species resistance to actual fishing effort by all cumulated segments
SW_(*)_QS_*X*_: Sum of fishing effort Weighed (multiplied) Qualitative Scores	SW_(*)_QS_*X*_ = ∑_*I*_ (QS_IX_*f_*I*_)	It measures the response of a species resilience to actual fishing effort by all cumulated segments and based on qualitative scores alone.
SW_*(/)*_QS_*X*_: Sum of fishing effort Weighed (divided) Qualitative Scores	SW_*(/)*_QS_*X*_ = ∑_*I*_ (QS_*IX*_/f_*I*_)	It measures the response of a species resistance to actual fishing effort by all cumulated segments and based on qualitative scores alone.

### 2.5 Statistical analyses

We verified IFR_*X*_ and IERF_*X*_ by comparing them with ranked IUCN red lists (IT and MED IUCN_*RX*_) on the Italian and Mediterranean scales (Dulvy et al., 2016; Rondinini et al., 2013). Rankization of IUCN red lists followed a linear scale as follows: LC = 0.2 > NT = 0.4 > VU = 0.6 > EN = 0.8 > CR = 1, and the rank for IUCN species not applicable (NA) (that is species considered occasional and irregular vagrant within a geographical area) was replaced by the corresponding rank at the contiguous and larger geographical scale. To verify the metrics, we first used a descriptive principal component analysis (PCA) to explore the correlation between the active (DRB_*IX*_, IT or MED IERF_*X*_, IT or MED IFR_*X*_ and IVF_*X*_) and supplementary (IT IUCN_*RX*_, MED IUCN_*RX*_ and GLO IUCN_*RX*_) variables on the Italian and Mediterranean scales. Global IUCN risk assessment (GLO IUCN_*RX*_) and IVF_*X*_ were used as control series. They functioned as a control for experimental variables because they were directly involved in metric formulation (IVF_*X*_) or did not have a corresponding fishing effort‐weighted data to compare with GLO IUCN_*RX*_.

Using separate Kruskal–Wallis ANOVAs (DD species included), we tested for differences in DRB_IX_, IERF_X_, and IFR_X_ between species grouped by IUCN risk category on the two scales.

The goodness‐of‐fit models (IERF_*X*_ and IFR_*X*_ vs. IUCN_*RX*_ at the corresponding geographical scale) was tested using linear (y = a*x in the case of IERF_*X*_), or nonlinear (y = a*x^2^‐b*x + c in the case of IFR_*X*_) 10TH stepped percentile regressions (DD species excluded). We represented the first derivative (y = (2 * a * x)‐b) of the estimated functions having significant models to obtain IOR_*X*_.

We performed a generalized linear model analysis (GLMA) to test for the best predictor among IUCN_*RX*_ at different scales (IT and MED, and GLO as control; DD species excluded) and corresponding response variables (IERF_*X*_ and IFR_*X*_, and IVF_*X*_ as control) at the two tested scales. We used the best distribution models (Beta) to fit response variables (S2) and a logit link function as error distributions in the analyses. We finally performed an identically structured GLMA with SW_(*/*)_QS_*X*_ and SW_(*)_QS_*X*_ as response variables of the analysis for a back‐verification of the method.

**FIGURE 1:**
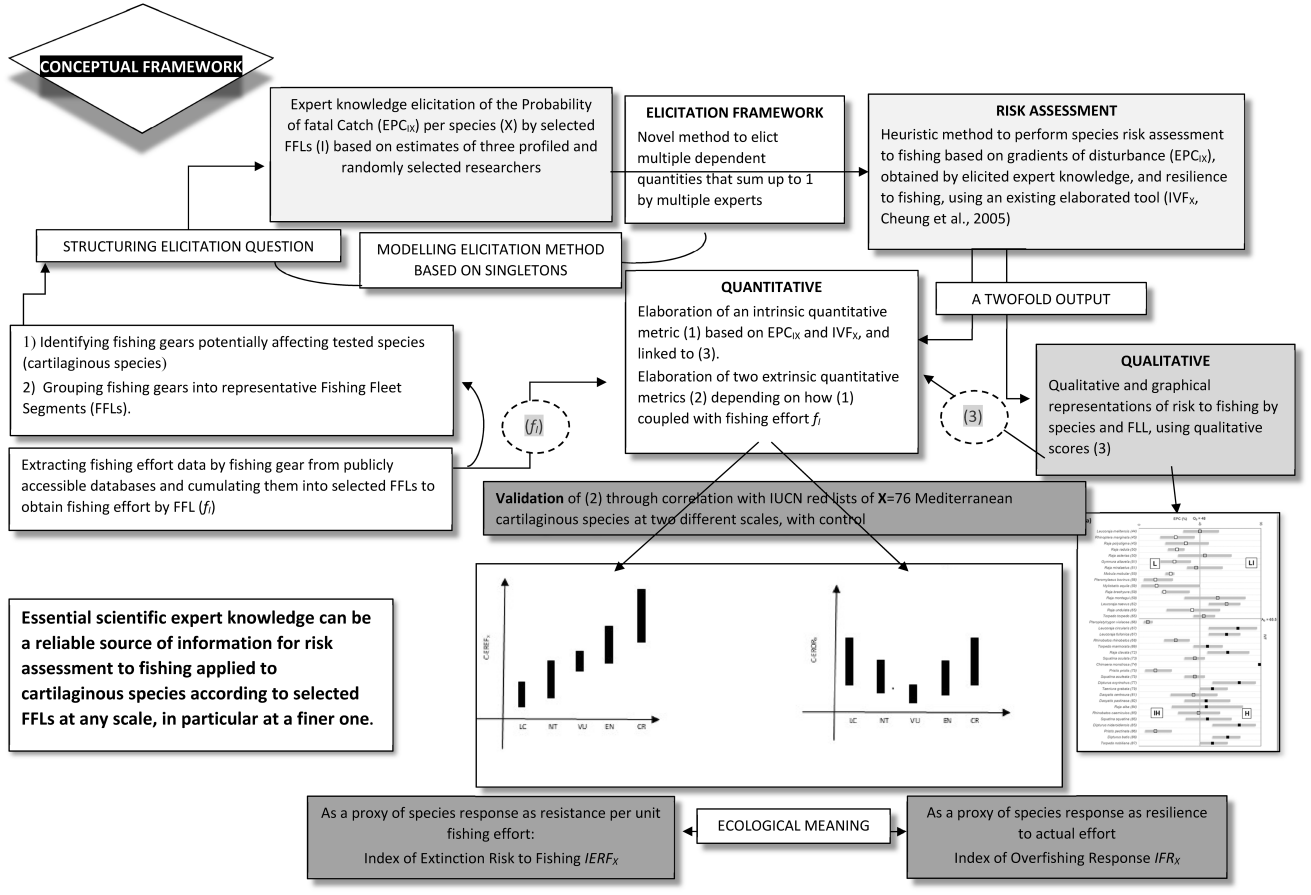
General conceptual framework followed to test the fisheries related species risk assessments on Mediterranean cartilaginous fauna. Using a minimum number of experts to provide estimations of the probability of fatal catch by species according to five representative fishing fleet segments, which had corresponding fishing effort data.

We performed statistical analyses by *STATISTICA packages 7.1 and + R3.6.1 (R core team, 2019). A schematic model of the general conceptual framework followed in this work and a glossary of acronyms used throughout is provided in Figure 1 and in S3, respectively.

## 3 RESULTS

### 3.1 The elicitation experiment

**FIGURE 2:**
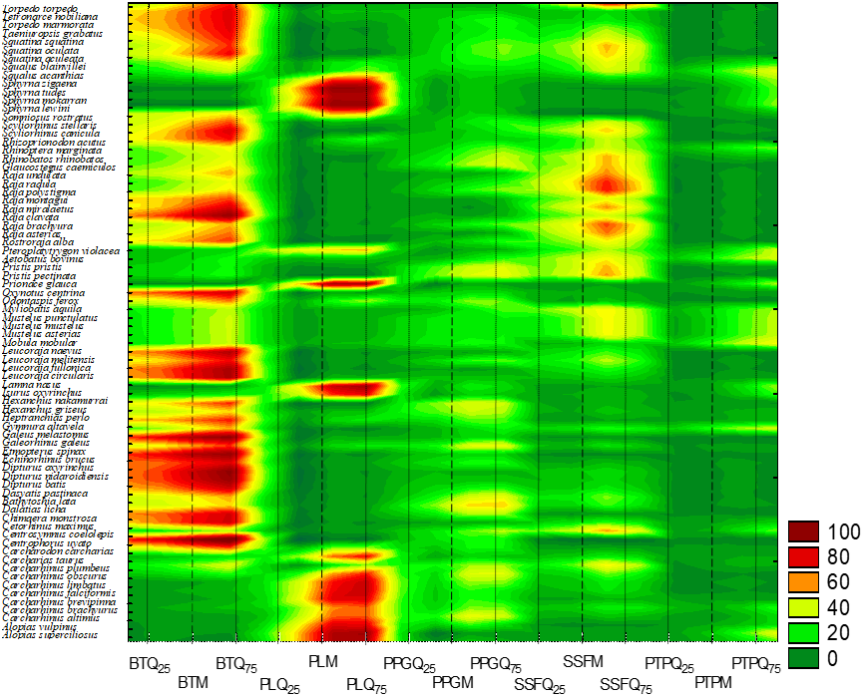
Distribution map of EPC_*IX*_ based on median (M), first (Q25) and third (Q75) quartiles from experts' aggregated distributions of elicited estimates, with *X* as species (76 Mediterranean cartilaginous fish) and *I* as fishing fleet segments (BT, bottom trawl; PL, pelagic longlines; PPG, passive polyvalent gears; PTP, pelagic trawlers and purse seine; SSF, small scale fishery).

The elicitation results (S4) are graphically summarized in the distribution map of EPC_*IX*_. It appeared the disaggregated probability of fatal catch was clearly different according to FFL and species (Figure 2). The level of uncertainty between EPC_*IX*_ was generally lower for species of group A than B, in all FFLs considered (Appendix B1).

### 3.2 Fishing risk charts of species per fishing segment

**FIGURE 3:**
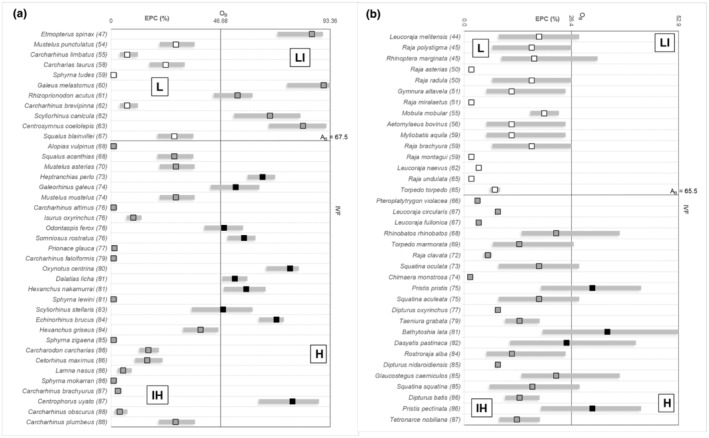
(a, b) Graphical representations of the qualitative risk from fishing for species of group A (Hexanchiformes, Lamniformes, Carcharhiniformes and Squaliformes) in bottom trawls (a) and for species of group B (Squatiniformes, Rajiformes, Rhinopristiformes and Chimaeriformes) in passive polyvalent gears (b). EPC (as median, 1st and 3rd quartiles) is the Estimated Probability of fatal Catch based on estimates by three independent, profiled and selected sharks experts and plotted against Intrinsic Vulnerability to Fishing IVF (Cheung et al., 2005) (values in brackets). Based on half the horizontal (OB) and vertical (AB) axes, the quadrants identify four different levels of risk to fishing (L: low risk; LI: low to intermediate risk; IH: intermediate to high risk; H: high risk) where species may fall (L: white squares; LI and IH: gray squares; H: dark squares; 1 shared species IH‐H in species group A within BT, dark line), according to heuristic rules based on relative differences between species horizontal and vertical values and horizontal (OB) and vertical (AB) reference values, respectively. Data used for IVF_*X*_ refer to those downloaded at (www.fishbase.de) in 2015–2016. Scale fishery, PTP Pelagic Trawlers and Purse Seine.

Results from the qualitative risk assessment to fishing showed FFLs targeted different assemblages of species (Figure 3a,b; Appendices B2a–h; S5), with different categorizations of fishing threats (L, LI, IH, H, and corresponding QS_IX_) within and between the different FFLs. Within‐check indicated that all segments exhibited a prevalence of IH and H species over the total targeted, but significant for the species of group A only, in four segments over five (Appendix B3). The analysis between segments indicated BT, PPG, and PL as segments of primary threat to the tested species (in terms of the number of targeted H species) and, secondarily, PTP and SSF (in terms of the number of targeted IH species) (Appendix B4).

### 3.3 Fishing risk metrics of species by segments

The results of the quantitative risk assessment to fishing, DRB_*IX*_ showed three different patterns describing how FLLs exert fishing threat on the species considered, as highlighted by both descriptive (Appendix B5a,b) and quantitative analyses (Appendix B6; S5). FFLs distributed along two gradients, one measuring the variation between strongly active (BT) and passive (PL) fishing gear, and other describing the variation in fishing selectivity of the segment (mono vs multigear, PTP vs PPG‐SSF, respectively) (Appendix B5a,b). Three different groups of segments can be discerned (Appendix B5a,b). The highest fishing effort segment (BT) was directly and strongly correlated with IFR_*X*_ (Appendix B5a,b) and showed higher values for LC and CR species compared with other IUCN categories (Appendix B6; S5). The intermediate fishing effort segments (PTP and PL) were inversely correlated with IFR_*X*_ (Appendix B5a,b) and showed higher values for the species contained within central IUCN risk categories (Appendix B6; S5) with respect to low‐ and high‐risk categories. The lowest fishing effort segments (PPG and SSF) were directly and well‐correlated with IERF_*X*_ (Appendix B5a,b) and exhibited higher values for the species having higher IUCN risk of extinction compared with others (Appendix B6; S5).

**FIGURE 4:**
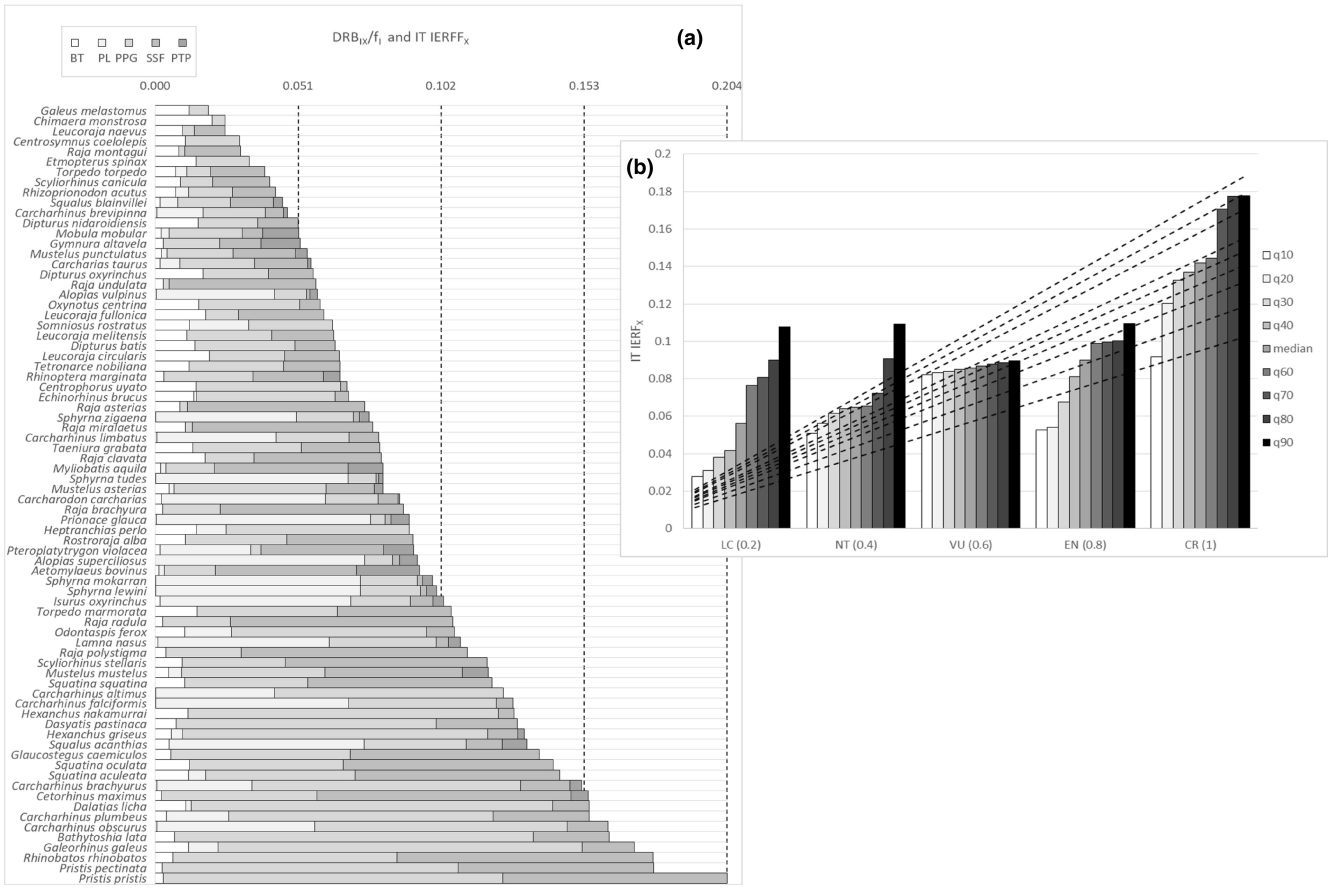
(a) Value distributions of IT IERF_*X*_ and DRB_*IX*_/f_*I*_ rates by species (*X* = 76 cartilaginous species recorded at the Italian scale) and fishing fleet segments (*I* = BT: bottom trawls; PL: pelagic longlines; PPG: passive polyvalent gears; SSF: small‐scale fishery; PTP: pelagic pair trawl and purse seine) at the Italian scale. DRB_IX_ is Disturbance Resilience Balance; IERF_*X*_ is Index of Extinction Risk to Fishing; f_*I*_ is fishing effort by fishing fleet segment. Species are ranked according to the increasing values of IERF_*X*_. (b). Percentile regression (deciles) of Index of Extinction risk to Fishing (IT IERF_*X*_) at the Italian scale along corresponding ranked IUNC red list categories (IT IUCN_*RX*_). Estimated linear models (dotted lines) are shown according to the general relation IT IERF_*X*_ = a*(IT IUCN_*RX*_).

**FIGURE 5:**
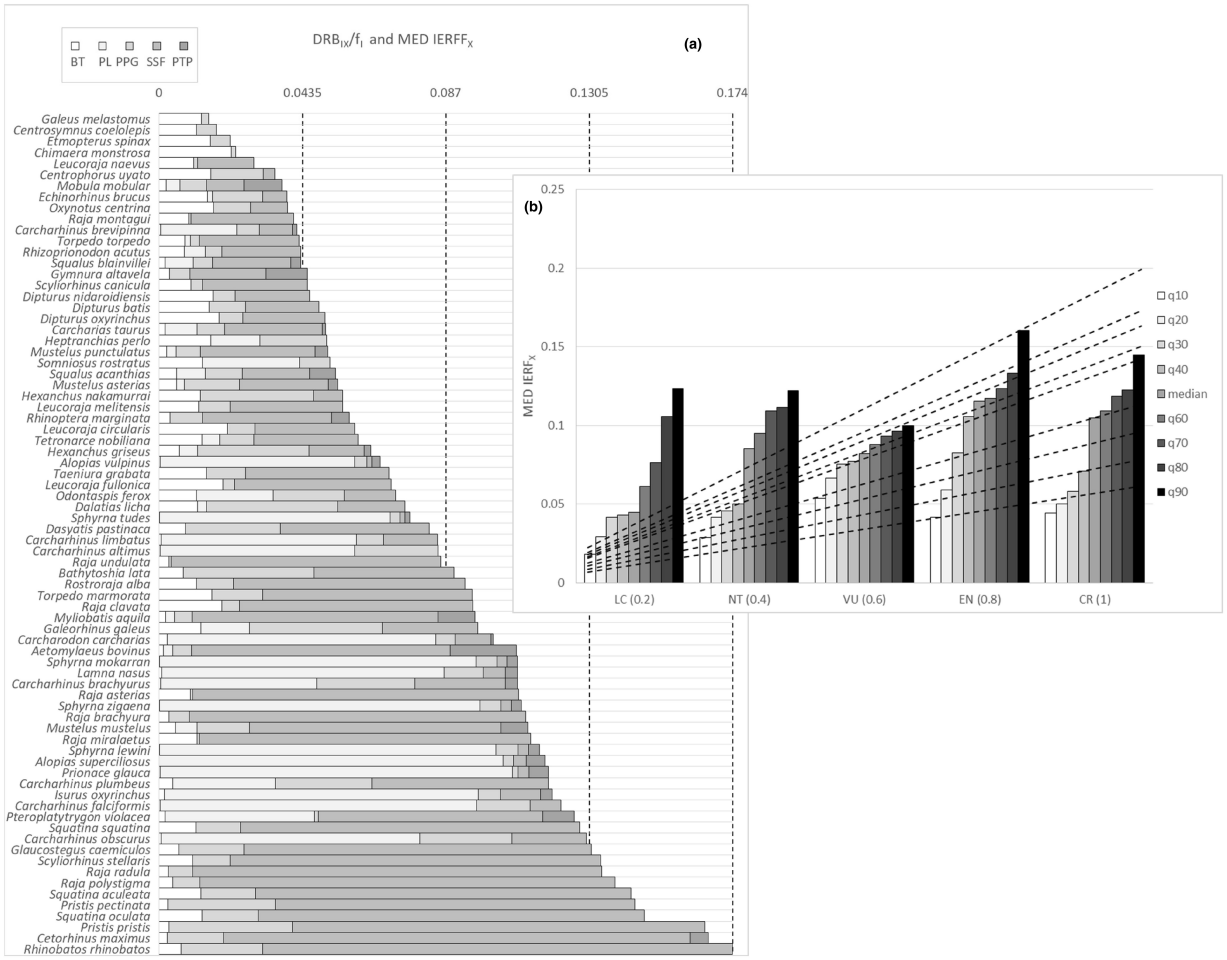
(a) Value distributions of MED IERF_*X*_ and DRB_*IX*_ /f_*I*_ rates by species (*X* = 76 cartilaginous species recorded at the Mediterranean scale) and fishing fleet segments (*I* = BT: bottom trawls; PL: pelagic longlines; PPG: passive polyvalent gears; SSF: small‐scale fishery; PTP: pelagic pair trawl and purse seine) at the Mediterranean scale. DRB_*IX*_ is Disturbance Resilience Balance; IERF_*X*_ is Index of Extinction Risk to Fishing; f_*I*_ is fishing effort by fishing fleet segment. Species are arranged according to the increasing values of IERF_*X*_. (**b**) Percentile regression (deciles) of Index of Extinction risk to Fishing (MED IERF_*X*_) at the Mediterranean scale along corresponding ranked IUNC red list categories (MED IUCN_*RX*_). Estimated linear models (dotted lines) are shown according to the general relation MED IERF_*X*_ = a*(MED IUCN_*RX*_).

As IT IERF_*X*_ and MED, IERF_*X*_ increased linearly with increasing IUCN extinction risk at the corresponding scales (Figures 4b and 5b, Appendix B7a), representing a species‐specific and cumulative index of extinction risk to fishing at the corresponding geographical scales (Figures 4a and 5a).

Nineteen and nine species resulted within the third and fourth quartiles of IERF_*X*_, respectively, the latter species appearing to suffer from the highest cumulative threat from almost all FFLs (Figure 4a). Twenty‐five and 10 species showed the same pattern at the Mediterranean scale (Figure 5a). Not threatened species had lower IERF_*X*_ than higher species of IUCN extinction risk, which showed values comparable with those of data‐deficient species, as classified by IUCN, at both scales.

**FIGURE 6:**
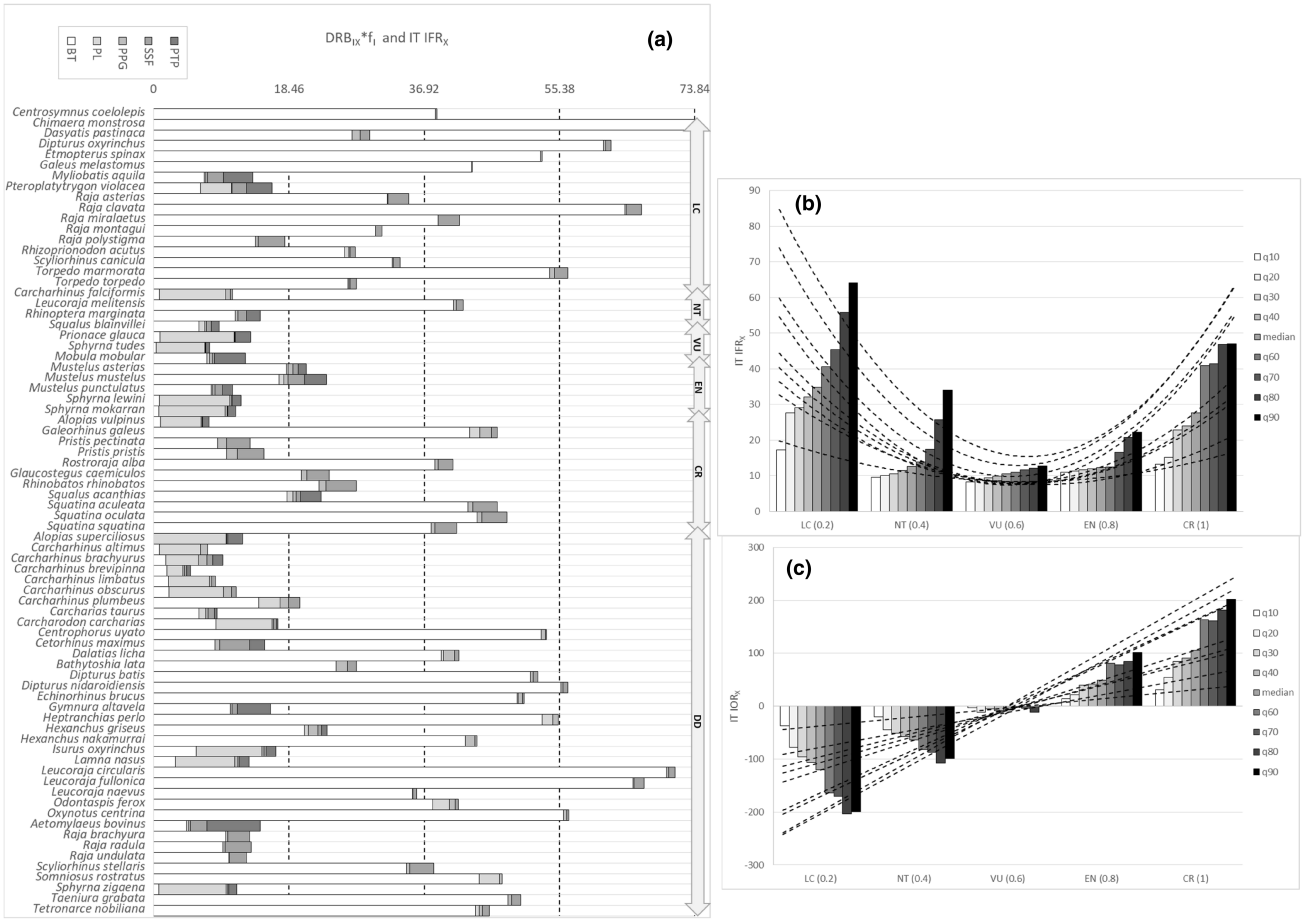
(a) Value distributions of IT IFR_*X*_ and DRB_*IX*_ *f_*I*_ products by species (*X* = 76 cartilaginous species recorded at the Mediterranean scale) and fishing fleet segments (*I* = BT: bottom trawls; PL: pelagic longlines; PPG: passive polyvalent gears; SSF: small‐scale fishery; PTP: pelagic pair trawl and purse seine) at the Italian scale. DRB_IX_ is Disturbance Resilience Balance; IFR_X_ is Index of Overfishing Response; f_*I*_ is fishing effort by fishing fleet segment. (b). Percentile regression (deciles) of Index of Overfishing Response (IT IFR_*X*_) along corresponding ranked IUNC red list categories (IT IUCN_*RX*_). Estimated parabolic models (dotted lines) are shown according to the general relation: IT IFR_*X*_ = a*(IT IUCN_*RX*_)^2^‐b* (IT IUCN_*RX*_) + c. (c) Representation of the estimated linear models describing relationships between the first derivative (IT IOR_*X*_ = IT 'IFR_*X*_ = 2*a* (IT IUCN_*RX*_)‐b) of the parabolic estimated function (IT IFR_*X*_) and IUCN ranked extinction risk categories at Italian scale.

**FIGURE 7:**
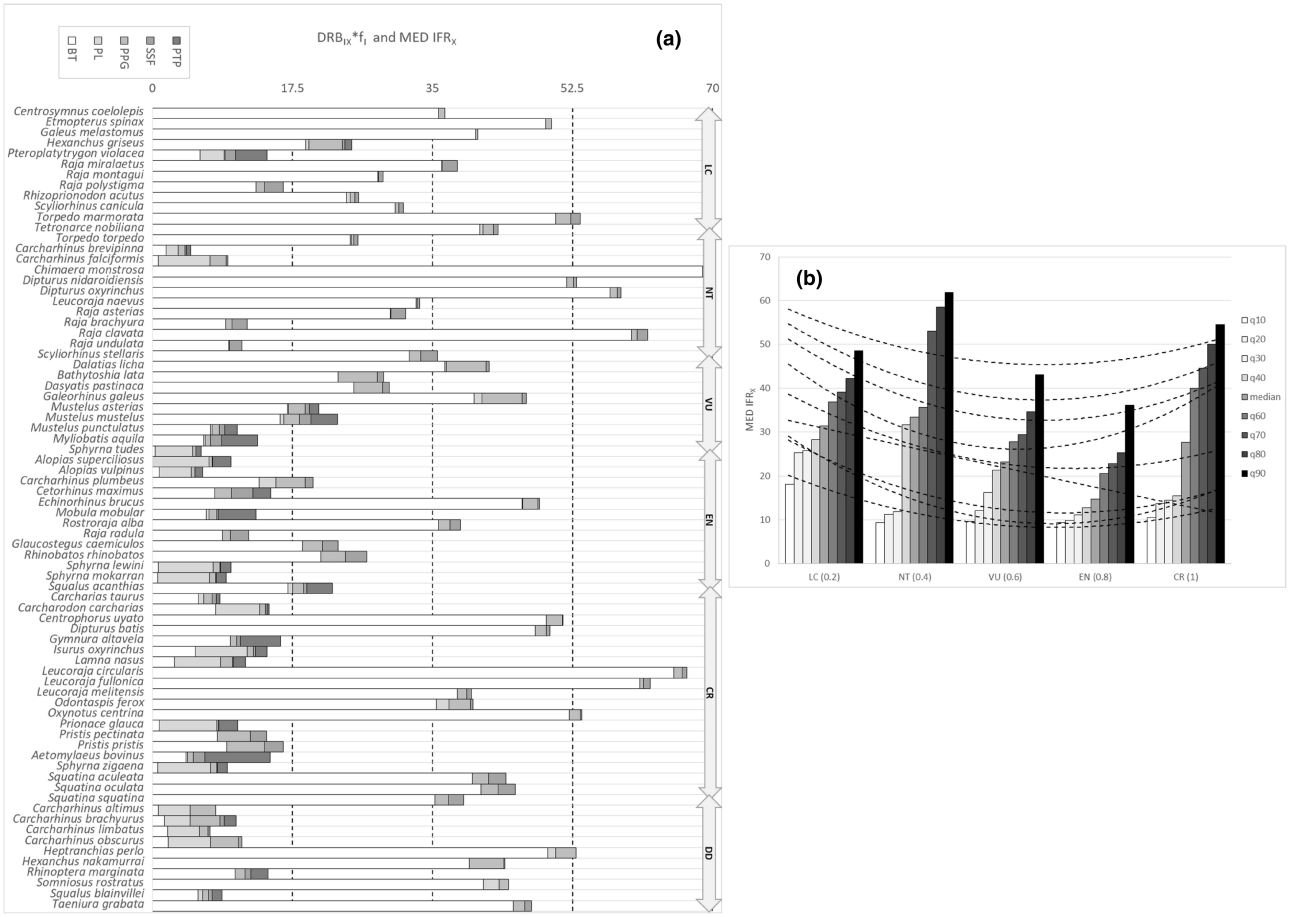
(a). Value distributions of MED IFR_*X*_ and DRB_*IX*_ *f_*I*_ products by species (*X* = 76 cartilaginous species recorded at the Mediterranean scale) and fishing fleet segments (*I* = BT: bottom trawls; PL: pelagic longlines; PPG: passive polyvalent gears; SSF: small‐scale fishery; PTP: pelagic pair trawl and purse seine) at the Mediterranean scale. DRB_*IX*_ is Disturbance Resilience Balance; IFR_*X*_ is Index of Fishing Response; f_*I*_ is fishing effort by fishing fleet segment. (b). Percentile regression (deciles) of Index of Overfishing Response (MED IFR_*X*_) along corresponding ranked IUNC red list categories (MED IUCN_*RX*_). Estimated parabolic models (dotted lines) are shown according to the general relation: MED IFR_*X*_ = a*(MED IUCN_*RX*_)^2^‐b* (MED IUCN_*RX*_) + c.

IFR_*X*_ indicated fishing threat by exhibiting a convex parabolic trend with increasing IUCN species extinction risk in particular at the Italian scale (Figures 6b and 7b, Appendix B7b); therefore, it represented a species‐specific and cumulative index of fishing response at Italian and, weekly, at the Mediterranean scale (Figures 6a and 7a). Referring to the first derivative of the estimated function (IOR_*X*_ = 'IT IFR_*X*_) on the Italian scale, species having positive values of IOR_*X*_ were considered as suffering from overfishing (Figure 6c) compared with species suffering from a sustainable fishing pressure (negative values).

According to GLM results and back‐verification, the couple of models including IERF_*X*_ and SW_(*/*)_QS_*X*_ contrasted better at the lesser than the larger scale (Appendix B8). Models involving IFR_*X*_ and SW_(*)_QS_*X*_ showed an opposite pattern, with the former contrasting on the smaller scale and the latter on the larger scale (Appendix B8). Therefore, the couple of models including IERF_*X*_ and IFR_*X*_ was found to be generally more effective at a smaller scale (IT, IUCN_*RX*_) than a larger one (MED and GLO IUCN_*RX*_); the couple of models involving SW_(*/*)_QS_*X*_ and SW_(*)_QS_*X*_ produced better geographically focused predictions than IERF_*X*_ and IFR_*X*_ (Appendix B8).

## 4 DISCUSSION

### 4.1 Expert knowledge

Although expert estimates are generally self‐constrained (Ip et al., 2011) and different from each other (McBride et al., 2012), interexpert variation contains the most information (Ip et al., 2011), both when the opinions of both experts tend to converge and when they do not (Humphreys et al., 2009; Johnson & Gillingham, 2004). From a different perspective, the level of subjectivity of experts' knowledge is at the core of the data (O'Hagan, 2019), when their processing methods take into account the variability of information and the data can be verified (Ip et al., 2011).

This applies well to the present case when comparing the degree of subjectivity in the probability of fatal capture between groups A and B. In fact, uncertainty was greatest where ecological diversity in swimming function was greatest (Portnoy & Heist, 2012), that is, in Group B species. Although these are more benthic and less mobile species (WoRMS Editorial Board, 2020), the group also includes several bent‐pelagic and pelagic‐adapted species (Tortonese, 1956). Group A consists of a collection of more mobile, demersal, and pelagic species (Camhi et al., 1998), with fewer true slow‐swimming benthic species (Scacco et al., 2010). Experts perceived this ecological diversity, as evidenced by their estimates. Group B species are inherently less predictable in their interaction with fishing gear than Group A species.

The expert elicitation method presented here enhanced interexpert differences by both considering the variability of estimates and adequately buffering their range of variation (Ip et al., 2011; Morgan & Henrion, 1990; Vercammen & Burgman, 2019). The variability in estimates is likely related to the different perceptions experts had of (1) gear selectivity and species‐specific interaction (Millar & Fryer, 1999; Stewart, 2002; Young et al., 2019) and (2) level of overlap between fishing areas and species density and distribution (Kroodsma et al., 2018; Murua et al., 2021; Queiroz et al., 2019; Williamson et al., 2020). The structuring of the elicitation question compensated for both number one, by “averaging” the fishing selectivity of the different gears co‐used at the segment level, and number two, by roughly stratifying over the two fishing areas (inshore and offshore). The fleet segmentation used for the analyses is thus representative of the real situation, as it is almost in line with that officially used in EU waters (JRC, 2020) under the Common Fisheries Policy (EU Council Regulation, 2006). In addition, the range of variation in expert estimates was buffered normalizing the species risk assessment by both species group and segment, thus allowing comparability across species and segments.

The early stages of the expert knowledge elicitation protocol (expert selection, preparation of invitation formats, and structuring of the elicitation question and context) used in this work were largely inspired by the guidance provided by Burgman (2015). An initial modification involved a smaller number of experts used, compared with that suggested to achieve the crowd wisdom effect (Morgan, 2014). Our results suggest that a random selection of a minimum number of experts (three of 10 with an average, homogeneous profile) may represent an average combination of reliable expert knowledge, as demonstrated by the metrics derived from the estimates from the comparison with the IUCN red lists. Indeed, assuming that experts individually have different abilities to provide estimates along species and segments, we might expect the random probability of intercepting the best or worst combination of experts in estimation ability to be very low (D_10; 3_ = (n!)/(n–k)! = 0.008), as when drawing simultaneously from the same pool. Random selection of three experts out of an a‐priori choice of 10 profiled means in this case referring to about one‐third of the total, that is, having a much higher probability of intercepting an average combination of experts between the best and the worst. This result supports the idea that the availability of potential experts can be expanded, thus reducing search time and improving replicability, particularly when applied to smaller geographical scale (i.e., subregional and local scales). However, the future effort will be to replicate the experiment with a larger number of experts than in the current case, allowing comparison with the current results and their final validation. In addition, future work will include experts other than academia, as different perspectives and experiences on species–gear interactions are of great value in risk assessment for fisheries. This will allow comparisons of assessments within and among the different types of experts selected (fishermen, stakeholders, coast guards, and patrol forces).

The most important innovations contained in the method concern the framework we used to obtain the best estimates and assign the uncertainties associated with them. Based on a generalization of the Uniqueness Quantification Theory (Andrews, 2002; Kleene, 1952), an expert, called upon to provide a simultaneous evaluation of multiple dependent quantities that sum to one, is expected to return his or her estimates as singletons. The latter are uniquely defined objects because, due to a rule that binds them, they are bounded by each other (Stoll, 1961; Whitehead & Russell, 1910). Because we used a multivariate approach during the present experiment, the use of singletons allowed us to circumvent the axiom that a valid estimate by an expert must be an interval (de Finetti, 1937), particularly in univariate experiments, for which a multistep approach to defining the interval works best to reduce estimation overconfidence (Speirs‐Bridge et al., 2010). In addition, the proposed method partially reduced the biases arising from anchoring and under‐ and overconfidence (O'Hagan, 2019; Tversky & Kahneman, 1974) because the experts had time (Kahneman, 2011) to present the most accurate estimate possible, which was inherently a single estimate. They were able to independently refine their estimates by comparing them along segments before providing us with the data.

Overall, this approach shifted the problem of considering uncertainty related to multiple dependent quantities adding up to one from the experts to the elicitors. This required the elicitors to find appropriate and representative intervals that accounted for uncertainty around the best estimates. The mother distributions, as we called them, offer the possibility of assigning lower and upper bounds to the experts' estimates using their quartiles. This approach can find support in the fact that the best estimates are singletons, whose uncertainty may uniquely reside in a series of n replications spaced over time by the same expert. Species estimates along fishing segments are most likely expected to be roughly similar or even equal, that is, the best initial estimates have a high probability of being contained within the quartiles of the mother distributions to which they belong. The mathematical aggregation used to combine expert judgments (i.e., linear combination; Cooke, 1991) is simplified by averaging over quartiles (Lichtendahl et al., 2013). Unlike the classical model that averages over probability densities (Cooke & Goossens, 2008), averaging over quartiles produces distributions that are more concentrated around the median, thus reducing the supposed problem of large uncertainty distributions in combined ratings (Lichtendahl et al., 2013).

During the whole experimental process, the experts worked independently and did not interact with each other. The completely independent estimates (Rowe & Wright, 1999) were produced in a single round similarly to a minimum assessment and analyzed considering the experts having the same weight, as opposed to weighing the experts (Cooke, 1991). The method was then developed in line with the one developed for multiple dependent quantities whose sum must be 1 (Oakley & O'Hagan, 2016; O'Hagan, 2019).

In general, the method shortened the elicitation path compared with the main protocols (Gosling, 2018; Hemming et al., 2018; Oakley & O'Hagan, 2016; Rowe & Wright, 1999), particularly in those using behavioral aggregation (Gosling, 2018; Hemming et al., 2018). The sources of bias in the present method (lack of agreement among experts and higher probability of error) are that expected symmetrically with respect to the biases of behavioral approaches (biased and dependent estimates, conditional overall assessment) (Gosling, 2018; Hemming et al., 2018). The case of a triangular distribution better explained by Morgan and Henrion (1990) and in Kotz and Van Dorp (2004) support the results obtained, presented and discussed in this work.

The method suggests a simple and quick, but tentative, solution to deal with the intricate framework of processing multiple dependent quantities that add up to one by multiple experts. As it is actually a debated issue among experts in the field, elicitation of multiple quantities by multiple experts still has room for creative but rigorous solutions (O'Hagan, 2019) and simpler frameworks should be preferred (Stefan et al., 2020).

### 4.2 Method for risk assessment of species by fishing segments

#### 4.2.1 Qualitative level

Based on expert knowledge, the method developed for risk assessment offers a dual description of the fishery threat, both qualitative and quantitative.

Graphical representations made it easy to visualize the level of risk to species associated with a given segment, exploring the overall fishing risk in different FFLs and species divided by two major morphological groups. Such information can allow comparison between and within segments, thus helping to prioritize conservation actions. The qualitative scenario found is supported by data from the main literature. According to the current results, one‐third of BT target species are at high risk. The bottom trawl (BT) segment, which uses gear that varies in the level of direct interaction with the seafloor (FAO, 2016; Ferretti, 1981), targets almost all species, as it extensively and heavily impacts marine fauna and cartilaginous fish (Biton Porsmoguer & Lloret, 2020; Carbonell et al., 2003; Scacco et al., 2002; Stobutzki et al., 2002), due to its lack of selectivity. The PTP targeted the fewest number of species; however, more than half are classified as intermediate‐high and high risk. Many of these species have been recorded in the catch of pelagic trawlers (Fortuna et al., 2010; Scacco et al., 2009) and purse seiners (Amande et al., 2008). PL was found to be the main threat to sharks and pelagic rays, with about one‐third classified as high risk. It is well‐established that this segment produces major impacts on pelagic shark species in the world's oceans (Beerkircher et al., 2002; Gallagher et al., 2014; Gilman et al., 2008), including areas in the Mediterranean (Megalofonou et al., 2005). Pelagic longlines are medium‐to‐large vessels licensed to fish in the high seas (FAO, 2016; JRC, 2020), with a wide variation in fishing selectivity depending on local use and gear arrangement (Cosandey‐Godin & Morgan, 2011 and references therein), making them similar to a multigear segment. Representing a significant threat in the preferred environment of several pelagic species (Kroodsma et al., 2018; Queiroz et al., 2019), PL is co‐responsible for the collapse of their populations in the Mediterranean (Ferretti et al., 2008; Moro et al., 2019). Passive polyvalent gears has affected all species, with two‐thirds of the total number of species classified as intermediate‐high and high risk. Passive polyvalent gear segment is a very heterogeneous fishing segment, consisting of large vessels (>12 m), which generally fish in deep‐sea waters (>12 Nm), using mainly set gillnets, trammel nets, and demersal longlines (JRC, 2020). These gears tend to catch large numbers of sharks and rays (Benjamins et al., 2010; Perez & Wahrlich, 2005; Thorpe & Frierson, 2009), as do drift gillnets (McKinnell & Seki, 1998; Tudela et al., 2005), demersal longlines (Coelho et al., 2005; Valenzuela et al., 2008), and demersal trammel nets (Coelho et al., 2005; Scacco et al., 2012). The SSF showed a significant number of estimated intermediate‐high and high‐risk species, accounting for nearly two‐thirds of the total. Small‐scale fishery commonly uses low‐effort passive fishing gears (boat length < 12 m) but also low‐effort active and semi‐active gears (i.e., small trawlers and purse seines; FAO, 2016; JRC, 2020) in coastal areas (fishing area < 12 Nm) (JRC, 2020; Lloret et al., 2019; Smith & Basurto, 2019). Despite the increased selectivity of individual fishing gears and reduced effort, SSFs can have direct and indirect impacts on coastal‐dependent Chondrichthyes (Roff et al., 2018; Temple et al., 2018). The marked interchangeability in the use of different fishing gears lowers the average selectivity of this segment (Smith & Basurto, 2019), making it a very serious threat to several cartilaginous species; for example, directed fishing in mating, nursery, or foraging areas (Heupel et al., 2007; Kinney & Simpfendorfer, 2009; Ward‐Paige et al., 2014). Many of these species are found to be in critical condition (Dulvy et al., 2016; Pacoureau et al., 2021).

#### 4.2.2 Quantitative level

Quantitative information is embodied in the cumulative metrics, which were found to be reliable due to the correlation found with IUCN red lists in the metrics verification framework that included fishing effort in the analyses.

Because it was based on ecological interpretations of species resilience and disturbance (Grimm & Wissel, 1997; Holling, 1996; Walker et al., 2004), the method first provided an intrinsic measure of species response to different fishery threats, beyond applied fishing effort, such as DRB_*IX*_. This metric measures the response of species to the “average selectivity” that each FFL shows toward the species based on the fishing gear used. For example, the DRB_*IX*_ revealed the existence of three groups of FFLs, based on the threat patterns they exert on species. Species with the highest IUCN extinction risk were particularly affected by multiple gear segments (i.e., PPG and SSF). By contrast, single‐gear FFLs (i.e., BT and PTP) affected species according to a complementary quadratic pattern. PL fell somewhere between single and multigear segments, affecting both high‐ and low‐threatened species depending on the geographic scale. This scenario was confirmed when importing fishing effort into the analyses through cumulative metrics such as IERF_*X*_ and IFR_*X*_. The two metrics were able to distinguish the effect of disturbance on species in relation to their resistance and resilience properties.

On the one hand, IERF_*X*_ represents an index of species‐specific extinction risk relative to overall fisheries. In fact, it differentiates species based on their overall resistance to the corresponding overall disturbance (Walker et al., 2004). In a fishing context, the IERF_*X*_ approximates the CPUE (Ricker, 1975), being a measure of the response of species to an effort unit as a function of their resistence to fishing, that is, the average probability that an individual will be alive or dead from capture. Being ratio‐based, the IERF_*X*_ indicates that multigear FFLs at low effort are likely to represent more intense pulses and disturbances for cartilaginous fish species.

On the other hand, IFR_*X*_ represents a species‐specific index of response to fishing. In fact, it differentiates species based on their overall resilience to the corresponding overall disturbance (Walker et al., 2004). The IFR_X_ relates to the concept of fishing mortality (Sparre et al., 1989) as a proxy for the species' response to effective fishing efforts (Froese et al., 2017). Being based on product, the IFR_*X*_ indicated that high‐effort, single‐gear FFLs, such as BT and PTP, represent constant disturbances. In fact, these segments operate with fewer but larger vessels throughout the year, with very high effort in traditional fishing grounds (De Angelis et al., 2020; Russo et al., 2019).

The convex parabolic trend of the metric with increasing IUCN extinction risk illustrates the different responses species may have with respect to their ability to respond to numerical depletion. This capacity is a function of the level of IUCN extinction risk that the species is at (Froese et al., 2017; Walker et al., 2004). Reference to the first derivative of the estimated function (IOR_X_) allowed differentiation between species that are or are not in an overfished state (positive and negative values, respectively), with a linear relationship with IUCN_RX_, and parallel, as VU is the first of the threatened (and overfished) categories.

The metrics are designed to prioritize species for conservation actions. For example, species in the third and, especially, fourth quartiles of IT and MED IERF_*X*_ suffer from intermediate to high cumulative fishing threats from several or all FFLs. Finally, the estimates for several data‐deficient species obtained from this study were comparable with those for the species assessed as critically endangered and other threatened IUCN categories. The metrics provided a measure of the actual threat faced by these species, as already proposed on a larger scale by Walls and Dulvy (2020) and Pacoureau et al. (2021).

### 4.3 Comparison among scales and replicability

The consistency of quantitative results between the two different scales considered underscores the replicability of the method. In fact, it highlights the rationale behind the method, which is to provide an easy‐to‐replicate, fast and flexible method that maximizes the available data (estimates, actual FFL and associated fishing effort, species occurrence) at a defined spatial and temporal scale. Temporal consistency among the datasets used for risk assessment is also important and was considered in the framework of this paper. For example, IVF_*X*_ estimates are constantly updated based on the increasing knowledge gathered about species bio‐ecology over time (FishBase, 2021). Reference to coeval data of fishing effort would also help ensure that risk assessments for species are closer to the real‐time situation, as done in the development of quantitative metrics.

The present results demonstrated more reliable differentiation on the smaller (Italian) scale than on the larger (Mediterranean) scale. They also showed that the use of effort‐based metrics as a rate (IERF_*X*_ and SQ_(*/*)_Q_*X*_) was statistically more robust than the use of product‐based metrics (IFR_*X*_ and SQ_(*)_Q_*X*_).

The observed interscalar differences depended on dissimilarities in both IUCN species risk assessments (Dulvy et al., 2016; Rondinini et al., 2013) and fishing effort of the corresponding fleets considered at the two scales. For example, IUCN assessments at the Mediterranean scale had fewer DD species and appeared to be assessed at a higher extinction risk, compared with the Italian assessment. Further differences could result from the different relative weights given to threats other than fisheries in the baseline IUCN series (Dulvy et al., 2016; Rondinini et al., 2013). Indeed, they take into account an overall extinction risk, unlike the metrics developed that consider only the threat from fisheries. Finally, a lower accuracy of the fishery data for the Mediterranean scale cannot be ruled out, as it included data from all European countries bordering the Mediterranean and Black Sea.

A basic method, such as the one discussed so far, which is easy, quick, and at the same time reliable, will also promote a local and participatory approach to risk assessment associated with the bycatch of sharks, rays and chimaeras (Booth et al., 2019; Colloca et al., 2017; Hind, 2015). In this direction, further efforts will be needed to validate the method by involving not only academics but also other stakeholders in species risk assessment for fisheries. Diverse expert knowledge on a common topic will improve the potential for achieving species conservation goals.

## 5 CONCLUSIONS

With the present work, our results suggested that the use of the least expert scientific knowledge, coupled with a simple risk assessment to fishing, has the ability to buffer and weight the complexity of the data while acceptably reducing the accuracy of assessments. The methods tested on 76 Mediterranean Chondrichthyes on a fleet segment scale provided fishery segment‐related risk graphs that distinguished the fishing threat patterns of the species studied. Verification of the quantitative metrics used revealed a dual and reliable interpretation of cumulative fishery threat, particularly at finer scales. The replicability of the proposed framework may significantly improve the species risk assessments at the local level. It can accelerate decisions research and conservation priorities to correctly manage and protect marine species, such as adopting more accurate mitigation measures for species based on the composition and use of fishing gear in local fishing segments. The flexibility allows it to be applied not only to Elasmobranchii and Holocephali, but also to other marine species of conservation concern. Protection of locally distributed and migratory species would benefit from conservation efforts performed at multiple local scales, preserving the remaining refugia and marine species from our mistakes.

## AUTHOR CONTRIBUTIONS

**Umberto Scacco:** Conceptualization (lead); data curation (lead); formal analysis (lead); investigation (lead); methodology (lead); resources (lead); software (lead); supervision (lead); validation (lead); visualization (lead); writing – original draft (lead); writing – review and editing (lead). **Simone Di Crescenzo:** Writing – review and editing (equal). **Alice Sbrana:** Formal analysis (equal); methodology (equal); software (equal); writing – review and editing (equal).

## FUNDING INFORMATION

None.

## CONFLICT OF INTEREST STATEMENT

The work is all original research carried out by the authors, which agree with the content of the manuscript and its submission to Your journal. No part of the research has been published in any form elsewhere, neither it is being considered for publication elsewhere while it is being considered for publication in Ecology and Evolution. Any research in the paper not carried out by the authors is fully acknowledged and authors declare any direct financial benefit that could result from publication, neither specific founding was provided to carry out the work. All appropriate ethics and other approval were obtained for the research.

## Supporting information


Data S1.

Data S2.

Data S3.

Data S4.

Data S5.

Appendix S1.

Images S1.

## Data Availability

Data availability statement: All data related to fishing effort at the two studied scales and statistical details of the elicitation framework implemented are available at Dryad, Dataset: “Fishing effort data, fishing fleet segmentation, and statistical details used in the expert knowledge elicitation experiment,” https://doi.org/10.5061/dryad.8cz8w9gw4.
