# Supplementary material for: Exploring fishing threat at fleet segment and subregional scale: Least expert knowledge and a resilience versus disturbance‐based approach as conservation's tools for cartilaginous fish
Source: Ecol Evol. 2023 Mar 19;13(3):e9881. doi: 10.1002/ece3.9881 (PMC10025082; doi:10.1002/ece3.9881)
Supplement: Supplementary file 1 — Data S1. [file ECE3-13-e9881-s002.pdf]

S1

S6: Inclusion and exclusion criteria used to aggregate different fishing gear into fishing fleet segments

List of fishing gears codes excluded	List of fishing gears codes included	vessel lenght		Fishing fleet segments
FPO	PS	> 12m	< 12m	BT
FPN	LA	> 12m	< 12m	PPT
FYK	SB >12 m	> 12m	< 12m	SSF
DRB	SV>12m	> 12m	< 12m	PPG
NK	TBB	> 12m	< 12m	PL
NO	OTB	> 12m	< 12m	
HMD	OTT	> 12m	< 12m	
SPR	PTB	> 12m	< 12m	
LNB	OTM	> 12m	< 12m	
SDN	PTM	> 12m	< 12m	
	GNC	> 12m	< 12m	
	GND	> 12m	< 12m	
	GNS	> 12m	< 12m	
	GTN	> 12m	< 12m	
	GTR	> 12m	< 12m	
	LHM	> 12m	< 12m	
	LHP	> 12m	< 12m	
	LLD	> 12m	< 12m	
	LLS	> 12m	< 12m	
	LTL	> 12m	< 12m	

Author:
For gears codes and a short description of the gears please visit http://www.fao.org/3/a-bt988e.pdf

Author:
Same colour indicates association between a fishing gears and fishing segments.
