# Supplementary material for: Exploring fishing threat at fleet segment and subregional scale: Least expert knowledge and a resilience versus disturbance‐based approach as conservation's tools for cartilaginous fish
Source: Ecol Evol. 2023 Mar 19;13(3):e9881. doi: 10.1002/ece3.9881 (PMC10025082; doi:10.1002/ece3.9881)
Supplement: Supplementary file 2 — Data S2. [file ECE3-13-e9881-s001.pdf]

S2

S2: Panel reporting the check for the best distributions fitting response variables used in GLMA. Capital letters inside the graphs indicate variables. A: IT IERFX, B: IT IFRX, C: IT SQ(/)QX, D: IT SQ(*)QX, E: IT MED IERFX, F: MED IFRX, G: MED SQ(/)QX, H: MED SQ(*)QX and J: IVFX

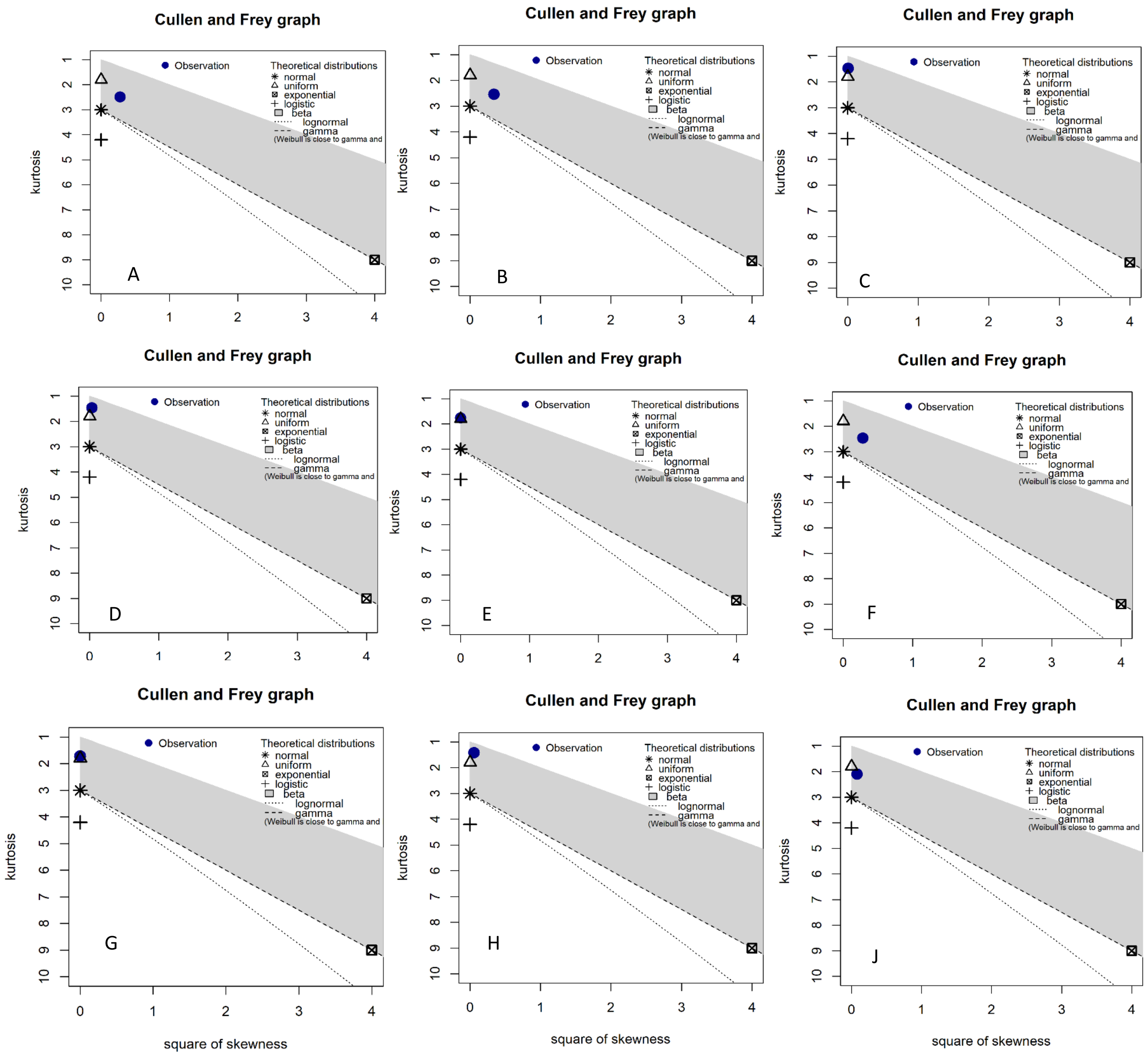
Cullen and Frey graph
Observation
Theoretical distributions
normal
uniform
exponential
logistic
beta
lognormal
gamma
(Weibull is close to gamma and
kurtosis
A
Cullen and Frey graph
Observation
Theoretical distributions
normal
uniform
exponential
logistic
beta
lognormal
gamma
(Weibull is close to gamma and
kurtosis
B
Cullen and Frey graph
Observation
Theoretical distributions
normal
uniform
exponential
logistic
beta
lognormal
gamma
(Weibull is close to gamma and
kurtosis
C
Cullen and Frey graph
Observation
Theoretical distributions
normal
uniform
exponential
logistic
beta
lognormal
gamma
(Weibull is close to gamma and
kurtosis
D
Cullen and Frey graph
Observation
Theoretical distributions
normal
uniform
exponential
logistic
beta
lognormal
gamma
(Weibull is close to gamma and
kurtosis
E
Cullen and Frey graph
Observation
Theoretical distributions
normal
uniform
exponential
logistic
beta
lognormal
gamma
(Weibull is close to gamma and
kurtosis
F
Cullen and Frey graph
Observation
Theoretical distributions
normal
uniform
exponential
logistic
beta
lognormal
gamma
(Weibull is close to gamma and
kurtosis
G
square of skewness
Cullen and Frey graph
Observation
Theoretical distributions
normal
uniform
exponential
logistic
beta
lognormal
gamma
(Weibull is close to gamma and
kurtosis
H
square of skewness
Cullen and Frey graph
Observation
Theoretical distributions
normal
uniform
exponential
logistic
beta
lognormal
gamma
(Weibull is close to gamma and
kurtosis
J
square of skewness
