# Supplementary material for: Exploring fishing threat at fleet segment and subregional scale: Least expert knowledge and a resilience versus disturbance‐based approach as conservation's tools for cartilaginous fish
Source: Ecol Evol. 2023 Mar 19;13(3):e9881. doi: 10.1002/ece3.9881 (PMC10025082; doi:10.1002/ece3.9881)
Supplement: Supplementary file 3 — Data S3. [file ECE3-13-e9881-s006.pdf]

SUPPORTING INFORMATION 3 (S1): DIRICHELET FITS OF COMBINED EXPERTS'ESTIMATES FOR THE SPECIES CONSIDERED IN THE ELICITATION FRAMEWORK

Dirichelet fits...p. 3

Etmopterus spinax...pp. 4-5

Mustelus punctulatus...pp. 5-6

Carcharhinus limbatus...pp. 7-8

Carcharias taurus...pp. 8-9

Sphyrna tudes...pp. 10-11

Galeus melastomus...pp. 11-12

Rhizoprionodon acutus...pp. 13-14

Carcharhinus brevipinna...pp. 14-15

Scyliorhinus canicula...pp. 16-17

Centrosymnus coelolepis...pp. 17-18

Squalus blainvillei...pp. 19-20

Alopias vulpinus...pp. 20-21

Squalus acanthias...pp. 22-23

Mustelus asterias...pp. 23-24

Heptranchias perlo...pp. 25-26

Galeorhinus galeus...pp. 26-27

Mustelus mustelus...pp. 28-29

Carcharhinus altimus...pp. 29-30

Isurus oxyrinchus...pp. 31-32

Odontaspis ferox...pp. 32-33

Somniosus rostratus...pp. 34-35

Prionace glauca...pp. 35-36

Alopias superciliosus...pp. 37-38

Carcharhinus falciformis...pp. 39-40

Oxynotus centrina...pp. 40-41

Dalatias licha...pp. 42-43

Hexanchus nakamurrai...pp. 43-44

Sphyrna lewini...pp. 45-46

Scyliorhinus stellaris...pp. 47-48

Echinorhinus brucus...pp. 48-49

Hexanchus griseus...pp 49-50

36 *Sphyrna zigaena*......pp. 51-52

37 *Carcharodon carcharias*......pp. 52-53

38 *Cetorhinus maximus*......pp. 54-55

39 *Lamna nasus*......pp. 55-56

40 *Sphyrna mokarran*......pp. 57-58

41 *Carcharhinus brachyurus*......pp. 58-59

42 *Centrophorus uyato*......pp. 60-61

43 *Carcharhinus obscurus*......pp. 61-62

44 *Carcharhinus plumbeus*......pp. 63-64

45 *Leucoraja melitensis*......pp. 64-65

46 *Raja polystigma*......pp. 66-67

47 *Rhinoptera marginata*......pp. 67-68

48 *Raja asterias*......pp. 69-70

49 *Raja radula*......pp. 70-71

50 *Gymnura altavela*......pp. 72-73

51 *Raja miralaetus*......pp. 73-74

52 *Mobula mobular*......pp. 75-76

53 *Aetomylaeus bovinus*......pp. 76-77

54 *Myliobatis aquila*......pp. 78-79

55 *Raja brachyura*......pp. 79-80

56 *Raja montagui*......pp. 81-82

57 *Leucoraja naevus*......pp. 83-84

58 *Raja undulata*......pp. 84-85

59 *Torpedo torpedo*......pp. 86-87

60 *Pteroplatytrygon violacea*......pp. 87-88

61 *Leucoraja circularis*......pp. 89-90

62 *Leucoraja fullonica*......pp. 90-91

63 *Rhinobatos rhinobatos*......pp. 92-93

64 *Torpedo marmorata*......pp. 93-94

65 *Raja clavata*......pp. 95-96

66 *Squatina oculata*......pp. 96-97

67 *Chimaera monstrosa*......pp. 98-99

68 *Pristis pristis*......pp. 99-100

69 *Squatina aculeata*......pp. 101-102

70 *Dipturus oxyrinchus*......pp. 102-103

Taeniura grabata..pp. 104-105
Bathytoshia lata..pp. 105-106
Dasyatis pastinaca..pp. 107-108
Rostroraja alba..pp. 108-109
Dipturus nidaroidiensis..pp. 110-111
Glaucostegus caemiculos..pp. 111-112
Squatina squatina..pp. 113-114
Dipturus batis..pp. 114-115
Pristis pectinata..pp. 116-117
Tetronarce nobiliana..pp. 117-118

2.4A Dirichelet fit

The following sheets (1 to 76) reported comparison between directly elicted beta marginal and beta marginal distributions from Dirichlet fit, separately for each of the considered species along fishing segments (A: bottom trawl segment; B: pelagic longlines; C: passive polyvalent gears; D: small scale fishery; E: pelagic trawls + purse seines). The sum of elicited marginal means (figure below) ranged between 0.818 and 1.401 following a tight normal distribution (Chi-Squared = 3.46, d.f = 2, p = 0.18) with a mean of 1.005 and a variance of $9.7*10^{-3}$ This results suggests that the species-specific Dirichelet multinomial distributions fitted at best the corresponding aggregated estimates per fishing segment provided by the experts.

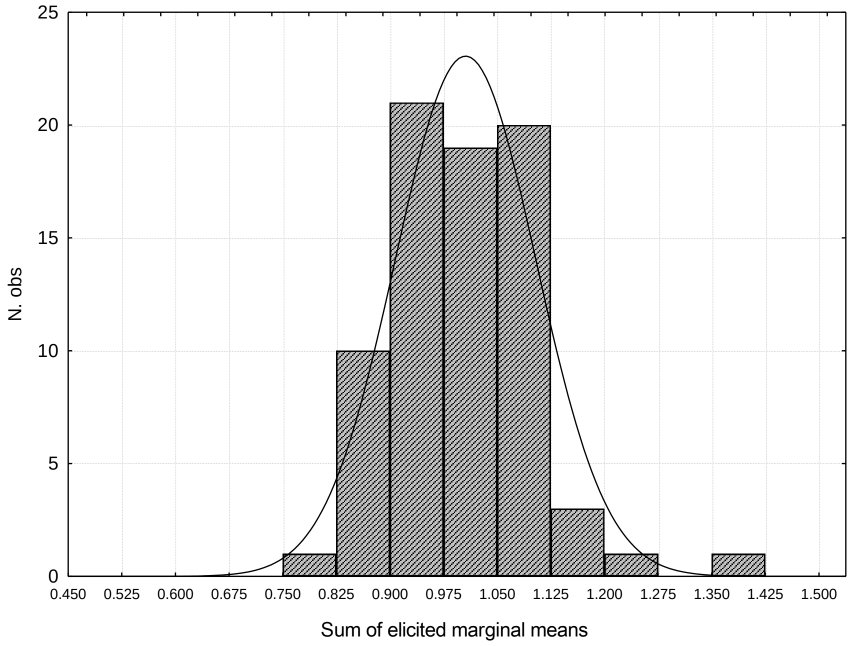

Etmopterus spinax

15 December 2021, 10:24

Elicited judgements

quantiles	A	B	C	D	E
0.25	0.7107	1e-04	0.0853	1e-04	1e-04
0.50	0.8557	2e-04	0.1183	2e-04	2e-04
0.75	0.9033	3e-04	0.1663	3e-04	3e-04

Dirichlet density function and parameters

Define the vector of unknown population proportions as

$$\theta := (\theta_1, \ldots, \theta_k),$$

with $= 5$. We write

$$\theta \sim Dirichlet(a_1, \ldots, a_k),$$

with

$$f(\theta) = \frac{\Gamma(a_1 + \cdots + a_k)}{\Gamma(a_1) \ldots \Gamma(a_k)} \prod_{i=1}^{k} \theta_i^{a_i - 1}.$$

The fitted parameter values $a_1, \ldots, a_k$ are as follows:

```
##          A          B          C          D          E
## 9.94884594 0.00284502 1.58863704 0.00284502 0.00284502
```

Comparing the elicited marginals with the marginals from the Dirichlet fit

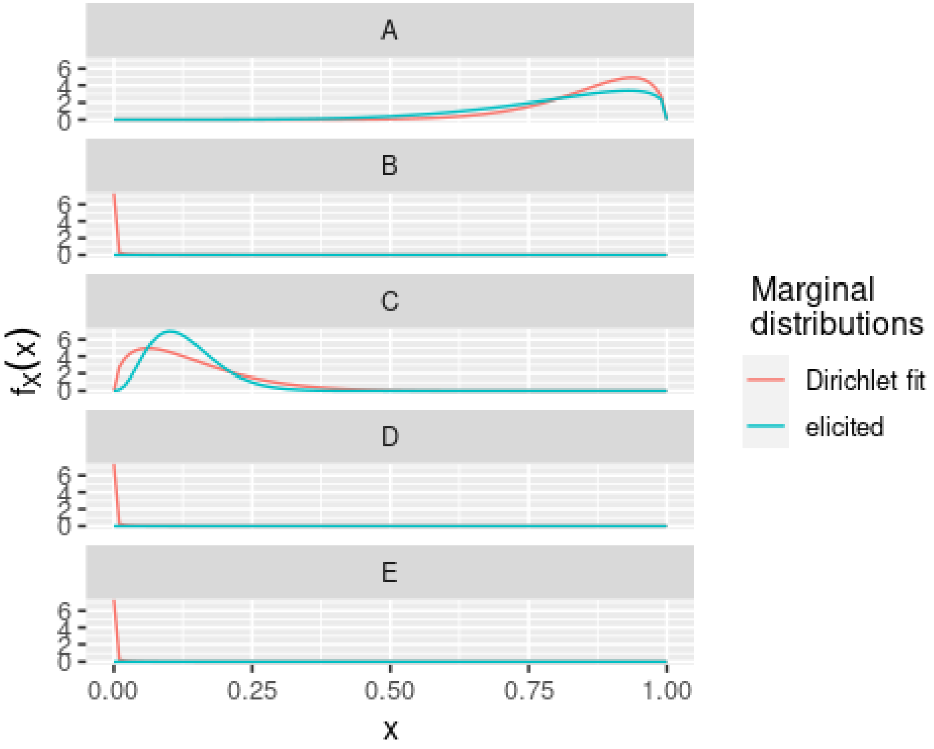

```
##
##       Directly       elicted       beta       marginal       distributions:
##
##                        A              B            C             D            E
##      shape1      5.530      1.80e+00          3.8300      1.80e+00      1.80e+00
##       shape2       1.330       7.82e+03       25.9000       7.82e+03      7.82e+03
##     mean              0.806      2.30e-04         0.1290      2.30e-04      2.30e-04
##    sd                    0.141     1.72e-04         0.0604      1.72e-04      1.72e-04
##     sum                  6.870      7.82e+03      29.7000      7.82e+03      7.82e+03
##
##       Sum        of        elicited        marginal        means:        0.935
##
##      Beta      marginal      distributions       from       Dirichlet       fit:
##
##                          A             B            C             D            E
##     shape1          9.9500      2.85e-03         1.5900      2.85e-03      2.85e-03
##     shape2          1.6000      1.15e+01         9.9600      1.15e+01      1.15e+01
##    mean                0.8620      2.46e-04         0.1380      2.46e-04      2.46e-04
##    sd                     0.0975     4.43e-03         0.0973      4.43e-03      4.43e-03
## sum    11.5000 1.15e+01 11.5000 1.15e+01 1.15e+01
```

Mustelus punctulatus

15 December 2021, 10:48

Elicited judgements

quantiles	A	B	C	D	E
0.25	0.2077	0.0490	0.1490	0.1867	0.0530
0.50	0.2767	0.0517	0.1743	0.3200	0.1487
0.75	0.3570	0.0547	0.2017	0.4700	0.2890

Dirichlet density function and parameters

Define the vector of unknown population proportions as

$$\theta := (\theta_1, \dots, \theta_k),$$

with $= 5$. We write

$$\theta \sim Dirichlet(a_1, \dots, a_k),$$

with

$$f(\theta) = \frac{\Gamma(a_1 + \cdots + a_k)}{\Gamma(a_1) \dots \Gamma(a_k)} \prod_{i=1}^{k} \theta_i^{a_i - 1}.$$

The fitted parameter values $a_1, \dots, a_k$ are as follows:

```
##                 A                B                C                D                E
## 3.0071955 0.5448237 1.8549925 3.5730902 2.0566034
```

Comparing the elicited marginals with the marginals from the Dirichlet fit

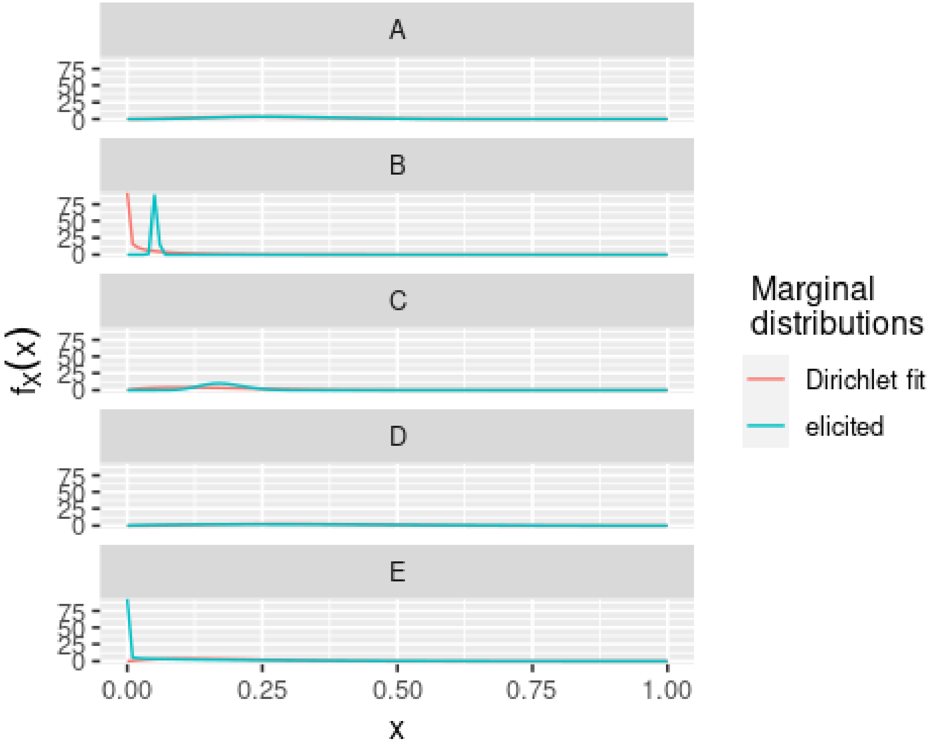


```
##
## Directly elicted beta marginal distributions:
##
##              A          B       C      D      E
## shape1   4.760   1.43e+02  16.700  1.740  0.800
## shape2  11.900   2.61e+03  77.800  3.380  3.290
## mean     0.286   5.19e-02   0.177  0.340  0.196
## sd       0.108   4.23e-03   0.039  0.192  0.176
## sum     16.600   2.75e+03  94.500  5.120  4.090
##
## Sum of elicited marginal means: 1.051
##
## Beta marginal distributions from Dirichlet fit:
##
##              A        B       C      D      E
## shape1   3.010   0.5450   1.850  3.570  2.060
## shape2   8.030  10.5000   9.180  7.460  8.980
## mean     0.272   0.0494   0.168  0.324  0.186
## sd       0.128   0.0624   0.108  0.135  0.112
## sum    11.000 11.0000 11.000 11.000 11.000
```

Carcharhinus limbatus

15 December 2021, 10:56

Elicited judgements

quantiles	A	B	C	D	E
0.25	0.0386	0.2220	0.1637	0.0898	1e-04
0.50	0.0696	0.5673	0.1977	0.1499	2e-04
0.75	0.1147	0.8197	0.2337	0.2280	3e-04

Dirichlet density function and parameters

Define the vector of unknown population proportions as

$$\theta := (\theta_1, \dots, \theta_k),$$

with $= 5$. We write

$$\theta \sim Dirichlet(a_1, \dots, a_k),$$

with

$$f(\theta) = \frac{\Gamma(a_1 + \cdots + a_k)}{\Gamma(a_1) \dots \Gamma(a_k)} \prod_{i=1}^{k} \theta_i^{a_i - 1}.$$

The fitted parameter values $a_1, \dots, a_k$ are as follows:

```
##           A           B           C           D           E
## 0.510714463 3.241670327 1.230509770 1.030591660 0.001412081
```

Comparing the elicited marginals with the marginals from the Dirichlet fit

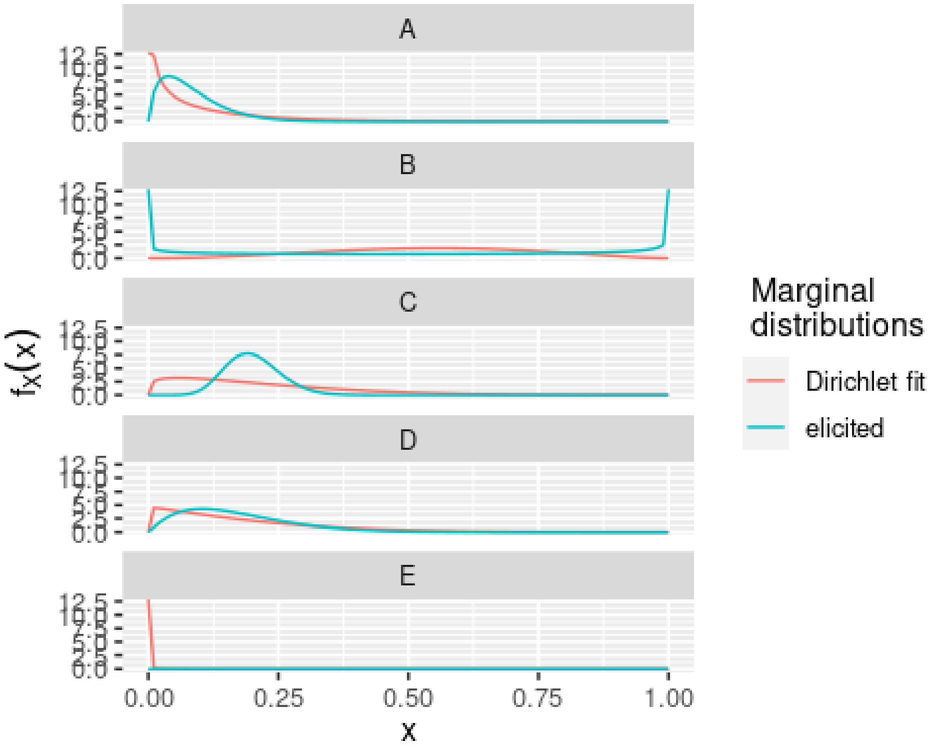

```
##
##         Directly         elicted         beta         marginal         distributions:
##
##                                A          B             C            D          E
##       shape1         1.7000      0.741      11.8000          2.070     1.80e+00
##         shape2       18.7000      0.660       47.1000      10.200     7.82e+03
##     mean                0.0833      0.529          0.2010         0.168     2.30e-04
##    sd                      0.0597     0.322          0.0517         0.103     1.72e-04
##     sum                   20.4000      1.400       58.9000       12.300     7.82e+03
##
##         Sum        of         elicited         marginal         means:         0.981
##
##       Beta       marginal       distributions       from       Dirichlet       fit:
##
##                                 A           B           C           D               E
##         shape1        0.5110          3.240       1.230       1.030        0.001410
##         shape2        5.5000          2.770       4.780       4.980        6.010000
##       mean                   0.0849       0.539       0.205       0.171        0.000235
##     sd                             0.1050       0.188       0.152       0.142        0.005780
## sum    6.0100 6.010 6.010 6.010 6.010000
```

Carcharias taurus

15 December 2021, 11:02

Elicited judgements

quantiles	A	B	C	D	E
0.25	0.1650	0.0652	0.1790	0.1727	0.0268
0.50	0.2340	0.1680	0.2137	0.2959	0.0457
0.75	0.3147	0.3257	0.2493	0.4230	0.0707

Dirichlet density function and parameters

Define the vector of unknown population proportions as

$$\theta := (\theta_1, \ldots, \theta_k),$$

with $= 5$. We write

$$\theta \sim Dirichlet(a_1, \ldots, a_k),$$

with

$$f(\theta) = \frac{\Gamma(a_1 + \cdots + a_k)}{\Gamma(a_1) \ldots \Gamma(a_k)} \prod_{i=1}^{k} \theta_i^{a_i - 1}.$$

The fitted parameter values $a_1, \ldots, a_k$ are as follows:

```
##                 A                B                C                D                E
## 2.4562634 2.1757785 2.1606328 3.1186461 0.5268918
```

Comparing the elicited marginals with the marginals from the Dirichlet fit

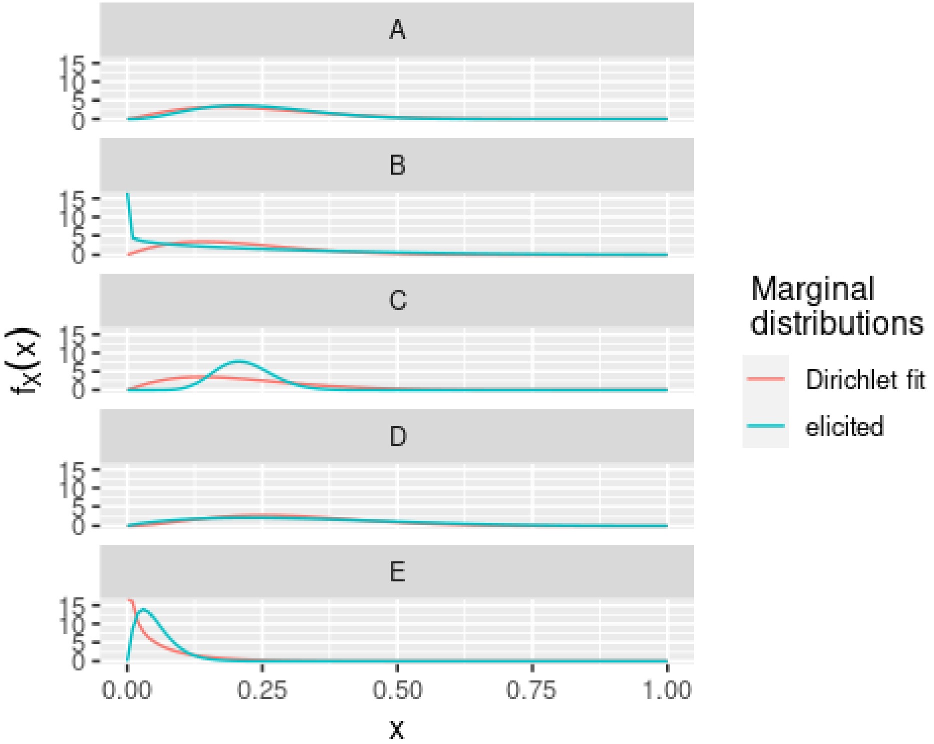


```
##
##      Directly      elicted      beta      marginal      distributions:
##
##                           A          B          C          D          E
##      shape1           3.630      0.840    13.4000      1.910     2.1400
##      shape2          11.100      3.020    48.5000      4.210    38.4000
##    mean               0.246      0.218     0.2160      0.312     0.0527
##    sd                 0.108      0.187     0.0519      0.174     0.0347
##     sum             14.800      3.860    61.8000      6.120    40.5000
##
##      Sum      of      elicited      marginal      means:      1.045
##
##      Beta      marginal      distributions      from      Dirichlet      fit:
##
##                           A          B          C          D          E
##    shape1             2.460      2.180      2.160      3.120     0.5270
##    shape2             7.980      8.260      8.280      7.320     9.9100
##    mean               0.235      0.208      0.207      0.299     0.0505
##    sd                 0.125      0.120      0.120      0.135     0.0647
## sum    10.400 10.400 10.400 10.400 10.4000
```

Sphyrna tudes

15 December 2021, 11:09

Elicited judgements

quantiles	A	B	C	D	E
0.25	0.0033	0.3067	0.0751	0.0128	0.0005
0.50	0.0109	0.8073	0.0801	0.0151	0.0250
0.75	0.0264	0.9223	0.0853	0.0176	0.1893

Dirichlet density function and parameters

Define the vector of unknown population proportions as

$$\theta := (\theta_1, \dots, \theta_k),$$

with $= 5$. We write

$$\theta \sim Dirichlet(a_1, \dots, a_k),$$

with

$$f(\theta) = \frac{\Gamma(a_1 + \dots + a_k)}{\Gamma(a_1) \dots \Gamma(a_k)} \prod_{i=1}^{k} \theta_i^{a_i - 1}.$$

The fitted parameter values $a_1, \dots, a_k$ are as follows:

```
##          A          B          C          D          E
## 0.05238385 1.74219719 0.22041104 0.04215420 0.40603851
```

Comparing the elicited marginals with the marginals from the Dirichlet fit

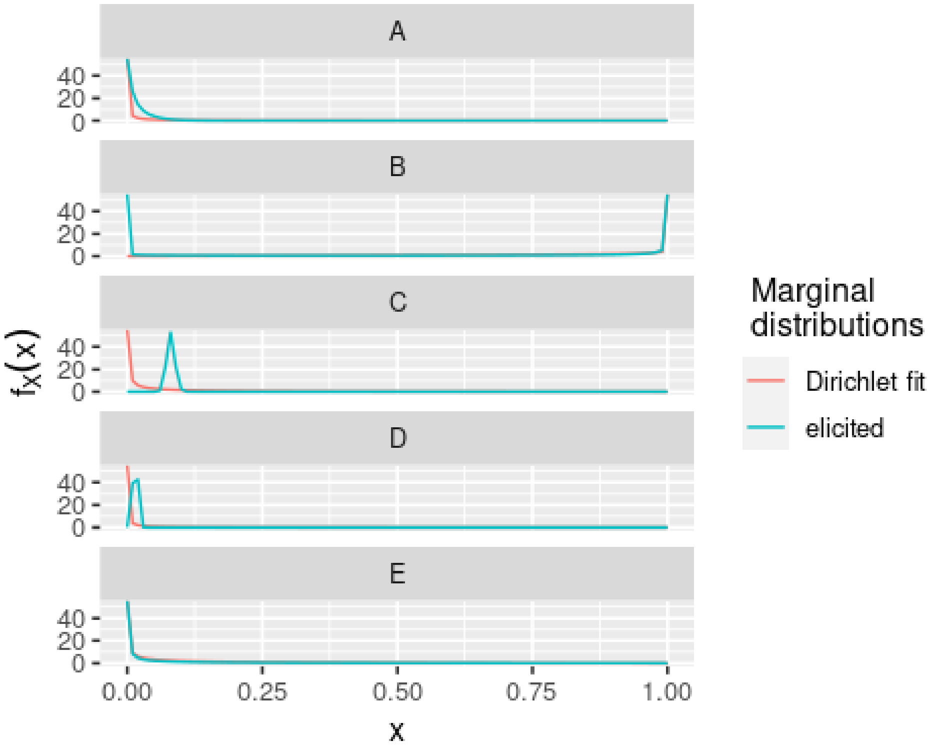

```
##
##        Directly        elicted        beta        marginal        distributions:
##
##                          A          B              C               D          E
##       shape1           0.6590     0.682      1.04e+02       1.80e+01      0.189
##        shape2       33.9000     0.392      1.19e+03      1.15e+03       1.090
##      mean                    0.0191      0.635      8.03e-02       1.54e-02     0.148
##     sd                          0.0230      0.334      7.57e-03       3.59e-03     0.235
##      sum                      34.5000      1.070      1.29e+03      1.17e+03      1.280
##
##        Sum        of        elicited        marginal        means:        0.898
##
##      Beta       marginal       distributions        from        Dirichlet        fit:
##
##                                A            B             C              D          E
##        shape1        0.0524          1.740        0.2200         0.0422        0.406
##        shape2        2.4100          0.721        2.2400         2.4200        2.060
##       mean                      0.0213        0.707        0.0895        0.0171       0.165
##      sd                              0.0775        0.244        0.1530        0.0697       0.199
## sum    2.4600 2.460 2.4600 2.4600 2.460
```

Galeus melastomus

15 December 2021, 11:17

Elicited judgements

quantiles	A	B	C	D	E
0.25	0.7510	1e-04	0.0230	1e-04	1e-04
0.50	0.9060	2e-04	0.0533	2e-04	2e-04
0.75	0.9337	3e-04	0.0987	3e-04	3e-04

Dirichlet density function and parameters

Define the vector of unknown population proportions as

$$\theta := (\theta_1, \dots, \theta_k),$$

with = 5 . We write

$$\theta \sim Dirichlet(a_1, \dots, a_k),$$

with

$$f(\theta) = \frac{\Gamma(a_1 + \cdots + a_k)}{\Gamma(a_1) \dots \Gamma(a_k)} \prod_{i=1}^{k} \theta_i^{a_i - 1}.$$

The fitted parameter values $a_1, \dots, a_k$ are as follows:

```
##                  A                 B                 C                 D                 E
## 7.008898167 0.001924015 0.588813150 0.001924015 0.001924015
```

Comparing the elicited marginals with the marginals from the Dirichlet fit

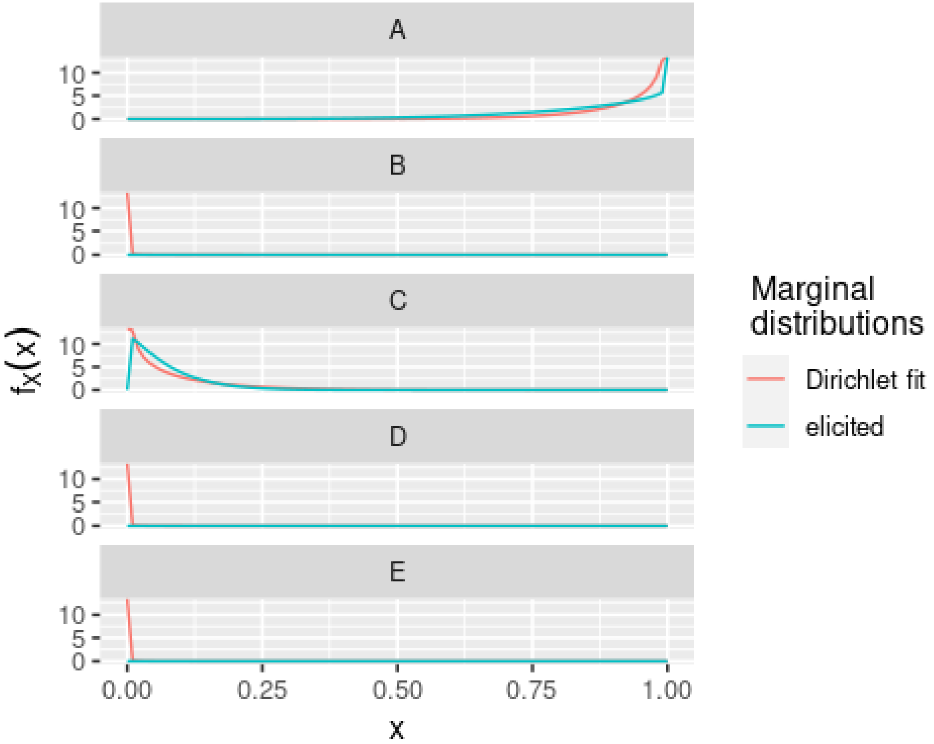


```
##
##      Directly      elicted      beta      marginal      distributions:
##
##                       A             B          C             D            E
##     shape1     4.730     1.80e+00         1.0800     1.80e+00     1.80e+00
##      shape2     0.905      7.82e+03     14.3000     7.82e+03     7.82e+03
##    mean               0.839     2.30e-04          0.0705     2.30e-04     2.30e-04
##    sd                     0.143     1.72e-04          0.0632     1.72e-04     1.72e-04
##    sum                    5.630      7.82e+03     15.4000     7.82e+03     7.82e+03
##
##      Sum       of       elicited        marginal        means:        0.911
##
##     Beta      marginal      distributions       from       Dirichlet       fit:
##
##                           A             B           C             D            E
##      shape1      7.0100      0.001920      0.5890      0.001920     0.001920
##      shape2      0.5950      7.600000      7.0100      7.600000     7.600000
##     mean               0.9220      0.000253      0.0774      0.000253     0.000253
##    sd                       0.0915      0.005420      0.0911      0.005420     0.005420
## sum     7.6000 7.600000 7.6000 7.600000 7.600000
```

Rhizoprionodon acutus

15 December 2021, 11:23

Elicited judgements

quantiles	A	B	C	D	E
0.25	0.4373	0.008	0.1013	0.1457	1e-04
0.50	0.5397	0.100	0.1327	0.2500	2e-04
0.75	0.6040	0.277	0.1660	0.3610	3e-04

Dirichlet density function and parameters

Define the vector of unknown population proportions as

$$\theta := (\theta_1, \ldots, \theta_k),$$

with $= 5$. We write

$$\theta \sim Dirichlet(a_1, \ldots, a_k),$$

with

$$f(\theta) = \frac{\Gamma(a_1 + \cdots + a_k)}{\Gamma(a_1) \ldots \Gamma(a_k)} \prod_{i=1}^{k} \theta_i^{a_i - 1}.$$

The fitted parameter values $a_1, \ldots, a_k$ are as follows:

```
##           A           B           C           D           E
## 3.769175408 1.379468277 0.977014664 1.909131629 0.001644918
```

Comparing the elicited marginals with the marginals from the Dirichlet fit

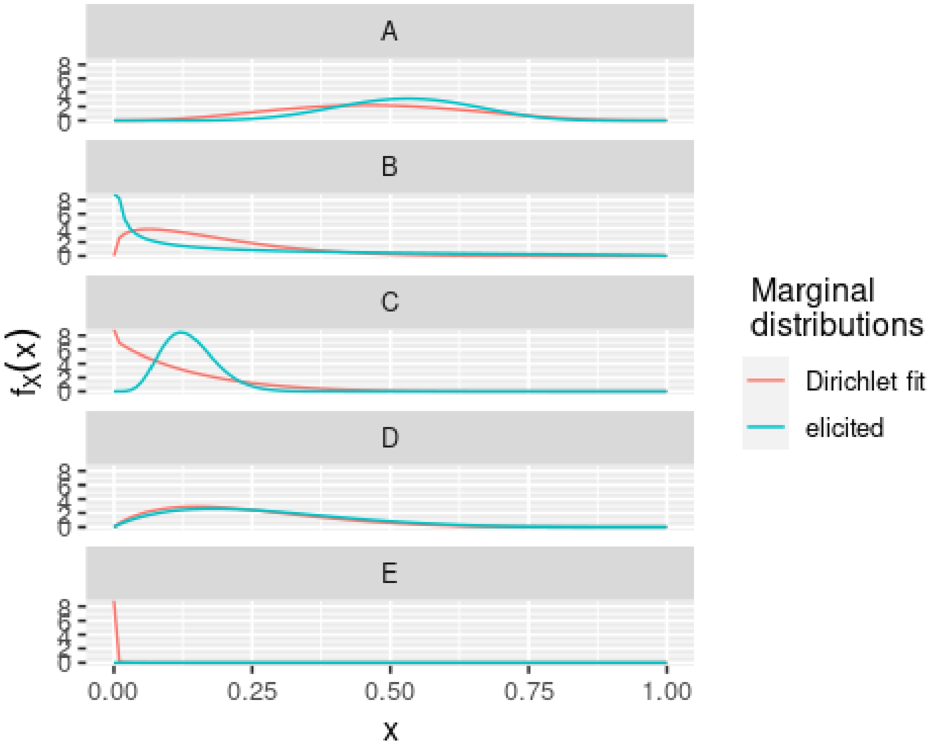

```
##
##         Directly         elicted         beta         marginal         distributions:
##
##                           A             B                 C            D                E
##       shape1             8.510       0.330              6.7900       1.970       1.80e+00
##        shape2              7.610        1.380        42.8000        5.390       7.82e+03
##      mean                     0.528       0.193              0.1370      0.267       2.30e-04
##     sd                           0.121       0.240              0.0483      0.153       1.72e-04
##      sum                        16.100       1.710        49.6000        7.360       7.82e+03
##
##         Sum         of         elicited         marginal         means:         1.126
##
##        Beta        marginal        distributions        from        Dirichlet        fit:
##
##                           A             B             C             D                E
##         shape1          3.770         1.380         0.977         1.910         0.001640
##         shape2          4.270         6.660         7.060         6.130         8.030000
##        mean                   0.469        0.172        0.122        0.238        0.000205
##       sd                           0.166        0.125        0.109        0.142        0.004760
## sum    8.040 8.040 8.040 8.040 8.040000
```

Carcharhinus brevipinna

15 December 2021, 11:29

Elicited judgements

quantiles	A	B	C	D	E
0.25	0.0386	0.1827	0.1550	0.0630	0.0289
0.50	0.0696	0.5557	0.1900	0.1042	0.0567
0.75	0.1147	0.6663	0.2277	0.1573	0.1047

Dirichlet density function and parameters

Define the vector of unknown population proportions as

$$\theta := (\theta_1, \ldots, \theta_k),$$

with $= 5$. We write

$$\theta \sim Dirichlet(a_1, \ldots, a_k),$$

with

$$f(\theta) = \frac{\Gamma(a_1 + \cdots + a_k)}{\Gamma(a_1) \ldots \Gamma(a_k)} \prod_{i=1}^{k} \theta_i^{a_i - 1}.$$

The fitted parameter values $a_1, \ldots, a_k$ are as follows:

```
##                  A                 B                 C                 D                 E
## 0.6388749 3.6546940 1.4844147 0.8993316 0.5662762
```

Comparing the elicited marginals with the marginals from the Dirichlet fit

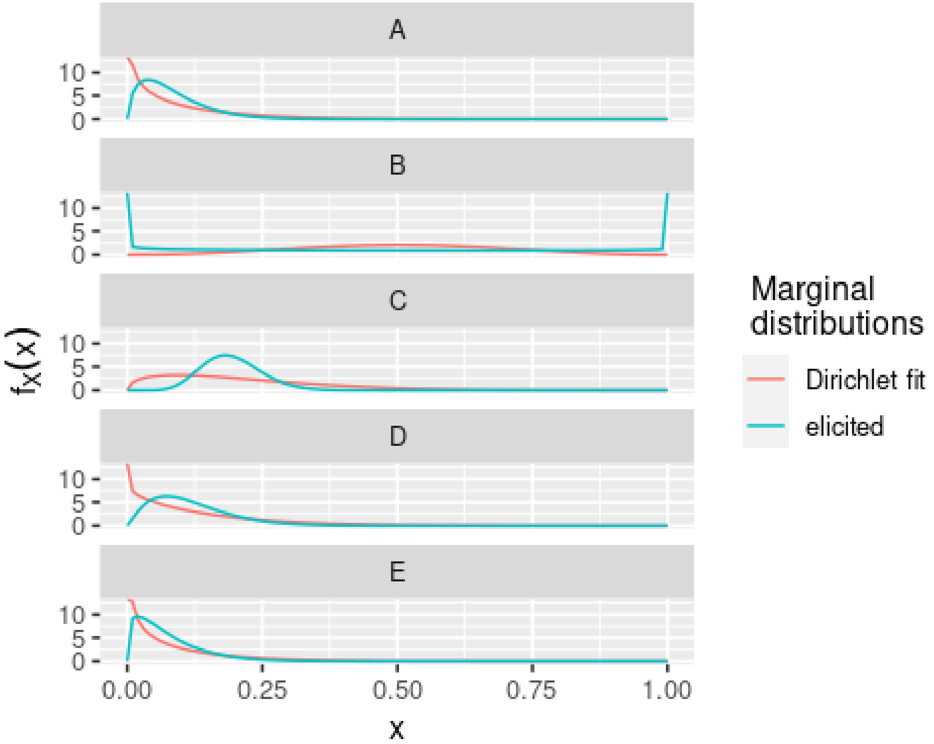


```
##
##        Directly        elicted        beta        marginal        distributions:
##
##                          A        B           C           D           E
##      shape1         1.7000    0.824    10.3000      2.2400      1.2900
##        shape2       18.7000    0.905    42.8000     16.8000     16.2000
##     mean             0.0833    0.477     0.1940      0.1170      0.0739
##     sd               0.0597    0.302     0.0537      0.0718      0.0609
##      sum            20.4000    1.730    53.1000     19.1000     17.5000
##
##        Sum        of        elicited        marginal        means:        0.945
##
##       Beta       marginal       distributions       from       Dirichlet       fit:
##
##                          A          B          C          D          E
##        shape1       0.6390      3.650      1.480      0.899     0.5660
##        shape2       6.6000      3.590      5.760      6.340     6.6800
##       mean          0.0882      0.505      0.205      0.124     0.0782
##      sd             0.0988      0.174      0.141      0.115     0.0935
## sum     7.2400 7.240 7.240 7.240 7.2400
```

Scyliorhinus canicula

15 December 2021, 11:34

Elicited judgements

quantiles	A	B	C	D	E
0.25	0.5247	1e-04	0.0553	0.2417	1e-04
0.50	0.6777	2e-04	0.0887	0.3480	2e-04
0.75	0.8080	3e-04	0.1497	0.4327	3e-04

Dirichlet density function and parameters

Define the vector of unknown population proportions as

$$\theta := (\theta_1, \dots, \theta_k),$$

with = 5 . We write

$$\theta \sim Dirichlet(a_1, \dots, a_k),$$

with

$$f(\theta) = \frac{\Gamma(a_1 + \dots + a_k)}{\Gamma(a_1) \dots \Gamma(a_k)} \prod_{i=1}^{k} \theta_i^{a_i - 1}.$$

The fitted parameter values $a_1, \dots, a_k$ are as follows:

```
##           A           B           C           D           E
## 5.000271949 0.001755421 0.817433024 2.655991336 0.001755421
```

Comparing the elicited marginals with the marginals from the Dirichlet fit

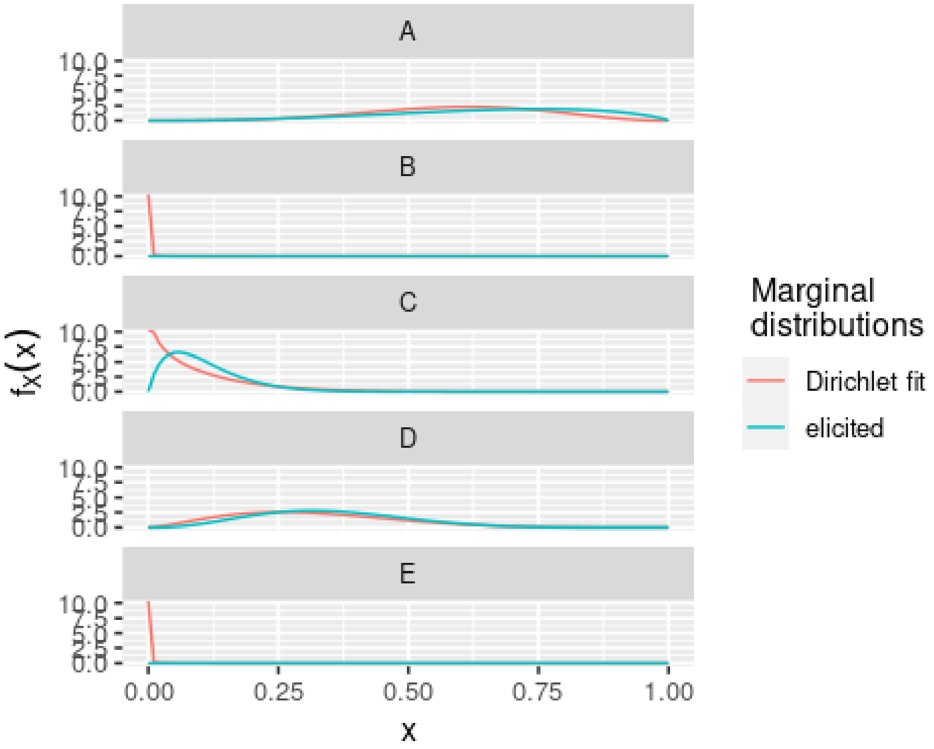

```
##
##      Directly      elicted      beta      marginal      distributions:
##
##                  A          B          C          D          E
##      shape1   3.390   1.80e+00   1.8800   3.870   1.80e+00
##      shape2   1.780   7.82e+03   15.7000   7.240   7.82e+03
##      mean     0.656   2.30e-04   0.1070   0.349   2.30e-04
##      sd       0.191   1.72e-04   0.0718   0.137   1.72e-04
##      sum      5.170   7.82e+03   17.6000   11.100   7.82e+03
##
##      Sum      of      elicited      marginal      means:      1.113
##
##      Beta      marginal      distributions      from      Dirichlet      fit:
##
##                  A          B          C          D          E
##      shape1   5.00   0.001760   0.8170   2.660   0.001760
##      shape2   3.48   8.480000   7.6600   5.820   8.480000
##      mean     0.59   0.000207   0.0964   0.313   0.000207
##      sd       0.16   0.004670   0.0959   0.151   0.004670
## sum    8.48 8.480000 8.4800 8.480 8.480000
```

Centroscymnus coelolepis

15 December 2021, 11:39

Elicited judgements

quantiles	A	B	C	D	E
0.25	0.6733	1e-04	0.1127	1e-04	1e-04
0.50	0.8177	2e-04	0.1587	2e-04	2e-04
0.75	0.9183	3e-04	0.2363	3e-04	3e-04

Dirichlet density function and parameters

Define the vector of unknown population proportions as

$$\theta := (\theta_1, \ldots, \theta_k),$$

with $= 5$. We write

$$\theta \sim Dirichlet(a_1, \ldots, a_k),$$

with

$$f(\theta) = \frac{\Gamma(a_1 + \cdots + a_k)}{\Gamma(a_1) \ldots \Gamma(a_k)} \prod_{i=1}^{k} \theta_i^{a_i - 1}.$$

The fitted parameter values $a_1, \ldots, a_k$ are as follows:

```
##           A           B           C           D           E
## 6.381624564 0.001892519 1.455205829 0.001892519 0.001892519
```

Comparing the elicited marginals with the marginals from the Dirichlet fit

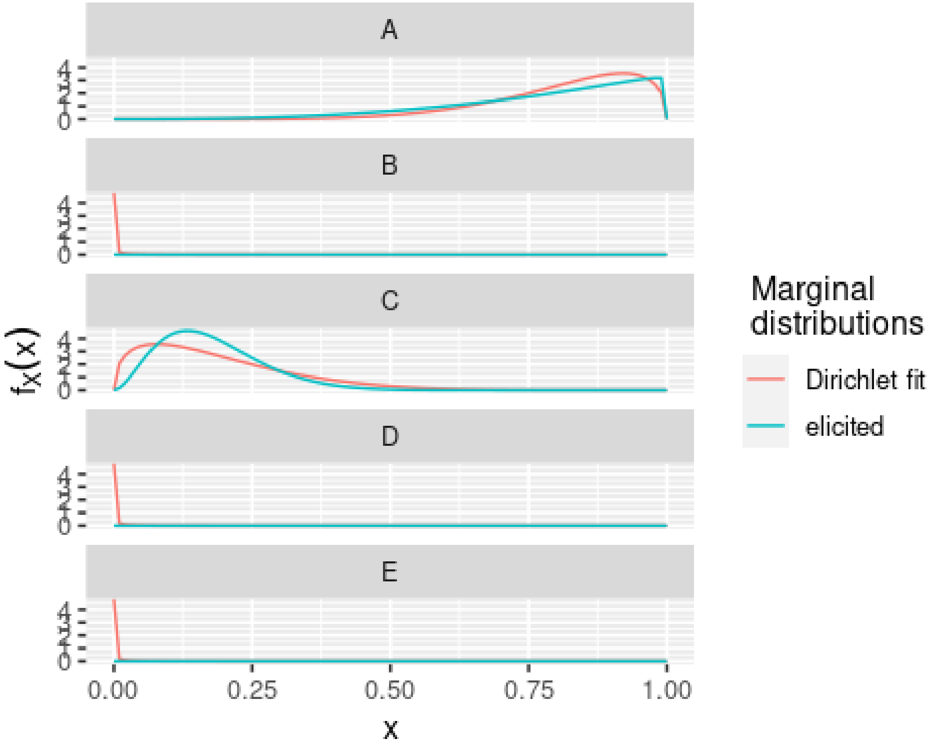


```
##
##      Directly      elicted      beta      marginal      distributions:
##
##                     A              B            C             D             E
##      shape1     3.620     1.80e+00        2.9600     1.80e+00      1.80e+00
##       shape2     1.040      7.82e+03     13.7000     7.82e+03      7.82e+03
##     mean              0.777     2.30e-04        0.1770     2.30e-04      2.30e-04
##    sd                   0.175     1.72e-04         0.0908     1.72e-04      1.72e-04
##     sum                 4.660      7.82e+03      16.7000     7.82e+03      7.82e+03
##
##      Sum      of      elicited      marginal      means:      0.955
##
##     Beta     marginal     distributions      from     Dirichlet     fit:
##
##                          A               B        C              D              E
##       shape1       6.380       0.001890     1.460      0.001890      0.001890
##       shape2       1.460       7.840000     6.390      7.840000      7.840000
##     mean                0.814     0.000241      0.186     0.000241      0.000241
##     sd                       0.131     0.005220      0.131     0.005220      0.005220
## sum    7.840 7.840000 7.840 7.840000 7.840000
```

Squalus blainvillei

15 December 2021, 11:58

Elicited judgements

quantiles	A	B	C	D	E
0.25	0.1990	0.0652	0.1490	0.1570	0.0530
0.50	0.2697	0.1680	0.1743	0.2690	0.1487
0.75	0.3513	0.3257	0.2017	0.3997	0.2890

Dirichlet density function and parameters

Define the vector of unknown population proportions as

$$\theta := (\theta_1, \dots, \theta_k),$$

with = 5 . We write

$$\theta \sim Dirichlet(a_1, \dots, a_k),$$

with

$$f(\theta) = \frac{\Gamma(a_1 + \cdots + a_k)}{\Gamma(a_1) \dots \Gamma(a_k)} \prod_{i=1}^{k} \theta_i^{a_i - 1}.$$

The fitted parameter values $a_1, \dots, a_k$ are as follows:

```
##                 A                 B                 C                 D                 E
## 2.014994 1.568493 1.272005 2.094807 1.410253
```

Comparing the elicited marginals with the marginals from the Dirichlet fit

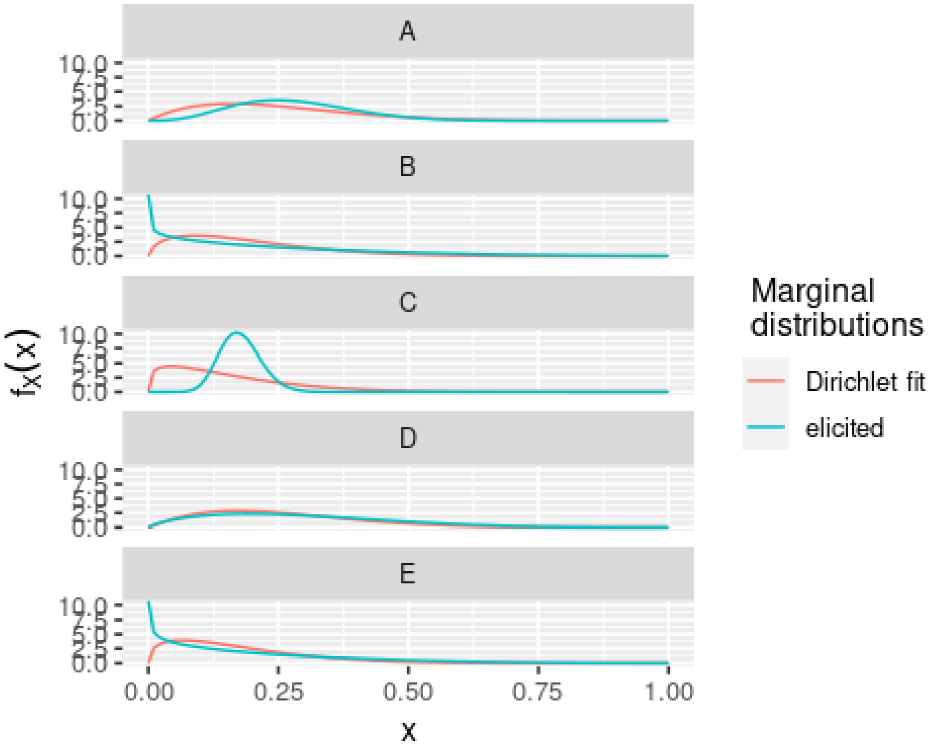

```
##
##         Directly         elicted         beta         marginal         distributions:
##
##                               A            B             C            D            E
##        shape1                4.40        0.840        16.700        1.810        0.800
##         shape2        11.30        3.020        77.800        4.420        3.290
##      mean                         0.28       0.218               0.177      0.291      0.196
##     sd                              0.11      0.187               0.039      0.169      0.176
##      sum                          15.70       3.860       94.500       6.230       4.090
##
##         Sum         of         elicited         marginal         means:         1.161
##
##        Beta        marginal        distributions        from        Dirichlet        fit:
##
##                                A            B            C            D            E
##          shape1          2.010          1.570          1.270          2.090          1.410
##          shape2          6.350          6.790          7.090          6.270          6.950
##       mean                        0.241        0.188        0.152        0.251        0.169
##      sd                              0.140        0.128        0.117        0.142        0.122
## sum    8.360 8.360 8.360 8.360 8.360
```

Alopias vulpinus

15 December 2021, 12:05

Elicited judgements

quantiles	A	B	C	D	E
0.25	0.0033	0.3240	0.0319	0.0159	0.0199
0.50	0.0109	0.7487	0.0360	0.0180	0.1005
0.75	0.0264	0.9103	0.0403	0.0202	0.2870

Dirichlet density function and parameters

Define the vector of unknown population proportions as

$$\theta := (\theta_1, \dots, \theta_k),$$

with $= 5$. We write

$$\theta \sim Dirichlet(a_1, \dots, a_k),$$

with

$$f(\theta) = \frac{\Gamma(a_1 + \cdots + a_k)}{\Gamma(a_1) \dots \Gamma(a_k)} \prod_{i=1}^{k} \theta_i^{a_i - 1}.$$

The fitted parameter values $a_1, \dots, a_k$ are as follows:

```
##          A          B          C          D          E
## 0.05495308 1.80136988 0.10454082 0.05229625 0.53366983
```

Comparing the elicited marginals with the marginals from the Dirichlet fit

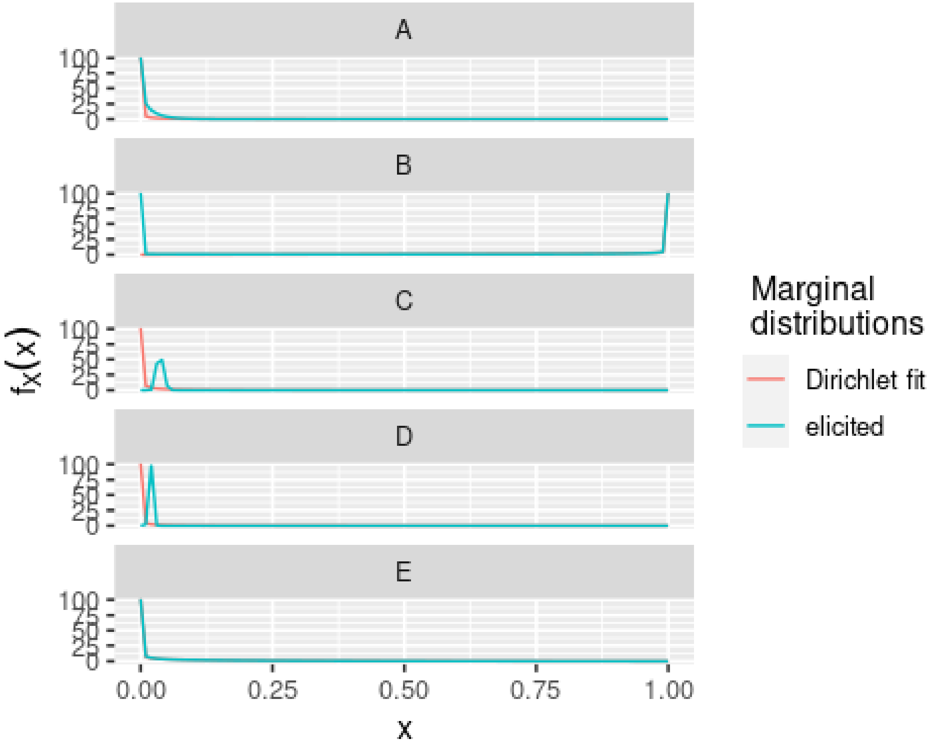


```
##
## Directly elicted beta marginal distributions:
##
##                A      B        C        D     E
## shape1    0.6590  0.790 3.24e+01 3.15e+01 0.442
## shape2   33.9000  0.473 8.60e+02 1.70e+03 1.940
## mean      0.0191  0.626 3.63e-02 1.82e-02 0.185
## sd        0.0230  0.322 6.26e-03 3.21e-03 0.211
## sum      34.5000  1.260 8.93e+02 1.73e+03 2.380
##
## Sum of elicited marginal means: 0.885
##
## Beta marginal distributions from Dirichlet fit:
##
##               A     B     C      D     E
## shape1   0.0550 1.800 0.105 0.0523 0.534
## shape2   2.4900 0.745 2.440 2.4900 2.010
## mean     0.0216 0.707 0.041 0.0205 0.210
## sd       0.0772 0.242 0.105 0.0753 0.216
## sum    2.5500 2.550 2.550 2.5500 2.550
```

Squalus acanthias

15 December 2021, 12:28

Elicited judgements

quantiles	A	B	C	D	E
0.25	0.1990	0.0718	0.0910	0.1208	0.1020
0.50	0.2697	0.1813	0.1120	0.2324	0.2003
0.75	0.3513	0.3290	0.1383	0.3626	0.3437

Dirichlet density function and parameters

Define the vector of unknown population proportions as

$$\theta := (\theta_1, \dots, \theta_k),$$

with $= 5$. We write

$$\theta \sim Dirichlet(a_1, \dots, a_k),$$

with

$$f(\theta) = \frac{\Gamma(a_1 + \cdots + a_k)}{\Gamma(a_1) \dots \Gamma(a_k)} \prod_{i=1}^{k} \theta_i^{a_i - 1}.$$

The fitted parameter values $a_1, \dots, a_k$ are as follows:

```
##         A         B         C         D         E
## 1.9856537 1.5900957 0.8220773 1.8359049 1.6847566
```

Comparing the elicited marginals with the marginals from the Dirichlet fit

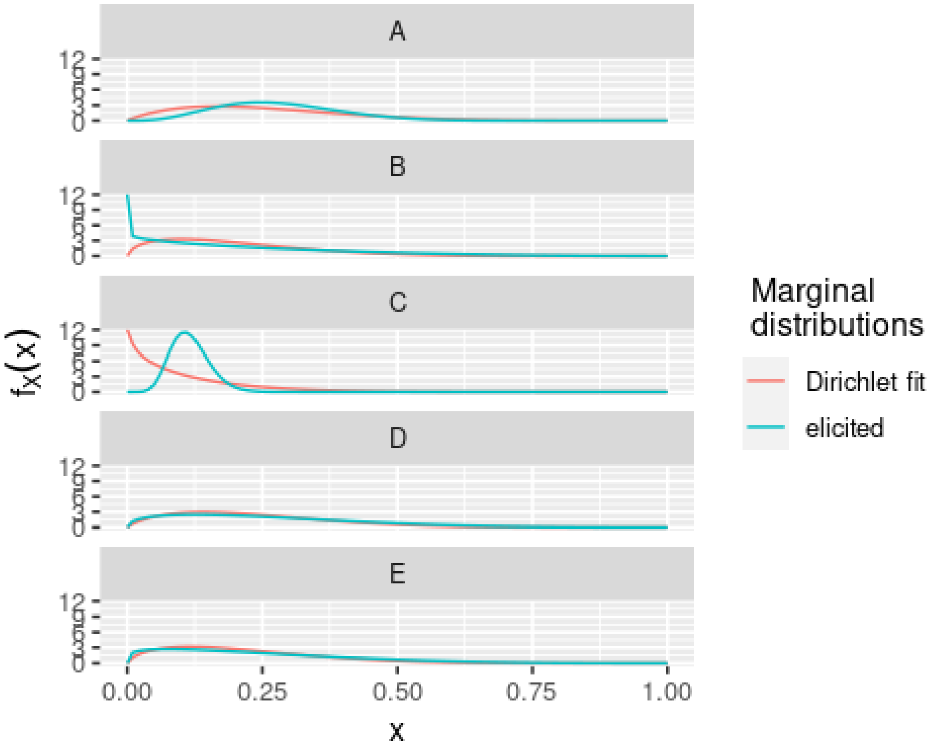

```
##
##          Directly          elicted          beta          marginal          distributions:
##
##                                 A             B                  C             D             E
##        shape1               4.40        0.913              9.4600        1.450         1.240
##          shape2          11.30          3.160         72.3000          4.150         4.000
##       mean                               0.28      0.224             0.1160       0.259        0.237
##      sd                                      0.11      0.185            0.0352      0.170        0.170
##       sum                                  15.70      4.080       81.7000       5.600        5.240
##
##          Sum          of          elicited          marginal          means:          1.116
##
##         Beta        marginal        distributions        from        Dirichlet        fit:
##
##                                    A             B             C             D              E
##           shape1           1.990         1.590         0.822         1.840          1.680
##           shape2           5.930         6.330         7.100         6.080          6.230
##        mean                            0.251       0.201       0.104       0.232        0.213
##       sd                                    0.145       0.134       0.102       0.141        0.137
## sum    7.920 7.920 7.920 7.920 7.920
```

Mustelus asterias

15 December 2021, 12:34

Elicited judgements

quantiles	A	B	C	D	E
0.25	0.2077	0.0490	0.1490	0.1867	0.0530
0.50	0.2767	0.0517	0.1743	0.3200	0.1487
0.75	0.3570	0.0547	0.2017	0.4700	0.2890

Dirichlet density function and parameters

Define the vector of unknown population proportions as

$$\theta := (\theta_1, \dots, \theta_k),$$

with $= 5$. We write

$$\theta \sim Dirichlet(a_1, \dots, a_k),$$

with

$$f(\theta) = \frac{\Gamma(a_1 + \dots + a_k)}{\Gamma(a_1) \dots \Gamma(a_k)} \prod_{i=1}^{k} \theta_i^{a_i - 1}.$$

The fitted parameter values $a_1, \dots, a_k$ are as follows:

```
##                   A                  B                  C                  D                  E
## 3.0071955 0.5448237 1.8549925 3.5730902 2.0566034
```

Comparing the elicited marginals with the marginals from the Dirichlet fit

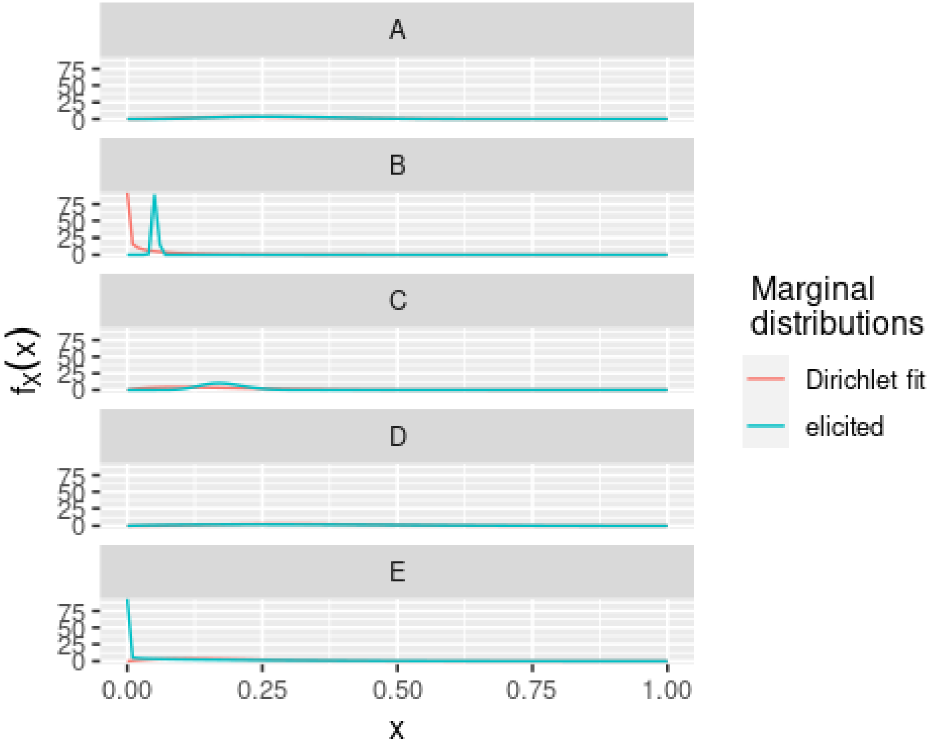


```
##
## Directly elicted beta marginal distributions:
##
## A B C D E
## shape1 4.760 1.43e+02 16.700 1.740 0.800
## shape2 11.900 2.61e+03 77.800 3.380 3.290
## mean 0.286 5.19e-02 0.177 0.340 0.196
## sd 0.108 4.23e-03 0.039 0.192 0.176
## sum 16.600 2.75e+03 94.500 5.120 4.090
##
## Sum of elicited marginal means: 1.051
##
## Beta marginal distributions from Dirichlet fit:
##
## A B C D E
## shape1 3.010 0.5450 1.850 3.570 2.060
## shape2 8.030 10.5000 9.180 7.460 8.980
## mean 0.272 0.0494 0.168 0.324 0.186
## sd 0.128 0.0624 0.108 0.135 0.112
## sum    11.000 11.0000 11.000 11.000 11.000
```

Heptranchias perlo

15 December 2021, 12:38

Elicited judgements

quantiles	A	B	C	D	E
0.25	0.5823	0.0453	0.1493	1e-04	1e-04
0.50	0.6460	0.1127	0.2160	2e-04	2e-04
0.75	0.6993	0.1990	0.2950	3e-04	3e-04

Dirichlet density function and parameters

Define the vector of unknown population proportions as

$$\theta := (\theta_1, \ldots, \theta_k),$$

with $= 5$. We write

$$\theta \sim Dirichlet(a_1, \ldots, a_k),$$

with

$$f(\theta) = \frac{\Gamma(a_1 + \cdots + a_k)}{\Gamma(a_1) \ldots \Gamma(a_k)} \prod_{i=1}^{k} \theta_i^{a_i - 1}.$$

The fitted parameter values $a_1, \ldots, a_k$ are as follows:

```
##            A           B           C           D           E
## 10.213131790  2.253356139  3.643199084  0.003672282  0.003672282
```

Comparing the elicited marginals with the marginals from the Dirichlet fit

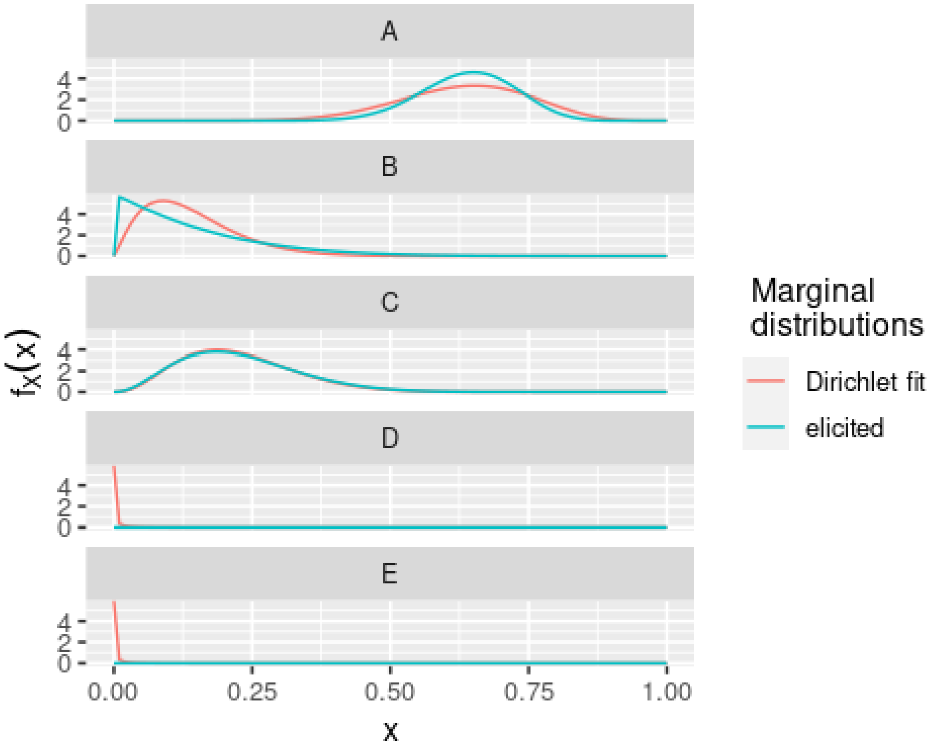

```
##
## Directly elicted beta marginal distributions:
##
##              A       B       C        D        E
## shape1 19.7000   1.020   3.370 1.80e+00 1.80e+00
## shape2 11.0000   6.190  11.400 7.82e+03 7.82e+03
## mean    0.6410   0.141   0.229 2.30e-04 2.30e-04
## sd      0.0852   0.122   0.106 1.72e-04 1.72e-04
## sum    30.7000   7.200  14.700 7.82e+03 7.82e+03
##
## Sum of elicited marginal means: 1.011
##
## Beta marginal distributions from Dirichlet fit:
##
##             A       B      C        D        E
## shape1 10.200  2.2500  3.640 3.67e-03 3.67e-03
## shape2  5.900 13.9000 12.500 1.61e+01 1.61e+01
## mean    0.634  0.1400  0.226 2.28e-04 2.28e-04
## sd      0.116  0.0838  0.101 3.65e-03 3.65e-03
## sum    16.100 16.1000 16.100 1.61e+01 1.61e+01
```

Galeorhinus galeus

15 December 2021, 12:43

Elicited judgements

quantiles	A	B	C	D	E
0.25	0.4253	0.0453	0.1797	0.0813	1e-04
0.50	0.5313	0.1127	0.3037	0.1203	2e-04
0.75	0.6327	0.1990	0.5147	0.1680	3e-04

Dirichlet density function and parameters

Define the vector of unknown population proportions as

$$\theta := (\theta_1, \ldots, \theta_k),$$

with $= 5$. We write

$$\theta \sim Dirichlet(a_1, \ldots, a_k),$$

with

$$f(\theta) = \frac{\Gamma(a_1 + \cdots + a_k)}{\Gamma(a_1) \ldots \Gamma(a_k)} \prod_{i=1}^{k} \theta_i^{a_i - 1}.$$

The fitted parameter values $a_1, \ldots, a_k$ are as follows:

```
##           A           B           C           D           E
## 3.547500403 0.948699664 2.340158642 0.871144497 0.001546091
```

Comparing the elicited marginals with the marginals from the Dirichlet fit

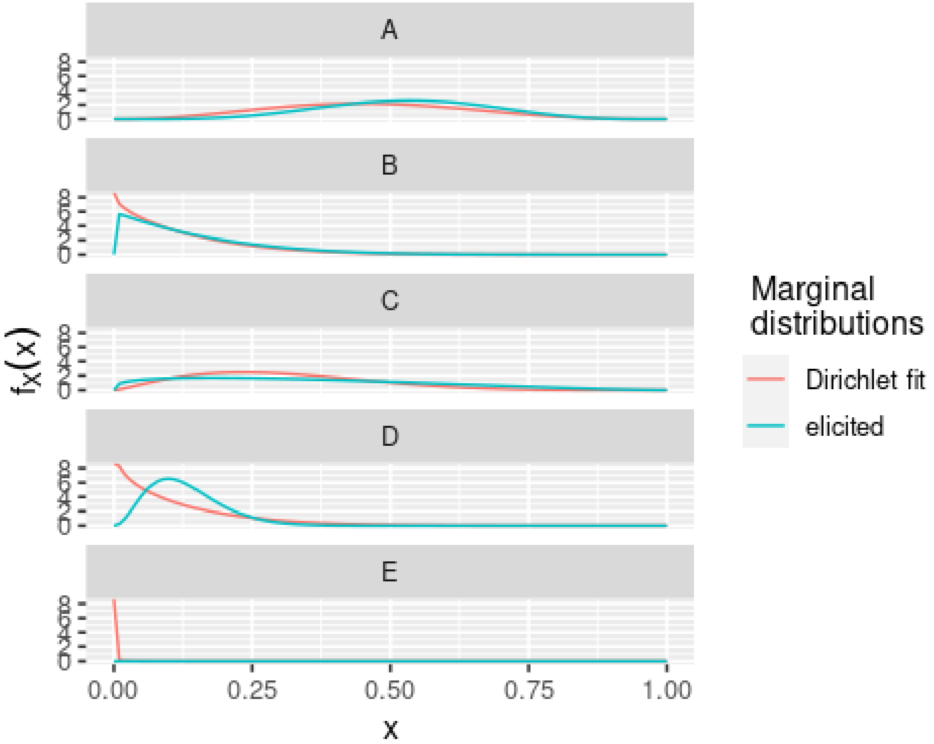


```
##
##      Directly       elicted       beta       marginal       distributions:
##
##                         A         B         C              D           E
##     shape1          5.710     1.020     1.310         3.3300    1.80e+00
##      shape2            5.090      6.190      2.450     22.3000    7.82e+03
##    mean                  0.529     0.141     0.349        0.1300    2.30e-04
##    sd                       0.145     0.122     0.218      0.0651    1.72e-04
##     sum                   10.800     7.200     3.760     25.7000    7.82e+03
##
##      Sum       of       elicited       marginal       means:       1.149
##
##      Beta      marginal      distributions      from      Dirichlet      fit:
##
##                          A          B          C          D           E
##       shape1       3.550      0.949      2.340      0.871      0.001550
##       shape2       4.160      6.760      5.370      6.840      7.710000
##     mean                 0.460      0.123      0.304      0.113      0.000201
##     sd                       0.169      0.111      0.156      0.107      0.004800
## sum    7.710 7.710 7.710 7.710 7.710000
```

Mustelus mustelus

15 December 2021, 12:50

Elicited judgements

quantiles	A	B	C	D	E
0.25	0.2077	0.0490	0.1490	0.1867	0.0530
0.50	0.2767	0.0517	0.1743	0.3200	0.1487
0.75	0.3570	0.0547	0.2017	0.4700	0.2890

Dirichlet density function and parameters

Define the vector of unknown population proportions as

$$\theta := (\theta_1, \dots, \theta_k),$$

with $= 5$. We write

$$\theta \sim Dirichlet(a_1, \dots, a_k),$$

with

$$f(\theta) = \frac{\Gamma(a_1 + \dots + a_k)}{\Gamma(a_1) \dots \Gamma(a_k)} \prod_{i=1}^{k} \theta_i^{a_i - 1}.$$

The fitted parameter values $a_1, \dots, a_k$ are as follows:

```
##         A         B         C         D         E
## 3.0071955 0.5448237 1.8549925 3.5730902 2.0566034
```

Comparing the elicited marginals with the marginals from the Dirichlet fit

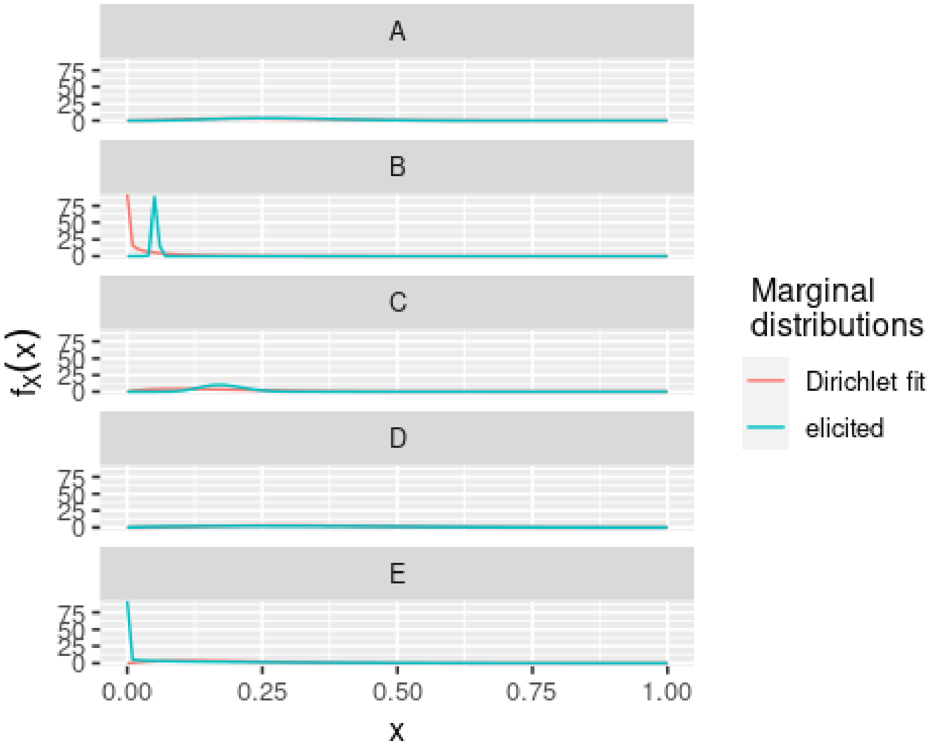

```
##
##       Directly        elicted        beta        marginal        distributions:
##
##                          A              B            C          D           E
##      shape1            4.760       1.43e+02      16.700      1.740       0.800
##       shape2         11.900        2.61e+03      77.800      3.380       3.290
##     mean                 0.286       5.19e-02        0.177      0.340       0.196
##    sd                      0.108      4.23e-03        0.039      0.192       0.176
##      sum                 16.600      2.75e+03      94.500      5.120       4.090
##
##       Sum       of        elicited        marginal        means:        1.051
##
##      Beta      marginal       distributions       from       Dirichlet       fit:
##
##                         A             B           C            D            E
##     shape1          3.010         0.5450        1.850        3.570        2.060
##      shape2          8.030      10.5000        9.180        7.460        8.980
##    mean               0.272         0.0494        0.168        0.324        0.186
##    sd                     0.128         0.0624        0.108        0.135        0.112
## sum    11.000 11.0000 11.000 11.000 11.000
```

Carcharhinus altimus

15 December 2021, 12:55

Elicited judgements

quantiles	A	B	C	D	E
0.25	0.0033	0.1883	0.1645	1e-04	1e-04
0.50	0.0109	0.6233	0.3421	2e-04	2e-04
0.75	0.0264	0.6653	0.3509	3e-04	3e-04

Dirichlet density function and parameters

Define the vector of unknown population proportions as

$$\theta := (\theta_1, \ldots, \theta_k),$$

with $= 5$. We write

$$\theta \sim Dirichlet(a_1, \ldots, a_k),$$

with

$$f(\theta) = \frac{\Gamma(a_1 + \cdots + a_k)}{\Gamma(a_1) \ldots \Gamma(a_k)} \prod_{i=1}^{k} \theta_i^{a_i - 1}.$$

The fitted parameter values $a_1, \ldots, a_k$ are as follows:

```
##                 A                B                C                D                E
## 0.0686607372 1.7747302468 1.0972301752 0.0008287596 0.0008287596
```

Comparing the elicited marginals with the marginals from the Dirichlet fit

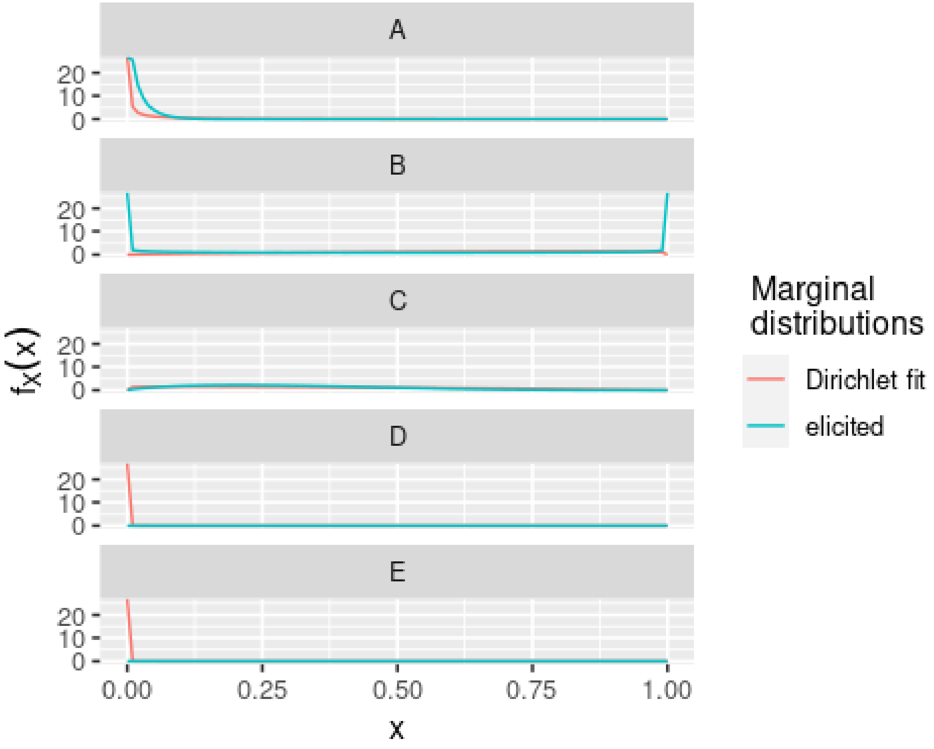


```
##
##    Directly elicted beta marginal distributions:
##
##                    A       B       C          D          E
##    shape1     0.6590   0.730   1.810   1.80e+00   1.80e+00
##    shape2    33.9000   0.750   4.130   7.82e+03   7.82e+03
##    mean       0.0191   0.493   0.305   2.30e-04   2.30e-04
##    sd         0.0230   0.317   0.175   1.72e-04   1.72e-04
##    sum       34.5000   1.480   5.940   7.82e+03   7.82e+03
##
##    Sum of elicited marginal means: 0.818
##
##    Beta marginal distributions from Dirichlet fit:
##
##                    A       B       C          D          E
##    shape1     0.0687   1.770   1.100   0.000829   0.000829
##    shape2     2.8700   1.170   1.850   2.940000   2.940000
##    mean       0.0233   0.603   0.373   0.000282   0.000282
##    sd         0.0760   0.246   0.244   0.008450   0.008450
## sum     2.9400 2.940 2.940 2.940000 2.940000
```

Isurus oxyrinchus

15 December 2021, 13:00

Elicited judgements

quantiles	A	B	C	D	E
0.25	0.0673	0.2680	0.0526	0.0365	0.0050
0.50	0.0949	0.6547	0.0733	0.0523	0.0250
0.75	0.1300	0.8600	0.0988	0.0728	0.1893

Dirichlet density function and parameters

Define the vector of unknown population proportions as

$$\theta := (\theta_1, \dots, \theta_k),$$

with $= 5$. We write

$$\theta \sim Dirichlet(a_1, \dots, a_k),$$

with

$$f(\theta) = \frac{\Gamma(a_1 + \cdots + a_k)}{\Gamma(a_1) \dots \Gamma(a_k)} \prod_{i=1}^{k} \theta_i^{a_i - 1}.$$

The fitted parameter values $a_1, \dots, a_k$ are as follows:

```
##         A         B         C         D         E
## 0.4615056 2.6019066 0.3543038 0.2571628 0.5628887
```

Comparing the elicited marginals with the marginals from the Dirichlet fit

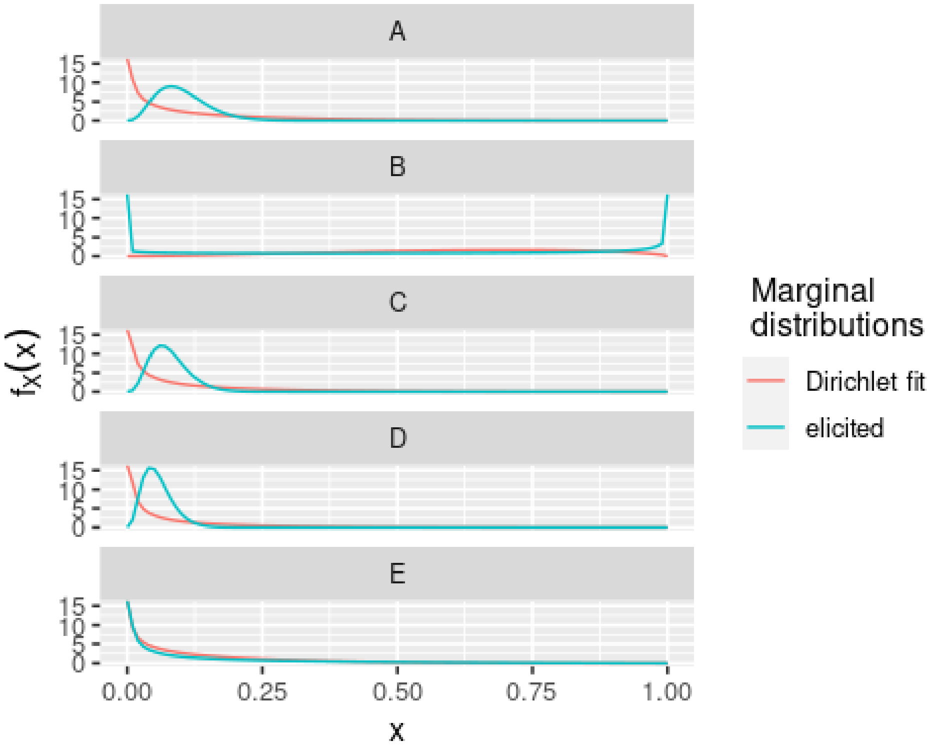

```
##
##          Directly          elicted          beta          marginal          distributions:
##
##                                     A           B              C               D          E
##       shape1           4.0800      0.787          4.5400          3.9000      0.280
##         shape2        36.0000        0.583       53.4000       64.8000        1.970
##      mean                    0.1020    0.574          0.0782          0.0568     0.124
##     sd                         0.0472    0.321          0.0350          0.0277    0.183
##       sum                      40.1000      1.370      58.0000      68.7000      2.250
##
##          Sum          of          elicited          marginal          means:          0.936
##
##        Beta        marginal        distributions         from        Dirichlet         fit:
##
##                                  A           B              C              D           E
##          shape1          0.462          2.600          0.3540          0.2570          0.563
##          shape2          3.780          1.640          3.8800          3.9800          3.670
##        mean                          0.109        0.614        0.0836       0.0607     0.133
##       sd                                 0.136       0.213        0.1210       0.1040     0.148
## sum    4.240 4.240 4.2400 4.2400 4.240
```

Odontaspis ferox

15 December 2021, 13:05

Elicited judgements

quantiles	A	B	C	D	E
0.25	0.3987	0.0718	0.1733	0.0442	1e-04
0.50	0.4810	0.1813	0.2397	0.0662	2e-04
0.75	0.5623	0.3290	0.3183	0.0953	3e-04

Dirichlet density function and parameters

Define the vector of unknown population proportions as

$$\theta := (\theta_1, \ldots, \theta_k),$$

with $= 5$. We write

$$\theta \sim Dirichlet(a_1, \ldots, a_k),$$

with

$$f(\theta) = \frac{\Gamma(a_1 + \cdots + a_k)}{\Gamma(a_1) \ldots \Gamma(a_k)} \prod_{i=1}^{k} \theta_i^{a_i - 1}.$$

The fitted parameter values $a_1, \ldots, a_k$ are as follows:

```
##                 A                B                C                D                E
## 5.427087354 2.526106635 2.828673781 0.823250496 0.002598261
```

Comparing the elicited marginals with the marginals from the Dirichlet fit

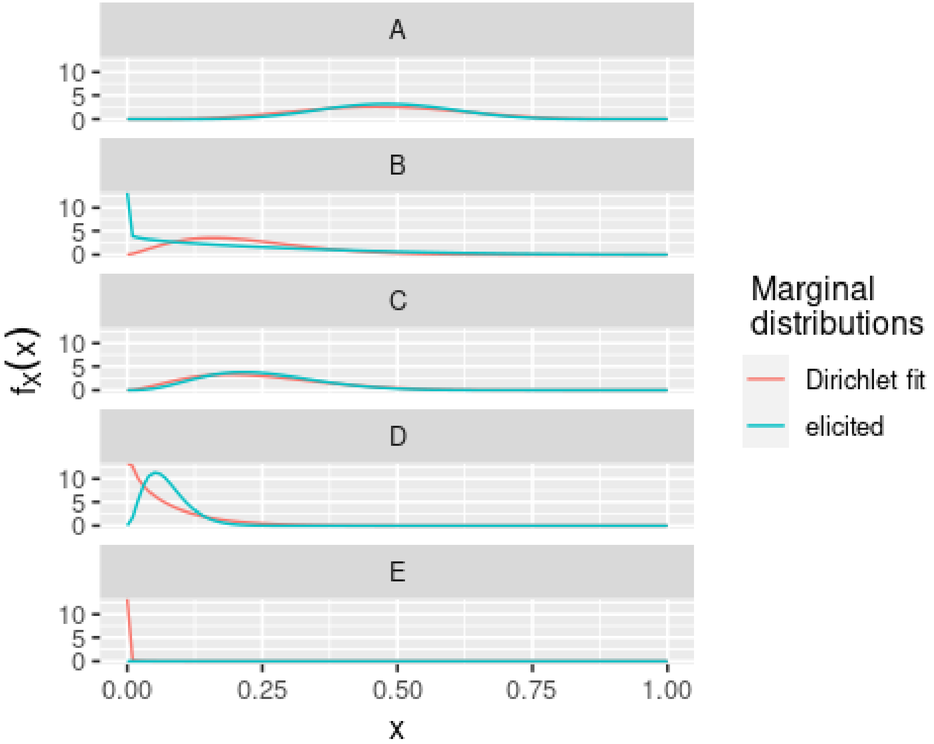


```
##
##      Directly      elicted      beta      marginal      distributions:
##
##                         A          B            C             D            E
##     shape1          8.290     0.913         4.020        3.1600     1.80e+00
##      shape2            8.940       3.160      12.000      40.1000     7.82e+03
##    mean                    0.481     0.224        0.251        0.0730     2.30e-04
##    sd                         0.117     0.185        0.105       0.0391     1.72e-04
##     sum                      17.200      4.080      16.000     43.3000     7.82e+03
##
##       Sum        of        elicited        marginal        means:        1.029
##
##      Beta        marginal        distributions        from        Dirichlet        fit:
##
##                        A            B            C            D            E
##    shape1          5.430          2.530        2.830        0.8230     2.60e-03
##     shape2            6.180          9.080         8.780     10.8000     1.16e+01
##    mean                  0.468          0.218       0.244        0.0709     2.24e-04
##    sd                       0.141          0.116        0.121        0.0723     4.21e-03
## sum    11.600 11.600 11.600 11.6000 1.16e+01
```

Somniosus rostratus

15 December 2021, 13:18

Elicited judgements

quantiles	A	B	C	D	E
0.25	0.4977	0.0717	0.0693	1e-04	1e-04
0.50	0.5667	0.3320	0.0953	2e-04	2e-04
0.75	0.6160	0.3333	0.1490	3e-04	3e-04

Dirichlet density function and parameters

Define the vector of unknown population proportions as

$$\theta := (\theta_1, \ldots, \theta_k),$$

with $= 5$. We write

$$\theta \sim Dirichlet(a_1, \ldots, a_k),$$

with

$$f(\theta) = \frac{\Gamma(a_1 + \cdots + a_k)}{\Gamma(a_1) \ldots \Gamma(a_k)} \prod_{i=1}^{k} \theta_i^{a_i - 1}.$$

The fitted parameter values $a_1, \ldots, a_k$ are as follows:

```
##           A           B           C           D           E
## 4.998359576 2.620327637 0.985364416 0.002055826 0.002055826
```

Comparing the elicited marginals with the marginals from the Dirichlet fit

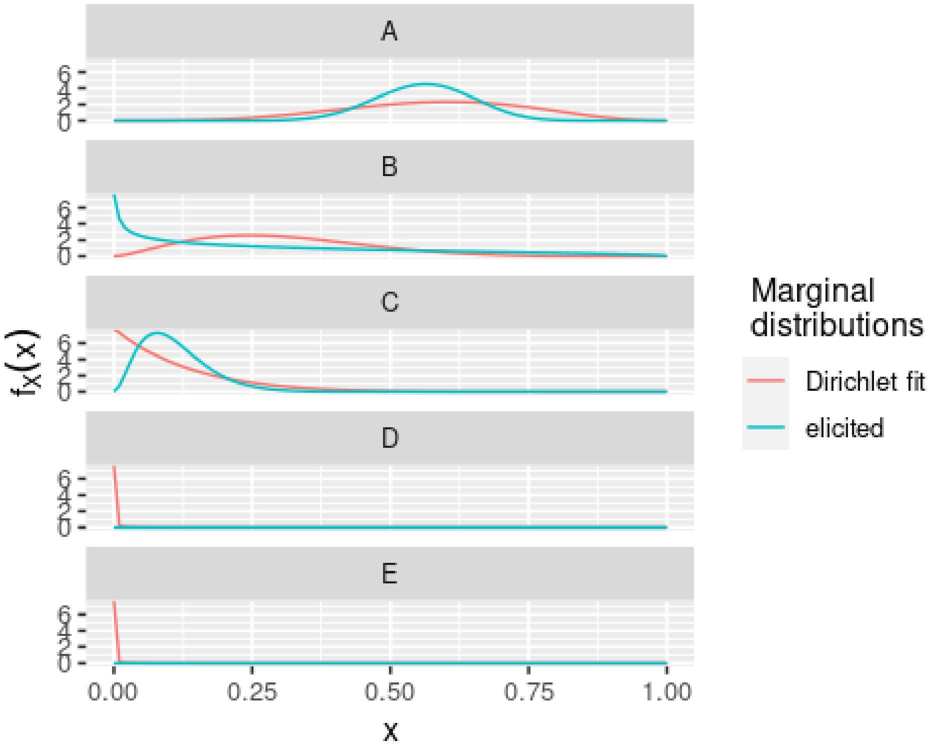

```
##
##          Directly          elicted          beta          marginal          distributions:
##
##                            A           B              C              D              E
##        shape1      17.9000       0.638             2.9000       1.80e+00       1.80e+00
##         shape2       14.0000        1.540       23.3000       7.82e+03       7.82e+03
##      mean                     0.5600      0.294          0.1100      2.30e-04      2.30e-04
##     sd                           0.0866      0.256          0.0601      1.72e-04      1.72e-04
##      sum                         31.9000      2.170      26.2000      7.82e+03      7.82e+03
##
##          Sum          of          elicited          marginal          means:          0.965
##
##        Beta        marginal        distributions        from        Dirichlet        fit:
##
##                          A            B            C              D              E
##          shape1        5.000         2.620        0.985       0.002060       0.002060
##          shape2        3.610         5.990        7.620       8.610000       8.610000
##        mean                    0.581       0.304       0.114      0.000239      0.000239
##       sd                            0.159       0.148       0.103      0.004990      0.004990
## sum    8.610 8.610 8.610 8.610000 8.610000
```

Prionace glauca

15 December 2021, 13:24

Elicited judgements

quantiles	A	B	C	D	E
0.25	0.0128	0.2863	0.0159	0.0128	0.0178
0.50	0.0151	0.8250	0.0180	0.0151	0.0895
0.75	0.0176	0.9197	0.0202	0.0176	0.2530

Dirichlet density function and parameters

Define the vector of unknown population proportions as

$$\theta := (\theta_1, \dots, \theta_k),$$

with $= 5$. We write

$$\theta \sim Dirichlet(a_1, \dots, a_k),$$

with

$$f(\theta) = \frac{\Gamma(a_1 + \cdots + a_k)}{\Gamma(a_1) \dots \Gamma(a_k)} \prod_{i=1}^{k} \theta_i^{a_i - 1}.$$

The fitted parameter values $a_1, \dots, a_k$ are as follows:

```
##                 A                 B                 C                 D                 E
## 0.03807129 1.56156814 0.04502282 0.03807129 0.41351926
```

Comparing the elicited marginals with the marginals from the Dirichlet fit

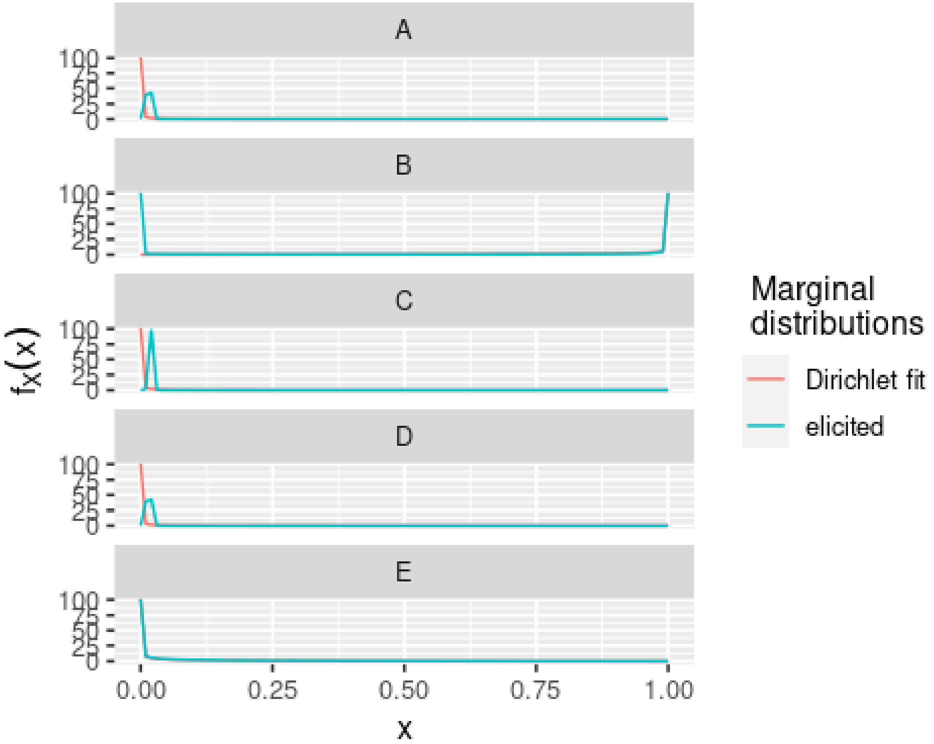


```
##
##         Directly        elicted        beta        marginal        distributions:
##
##                                A        B               C               D        E
##        shape1       1.80e+01      0.624      3.15e+01      1.80e+01      0.450
##        shape2       1.15e+03      0.367      1.70e+03      1.15e+03      2.250
##      mean                  1.54e-02     0.630      1.82e-02      1.54e-02     0.167
##     sd                         3.59e-03     0.342      3.21e-03      3.59e-03     0.194
##     sum                       1.17e+03     0.991      1.73e+03      1.17e+03     2.700
##
##         Sum        of        elicited        marginal        means:        0.846
##
##        Beta        marginal        distributions        from        Dirichlet        fit:
##
##                                A           B                C                D        E
##         shape1        0.0381        1.560        0.0450        0.0381        0.414
##         shape2        2.0600        0.535        2.0500        2.0600        1.680
##       mean                 0.0182       0.745       0.0215       0.0182       0.197
##      sd                         0.0759      0.248      0.0824      0.0759       0.226
## sum     2.1000 2.100 2.1000 2.1000 2.100
```

Alopias superciliosus

Fitted Dirichlet Distribution

Elicited judgements

quantiles	A	B	C	D	E
0.25	0.050	0.71	0.001	0.040	0.04
0.50	0.051	0.78	0.002	0.050	0.08
0.75	0.060	0.84	0.999	0.051	0.13

Dirichlet density function and parameters

Define the vector of unknown population proportions as

$$\theta := (\theta_1, \ldots, \theta_k),$$

with $k = 5$. We write

$$\theta \sim Dirichlet(a_1, \ldots, a_k),$$

with

$$f(\theta) = \frac{\Gamma(a_1 + \cdots + a_k)}{\Gamma(a_1) \ldots \Gamma(a_k)} \prod_{i=1}^{k} \theta_i^{a_i - 1}.$$

The fitted parameter values $a_1, \ldots, a_k$ are as follows:

```
##         A         B         C         D         E
## 0.1239559 1.7730185 1.0002144 0.1099376 0.2189328
```

Comparing the elicited marginals with the marginals from the Dirichlet fit

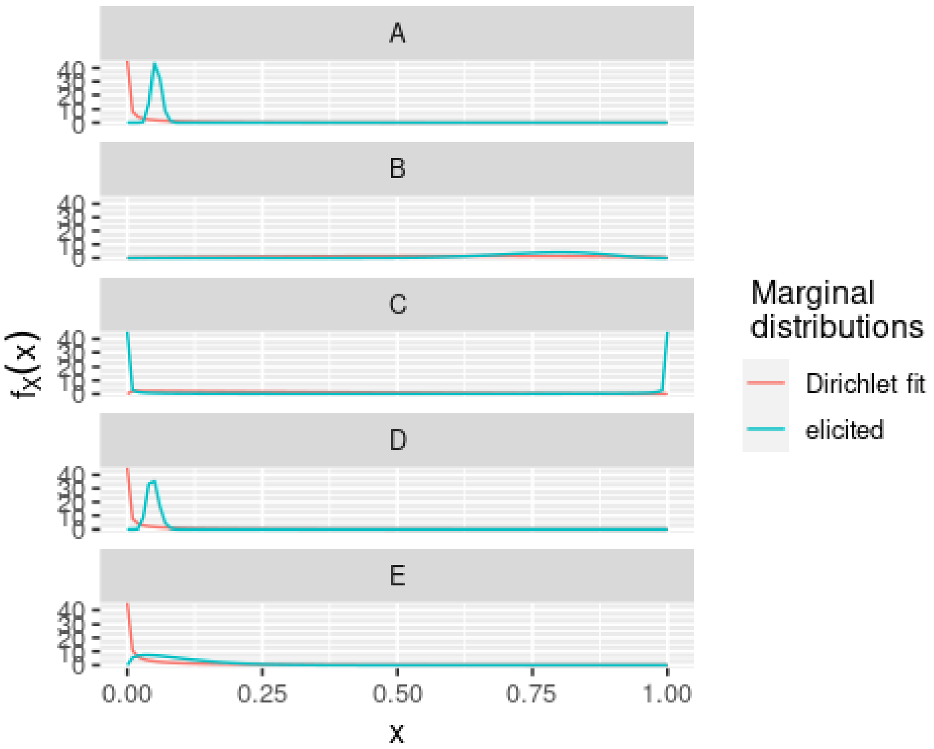


```
##
## Directly elicted beta marginal distributions:
##
##          A       B       C       D        E
## shape1   3.45e+01 14.4000 0.0645 19.8000  1.5000
## shape2   6.06e+02 4.3000  0.0840 395.0000 14.3000
## mean     5.38e-02 0.7700  0.4340 0.0477   0.0951
## sd       8.91e-03 0.0948  0.4630 0.0105   0.0717
## sum      6.40e+02 18.7000 0.1490 415.0000 15.8000
##
## Sum of elicited marginal means: 1.401
##
## Beta marginal distributions from Dirichlet fit:
##
##          A      B     C     D      E
## shape1   0.1240 1.770 1.000 0.1100 0.2190
## shape2   3.1000 1.450 2.230 3.1200 3.0100
## mean     0.0384 0.550 0.310 0.0341 0.0679
## sd       0.0935 0.242 0.225 0.0883 0.1220
## sum    3.2300 3.230 3.230 3.2300 3.2300
```

Carcharhinus falciformis

15 December 2021, 13:35

Elicited judgements

quantiles	A	B	C	D	E
0.25	0.0033	0.2603	0.1497	0.0258	1e-04
0.50	0.0109	0.7407	0.1910	0.0406	2e-04
0.75	0.0264	0.8707	0.2343	0.0613	3e-04

Dirichlet density function and parameters

Define the vector of unknown population proportions as

$$\theta := (\theta_1, \ldots, \theta_k),$$

with $= 5$. We write

$$\theta \sim Dirichlet(a_1, \ldots, a_k),$$

with

$$f(\theta) = \frac{\Gamma(a_1 + \cdots + a_k)}{\Gamma(a_1) \ldots \Gamma(a_k)} \prod_{i=1}^{k} \theta_i^{a_i - 1}.$$

The fitted parameter values $a_1, \ldots, a_k$ are as follows:

```
##           A           B           C           D           E
## 0.100168108 3.115304355 1.025442662 0.242129743 0.001209065
```

Comparing the elicited marginals with the marginals from the Dirichlet fit

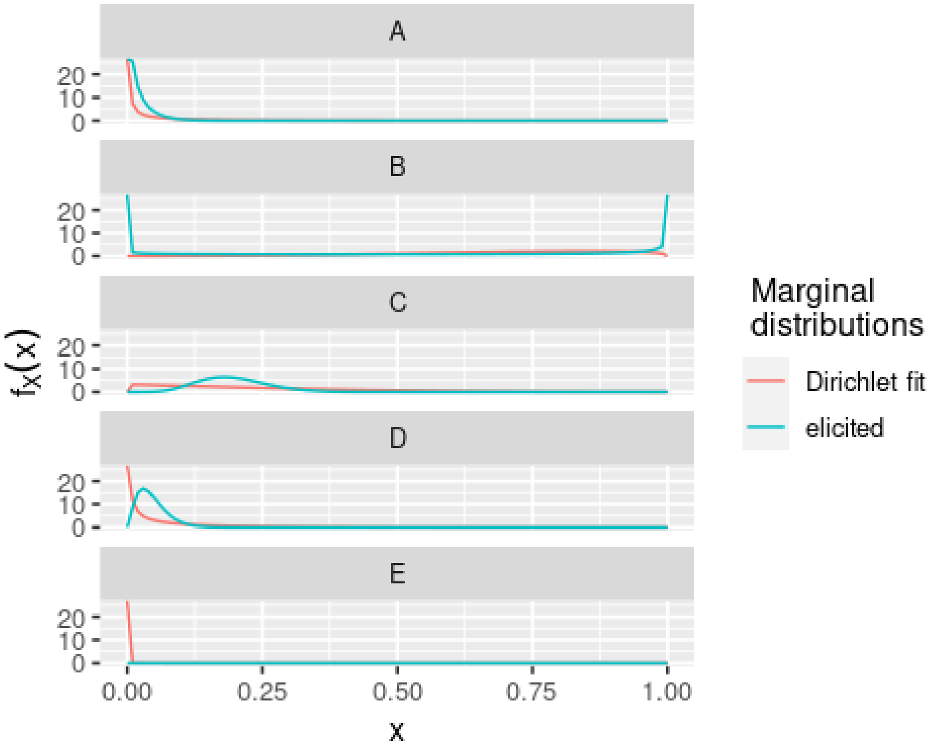

```
##
##         Directly          elicted          beta          marginal          distributions:
##
##                                A          B              C              D               E
##      shape1              0.6590      0.702         7.6600          2.6100     1.80e+00
##        shape2           33.9000        0.480       31.5000       53.9000     7.82e+03
##     mean                       0.0191     0.594         0.1950          0.0461     2.30e-04
##     sd                            0.0230     0.332         0.0625          0.0277     1.72e-04
##      sum                          34.5000      1.180      39.2000      56.6000     7.82e+03
##
##         Sum          of          elicited          marginal          means:          0.855
##
##        Beta        marginal        distributions          from        Dirichlet          fit:
##
##                                  A            B            C            D              E
##         shape1          0.1000         3.120        1.030        0.2420          0.00121
##         shape2          4.3800         1.370        3.460        4.2400          4.48000
##       mean                        0.0223       0.695       0.229       0.0540       0.00027
##      sd                               0.0631       0.197       0.179       0.0965       0.00701
## sum    4.4800 4.480 4.480 4.4800 4.48000
```

Oxynotus centrina

15 December 2021, 13:41

Elicited judgements

quantiles	A	B	C	D	E
0.25	0.6627	1e-04	0.1013	0.0490	1e-04
0.50	0.7630	2e-04	0.1327	0.0517	2e-04
0.75	0.8007	3e-04	0.1660	0.0547	3e-04

Dirichlet density function and parameters

Define the vector of unknown population proportions as

$$\theta := (\theta_1, \ldots, \theta_k),$$

with $= 5$. We write

$$\theta \sim Dirichlet(a_1, \ldots, a_k),$$

with

$$f(\theta) = \frac{\Gamma(a_1 + \cdots + a_k)}{\Gamma(a_1) \ldots \Gamma(a_k)} \prod_{i=1}^{k} \theta_i^{a_i - 1}.$$

The fitted parameter values $a_1, \ldots, a_k$ are as follows:

```
##                 A                B                C                D                E
## 25.40339288  0.00793262  4.71165360  1.78564253  0.00793262
```

Comparing the elicited marginals with the marginals from the Dirichlet fit

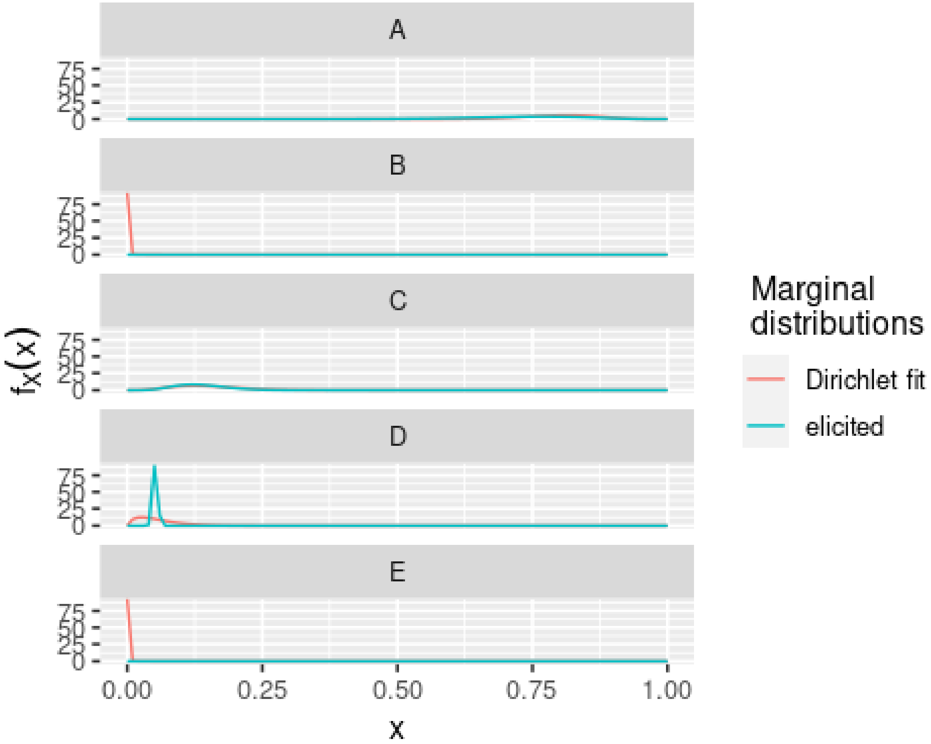


```
##
##        Directly        elicted        beta        marginal        distributions:
##
##                                A                 B              C                 D                 E
##       shape1      12.700      1.80e+00              6.7900      1.43e+02       1.80e+00
##       shape2                   4.490      7.82e+03      42.8000      2.61e+03       7.82e+03
##     mean                          0.738     2.30e-04          0.1370      5.19e-02      2.30e-04
##     sd                               0.103     1.72e-04          0.0483     4.23e-03      1.72e-04
##      sum                          17.100     7.82e+03      49.6000      2.75e+03       7.82e+03
##
##        Sum         of         elicited         marginal         means:            0.927
##
##      Beta       marginal       distributions        from       Dirichlet          fit:
##
##                                  A                 B              C                 D                 E
##      shape1      25.4000      7.93e-03             4.7100           1.7900       7.93e-03
##       shape2                 6.5100       3.19e+01      27.2000      30.1000       3.19e+01
##     mean                      0.7960     2.49e-04        0.1480          0.0559      2.49e-04
##     sd                            0.0702     2.75e-03         0.0618          0.0401      2.75e-03
## sum    31.9000 3.19e+01 31.9000 31.9000 3.19e+01
```

Dalatias licha

15 December 2021, 13:45

Elicited judgements

quantiles	A	B	C	D	E
0.25	0.4743	0.0147	0.1867	0.069	1e-04
0.50	0.5270	0.0230	0.3520	0.094	2e-04
0.75	0.5813	0.0343	0.5450	0.122	3e-04

Dirichlet density function and parameters

Define the vector of unknown population proportions as

$$\theta := (\theta_1, \dots, \theta_k),$$

with $= 5$. We write

$$\theta \sim Dirichlet(a_1, \dots, a_k),$$

with

$$f(\theta) = \frac{\Gamma(a_1 + \dots + a_k)}{\Gamma(a_1) \dots \Gamma(a_k)} \prod_{i=1}^{k} \theta_i^{a_i - 1}.$$

The fitted parameter values $a_1, \dots, a_k$ are as follows:

```
##           A           B           C           D           E
## 6.236078641 0.307619848 4.464359245 1.164827887 0.002725871
```

Comparing the elicited marginals with the marginals from the Dirichlet fit

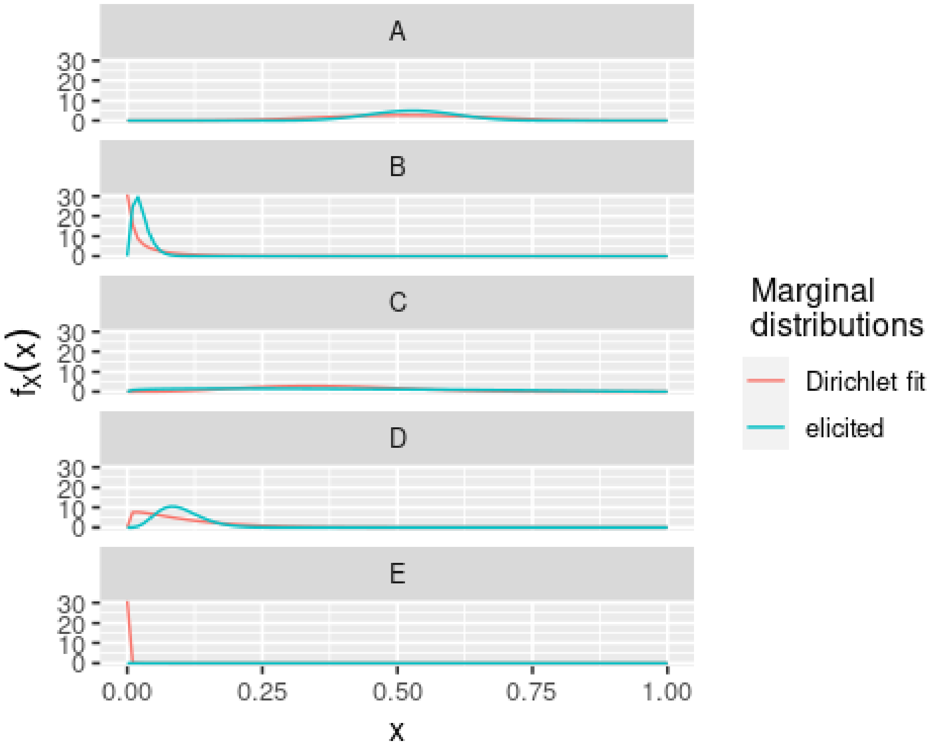

```
##
##         Directly          elicted          beta          marginal          distributions:
##
##                           A              B          C            D            E
##      shape1     21.0000              2.7600     1.300        5.3900     1.80e+00
##        shape2       18.9000      103.0000     2.140      49.3000     7.82e+03
##     mean                 0.5270            0.0260    0.377        0.0985     2.30e-04
##     sd                      0.0781           0.0154    0.230        0.0399    1.72e-04
##      sum                    39.9000     106.0000     3.440      54.7000     7.82e+03
##
##         Sum          of          elicited          marginal          means:          1.029
##
##        Beta        marginal        distributions         from        Dirichlet         fit:
##
##                          A              B            C             D            E
##      shape1        6.240         0.3080          4.460         1.1600     2.73e-03
##       shape2          5.940      11.9000          7.710      11.0000     1.22e+01
##     mean                0.512         0.0253        0.367         0.0957     2.24e-04
##     sd                     0.138          0.0432        0.133         0.0810     4.12e-03
## sum    12.200 12.2000 12.200 12.2000 1.22e+01
```

Hexanchus nakamurrai

15 December 2021, 13:49

Elicited judgements

quantiles	A	B	C	D	E
0.25	0.4813	1e-04	0.2230	0.0312	1e-04
0.50	0.5757	2e-04	0.3077	0.0407	2e-04
0.75	0.6583	3e-04	0.3987	0.0517	3e-04

Dirichlet density function and parameters

Define the vector of unknown population proportions as

$$\theta := (\theta_1, \ldots, \theta_k),$$

with $= 5$. We write

$$\theta \sim Dirichlet(a_1, \ldots, a_k),$$

with

$$f(\theta) = \frac{\Gamma(a_1 + \cdots + a_k)}{\Gamma(a_1) \ldots \Gamma(a_k)} \prod_{i=1}^{k} \theta_i^{a_i - 1}.$$

The fitted parameter values $a_1, \ldots, a_k$ are as follows:

```
##                  A                  B                  C                  D                  E
## 9.492032953 0.003837235 5.273194291 0.707128972 0.003837235
```

Comparing the elicited marginals with the marginals from the Dirichlet fit

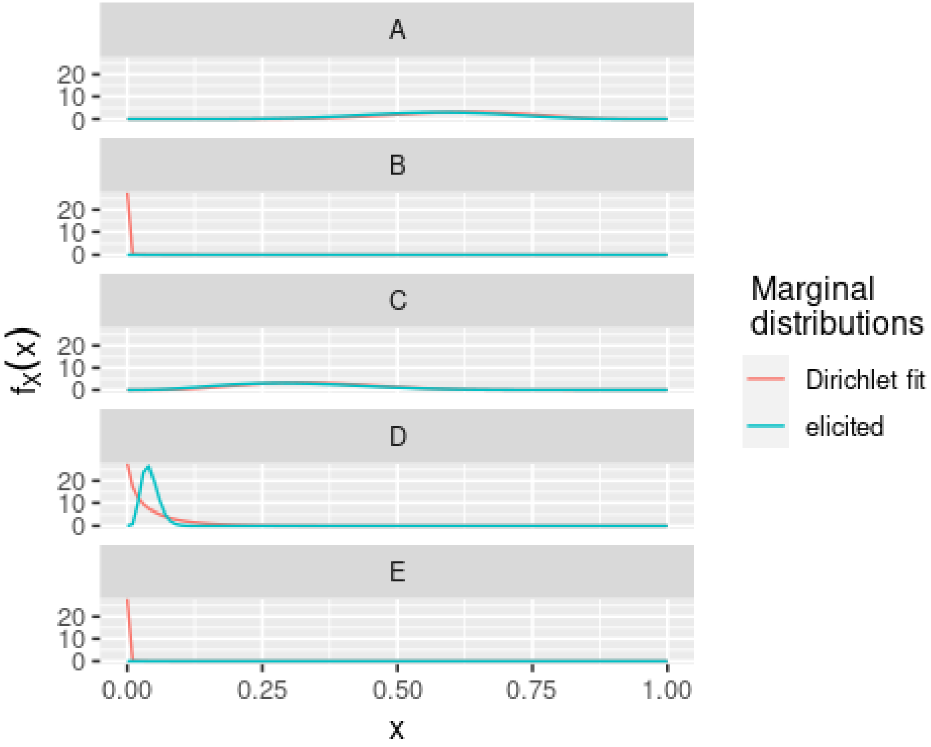


```
##
## Directly elicted beta marginal distributions:
##
##             A        B      C        D        E
## shape1  8.250 1.80e+00  4.040   7.1500 1.80e+00
## shape2  6.220 7.82e+03  8.710 161.0000 7.82e+03
## mean    0.570 2.30e-04  0.317   0.0425 2.30e-04
## sd      0.126 1.72e-04  0.125   0.0155 1.72e-04
## sum    14.500 7.82e+03 12.700 168.0000 7.82e+03
##
## Sum of elicited marginal means: 0.929
##
## Beta marginal distributions from Dirichlet fit:
##
##             A        B      C        D        E
## shape1  9.490 3.84e-03  5.270   0.7070 3.84e-03
## shape2  5.990 1.55e+01 10.200  14.8000 1.55e+01
## mean    0.613 2.48e-04  0.341   0.0457 2.48e-04
## sd      0.120 3.88e-03  0.117   0.0514 3.88e-03
## sum    15.500 1.55e+01 15.500  15.5000 1.55e+01
```

Sphyrna lewini

15 December 2021, 13:55

Elicited judgements

quantiles	A	B	C	D	E
0.25	0.0033	0.3067	0.0751	0.0128	0.0050
0.50	0.0109	0.8073	0.0801	0.0151	0.0250
0.75	0.0264	0.9223	0.0853	0.0176	0.1893

Dirichlet density function and parameters

Define the vector of unknown population proportions as

$$\theta := (\theta_1, \dots, \theta_k),$$

with $= 5$. We write

$$\theta \sim Dirichlet(a_1, \dots, a_k),$$

with

$$f(\theta) = \frac{\Gamma(a_1 + \dots + a_k)}{\Gamma(a_1) \dots \Gamma(a_k)} \prod_{i=1}^{k} \theta_i^{a_i - 1}.$$

The fitted parameter values $a_1, \dots, a_k$ are as follows:

```
##          A          B          C          D          E
## 0.06338978 2.10823531 0.26671972 0.05101086 0.41267075
```

Comparing the elicited marginals with the marginals from the Dirichlet fit

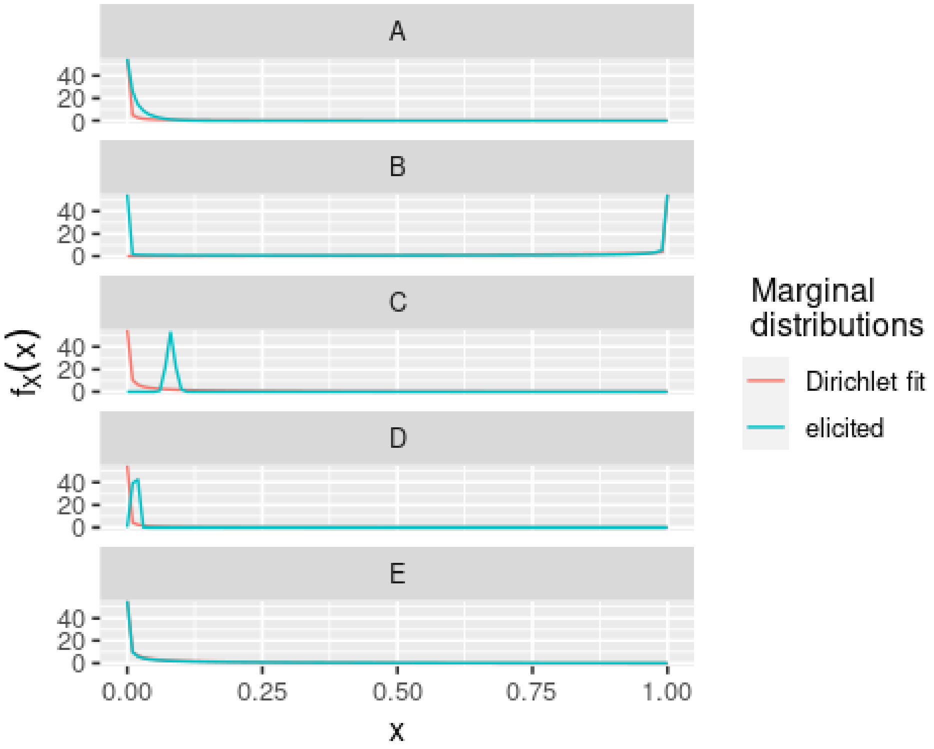

```
##
##      Directly       elicted      beta      marginal      distributions:
##
##                        A           B             C              D         E
##      shape1         0.6590       0.682      1.04e+02      1.80e+01     0.280
##       shape2     33.9000      0.392      1.19e+03      1.15e+03      1.970
##     mean                    0.0191     0.635     8.03e-02     1.54e-02    0.124
##     sd                          0.0230     0.334     7.57e-03     3.59e-03    0.183
##     sum                     34.5000     1.070     1.29e+03     1.17e+03    2.250
##
##      Sum      of      elicited      marginal      means:      0.874
##
##      Beta      marginal      distributions      from      Dirichlet      fit:
##
##                             A           B              C              D          E
##      shape1         0.0634        2.110        0.2670        0.0510        0.413
##      shape2         2.8400        0.794        2.6400        2.8500        2.490
##     mean                    0.0218       0.726      0.0919      0.0176      0.142
##     sd                          0.0740      0.226      0.1460      0.0665      0.177
## sum    2.9000 2.900 2.9000 2.9000 2.900
```

Scyliorhinus stellaris

15 December 2021, 14:01

Elicited judgements

quantiles	A	B	C	D	E
0.25	0.3443	1e-04	0.0553	0.2673	1e-04
0.50	0.4777	2e-04	0.1177	0.4060	2e-04
0.75	0.6030	3e-04	0.2500	0.5360	3e-04

Dirichlet density function and parameters

Define the vector of unknown population proportions as

$$\theta := (\theta_1, \dots, \theta_k),$$

with $= 5$. We write

$$\theta \sim Dirichlet(a_1, \dots, a_k),$$

with

$$f(\theta) = \frac{\Gamma(a_1 + \dots + a_k)}{\Gamma(a_1) \dots \Gamma(a_k)} \prod_{i=1}^{k} \theta_i^{a_i - 1}.$$

The fitted parameter values $a_1, \dots, a_k$ are as follows:

```
##           A             B             C             D             E
## 2.886362591 0.001394191 1.002807509 2.484490046 0.001394191
```

Comparing the elicited marginals with the marginals from the Dirichlet fit

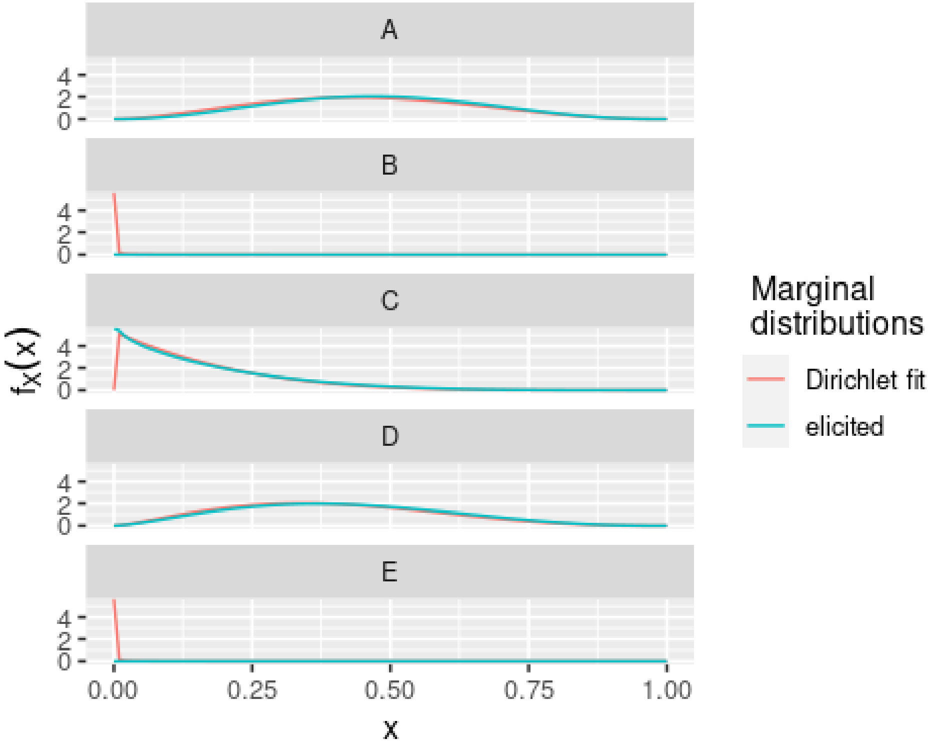


```
##
##     Directly    elicted    beta    marginal    distributions:
##
##                    A          B          C        D          E
##     shape1     3.350   1.80e+00   0.920    2.560   1.80e+00
##     shape2     3.670   7.82e+03   4.630    3.680   7.82e+03
##     mean           0.477   2.30e-04   0.166    0.411   2.30e-04
##     sd                0.176    1.72e-04   0.145    0.183   1.72e-04
##     sum             7.020    7.82e+03   5.550    6.240   7.82e+03
##
##     Sum     of     elicited     marginal     means:     1.054
##
##     Beta     marginal     distributions     from     Dirichlet     fit:
##
##                    A          B          C        D          E
##     shape1     2.890   0.001390   1.000    2.48    0.001390
##     shape2     3.490   6.380000   5.370    3.89    6.380000
##     mean           0.453   0.000219   0.157    0.39    0.000219
##     sd                0.183   0.005440   0.134    0.18    0.005440
## sum    6.380 6.380000 6.380 6.38 6.380000
```

Echinorhinus brucus

15 December 2021, 14:05

Elicited judgements

quantiles	A	B	C	D	E
0.25	0.6313	0.0033	0.1243	0.0206	1e-04
0.50	0.7053	0.0109	0.1860	0.0343	2e-04
0.75	0.7370	0.0264	0.2647	0.0527	3e-04

Dirichlet density function and parameters

Define the vector of unknown population proportions as

$$\theta := (\theta_1, \dots, \theta_k),$$

with $= 5$. We write

$$\theta \sim Dirichlet(a_1, \dots, a_k),$$

with

$$f(\theta) = \frac{\Gamma(a_1 + \cdots + a_k)}{\Gamma(a_1) \dots \Gamma(a_k)} \prod_{i=1}^{k} \theta_i^{a_i - 1}.$$

The fitted parameter values $a_1, \dots, a_k$ are as follows:

```
##                    A                B                C                D                E
## 17.493004561  0.483826333  5.097611793  1.000147788  0.005839957
```

Comparing the elicited marginals with the marginals from the Dirichlet fit

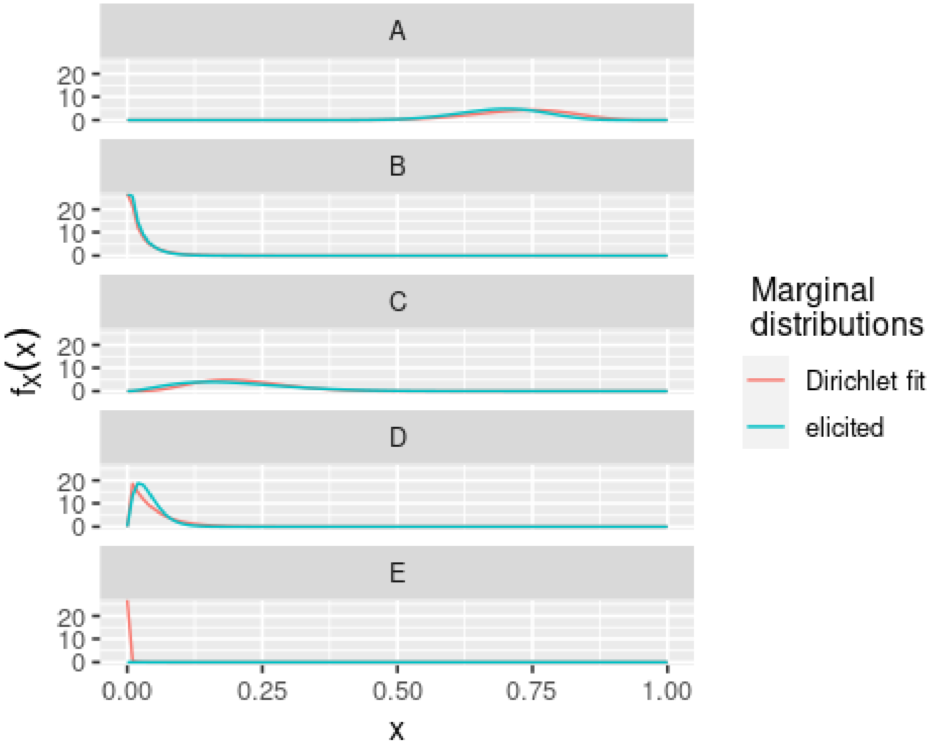

```
##
##          Directly          elicted          beta          marginal          distributions:
##
##                             A                B              C               D                E
##      shape1      22.5000          0.6590           2.870           2.2800      1.80e+00
##        shape2         10.1000         33.9000       11.400        55.5000      7.82e+03
##     mean                     0.6900          0.0191          0.201          0.0395      2.30e-04
##     sd                          0.0798          0.0230          0.103          0.0254      1.72e-04
##      sum                          32.6000      34.5000      14.300      57.8000      7.82e+03
##
##          Sum           of          elicited           marginal           means:           0.95
##
##        Beta         marginal        distributions          from         Dirichlet          fit:
##
##                               A                B               C               D                E
##      shape1      17.500           0.4840           5.1000          1.0000      5.84e-03
##       shape2                   6.590         23.6000        19.0000        23.1000      2.41e+01
##     mean                      0.726         0.0201          0.2120          0.0415      2.43e-04
##     sd                          0.089           0.0280          0.0816          0.0398      3.11e-03
## sum    24.100 24.1000 24.1000 24.1000 2.41e+01
```

Hexanchus griseus

15 December 2021, 14:13

Elicited judgements

quantiles	A	B	C	D	E
0.25	0.3083	0.0490	0.2370	0.0500	0.0268
0.50	0.3810	0.0517	0.3143	0.0787	0.0457
0.75	0.4580	0.0547	0.3980	0.1137	0.0707

Dirichlet density function and parameters

Define the vector of unknown population proportions as

$$\theta := (\theta_1, \ldots, \theta_k),$$

with $= 5$. We write

$$\theta \sim Dirichlet(a_1, \ldots, a_k),$$

with

$$f(\theta) = \frac{\Gamma(a_1 + \cdots + a_k)}{\Gamma(a_1) \ldots \Gamma(a_k)} \prod_{i=1}^{k} \theta_i^{a_i - 1}.$$

The fitted parameter values $a_1, \ldots, a_k$ are as follows:

```
##                 A                B                C                D                E
## 9.931598 1.337371 8.299815 2.236813 1.359687
```

Comparing the elicited marginals with the marginals from the Dirichlet fit

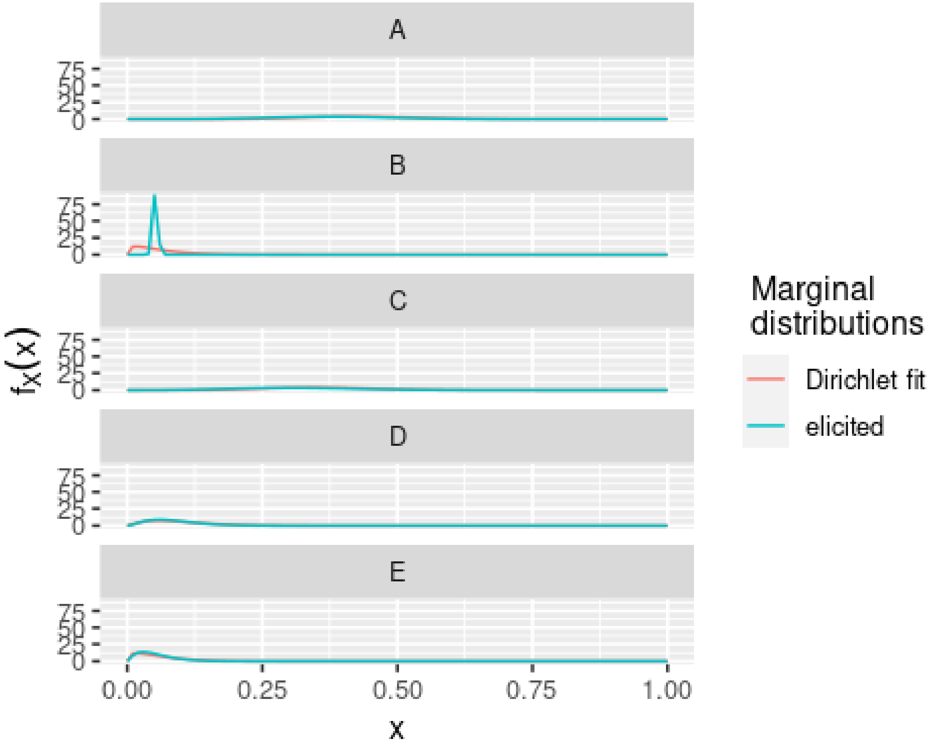


```
##
##        Directly        elicted        beta        marginal        distributions:
##
##                          A              B             C              D              E
##     shape1           7.480     1.43e+02         4.940         2.7800         2.1400
##        shape2         11.900        2.61e+03        10.400        29.3000        38.4000
##    mean                    0.385     5.19e-02        0.322        0.0867         0.0527
##    sd                         0.108     4.23e-03        0.116         0.0489         0.0347
##      sum                        19.400      2.75e+03      15.400       32.1000       40.5000
##
##        Sum         of         elicited         marginal         means:          0.898
##
##       Beta        marginal        distributions        from        Dirichlet        fit:
##
##                          A              B             C              D              E
##     shape1          9.930         1.3400         8.3000         2.2400         1.3600
##        shape2         13.200         21.8000        14.9000        20.9000        21.8000
##    mean                   0.429          0.0577         0.3580         0.0966         0.0587
##    sd                         0.101          0.0474         0.0975         0.0601         0.0478
## sum    23.200 23.2000 23.2000 23.2000 23.2000
```

Sphyrna zigaena

15 December 2021, 14:18

Elicited judgements

quantiles	A	B	C	D	E
0.25	0.0033	0.3067	0.0751	0.0128	0.0050
0.50	0.0109	0.8073	0.0801	0.0151	0.0250
0.75	0.0264	0.9223	0.0853	0.0176	0.1893

Dirichlet density function and parameters

Define the vector of unknown population proportions as

$$\theta := (\theta_1, \ldots, \theta_k),$$

with = 5 . We write

$$\theta \sim Dirichlet(a_1, \ldots, a_k),$$

with

$$f(\theta) = \frac{\Gamma(a_1 + \cdots + a_k)}{\Gamma(a_1) \ldots \Gamma(a_k)} \prod_{i=1}^{k} \theta_i^{a_i - 1}.$$

The fitted parameter values $a_1, \ldots, a_k$ are as follows:

```
##          A          B          C          D          E
## 0.06338978 2.10823531 0.26671972 0.05101086 0.41267075
```

Comparing the elicited marginals with the marginals from the Dirichlet fit

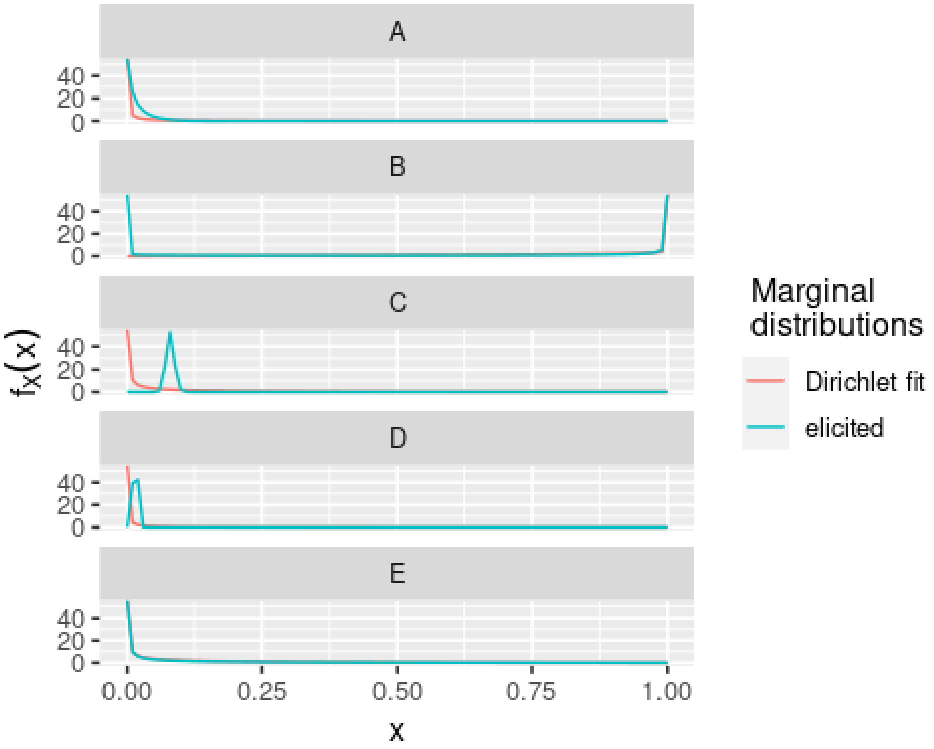

```
##
##      Directly      elicted      beta      marginal      distributions:
##
##                          A          B             C              D        E
##      shape1          0.6590      0.682      1.04e+02      1.80e+01    0.280
##       shape2        33.9000      0.392      1.19e+03      1.15e+03    1.970
##     mean                 0.0191     0.635      8.03e-02      1.54e-02    0.124
##    sd                       0.0230     0.334      7.57e-03      3.59e-03    0.183
##     sum                 34.5000      1.070      1.29e+03      1.17e+03    2.250
##
##      Sum      of      elicited      marginal      means:      0.874
##
##      Beta      marginal      distributions      from      Dirichlet      fit:
##
##                            A           B            C             D        E
##       shape1         0.0634       2.110       0.2670       0.0510    0.413
##       shape2         2.8400       0.794       2.6400       2.8500    2.490
##      mean                  0.0218      0.726       0.0919       0.0176    0.142
##     sd                        0.0740      0.226       0.1460       0.0665    0.177
## sum    2.9000 2.900 2.9000 2.9000 2.900
```

Carcharodon carcharias

15 December 2021, 14:23

Elicited judgements

quantiles	A	B	C	D	E
0.25	0.1205	0.2547	0.0526	0.0365	0.0128
0.50	0.1594	0.6093	0.0733	0.0523	0.0151
0.75	0.2039	0.8630	0.0988	0.0728	0.0176

Dirichlet density function and parameters

Define the vector of unknown population proportions as

$$\theta := (\theta_1, \ldots, \theta_k),$$

with $= 5$. We write

$$\theta \sim Dirichlet(a_1, \ldots, a_k),$$

with

$$f(\theta) = \frac{\Gamma(a_1 + \cdots + a_k)}{\Gamma(a_1) \ldots \Gamma(a_k)} \prod_{i=1}^{k} \theta_i^{a_i - 1}.$$

The fitted parameter values $a_1, \ldots, a_k$ are as follows:

```
##                 A           B           C           D           E
## 1.1054832 3.7317269 0.5219331 0.3788325 0.1024992
```

Comparing the elicited marginals with the marginals from the Dirichlet fit

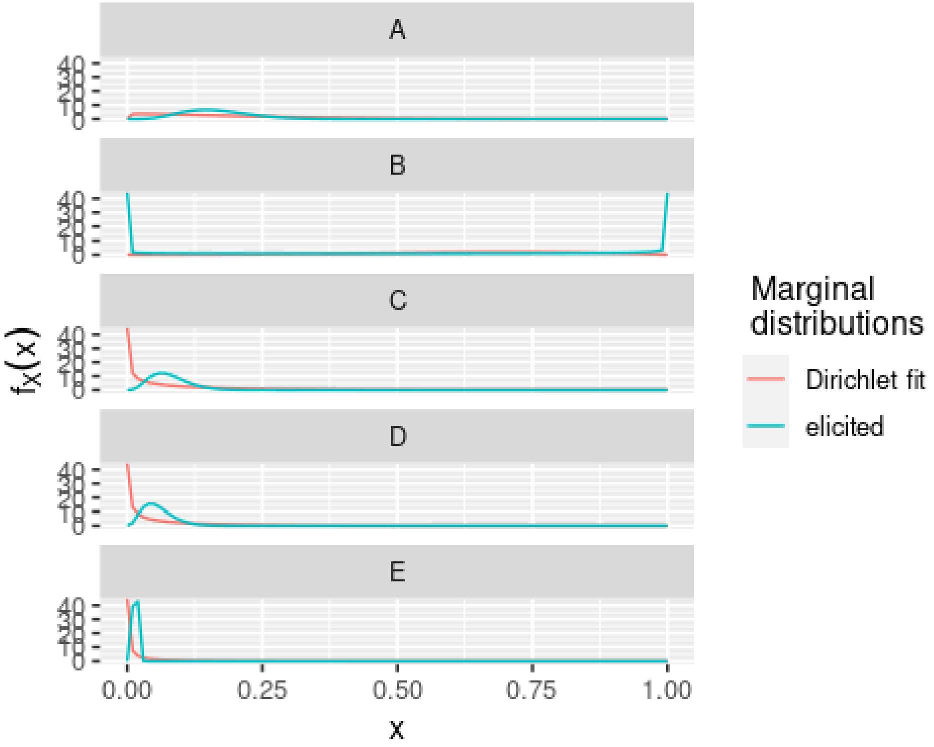


```
##
##        Directly        elicted        beta        marginal        distributions:
##
##                              A           B              C              D              E
##     shape1              5.8300      0.740         4.5400         3.9000      1.80e+01
##       shape2           29.4000        0.583        53.4000       64.8000      1.15e+03
##    mean                         0.1660      0.559         0.0782          0.0568      1.54e-02
##    sd                               0.0618     0.326          0.0350          0.0277      3.59e-03
##     sum                            35.2000       1.320       58.0000        68.7000      1.17e+03
##
##        Sum          of          elicited          marginal          means:          0.875
##
##       Beta        marginal        distributions         from         Dirichlet         fit:
##
##                              A              B              C              D              E
##        shape1          1.110          3.730          0.5220         0.3790         0.1020
##        shape2          4.730          2.110          5.3200         5.4600         5.7400
##      mean                        0.189         0.639        0.0894         0.0649        0.0175
##       sd                                0.150        0.184        0.1090         0.0942        0.0502
## sum     5.840 5.840 5.8400 5.8400 5.8400
```

Cetorhinus maximus

15 December 2021, 14:28

Elicited judgements

quantiles	A	B	C	D	E
0.25	0.1026	1e-04	0.1160	0.3480	0.0158
0.50	0.1543	2e-04	0.2000	0.5340	0.0832
0.75	0.2190	3e-04	0.3423	0.6953	0.2820

Dirichlet density function and parameters

Define the vector of unknown population proportions as

$$\theta := (\theta_1, \dots, \theta_k),$$

with = 5 . We write

$$\theta \sim Dirichlet(a_1, \dots, a_k),$$

with

$$f(\theta) = \frac{\Gamma(a_1 + \cdots + a_k)}{\Gamma(a_1) \dots \Gamma(a_k)} \prod_{i=1}^{k} \theta_i^{a_i - 1}.$$

The fitted parameter values $a_1, \dots, a_k$ are as follows:

```
##           A          B          C          D          E
## 0.74899840 0.00103221 1.06388895 2.34672907 0.80124306
```

Comparing the elicited marginals with the marginals from the Dirichlet fit

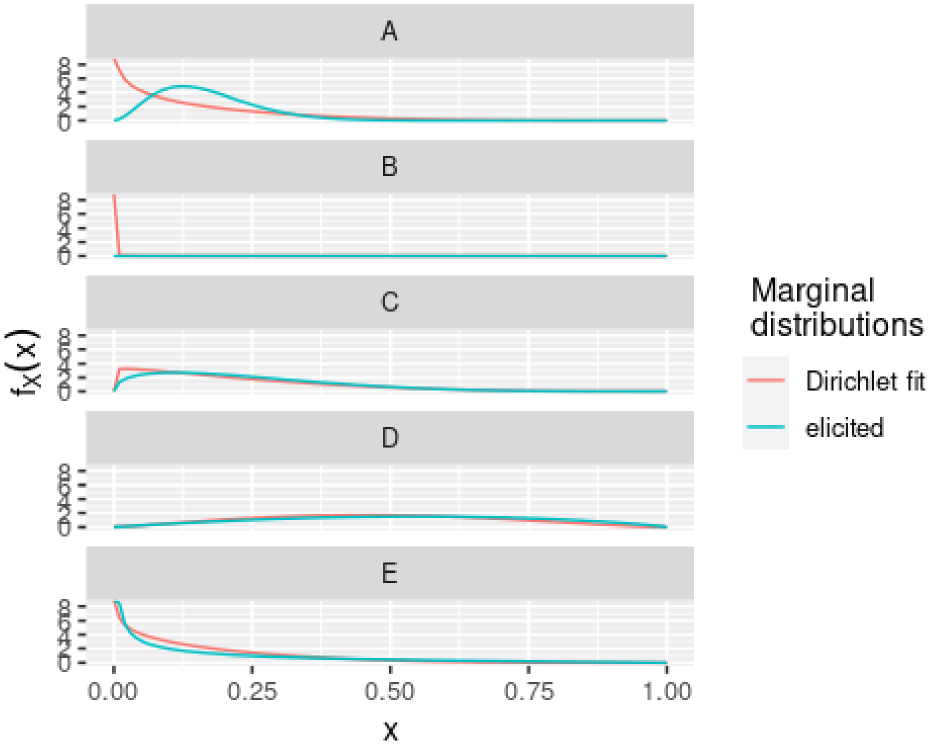

```
##
##        Directly        elicited        beta        marginal        distributions:
##
##                       A              B          C          D          E
##       shape1     2.9600      1.80e+00      1.460      2.090      0.396
##        shape2   14.8000      7.82e+03      4.680      1.900      1.820
##      mean          0.1670    2.30e-04      0.237      0.524      0.179
##     sd             0.0862    1.72e-04      0.159      0.224      0.214
##      sum          17.7000    7.82e+03      6.140      3.980      2.220
##
##        Sum        of        elicited        marginal        means:        1.108
##
##       Beta       marginal       distributions       from       Dirichlet       fit:
##
##                      A                  B          C          D          E
##        shape1     0.749          0.001030      1.060      2.350      0.801
##        shape2     4.210          4.960000      3.900      2.620      4.160
##       mean           0.151       0.000208      0.214      0.473      0.161
##      sd              0.147       0.005910      0.168      0.204      0.151
## sum    4.960 4.960000 4.960 4.960 4.960
```

Lamna nasus

15 December 2021, 14:33

Elicited judgements

quantiles	A	B	C	D	E
0.25	0.0291	0.2690	0.1173	0.0287	0.0133
0.50	0.0515	0.6697	0.1507	0.0331	0.0401
0.75	0.0876	0.8767	0.1862	0.0378	0.2069

Dirichlet density function and parameters

Define the vector of unknown population proportions as

$$\theta := (\theta_1, \ldots, \theta_k),$$

with $= 5$. We write

$$\theta \sim Dirichlet(a_1, \ldots, a_k),$$

with

$$f(\theta) = \frac{\Gamma(a_1 + \cdots + a_k)}{\Gamma(a_1) \ldots \Gamma(a_k)} \prod_{i=1}^{k} \theta_i^{a_i - 1}.$$

The fitted parameter values $a_1, \ldots, a_k$ are as follows:

```
##                 A               B               C               D               E
## 0.2882188 2.6552542 0.7061172 0.1528819 0.5939962
```

Comparing the elicited marginals with the marginals from the Dirichlet fit

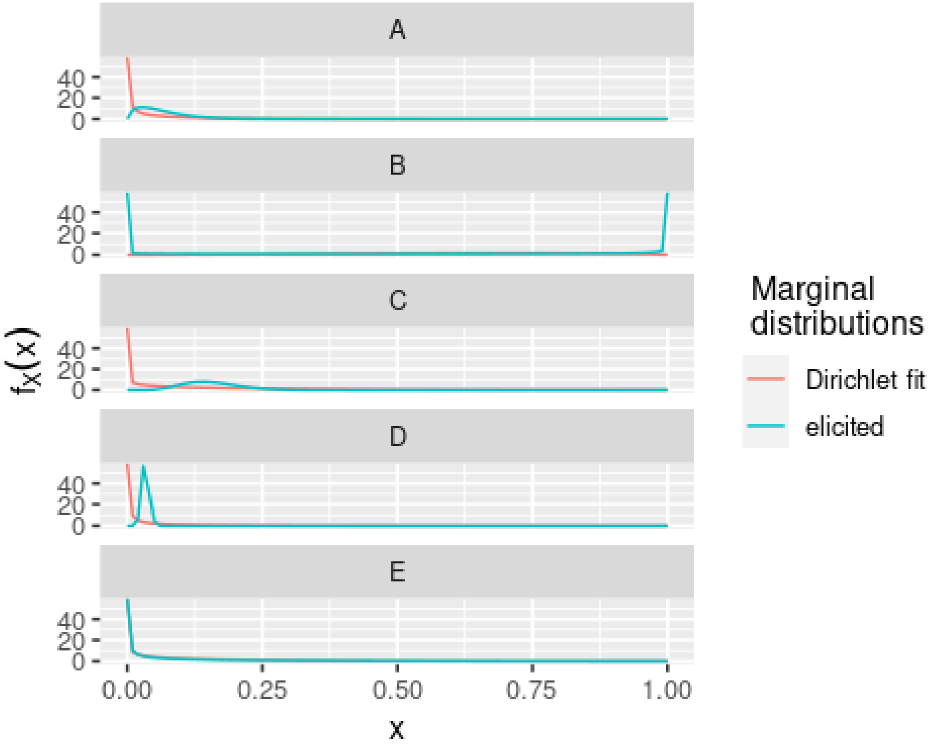


```
##
## Directly elicted beta marginal distributions:
##
##            A     B       C        D     E
## shape1  1.6800 0.748  7.5600 2.35e+01 0.379
## shape2 24.9000 0.537 41.3000 6.78e+02 2.530
## mean    0.0632 0.582  0.1550 3.35e-02 0.130
## sd      0.0463 0.326  0.0512 6.79e-03 0.170
## sum    26.6000 1.290 48.8000 7.01e+02 2.910
##
## Sum of elicited marginal means: 0.964
##
## Beta marginal distributions from Dirichlet fit:
##
##             A     B     C      D     E
## shape1 0.2880 2.660 0.706 0.1530 0.594
## shape2 4.1100 1.740 3.690 4.2400 3.800
## mean   0.0656 0.604 0.161 0.0348 0.135
## sd     0.1070 0.211 0.158 0.0789 0.147
## sum    4.4000 4.400 4.400 4.4000 4.400
```

Sphyrna mokarran

15 December 2021, 14:38

Elicited judgements

quantiles	A	B	C	D	E
0.25	0.0033	0.3067	0.0751	0.0128	0.0050
0.50	0.0109	0.8073	0.0801	0.0151	0.0250
0.75	0.0264	0.9223	0.0853	0.0176	0.1893

Dirichlet density function and parameters

Define the vector of unknown population proportions as

$$\theta := (\theta_1, \dots, \theta_k),$$

with $= 5$. We write

$$\theta \sim Dirichlet(a_1, \dots, a_k),$$

with

$$f(\theta) = \frac{\Gamma(a_1 + \dots + a_k)}{\Gamma(a_1) \dots \Gamma(a_k)} \prod_{i=1}^{k} \theta_i^{a_i - 1}.$$

The fitted parameter values $a_1, \dots, a_k$ are as follows:

```
##              A            B            C            D            E
## 0.06338978 2.10823531 0.26671972 0.05101086 0.41267075
```

Comparing the elicited marginals with the marginals from the Dirichlet fit

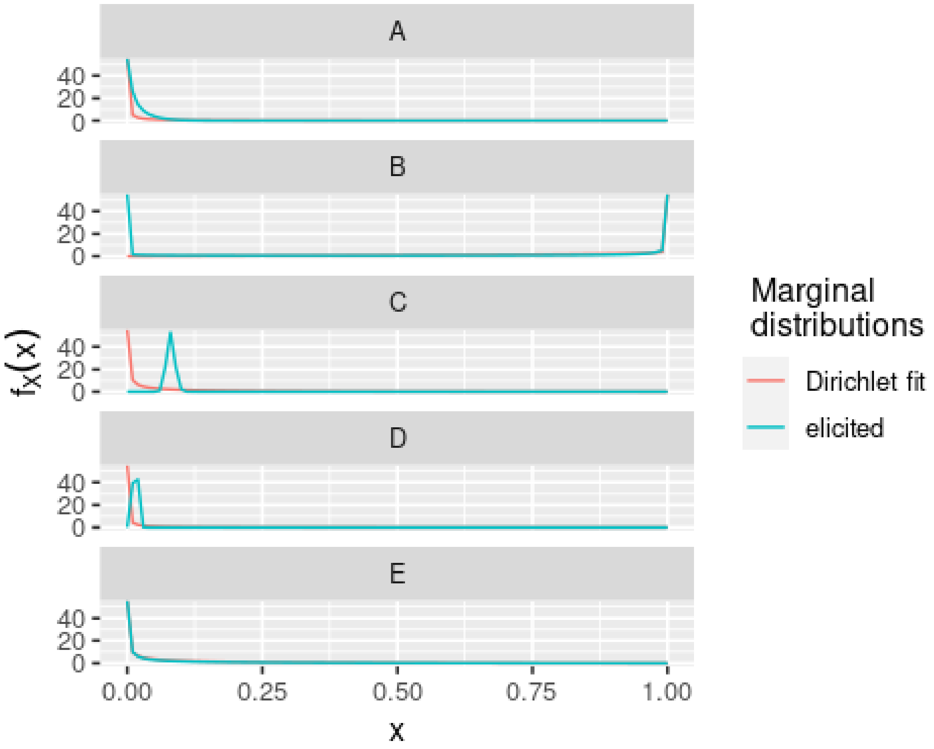

```
##
##         Directly        elicted        beta        marginal        distributions:
##
##                              A          B            C              D           E
##       shape1             0.6590      0.682      1.04e+02      1.80e+01      0.280
##        shape2      33.9000      0.392      1.19e+03      1.15e+03      1.970
##      mean                         0.0191     0.635     8.03e-02     1.54e-02     0.124
##     sd                              0.0230     0.334     7.57e-03     3.59e-03     0.183
##      sum                        34.5000     1.070     1.29e+03     1.17e+03     2.250
##
##         Sum         of         elicited         marginal         means:         0.874
##
##        Beta       marginal       distributions        from        Dirichlet        fit:
##
##                                A            B              C              D              E
##         shape1         0.0634         2.110         0.2670         0.0510         0.413
##         shape2         2.8400         0.794         2.6400         2.8500         2.490
##        mean                         0.0218        0.726        0.0919        0.0176        0.142
##      sd                                0.0740       0.226       0.1460       0.0665       0.177
## sum    2.9000 2.900 2.9000 2.9000 2.900
```

Carcharhinus brachyurus

15 December 2021, 14:44

Elicited judgements

quantiles	A	B	C	D	E
0.25	0.0192	0.1832	0.1720	0.0960	0.0026
0.50	0.0289	0.5087	0.2697	0.1352	0.0361
0.75	0.0465	0.6627	0.4237	0.1827	0.2233

Dirichlet density function and parameters

Define the vector of unknown population proportions as

$$\theta := (\theta_1, \ldots, \theta_k),$$

with $= 5$. We write

$$\theta \sim Dirichlet(a_1, \ldots, a_k),$$

with

$$f(\theta) = \frac{\Gamma(a_1 + \cdots + a_k)}{\Gamma(a_1) \ldots \Gamma(a_k)} \prod_{i=1}^{k} \theta_i^{a_i - 1}.$$

The fitted parameter values $a_1, \ldots, a_k$ are as follows:

```
##                    A                 B                 C                 D                 E
## 0.1266820 1.7135712 1.1172640 0.5316236 0.5619740
```

Comparing the elicited marginals with the marginals from the Dirichlet fit

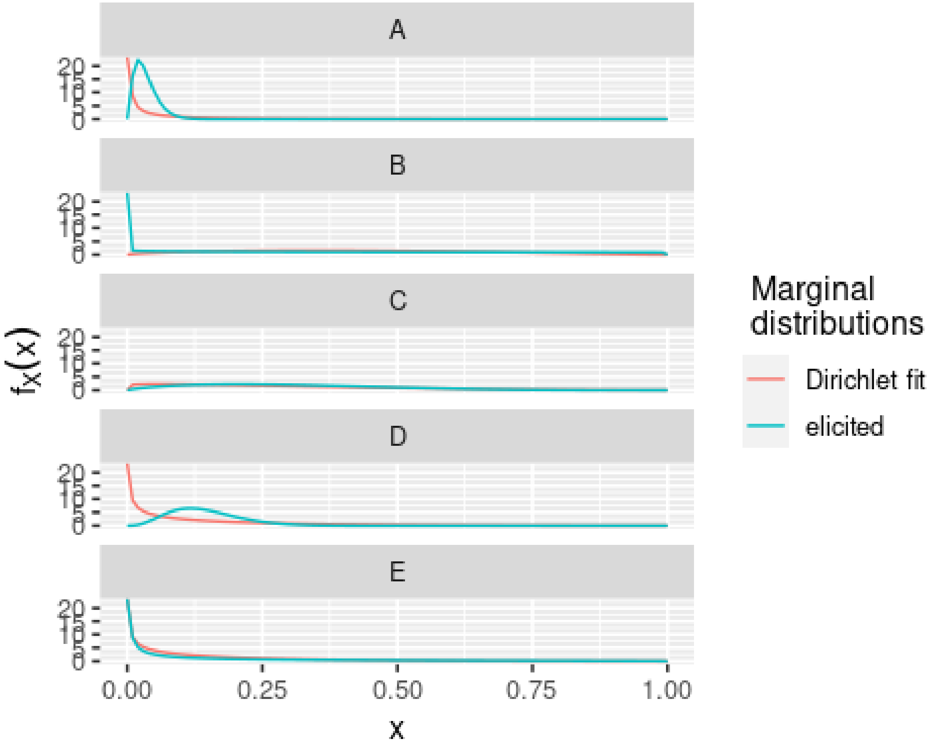


```
##         Directly        elicted        beta        marginal        distributions:
##
##                              A           B           C             D           E
##       shape1              2.4700      0.887      1.830         4.0900      0.246
##         shape2         69.6000        1.030        4.220      24.4000       1.370
##      mean                      0.0342     0.463     0.302           0.1440    0.152
##     sd                             0.0213     0.292    0.173           0.0646    0.222
##      sum                        72.0000     1.920      6.050      28.5000     1.620
##
##         Sum        of        elicited        marginal        means:           1.095
##
##        Beta       marginal       distributions        from       Dirichlet        fit:
##
##                               A            B            C            D            E
##         shape1         0.1270         1.710        1.120        0.532        0.562
##         shape2         3.9200         2.340        2.930        3.520        3.490
##        mean                  0.0313      0.423       0.276       0.131        0.139
##       sd                          0.0774      0.220       0.199       0.150       0.154
## sum     4.0500 4.050 4.050 4.050 4.050
```

Centrophorus uyato

15 December 2021, 14:48

Elicited judgements

quantiles	A	B	C	D	E
0.25	0.6297	1e-04	0.1396	0.0159	1e-04
0.50	0.7730	2e-04	0.1970	0.0180	2e-04
0.75	0.8863	3e-04	0.2832	0.0202	3e-04

Dirichlet density function and parameters

Define the vector of unknown population proportions as

$$\theta := (\theta_1, \ldots, \theta_k),$$

with = 5 . We write

$$\theta \sim Dirichlet(a_1, \ldots, a_k),$$

with

$$f(\theta) = \frac{\Gamma(a_1 + \cdots + a_k)}{\Gamma(a_1) \ldots \Gamma(a_k)} \prod_{i=1}^{k} \theta_i^{a_i - 1}.$$

The fitted parameter values $a_1, \ldots, a_k$ are as follows:

```
##           A           B           C           D           E
## 6.600886200 0.002052482 1.913816457 0.161822074 0.002052482
```

Comparing the elicited marginals with the marginals from the Dirichlet fit

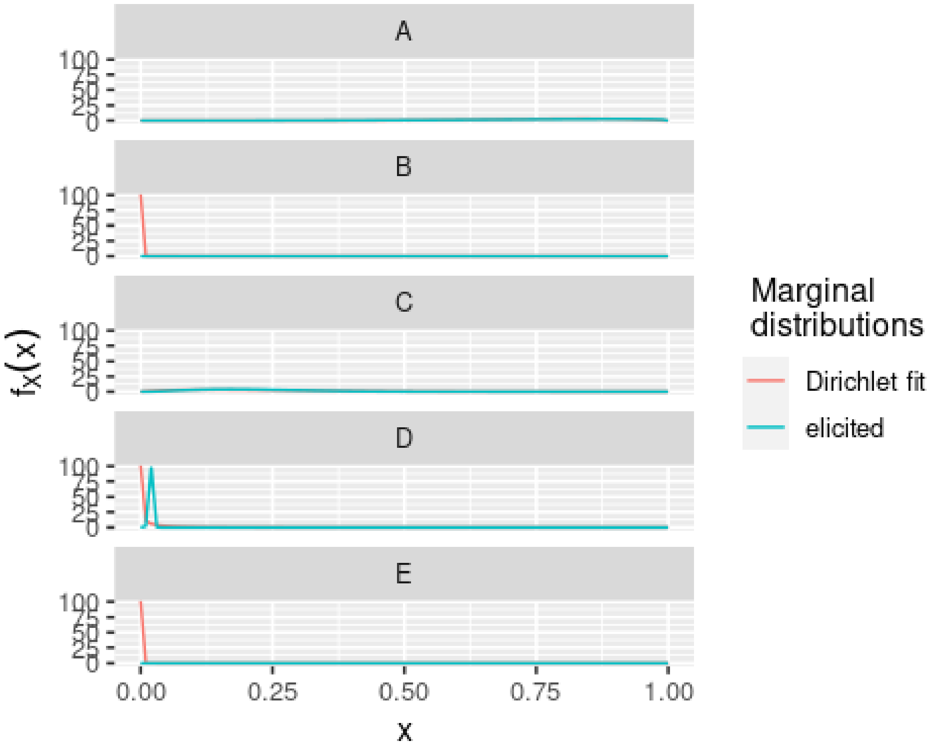

```
##
##        Directly       elicted       beta       marginal       distributions:
##
##                          A                 B           C             D            E
##      shape1       3.680       1.80e+00             3.120       3.15e+01     1.80e+00
##       shape2       1.290        7.82e+03       11.400       1.70e+03     7.82e+03
##     mean                0.741      2.30e-04          0.215      1.82e-02     2.30e-04
##    sd                       0.179      1.72e-04         0.104      3.21e-03     1.72e-04
##     sum                     4.970      7.82e+03     14.500      1.73e+03     7.82e+03
##
##        Sum        of        elicited        marginal        means:        0.974
##
##       Beta       marginal       distributions       from       Dirichlet       fit:
##
##                            A                   B          C            D            E
##       shape1        6.600        0.002050      1.910       0.1620     0.002050
##       shape2        2.080        8.680000      6.770       8.5200     8.680000
##      mean                  0.760      0.000236      0.220      0.0186     0.000236
##     sd                            0.137      0.004940     0.133      0.0435     0.004940
## sum    8.680 8.680000 8.680 8.6800 8.680000
```

Carcharhinus obscurus

15 December 2021, 14:53

Elicited judgements

quantiles	A	B	C	D	E
0.25	0.0163	0.2400	0.1567	0.0737	1e-04
0.50	0.0365	0.6247	0.2503	0.1115	2e-04
0.75	0.0700	0.8393	0.4160	0.1590	3e-04

Dirichlet density function and parameters

Define the vector of unknown population proportions as

$$\theta := (\theta_1, \ldots, \theta_k),$$

with $= 5$. We write

$$\theta \sim Dirichlet(a_1, \ldots, a_k),$$

with

$$f(\theta) = \frac{\Gamma(a_1 + \cdots + a_k)}{\Gamma(a_1) \ldots \Gamma(a_k)} \prod_{i=1}^{k} \theta_i^{a_i - 1}.$$

The fitted parameter values $a_1, \ldots, a_k$ are as follows:

```
##              A             B             C             D             E
## 0.1945440210 2.1660407201 1.1336933073 0.4760066303 0.0009013508
```

Comparing the elicited marginals with the marginals from the Dirichlet fit

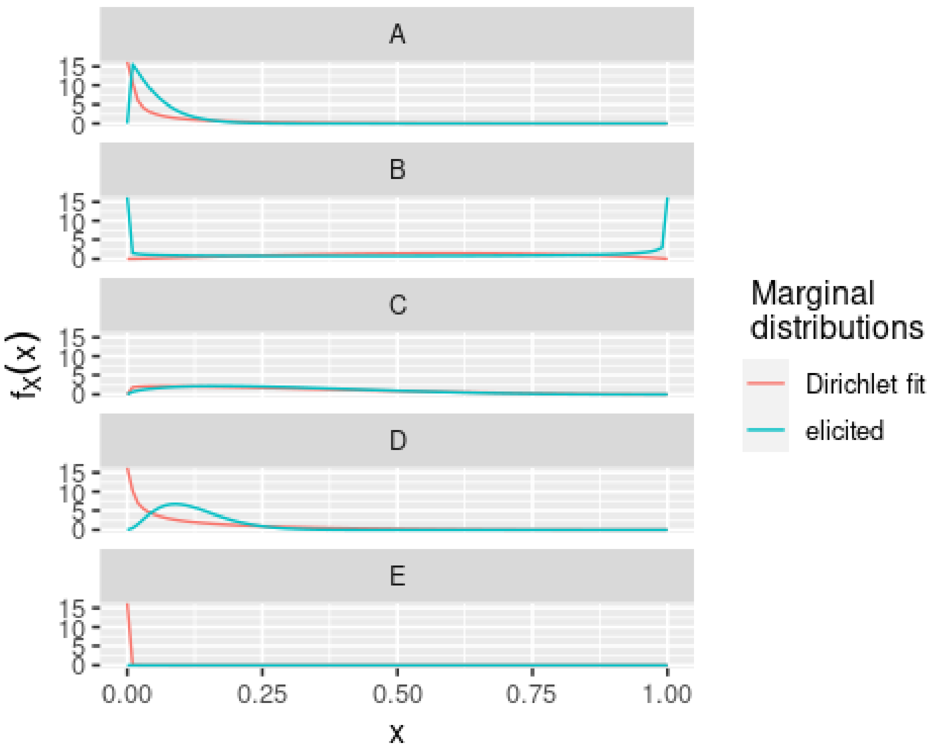


```
##
##      Directly      elicted      beta      marginal      distributions:
##
##                          A        B         C            D          E
##      shape1           1.0900    0.754     1.600       3.0200   1.80e+00
##       shape2         20.8000    0.607      3.910     21.8000   7.82e+03
##     mean                   0.0497    0.554     0.290     0.1220   2.30e-04
##    sd                          0.0454     0.324     0.178     0.0643   1.72e-04
##     sum                      21.9000      1.360      5.510    24.9000   7.82e+03
##
##       Sum       of       elicited       marginal       means:        1.015
##
##      Beta      marginal      distributions      from      Dirichlet      fit:
##
##                          A          B          C          D           E
##        shape1       0.1950      2.170      1.130      0.476     0.000901
##        shape2       3.7800      1.810      2.840      3.500     3.970000
##      mean                 0.0490     0.545      0.285      0.120     0.000227
##     sd                          0.0968      0.223      0.203      0.146     0.006760
## sum    3.9700 3.970 3.970 3.970 3.970000
```

Carcharhinus plumbeus

15 December 2021, 14:57

Elicited judgements

quantiles	A	B	C	D	E
0.25	0.2043	0.0700	0.1483	0.1503	1e-04
0.50	0.2749	0.2720	0.2827	0.2675	2e-04
0.75	0.3577	0.5553	0.4307	0.3980	3e-04

Dirichlet density function and parameters

Define the vector of unknown population proportions as

$$\theta := (\theta_1, \dots, \theta_k),$$

with $= 5$. We write

$$\theta \sim Dirichlet(a_1, \dots, a_k),$$

with

$$f(\theta) = \frac{\Gamma(a_1 + \dots + a_k)}{\Gamma(a_1) \dots \Gamma(a_k)} \prod_{i=1}^{k} \theta_i^{a_i - 1}.$$

The fitted parameter values $a_1, \dots, a_k$ are as follows:

```
##            A            B            C            D            E
## 1.0894628154 1.2933013878 1.1713603145 1.1035317235 0.0008803497
```

Comparing the elicited marginals with the marginals from the Dirichlet fit

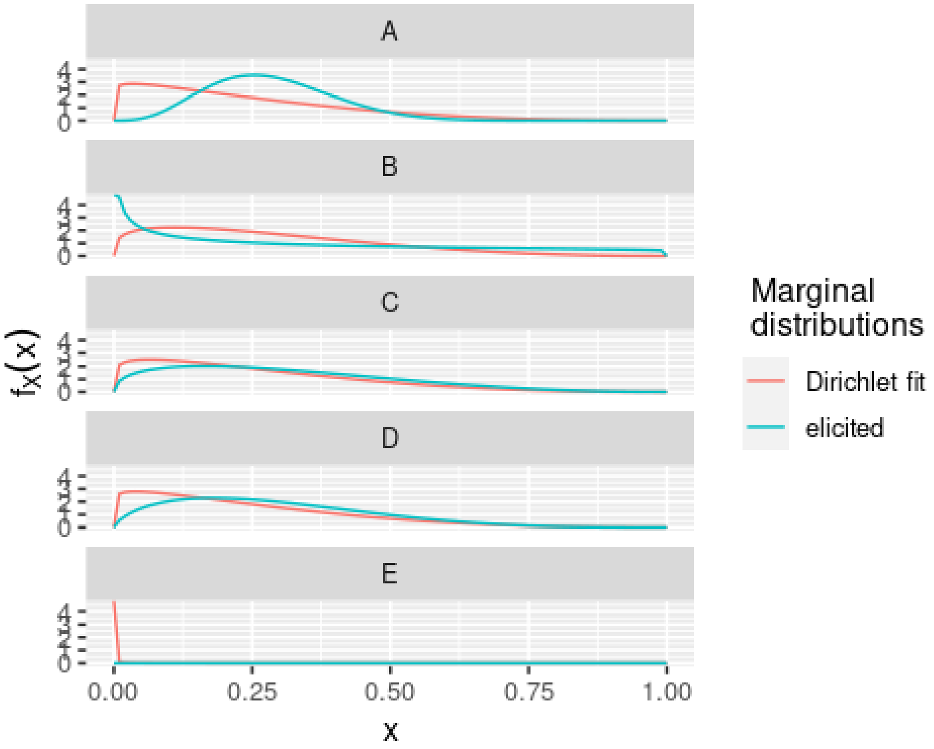

```
##
##      Directly      elicted      beta      marginal      distributions:
##
##                          A          B          C          D          E
##      shape1          4.480      0.544      1.450      1.710      1.80e+00
##       shape2      11.200      1.060      3.270      4.200      7.82e+03
##     mean                    0.285      0.338      0.307      0.289      2.30e-04
##     sd                          0.110      0.293      0.193      0.172      1.72e-04
##     sum                  15.700      1.610      4.720      5.900      7.82e+03
##
##      Sum      of      elicited      marginal      means:      1.219
##
##      Beta      marginal      distributions      from      Dirichlet      fit:
##
##                          A          B          C          D          E
##       shape1      1.090      1.290      1.170      1.100      0.000880
##       shape2      3.570      3.370      3.490      3.560      4.660000
##      mean                0.234      0.278      0.251      0.237      0.000189
##     sd                          0.178      0.188      0.182      0.179      0.005780
## sum    4.660 4.660 4.660 4.660 4.660000
```

Leucoraja melitensis

15 December 2021, 15:02

Elicited judgements

quantiles	A	B	C	D	E
0.25	0.4827	1e-04	0.0853	0.1832	1e-04
0.50	0.6000	2e-04	0.1847	0.2560	2e-04
0.75	0.7090	3e-04	0.2842	0.3403	3e-04

Dirichlet density function and parameters

Define the vector of unknown population proportions as

$$\theta := (\theta_1, \ldots, \theta_k),$$

with = 5 . We write

$$\theta \sim Dirichlet(a_1, \ldots, a_k),$$

with

$$f(\theta) = \frac{\Gamma(a_1 + \cdots + a_k)}{\Gamma(a_1) \ldots \Gamma(a_k)} \prod_{i=1}^{k} \theta_i^{a_i - 1}.$$

The fitted parameter values $a_1, \ldots, a_k$ are as follows:

```
##                    A                 B                 C                 D                 E
## 5.16931790 0.00201056 1.80301747 2.33271580 0.00201056
```

Comparing the elicited marginals with the marginals from the Dirichlet fit

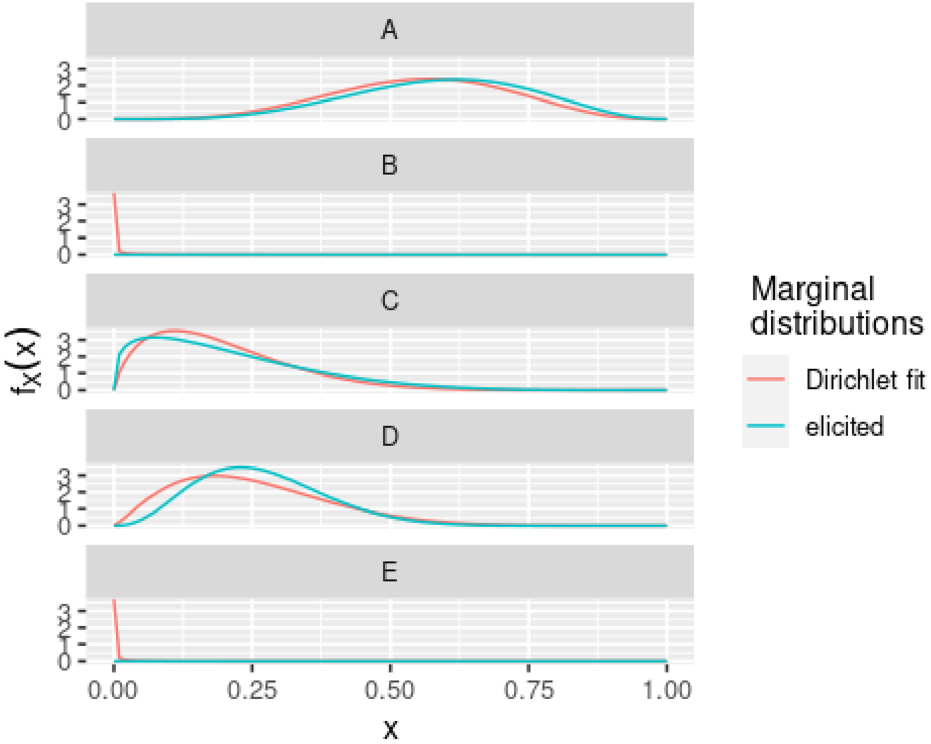


```
##
##     Directly    elicted    beta    marginal    distributions:
##
##                    A            B        C           D           E
##     shape1     5.200     1.80e+00    1.330       3.820    1.80e+00
##      shape2     3.580     7.82e+03    5.110      10.500    7.82e+03
##    mean              0.592    2.30e-04    0.207       0.267    2.30e-04
##    sd                   0.157    1.72e-04    0.148       0.113    1.72e-04
##    sum                  8.780    7.82e+03    6.440      14.300    7.82e+03
##
##     Sum    of    elicited    marginal    means:    1.067
##
##     Beta    marginal    distributions    from    Dirichlet    fit:
##
##                    A            B         C          D           E
##      shape1     5.170     0.002010     1.800      2.330     0.002010
##      shape2     4.140     9.310000     7.510      6.980     9.310000
##     mean              0.555     0.000216     0.194      0.251     0.000216
##    sd                   0.155     0.004580     0.123      0.135     0.004580
## sum    9.310 9.310000 9.310 9.310 9.310000
```

Raja polystigma

15 December 2021, 15:06

Elicited judgements

quantiles	A	B	C	D	E
0.25	0.3313	1e-04	0.0693	0.3107	1e-04
0.50	0.4150	2e-04	0.1667	0.4943	2e-04
0.75	0.5000	3e-04	0.2640	0.6637	3e-04

Dirichlet density function and parameters

Define the vector of unknown population proportions as

$$\theta := (\theta_1, \dots, \theta_k),$$

with $= 5$. We write

$$\theta \sim Dirichlet(a_1, \dots, a_k),$$

with

$$f(\theta) = \frac{\Gamma(a_1 + \cdots + a_k)}{\Gamma(a_1) \dots \Gamma(a_k)} \prod_{i=1}^{k} \theta_i^{a_i - 1}.$$

The fitted parameter values $a_1, \dots, a_k$ are as follows:

```
##           A           B           C           D           E
## 2.608334205 0.001437884 1.192853913 3.065655816 0.001437884
```

Comparing the elicited marginals with the marginals from the Dirichlet fit

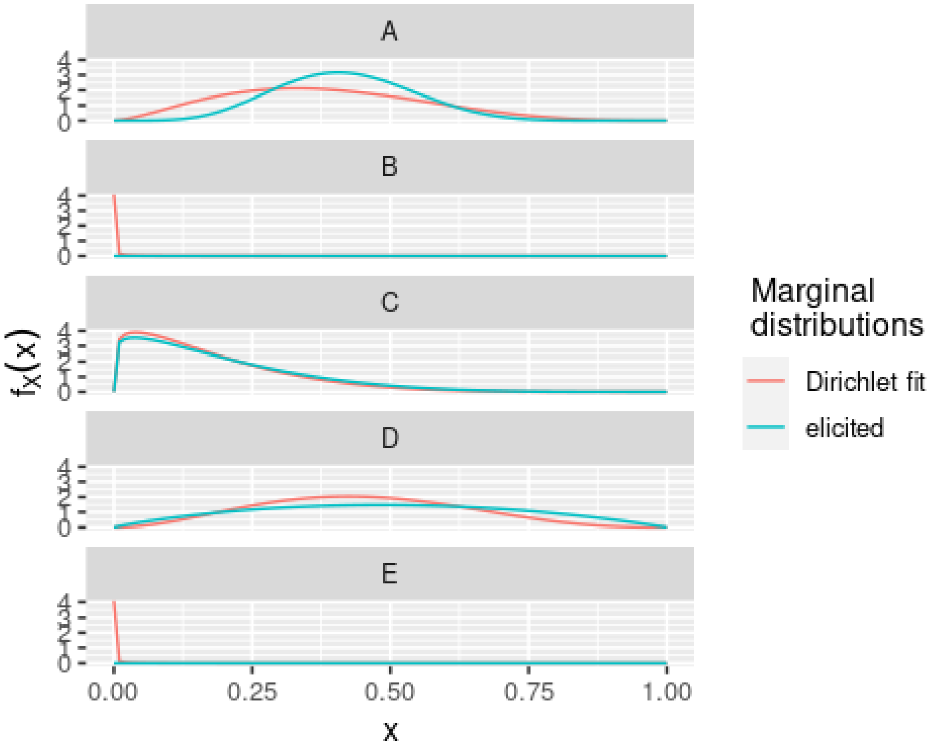

```
##
##        Directly        elicted        beta        marginal        distributions:
##
##                         A                 B           C          D              E
##       shape1            6.590      1.80e+00       1.150      1.900       1.80e+00
##       shape2            9.180      7.82e+03       4.860      1.970       7.82e+03
##      mean                   0.418      2.30e-04      0.191      0.491      2.30e-04
##     sd                          0.120      1.72e-04      0.149      0.227      1.72e-04
##      sum                   15.800      7.82e+03      6.010      3.870      7.82e+03
##
##        Sum        of        elicited        marginal        means:        1.101
##
##        Beta        marginal        distributions        from        Dirichlet        fit:
##
##                        A                   B           C           D             E
##        shape1       2.610         0.001440       1.190       3.070       0.001440
##        shape2       4.260         6.870000       5.680       3.800       6.870000
##      mean                   0.380      0.000209      0.174      0.446      0.000209
##      sd                         0.173      0.005160      0.135      0.177      0.005160
## sum    6.870 6.870000 6.870 6.870 6.870000
```

Rhinoptera marginata

15 December 2021, 15:11

Elicited judgements

quantiles	A	B	C	D	E
0.25	0.2580	1e-04	0.0920	0.2110	0.0987
0.50	0.3303	2e-04	0.1730	0.3080	0.1833
0.75	0.4080	3e-04	0.3287	0.3957	0.2983

Dirichlet density function and parameters

Define the vector of unknown population proportions as

$$\theta := (\theta_1, \ldots, \theta_k),$$

with $= 5$. We write

$$\theta \sim Dirichlet(a_1, \ldots, a_k),$$

with

$$f(\theta) = \frac{\Gamma(a_1 + \cdots + a_k)}{\Gamma(a_1) \ldots \Gamma(a_k)} \prod_{i=1}^{k} \theta_i^{a_i - 1}.$$

The fitted parameter values $a_1, \ldots, a_k$ are as follows:

```
##           A           B           C           D           E
## 2.929481892 0.002006962 1.920418067 2.731264394 1.845076064
```

Comparing the elicited marginals with the marginals from the Dirichlet fit

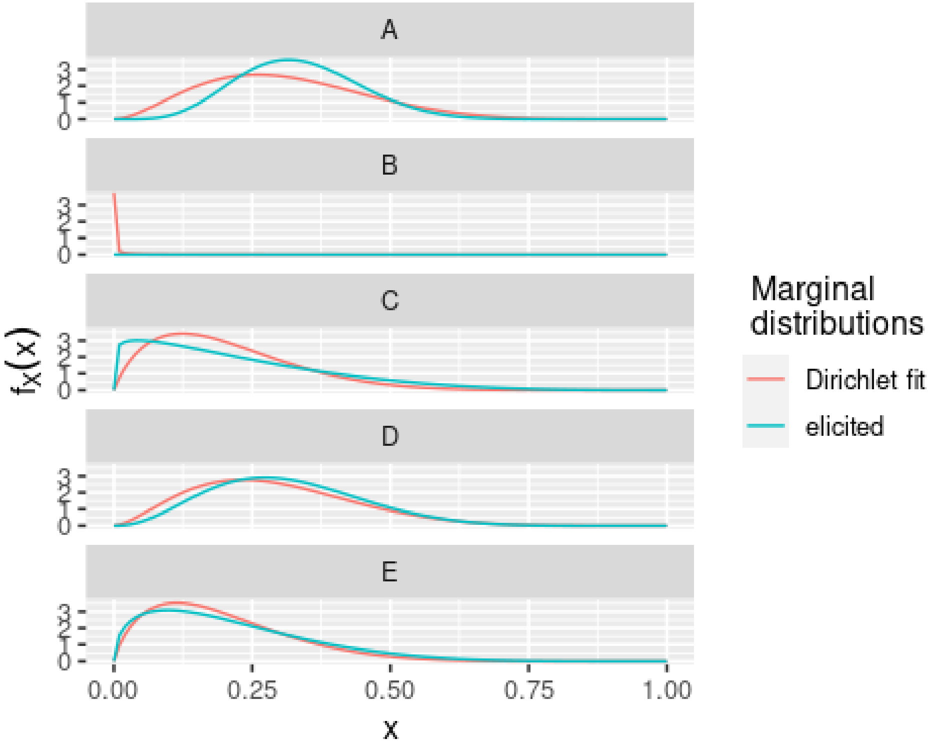


```
##
##       Directly        elicted        beta        marginal        distributions:
##
##                                 A                    B          C              D           E
##      shape1                6.100        1.80e+00       1.130                3.540       1.490
##       shape2        12.000        7.82e+03       4.010                    7.740       5.540
##     mean                             0.336      2.30e-04     0.220           0.314       0.212
##     sd                                   0.108      1.72e-04     0.167           0.132       0.144
##      sum                               18.100       7.82e+03      5.140       11.300       7.020
##
##       Sum          of          elicited          marginal          means:          1.082
##
##      Beta        marginal        distributions         from        Dirichlet         fit:
##
##                                  A                    B             C           D            E
##        shape1          2.930           0.002010          1.920        2.73         1.850
##        shape2          6.500           9.430000          7.510        6.70         7.580
##       mean                           0.311        0.000213        0.204        0.29        0.196
##      sd                                    0.143        0.004520       0.125        0.14        0.123
## sum     9.430 9.430000 9.430 9.43 9.430
```

Raja asterias

15 December 2021, 15:15

Elicited judgements

quantiles	A	B	C	D	E
0.25	0.4410	1e-04	0.0159	0.2763	1e-04
0.50	0.5337	2e-04	0.0180	0.4537	2e-04
0.75	0.6137	3e-04	0.0202	0.5695	3e-04

Dirichlet density function and parameters

Define the vector of unknown population proportions as

$$\theta := (\theta_1, \dots, \theta_k),$$

with $= 5$. We write

$$\theta \sim Dirichlet(a_1, \dots, a_k),$$

with

$$f(\theta) = \frac{\Gamma(a_1 + \dots + a_k)}{\Gamma(a_1) \dots \Gamma(a_k)} \prod_{i=1}^{k} \theta_i^{a_i - 1}.$$

The fitted parameter values $a_1, \dots, a_k$ are as follows:

```
##           A           B           C           D           E
## 4.923290220 0.002143688 0.169012931 4.105534888 0.002143688
```

Comparing the elicited marginals with the marginals from the Dirichlet fit

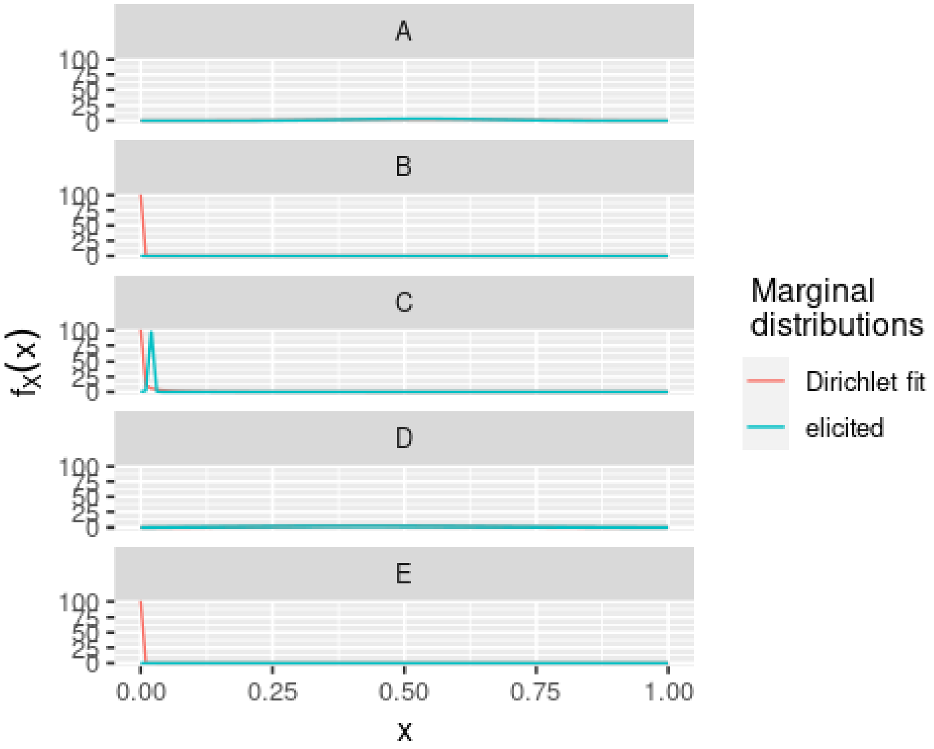

```
##
##        Directly        elicted        beta        marginal        distributions:
##
##                        A              B               C          D              E
##      shape1            8.160      1.80e+00     3.15e+01      2.290      1.80e+00
##      shape2            7.260      7.82e+03     1.70e+03      2.910      7.82e+03
##     mean                   0.529      2.30e-04     1.82e-02     0.441      2.30e-04
##    sd                         0.123      1.72e-04     3.21e-03     0.199      1.72e-04
##     sum                    15.400      7.82e+03     1.73e+03     5.200      7.82e+03
##
##        Sum        of        elicited        marginal        means:        0.989
##
##       Beta       marginal       distributions        from       Dirichlet       fit:
##
##                        A                B              C          D              E
##         shape1      4.920         0.002140       0.1690      4.110       0.002140
##         shape2      4.280         9.200000       9.0300      5.100       9.200000
##       mean                 0.535      0.000233       0.0184      0.446      0.000233
##      sd                          0.156      0.004780      0.0420      0.156      0.004780
## sum    9.200 9.200000 9.2000 9.200 9.200000
```

Raja radula

15 December 2021, 15:19

Elicited judgements

quantiles	A	B	C	D	E
0.25	0.2300	1e-04	0.0693	0.3623	1e-04
0.50	0.3110	2e-04	0.1667	0.5473	2e-04
0.75	0.3993	3e-04	0.2640	0.6980	3e-04

Dirichlet density function and parameters

Define the vector of unknown population proportions as

$$\theta := (\theta_1, \dots, \theta_k),$$

with $= 5$. We write

$$\theta \sim Dirichlet(a_1, \dots, a_k),$$

with

$$f(\theta) = \frac{\Gamma(a_1 + \cdots + a_k)}{\Gamma(a_1) \dots \Gamma(a_k)} \prod_{i=1}^{k} \theta_i^{a_i - 1}.$$

The fitted parameter values $a_1, \dots, a_k$ are as follows:

```
##           A           B           C           D           E
## 2.055177234 0.001481847 1.229324876 3.430493764 0.001481847
```

Comparing the elicited marginals with the marginals from the Dirichlet fit

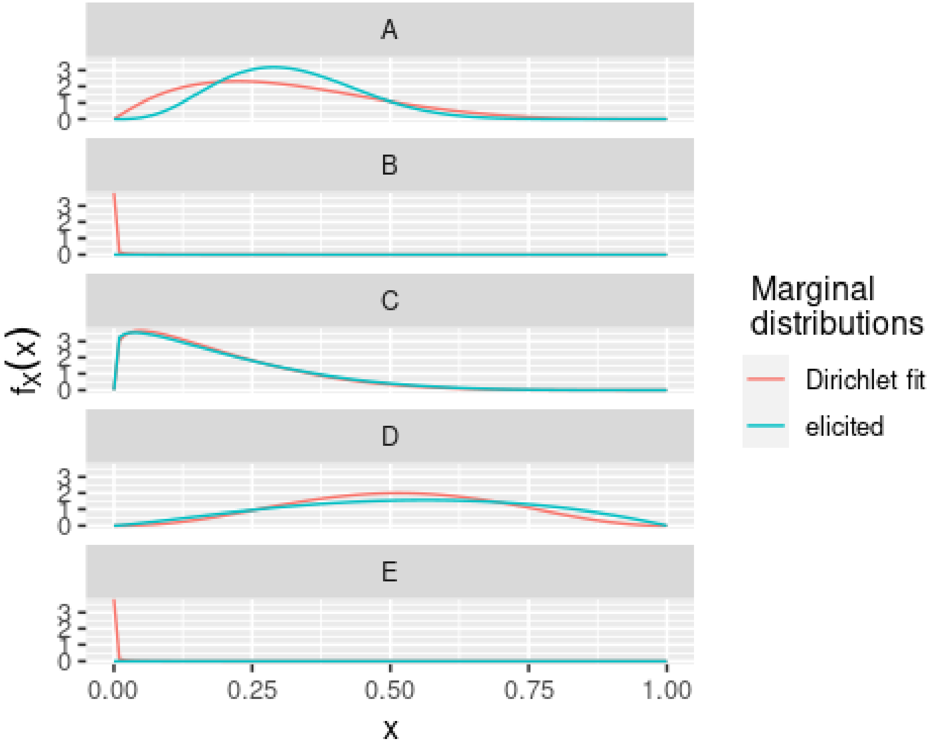


```
##
##          Directly          elicted          beta          marginal          distributions:
##
##                              A                    B            C            D                E
##        shape1               4.420         1.80e+00        1.150        2.260         1.80e+00
##        shape2               9.410         7.82e+03        4.860        1.980         7.82e+03
##      mean                        0.320        2.30e-04        0.191        0.533        2.30e-04
##     sd                               0.121        1.72e-04        0.149        0.218        1.72e-04
##      sum                        13.800        7.82e+03        6.010        4.230        7.82e+03
##
##          Sum          of          elicited          marginal          means:          1.045
##
##        Beta          marginal          distributions          from          Dirichlet          fit:
##
##                            A                         B            C            D                E
##          shape1          2.060          0.001480          1.230          3.430          0.001480
##          shape2          4.660          6.720000          5.490          3.290          6.720000
##        mean                    0.306          0.000221          0.183          0.511          0.000221
##       sd                            0.166          0.005350          0.139          0.180          0.005350
## sum     6.720 6.720000 6.720 6.720 6.720000
```

Gymnura altavela

15 December 2021, 15:24

Elicited judgements

quantiles	A	B	C	D	E
0.25	0.2377	1e-04	0.0553	0.1480	0.1333
0.50	0.3560	2e-04	0.1177	0.2023	0.2620
0.75	0.4713	3e-04	0.2500	0.2630	0.3697

Dirichlet density function and parameters

Define the vector of unknown population proportions as

$$\theta := (\theta_1, \ldots, \theta_k),$$

with $= 5$. We write

$$\theta \sim Dirichlet(a_1, \ldots, a_k),$$

with

$$f(\theta) = \frac{\Gamma(a_1 + \cdots + a_k)}{\Gamma(a_1) \ldots \Gamma(a_k)} \prod_{i=1}^{k} \theta_i^{a_i - 1}.$$

The fitted parameter values $a_1, \ldots, a_k$ are as follows:

```
##           A           B           C           D           E
## 2.941160307 0.001862593 1.339717507 1.701854791 2.219492254
```

Comparing the elicited marginals with the marginals from the Dirichlet fit

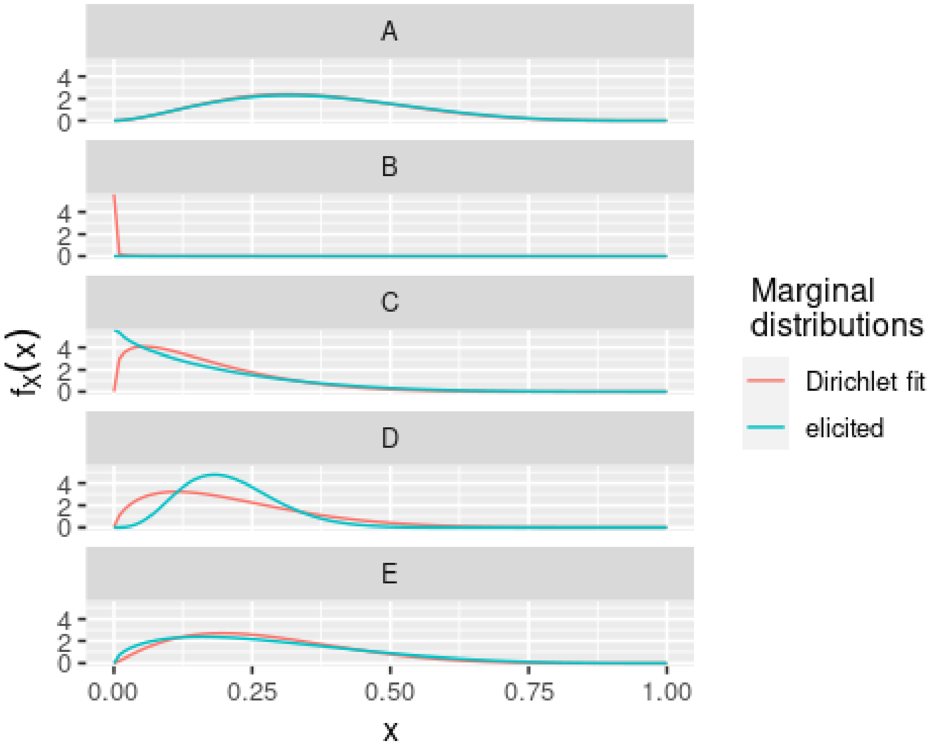

```
##
##        Directly        elicted        beta        marginal        distributions:
##
##                          A                     B         C            D         E
##       shape1       2.830      1.80e+00      0.920          4.7000     1.620
##        shape2        4.950       7.82e+03        4.630      17.6000     4.280
##      mean                  0.364     2.30e-04     0.166         0.2110     0.275
##     sd                        0.162     1.72e-04     0.145         0.0844     0.170
##      sum                       7.780      7.82e+03      5.550      22.3000     5.900
##
##        Sum        of        elicited        marginal        means:        1.015
##
##       Beta       marginal       distributions       from       Dirichlet       fit:
##
##                           A                     B         C          D         E
##        shape1        2.940         0.001860         1.340       1.700        2.220
##        shape2        5.260         8.200000         6.860       6.500        5.980
##       mean                    0.358      0.000227        0.163       0.207       0.271
##      sd                          0.158       0.004970       0.122       0.134       0.146
## sum    8.200 8.200000 8.200 8.200 8.200
```

Raja miralaetus

15 December 2021, 15:30

Elicited judgements

quantiles	A	B	C	D	E
0.25	0.5087	1e-04	0.0159	0.2936	1e-04
0.50	0.6647	2e-04	0.0180	0.4533	2e-04
0.75	0.7967	3e-04	0.0202	0.5815	3e-04

Dirichlet density function and parameters

Define the vector of unknown population proportions as

$$\theta := (\theta_1, \ldots, \theta_k),$$

with = 5 . We write

$$\theta \sim Dirichlet(a_1, \ldots, a_k),$$

with

$$f(\theta) = \frac{\Gamma(a_1 + \cdots + a_k)}{\Gamma(a_1) \ldots \Gamma(a_k)} \prod_{i=1}^{k} \theta_i^{a_i - 1}.$$

The fitted parameter values $a_1, \ldots, a_k$ are as follows:

```
##              A              B              C              D              E
## 3.368625837 0.001205292 0.095027816 2.347398593 0.001205292
```

Comparing the elicited marginals with the marginals from the Dirichlet fit

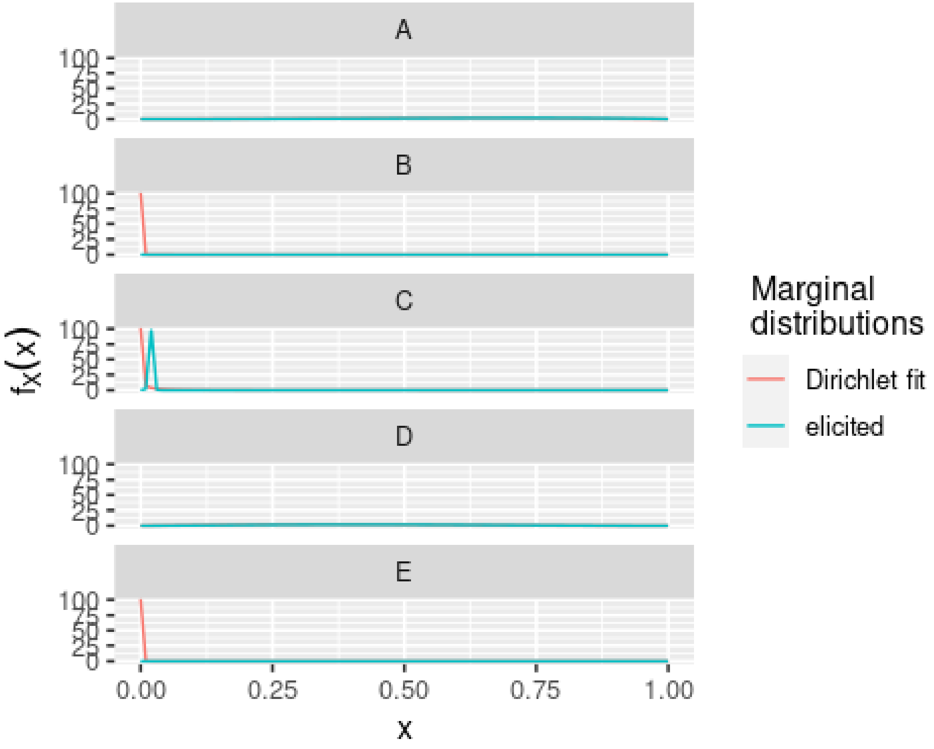


```
##
##       Directly        elicted        beta        marginal        distributions:
##
##                          A                 B                  C           D              E
##       shape1         3.300         1.80e+00         3.15e+01       2.500         1.80e+00
##       shape2         1.820         7.82e+03         1.70e+03       3.070         7.82e+03
##      mean                    0.644       2.30e-04       1.82e-02       0.449       2.30e-04
##     sd                          0.194       1.72e-04       3.21e-03       0.194       1.72e-04
##     sum                       5.120       7.82e+03       1.73e+03       5.570       7.82e+03
##
##       Sum         of         elicited         marginal         means:          1.111
##
##      Beta        marginal        distributions        from        Dirichlet        fit:
##
##                             A                    B               C           D              E
##       shape1         3.370         0.001210         0.0950         2.350         0.001210
##       shape2         2.440         5.810000         5.7200         3.470         5.810000
##      mean                    0.579       0.000207       0.0163       0.404       0.000207
##     sd                          0.189       0.005520       0.0486       0.188       0.005520
## sum     5.810 5.810000 5.8100 5.810 5.810000
```

Mobula mobular

15 December 2021, 15:36

Elicited judgements

quantiles	A	B	C	D	E
0.25	0.1910	0.0637	0.1637	0.0630	0.1085
0.50	0.2613	0.0747	0.1977	0.1042	0.2545
0.75	0.3437	0.0890	0.2337	0.1573	0.4007

Dirichlet density function and parameters

Define the vector of unknown population proportions as

$$\theta := (\theta_1, \ldots, \theta_k),$$

with $= 5$. We write

$$\theta \sim Dirichlet(a_1, \ldots, a_k),$$

with

$$f(\theta) = \frac{\Gamma(a_1 + \cdots + a_k)}{\Gamma(a_1) \ldots \Gamma(a_k)} \prod_{i=1}^{k} \theta_i^{a_i - 1}.$$

The fitted parameter values $a_1, \ldots, a_k$ are as follows:

```
##        A        B        C        D        E
## 4.045096 1.140008 2.984968 1.743961 4.181793
```

Comparing the elicited marginals with the marginals from the Dirichlet fit

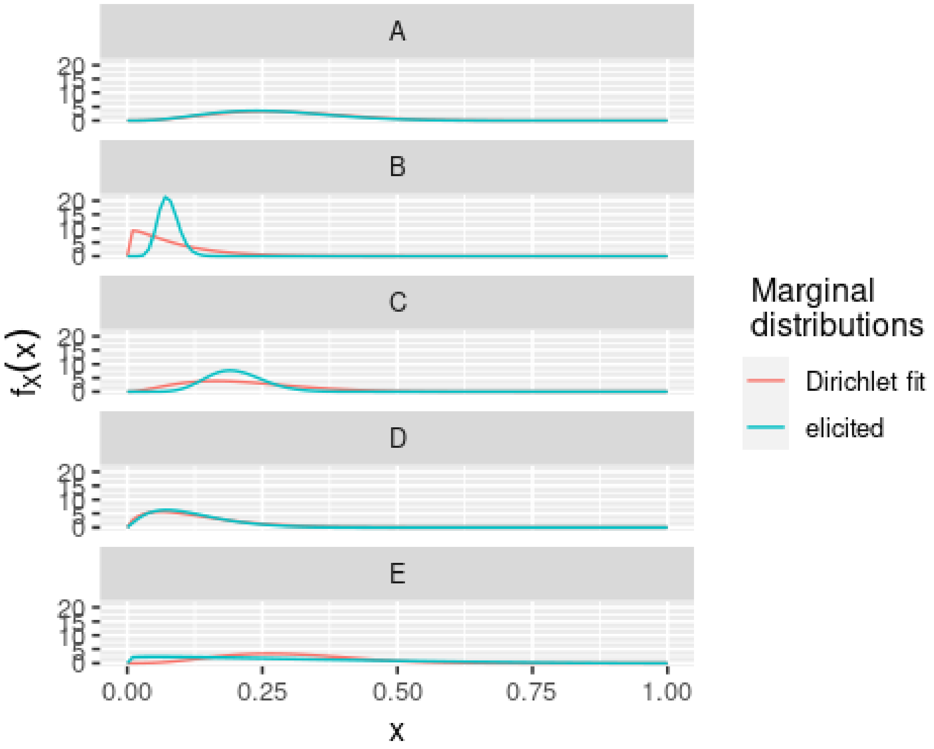

```
##
##         Directly         elicted         beta         marginal         distributions:
##
##                            A                B            C              D          E
##      shape1            4.170          15.2000      11.8000        2.2400      1.100
##        shape2         11.200         183.0000      47.1000      16.8000       2.800
##     mean                  0.272           0.0767        0.2010        0.1170     0.281
##     sd                       0.110           0.0188        0.0517        0.0718   0.203
##      sum                        15.300      198.0000      58.9000      19.1000    3.900
##
##         Sum         of         elicited         marginal         means:         0.948
##
##        Beta        marginal        distributions        from        Dirichlet        fit:
##
##                             A              B             C               D               E
##     shape1           4.050          1.1400          2.980          1.7400             4.180
##       shape2        10.100        13.0000        11.100        12.4000             9.910
##     mean                0.287         0.0809         0.212          0.1240           0.297
##     sd                     0.116          0.0702         0.105          0.0847          0.118
## sum    14.100 14.1000 14.100 14.1000 14.100
```

Aetomylaeus bovinus

15 December 2021, 15:42

Elicited judgements

quantiles	A	B	C	D	E
0.25	0.1102	0.0490	0.0553	0.2850	0.1085
0.50	0.1640	0.0517	0.1177	0.4070	0.2243
0.75	0.2293	0.0547	0.2500	0.4483	0.3303

Dirichlet density function and parameters

Define the vector of unknown population proportions as

$$\theta := (\theta_1, \ldots, \theta_k),$$

with $= 5$. We write

$$\theta \sim Dirichlet(a_1, \ldots, a_k),$$

with

$$f(\theta) = \frac{\Gamma(a_1 + \cdots + a_k)}{\Gamma(a_1) \ldots \Gamma(a_k)} \prod_{i=1}^{k} \theta_i^{a_i - 1}.$$

The fitted parameter values $a_1, \ldots, a_k$ are as follows:

```
##                     A                  B                  C                  D                  E
## 1.8468874 0.5435636 1.7368714 4.0576645 2.5387926
```

Comparing the elicited marginals with the marginals from the Dirichlet fit

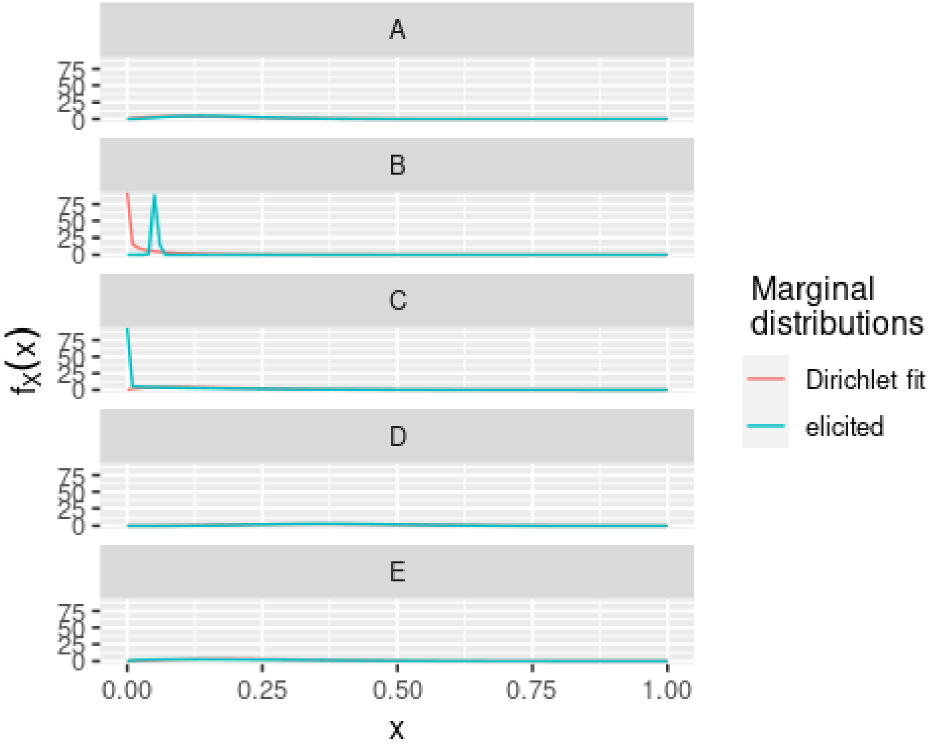


```
##
##    Directly    elicted    beta    marginal    distributions:
##
##                      A            B        C          D         E
##    shape1          3.130   1.43e+02    0.920      5.240     1.460
##     shape2     14.600     2.61e+03    4.630      8.290     4.560
##    mean                0.176    5.19e-02    0.166    0.387     0.242
##    sd                    0.088    4.23e-03    0.145    0.128    0.162
##     sum                17.700     2.75e+03    5.550   13.500     6.010
##
##    Sum    of    elicited    marginal    means:    1.023
##
##    Beta    marginal    distributions    from    Dirichlet    fit:
##
##                      A              B          C          D         E
##    shape1       1.850       0.5440       1.740      4.060     2.540
##     shape2       8.880    10.2000      8.990      6.670     8.180
##    mean              0.172       0.0507     0.162     0.378     0.237
##    sd                  0.110      0.0641     0.108     0.142     0.124
## sum    10.700 10.7000 10.700 10.700 10.700
```

Myliobatis aquila

15 December 2021, 15:46

Elicited judgements

quantiles	A	B	C	D	E
0.25	0.2115	0.0490	0.0553	0.2850	0.0844
0.50	0.2680	0.0517	0.1177	0.4070	0.1597
0.75	0.3300	0.0547	0.2500	0.4483	0.2847

Dirichlet density function and parameters

Define the vector of unknown population proportions as

$$\theta := (\theta_1, \dots, \theta_k),$$

with $= 5$. We write

$$\theta \sim Dirichlet(a_1, \dots, a_k),$$

with

$$f(\theta) = \frac{\Gamma(a_1 + \cdots + a_k)}{\Gamma(a_1) \dots \Gamma(a_k)} \prod_{i=1}^{k} \theta_i^{a_i - 1}.$$

The fitted parameter values $a_1, \dots, a_k$ are as follows:

```
##                 A                B                C                D                E
## 3.1619932 0.5986895 1.9130174 4.4691754 2.2662607
```

Comparing the elicited marginals with the marginals from the Dirichlet fit

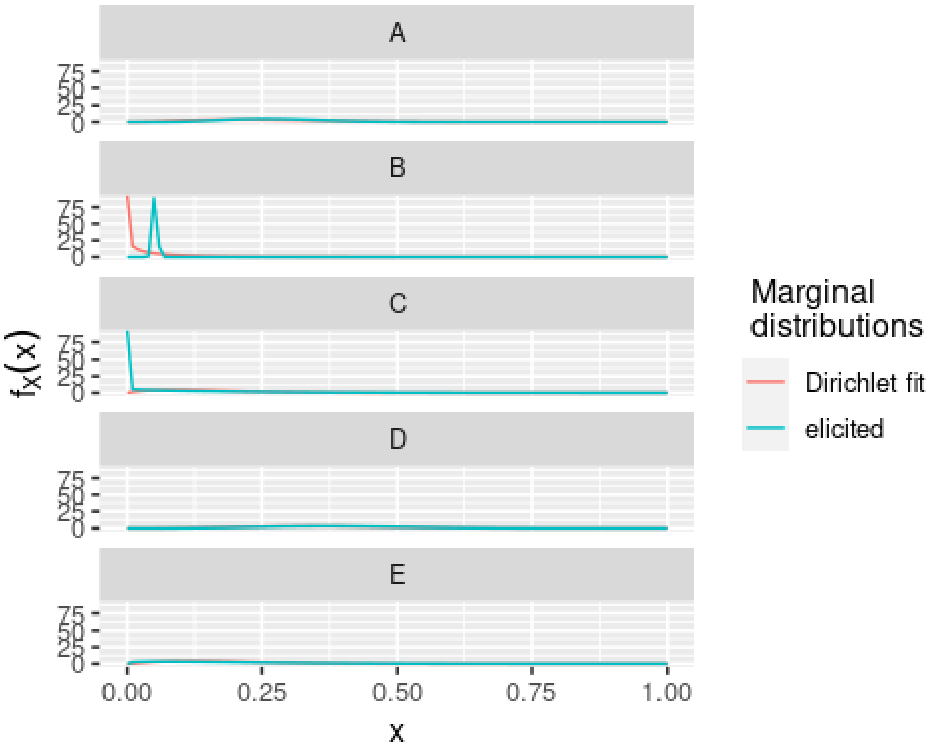

```
##
##        Directly         elicted         beta         marginal         distributions:
##
##                              A                 B            C                D           E
##       shape1           7.0300       1.43e+02       0.920             5.240       1.270
##         shape2       18.6000          2.61e+03       4.630             8.290       5.210
##      mean                        0.2740      5.19e-02     0.166           0.387       0.196
##     sd                               0.0864      4.23e-03     0.145           0.128       0.145
##      sum                           25.7000      2.75e+03       5.550      13.500       6.490
##
##        Sum         of          elicited         marginal         means:            1.075
##
##        Beta        marginal        distributions         from        Dirichlet          fit:
##
##                         A                  B                 C             D            E
##     shape1         3.160          0.5990          1.9100         4.470           2.270
##       shape2              9.250       11.8000       10.5000           7.940       10.100
##     mean                   0.255          0.0482          0.1540        0.360         0.183
##    sd                           0.119          0.0585          0.0986        0.131         0.106
## sum    12.400 12.4000 12.4000 12.400 12.400
```

Raja brachyura

15 December 2021, 15:53

Elicited judgements

quantiles	A	B	C	D	E
0.25	0.2977	1e-04	0.0693	0.3467	1e-04
0.50	0.3850	2e-04	0.1667	0.5293	2e-04
0.75	0.4750	3e-04	0.2640	0.6833	3e-04

Dirichlet density function and parameters

Define the vector of unknown population proportions as

$$\theta := (\theta_1, \dots, \theta_k),$$

with $= 5$. We write

$$\theta \sim Dirichlet(a_1, \dots, a_k),$$

with

$$f(\theta) = \frac{\Gamma(a_1 + \cdots + a_k)}{\Gamma(a_1) \dots \Gamma(a_k)} \prod_{i=1}^{k} \theta_i^{a_i - 1}.$$

The fitted parameter values $a_1, \dots, a_k$ are as follows:

```
##                A                B                C                D                E
## 2.431782982 0.001437751 1.192743274 3.237063440 0.001437751
```

Comparing the elicited marginals with the marginals from the Dirichlet fit

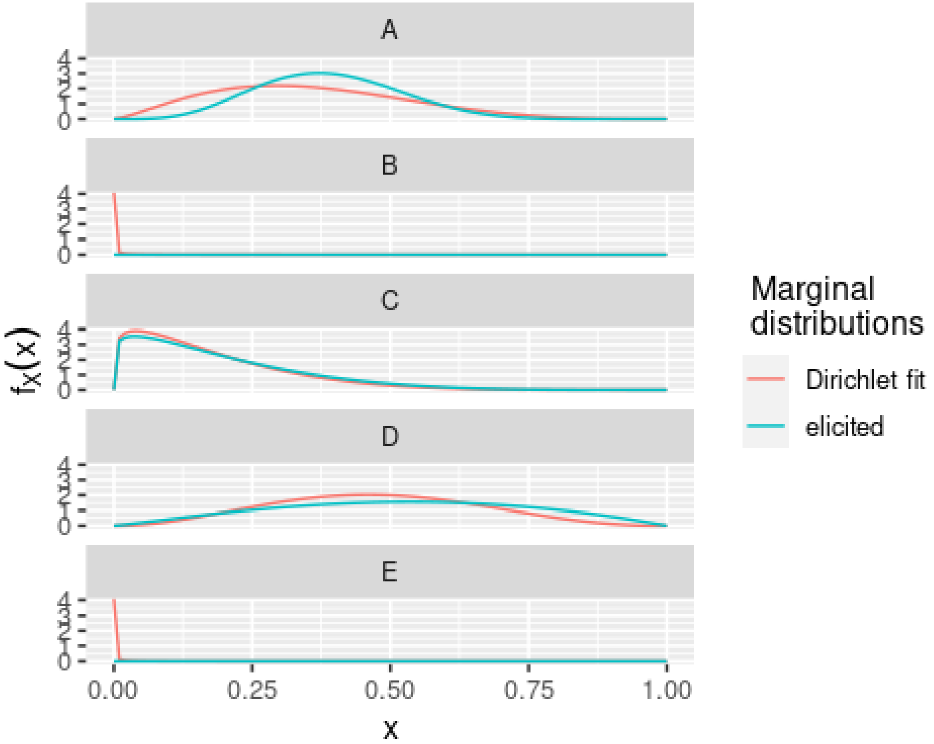


```
##
##          Directly          elicted          beta          marginal          distributions:
##
##                            A                  B             C             D              E
##        shape1              5.430        1.80e+00        1.150         2.190        1.80e+00
##        shape2              8.510        7.82e+03        4.860         2.040        7.82e+03
##      mean                        0.390       2.30e-04       0.191        0.519       2.30e-04
##     sd                                0.126       1.72e-04       0.149        0.218       1.72e-04
##      sum                       13.900       7.82e+03       6.010        4.230       7.82e+03
##
##          Sum          of          elicited          marginal          means:          1.1
##
##        Beta          marginal          distributions          from          Dirichlet          fit:
##
##                            A                    B            C             D              E
##          shape1       2.430          0.001440        1.190         3.240        0.001440
##          shape2       4.430          6.860000        5.670         3.630        6.860000
##       mean                    0.354        0.000209        0.174        0.472       0.000209
##      sd                            0.171        0.005160        0.135        0.178       0.005160
## sum     6.860 6.860000 6.860 6.860 6.860000
```

S2.4.52 A

Raja montagui

16 December 2021, 10:04

Elicited judgements

quantiles	A	B	C	D	E
0.25	0.5330	1e-04	0.0159	0.2264	1e-04
0.50	0.5960	2e-04	0.0180	0.3253	2e-04
0.75	0.6483	3e-04	0.0202	0.4012	3e-04

Dirichlet density function and parameters

Define the vector of unknown population proportions as

$$\theta := (\theta_1, \ldots, \theta_k),$$

with $= 5$. We write

$$\theta \sim Dirichlet(a_1, \ldots, a_k),$$

with

$$f(\theta) = \frac{\Gamma(a_1 + \cdots + a_k)}{\Gamma(a_1) \ldots \Gamma(a_k)} \prod_{i=1}^{k} \theta_i^{a_i - 1}.$$

The fitted parameter values $a_1, \ldots, a_k$ are as follows:

```
##            A           B           C           D           E
## 13.574450145  0.005286618  0.416808267  7.470462621  0.005286618
```

Comparing the elicited marginals with the marginals from the Dirichlet fit

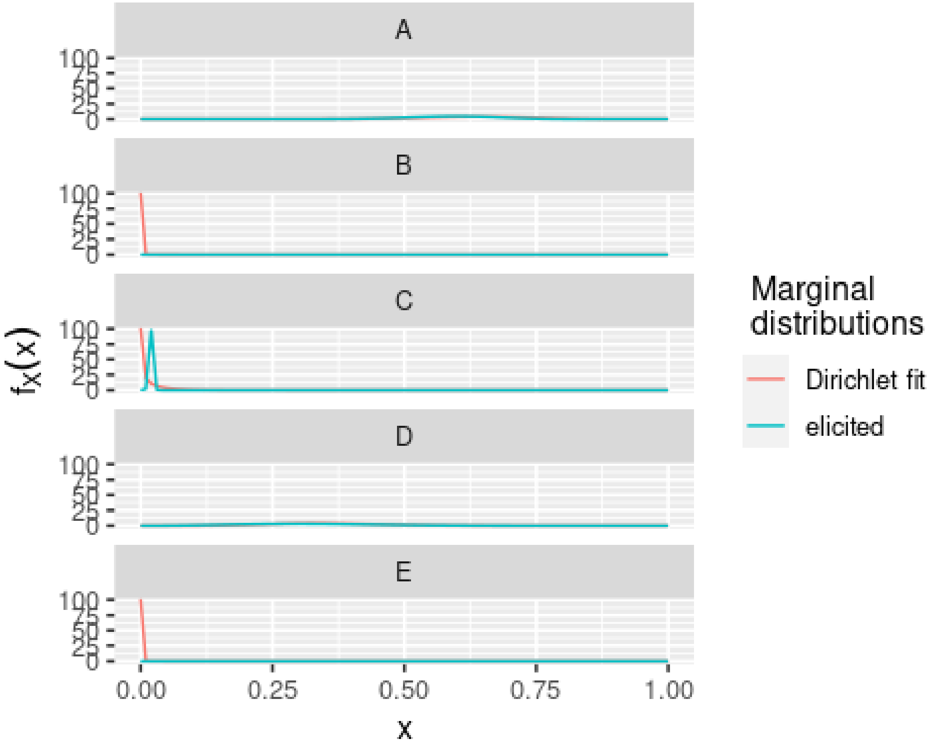


```
##
##      Directly    elicted     beta     marginal     distributions:
##
##                       A             B              C          D           E
##     shape1     19.700      1.80e+00      3.15e+01          4.120      1.80e+00
##     shape2     13.600      7.82e+03      1.70e+03          8.540      7.82e+03
##    mean                  0.592     2.30e-04     1.82e-02        0.326     2.30e-04
##    sd                       0.084     1.72e-04     3.21e-03        0.127     1.72e-04
##     sum                   33.200     7.82e+03     1.73e+03      12.700     7.82e+03
##
##      Sum       of       elicited       marginal       means:        0.936
##
##     Beta      marginal      distributions      from      Dirichlet      fit:
##
##                        A              B             C           D           E
##     shape1      13.600       5.29e-03          0.4170       7.470      5.29e-03
##      shape2            7.900      2.15e+01      21.1000     14.000      2.15e+01
##    mean                 0.632     2.46e-04      0.0194       0.348      2.46e-04
##    sd                       0.102     3.31e-03       0.0291        0.100     3.31e-03
## sum    21.500 2.15e+01 21.5000 21.500 2.15e+01
```

Leucoraja naevus

16 December 2021, 10:26

Elicited judgements

quantiles	A	B	C	D	E
0.25	0.6083	1e-04	0.0319	0.1301	1e-04
0.50	0.7347	2e-04	0.0360	0.1800	2e-04
0.75	0.8383	3e-04	0.0403	0.2385	3e-04

Dirichlet density function and parameters

Define the vector of unknown population proportions as

$$\theta := (\theta_1, \dots, \theta_k),$$

with $= 5$. We write

$$\theta \sim Dirichlet(a_1, \dots, a_k),$$

with

$$f(\theta) = \frac{\Gamma(a_1 + \cdots + a_k)}{\Gamma(a_1) \dots \Gamma(a_k)} \prod_{i=1}^{k} \theta_i^{a_i - 1}.$$

The fitted parameter values $a_1, \dots, a_k$ are as follows:

```
##           A           B           C           D           E
## 9.872657122 0.003190125 0.502783982 2.618066182 0.003190125
```

Comparing the elicited marginals with the marginals from the Dirichlet fit

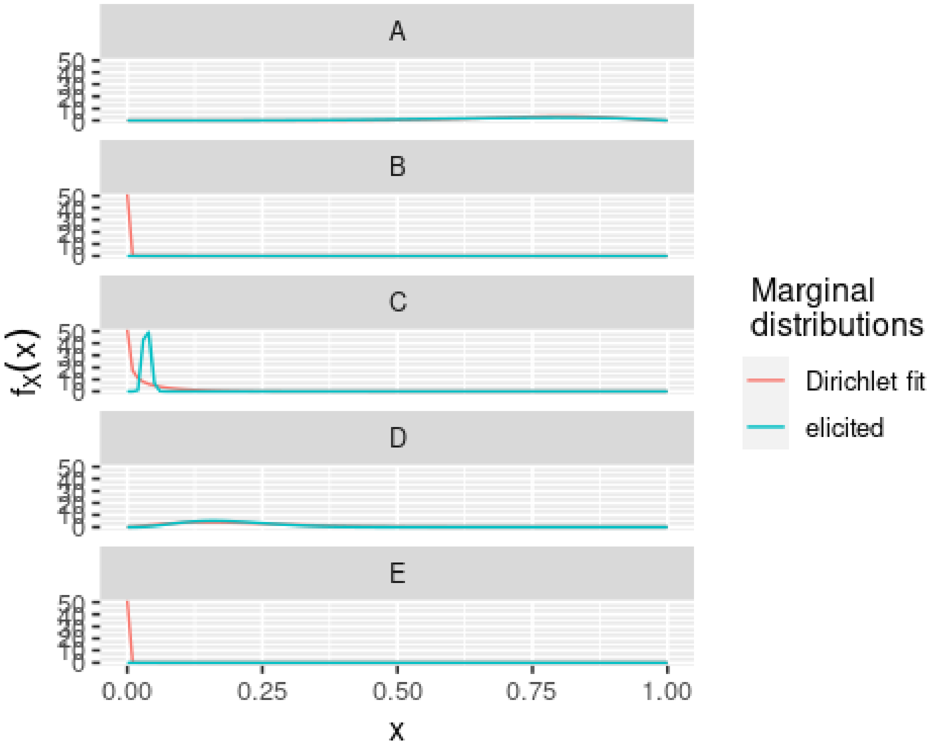

```
##
##        Directly        elicted        beta        marginal        distributions:
##
##                          A           B           C           D           E
##       shape1     4.940    1.80e+00    3.24e+01      4.3600    1.80e+00
##        shape2     1.990    7.82e+03    8.60e+02     18.7000    7.82e+03
##      mean         0.713    2.30e-04    3.63e-02      0.1890    2.30e-04
##     sd            0.161    1.72e-04    6.26e-03      0.0799    1.72e-04
##      sum          6.920    7.82e+03    8.93e+02     23.0000    7.82e+03
##
##        Sum        of        elicited        marginal        means:        0.939
##
##       Beta       marginal       distributions       from       Dirichlet       fit:
##
##                          A           B           C           D           E
##      shape1       9.870    3.19e-03      0.5030       2.620    3.19e-03
##       shape2      3.130    1.30e+01     12.5000      10.400    1.30e+01
##     mean          0.759    2.45e-04      0.0387       0.201    2.45e-04
##     sd            0.114    4.19e-03      0.0515       0.107    4.19e-03
## sum    13.000 1.30e+01 13.0000 13.000 1.30e+01
```

Raja undulata

16 December 2021, 10:29

Elicited judgements

quantiles	A	B	C	D	E
0.25	0.3557	1e-04	0.0159	0.3093	1e-04
0.50	0.4457	2e-04	0.0180	0.4713	2e-04
0.75	0.5287	3e-04	0.0202	0.5962	3e-04

Dirichlet density function and parameters

Define the vector of unknown population proportions as

$$\theta := (\theta_1, \ldots, \theta_k),$$

with $= 5$. We write

$$\theta \sim Dirichlet(a_1, \ldots, a_k),$$

with

$$f(\theta) = \frac{\Gamma(a_1 + \cdots + a_k)}{\Gamma(a_1) \ldots \Gamma(a_k)} \prod_{i=1}^{k} \theta_i^{a_i - 1}.$$

The fitted parameter values $a_1, \ldots, a_k$ are as follows:

```
##           A           B           C           D           E
## 4.581955136 0.002369705 0.186832592 4.769442383 0.002369705
```

Comparing the elicited marginals with the marginals from the Dirichlet fit

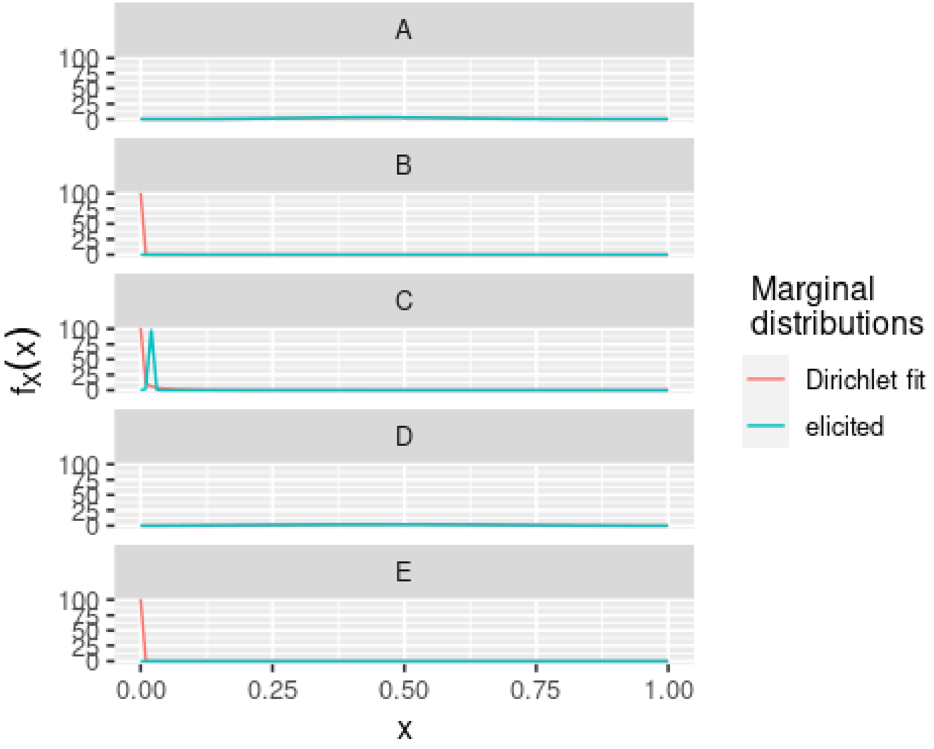


```
##
##        Directly         elicted         beta         marginal         distributions:
##
##                          A                    B                   C            D                E
##       shape1             6.780        1.80e+00        3.15e+01       2.610       1.80e+00
##       shape2             8.440        7.82e+03        1.70e+03       3.020       7.82e+03
##      mean                       0.446       2.30e-04       1.82e-02       0.464       2.30e-04
##     sd                              0.123       1.72e-04       3.21e-03       0.194       1.72e-04
##      sum                       15.200       7.82e+03       1.73e+03       5.640       7.82e+03
##
##        Sum         of         elicited         marginal         means:         0.928
##
##       Beta        marginal        distributions         from        Dirichlet         fit:
##
##                          A                     B                 C            D                E
##        shape1         4.580          0.002370          0.1870         4.770          0.002370
##        shape2         4.960          9.540000          9.3600         4.770          9.540000
##       mean                     0.480        0.000248        0.0196        0.500        0.000248
##      sd                              0.154        0.004850        0.0427        0.154        0.004850
## sum     9.540 9.540000 9.5400 9.540 9.540000
```

Torpedo torpedo

16 December 2021, 10:33

Elicited judgements

quantiles	A	B	C	D	E
0.25	0.4327	0.0206	0.0430	0.1953	1e-04
0.50	0.5760	0.0343	0.0763	0.3337	2e-04
0.75	0.7073	0.0527	0.0885	0.4927	3e-04

Dirichlet density function and parameters

Define the vector of unknown population proportions as

$$\theta := (\theta_1, \dots, \theta_k),$$

with $= 5$. We write

$$\theta \sim Dirichlet(a_1, \dots, a_k),$$

with

$$f(\theta) = \frac{\Gamma(a_1 + \dots + a_k)}{\Gamma(a_1) \dots \Gamma(a_k)} \prod_{i=1}^{k} \theta_i^{a_i - 1}.$$

The fitted parameter values $a_1, \dots, a_k$ are as follows:

```
##           A           B           C           D           E
## 3.967885653 0.276077276 0.525007716 2.480731381 0.001612041
```

Comparing the elicited marginals with the marginals from the Dirichlet fit

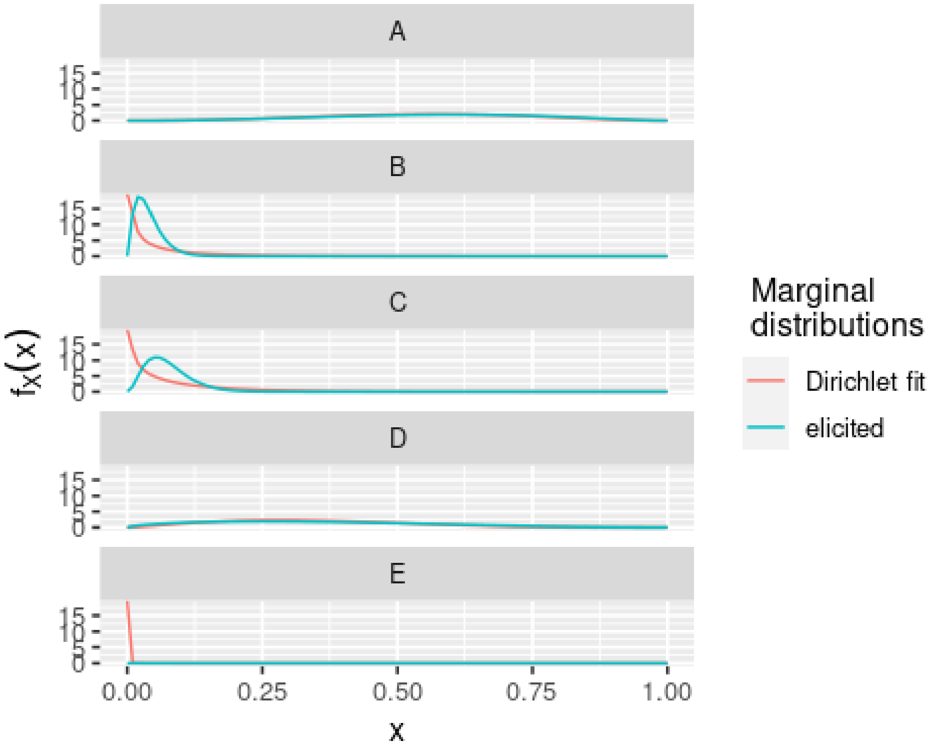

```
##
## Directly elicted beta marginal distributions:
##
##                 A       B       C     D        E
##   shape1    3.480  2.2800  3.1400 1.700 1.80e+00
##   shape2    2.660 55.5000 38.7000 3.090 7.82e+03
##   mean      0.567  0.0395  0.0750 0.355 2.30e-04
##   sd        0.185  0.0254  0.0402 0.199 1.72e-04
##   sum       6.140 57.8000 41.9000 4.780 7.82e+03
##
## Sum of elicited marginal means: 1.036
##
## Beta marginal distributions from Dirichlet fit:
##
##                 A       B       C     D        E
##   shape1    3.970  0.2760  0.5250 2.480 0.001610
##   shape2    3.280  6.9800  6.7300 4.770 7.250000
##   mean      0.547  0.0381  0.0724 0.342 0.000222
##   sd        0.173  0.0666  0.0902 0.165 0.005190
## sum    7.250 7.2500 7.2500 7.250 7.250000
```

Pteroplatytrygon violacea

16 December 2021, 10:37

Elicited judgements

quantiles	A	B	C	D	E
0.25	0.0651	0.4213	0.0287	0.1815	0.0763
0.50	0.0906	0.4900	0.0331	0.2627	0.1224
0.75	0.1229	0.5617	0.0378	0.3320	0.3147

Dirichlet density function and parameters

Define the vector of unknown population proportions as

$$\theta := (\theta_1, \dots, \theta_k),$$

with $= 5$. We write

$$\theta \sim Dirichlet(a_1, \dots, a_k),$$

with

$$f(\theta) = \frac{\Gamma(a_1 + \cdots + a_k)}{\Gamma(a_1) \dots \Gamma(a_k)} \prod_{i=1}^{k} \theta_i^{a_i - 1}.$$

The fitted parameter values $a_1, \dots, a_k$ are as follows:

```
##         A         B         C         D         E
## 1.3066755 6.6221418 0.4519658 3.6003780 2.6852997
```

Comparing the elicited marginals with the marginals from the Dirichlet fit

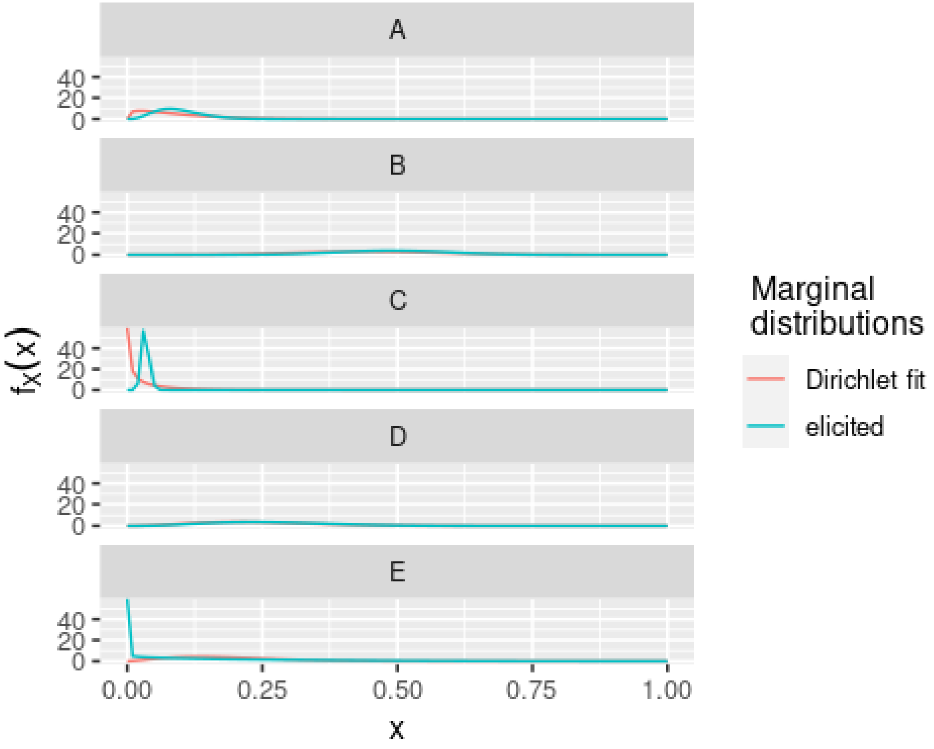


```
##
##        Directly        elicted        beta        marginal        distributions:
##
##                          A          B              C          D        E
##       shape1         4.3800     11.500       2.35e+01      4.030    0.845
##        shape2       40.8000     11.900       6.78e+02     11.100    3.400
##     mean                   0.0969       0.491     3.35e-02      0.267    0.199
##     sd                       0.0435       0.101     6.79e-03      0.110    0.174
##      sum                     45.2000      23.300      7.01e+02     15.100    4.240
##
##        Sum        of        elicited        marginal        means:        1.088
##
##       Beta       marginal       distributions       from       Dirichlet       fit:
##
##                          A          B             C          D          E
##     shape1       1.3100        6.620        0.4520       3.600      2.6900
##       shape2      13.4000         8.040       14.2000      11.100     12.0000
##    mean             0.0891        0.452       0.0308      0.245      0.1830
##    sd                  0.0720      0.126        0.0437      0.109      0.0977
## sum     14.7000 14.700 14.7000 14.700 14.7000
```

Leucoraja circularis

16 December 2021, 10:41

Elicited judgements

quantiles	A	B	C	D	E
0.25	0.6397	1e-04	0.0783	0.0873	1e-04
0.50	0.7923	2e-04	0.0830	0.1163	2e-04
0.75	0.9020	3e-04	0.0878	0.1477	3e-04

Dirichlet density function and parameters

Define the vector of unknown population proportions as

$$\theta := (\theta_1, \dots, \theta_k),$$

with = 5 . We write

$$\theta \sim Dirichlet(a_1, \dots, a_k),$$

with

$$f(\theta) = \frac{\Gamma(a_1 + \cdots + a_k)}{\Gamma(a_1) \dots \Gamma(a_k)} \prod_{i=1}^{k} \theta_i^{a_i - 1}.$$

The fitted parameter values $a_1, \dots, a_k$ are as follows:

```
##           A           B           C           D           E
## 10.864337200  0.003323426  1.199555819  1.740410291  0.003323426
```

Comparing the elicited marginals with the marginals from the Dirichlet fit

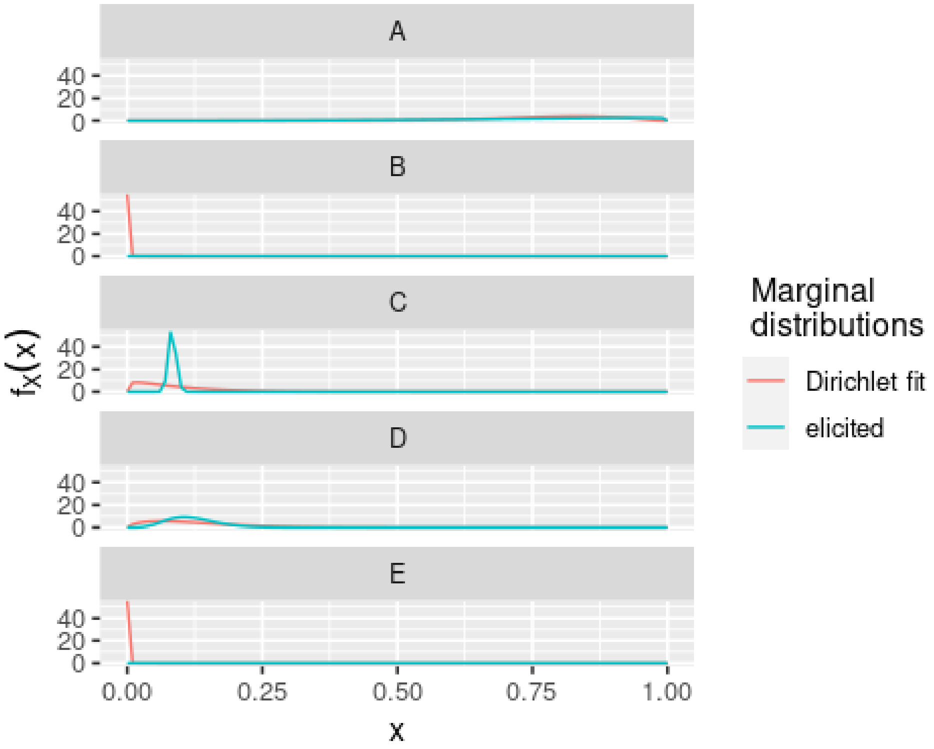

```
##
##        Directly        elicted        beta        marginal        distributions:
##
##                       A                 B                 C                 D                 E
##      shape1      3.410      1.80e+00      1.28e+02            6.1300      1.80e+00
##       shape2        1.120        7.82e+03        1.41e+03      44.7000      7.82e+03
##     mean                  0.753      2.30e-04      8.32e-02            0.1210      2.30e-04
##    sd                          0.183      1.72e-04      7.05e-03            0.0452      1.72e-04
##     sum                         4.520      7.82e+03      1.53e+03      50.8000      7.82e+03
##
##        Sum          of          elicited          marginal          means:          0.957
##
##       Beta        marginal        distributions        from        Dirichlet        fit:
##
##                         A                 B                 C                 D                 E
##      shape1      10.900      3.32e-03            1.2000            1.7400      3.32e-03
##       shape2                  2.950        1.38e+01      12.6000      12.1000      1.38e+01
##    mean                        0.787      2.41e-04            0.0869            0.1260      2.41e-04
##   sd                                 0.106      4.03e-03            0.0732            0.0862      4.03e-03
## sum    13.800 1.38e+01 13.8000 13.8000 1.38e+01
```

Leucoraja fullonica

16 December 2021, 10:44

Elicited judgements

quantiles	A	B	C	D	E
0.25	0.6083	1e-04	0.0319	0.1301	1e-04
0.50	0.7347	2e-04	0.0360	0.1800	2e-04
0.75	0.8383	3e-04	0.0403	0.2385	3e-04

Dirichlet density function and parameters

Define the vector of unknown population proportions as

$$\theta := (\theta_1, \dots, \theta_k),$$

with $= 5$. We write

$$\theta \sim Dirichlet(a_1, \dots, a_k),$$

with

$$f(\theta) = \frac{\Gamma(a_1 + \cdots + a_k)}{\Gamma(a_1) \dots \Gamma(a_k)} \prod_{i=1}^{k} \theta_i^{a_i - 1}.$$

The fitted parameter values $a_1, \dots, a_k$ are as follows:

```
##                    A                    B                    C                    D                    E
## 9.872657122 0.003190125 0.502783982 2.618066182 0.003190125
```

Comparing the elicited marginals with the marginals from the Dirichlet fit

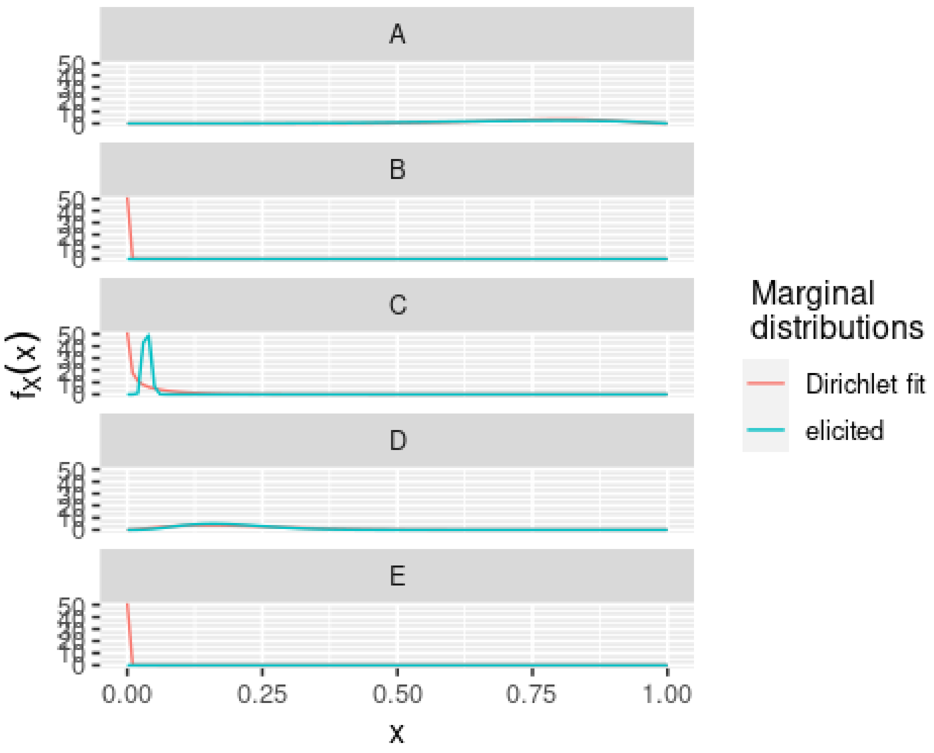


```
##
##      Directly     elicted     beta     marginal     distributions:
##
##                        A             B             C            D            E
##   shape1      4.940      1.80e+00      3.24e+01             4.3600      1.80e+00
##     shape2      1.990      7.82e+03      8.60e+02      18.7000      7.82e+03
##   mean                 0.713      2.30e-04      3.63e-02        0.1890      2.30e-04
##   sd                       0.161      1.72e-04      6.26e-03        0.0799      1.72e-04
##   sum                      6.920      7.82e+03      8.93e+02      23.0000      7.82e+03
##
##      Sum      of      elicited      marginal      means:      0.939
##
##      Beta      marginal      distributions      from      Dirichlet      fit:
##
##                        A             B             C            D            E
##   shape1          9.870      3.19e-03          0.5030        2.620      3.19e-03
##     shape2           3.130      1.30e+01      12.5000      10.400      1.30e+01
##   mean                0.759      2.45e-04      0.0387        0.201      2.45e-04
##   sd                      0.114      4.19e-03        0.0515        0.107      4.19e-03
## sum    13.000 1.30e+01 13.0000 13.000 1.30e+01
```

Rhinobatos rhinobatos

16 December 2021, 10:48

Elicited judgements

quantiles	A	B	C	D	E
0.25	0.2677	1e-04	0.142	0.2777	1e-04
0.50	0.3380	2e-04	0.227	0.4257	2e-04
0.75	0.4133	3e-04	0.384	0.5530	3e-04

Dirichlet density function and parameters

Define the vector of unknown population proportions as

$$\theta := (\theta_1, \ldots, \theta_k),$$

with $= 5$. We write

$$\theta \sim Dirichlet(a_1, \ldots, a_k),$$

with

$$f(\theta) = \frac{\Gamma(a_1 + \cdots + a_k)}{\Gamma(a_1) \ldots \Gamma(a_k)} \prod_{i=1}^{k} \theta_i^{a_i - 1}.$$

The fitted parameter values $a_1, \ldots, a_k$ are as follows:

```
##          A          B          C          D          E
## 2.80247936 0.00188026 2.17846015 3.47519515 0.00188026
```

Comparing the elicited marginals with the marginals from the Dirichlet fit

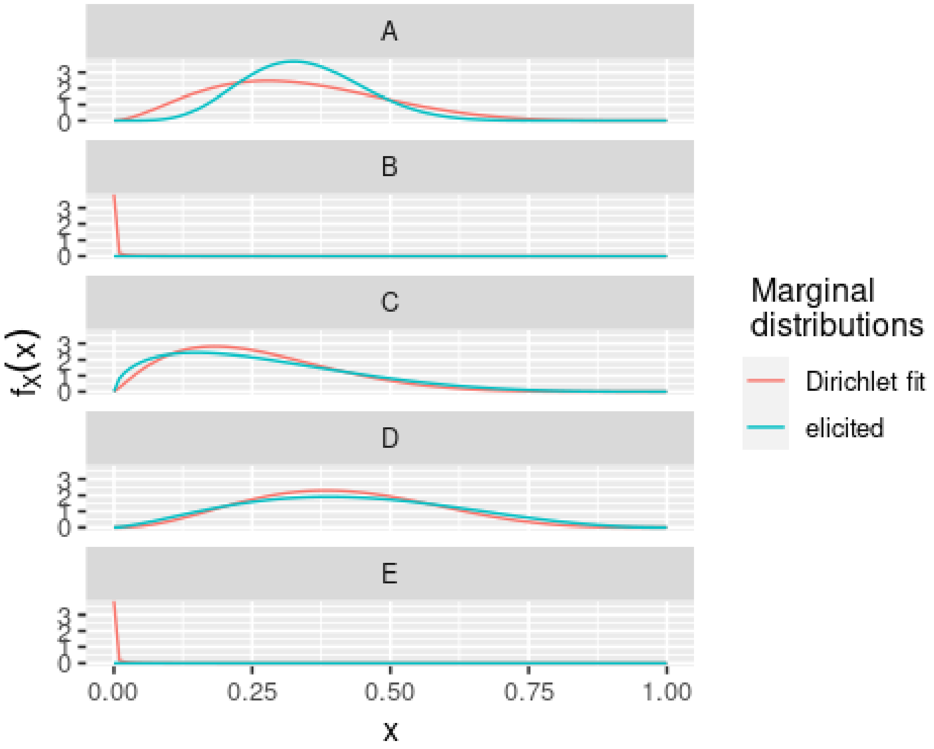

```
##
##         Directly          elicted         beta         marginal         distributions:
##
##                            A                 B           C           D                E
##        shape1             6.680       1.80e+00      1.580       2.560        1.80e+00
##         shape2        12.800       7.82e+03        4.350       3.450        7.82e+03
##      mean                       0.343      2.30e-04      0.267      0.426      2.30e-04
##     sd                             0.105      1.72e-04      0.168      0.187      1.72e-04
##      sum                      19.500      7.82e+03      5.930      6.000      7.82e+03
##
##         Sum          of          elicited          marginal          means:          1.037
##
##       Beta        marginal        distributions         from        Dirichlet          fit:
##
##                           A                  B           C           D                E
##         shape1        2.800         0.001880        2.180        3.480        0.001880
##         shape2        5.660         8.460000        6.280        4.980        8.460000
##       mean                     0.331      0.000222        0.258      0.411      0.000222
##      sd                            0.153      0.004850       0.142      0.160      0.004850
## sum    8.460 8.460000 8.460 8.460 8.460000
```

Torpedo marmorata

16 December 2021, 10:51

Elicited judgements

quantiles	A	B	C	D	E
0.25	0.5087	1e-04	0.0713	0.1748	1e-04
0.50	0.6223	2e-04	0.1357	0.2480	2e-04
0.75	0.7263	3e-04	0.2702	0.3337	3e-04

Dirichlet density function and parameters

Define the vector of unknown population proportions as

$$\theta := (\theta_1, \ldots, \theta_k),$$

with $= 5$. We write

$$\theta \sim Dirichlet(a_1, \ldots, a_k),$$

with

$$f(\theta) = \frac{\Gamma(a_1 + \cdots + a_k)}{\Gamma(a_1) \ldots \Gamma(a_k)} \prod_{i=1}^{k} \theta_i^{a_i - 1}.$$

The fitted parameter values $a_1, \ldots, a_k$ are as follows:

```
##              A              B              C              D              E
## 5.410034229 0.002031845 1.595520427 2.294702513 0.002031845
```

Comparing the elicited marginals with the marginals from the Dirichlet fit

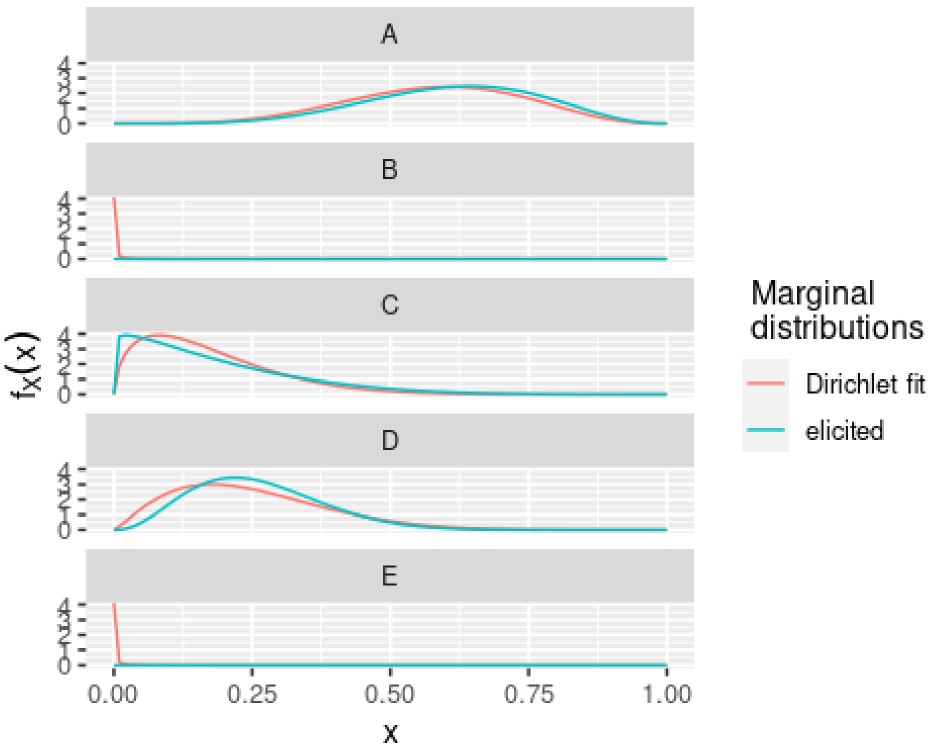


```
##
##      Directly        elicted         beta        marginal        distributions:
##
##                          A                   B          C             D                 E
##      shape1        5.700        1.80e+00        1.090                3.560        1.80e+00
##       shape2        3.590         7.82e+03        4.930        10.100        7.82e+03
##     mean                    0.613       2.30e-04       0.181           0.260        2.30e-04
##    sd                              0.152       1.72e-04       0.145           0.115       1.72e-04
##     sum                            9.290       7.82e+03       6.010       13.700       7.82e+03
##
##       Sum          of          elicited          marginal          means:          1.055
##
##      Beta        marginal        distributions        from        Dirichlet          fit:
##
##                           A                    B           C             D                 E
##       shape1        5.410          0.002030          1.600        2.290          0.002030
##       shape2        3.890          9.300000          7.710        7.010          9.300000
##      mean                    0.581       0.000218        0.171        0.247        0.000218
##     sd                                0.154       0.004600        0.117        0.134        0.004600
## sum    9.300 9.300000 9.300 9.300 9.300000
```

Raja clavata

16 December 2021, 10:55

Elicited judgements

quantiles	A	B	C	D	E
0.25	0.6320	1e-04	0.0483	0.2149	1e-04
0.50	0.7817	2e-04	0.0583	0.3023	2e-04
0.75	0.8907	3e-04	0.0683	0.3620	3e-04

Dirichlet density function and parameters

Define the vector of unknown population proportions as

$$\theta := (\theta_1, \ldots, \theta_k),$$

with = 5 . We write

$$\theta \sim Dirichlet(a_1, \ldots, a_k),$$

with

$$f(\theta) = \frac{\Gamma(a_1 + \cdots + a_k)}{\Gamma(a_1) \ldots \Gamma(a_k)} \prod_{i=1}^{k} \theta_i^{a_i - 1}.$$

The fitted parameter values $a_1, \ldots, a_k$ are as follows:

```
##                    A                       B                      C                      D                     E
## 7.369990089 0.002277572 0.585045938 2.968769142 0.002277572
```

Comparing the elicited marginals with the marginals from the Dirichlet fit

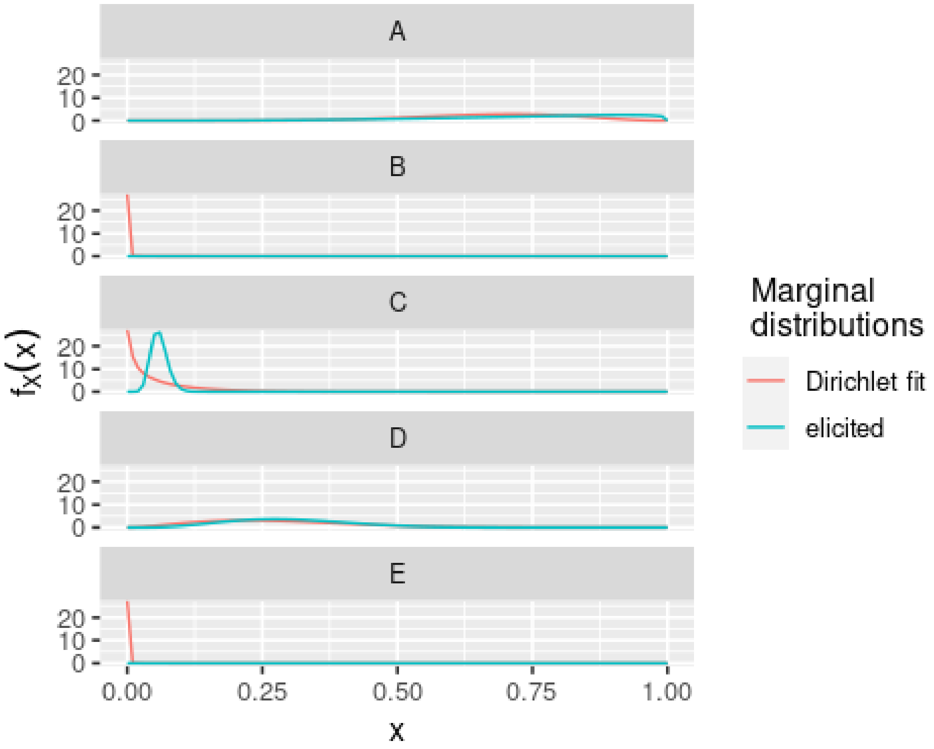

```
##
##       Directly       elicted       beta       marginal       distributions:
##
##                     A                B               C           D          E
##      shape1    3.610    1.80e+00          14.6000        5.010    1.80e+00
##       shape2     1.230     7.82e+03       232.0000    11.700    7.82e+03
##     mean          0.746    2.30e-04           0.0592       0.300    2.30e-04
##     sd               0.180    1.72e-04           0.0150       0.109    1.72e-04
##      sum                4.840     7.82e+03     247.0000    16.700    7.82e+03
##
##       Sum       of       elicited       marginal       means:       1.106
##
##      Beta      marginal      distributions       from      Dirichlet       fit:
##
##                       A                B               C           D          E
##      shape1          7.370     2.28e-03          0.5850         2.970    2.28e-03
##       shape2           3.560     1.09e+01      10.3000        7.960    1.09e+01
##     mean                 0.674     2.08e-04           0.0535       0.272    2.08e-04
##     sd                      0.136     4.18e-03           0.0652       0.129    4.18e-03
## sum    10.900 1.09e+01 10.9000 10.900 1.09e+01
```

Squatina oculata

16 December 2021, 10:59

Elicited judgements

quantiles	A	B	C	D	E
0.25	0.3907	1e-04	0.0853	0.2330	1e-04
0.50	0.5377	2e-04	0.1847	0.3663	2e-04
0.75	0.6743	3e-04	0.2842	0.5087	3e-04

Dirichlet density function and parameters

Define the vector of unknown population proportions as

$$\theta := (\theta_1, \dots, \theta_k),$$

with $= 5$. We write

$$\theta \sim Dirichlet(a_1, \dots, a_k),$$

with

$$f(\theta) = \frac{\Gamma(a_1 + \cdots + a_k)}{\Gamma(a_1) \dots \Gamma(a_k)} \prod_{i=1}^{k} \theta_i^{a_i - 1}.$$

The fitted parameter values $a_1, \dots, a_k$ are as follows:

```
##           A              B              C              D              E
## 2.84390949 0.00123169 1.10454725 2.02747180 0.00123169
```

Comparing the elicited marginals with the marginals from the Dirichlet fit

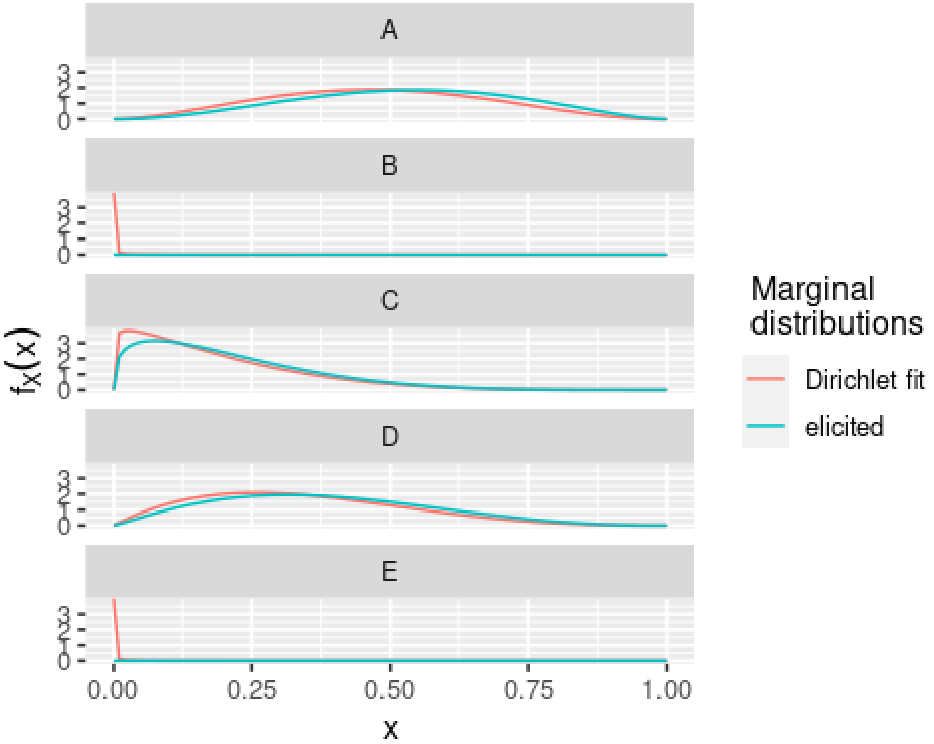


```
##
##   Directly   elicted   beta   marginal   distributions:
##
##                    A              B          C          D              E
##   shape1       3.120       1.80e+00      1.330      2.190       1.80e+00
##   shape2       2.750       7.82e+03      5.110      3.580       7.82e+03
##  mean                 0.532       2.30e-04      0.207      0.379  2.30e-04
##  sd                        0.190       1.72e-04      0.148      0.187  1.72e-04
##  sum                   5.870       7.82e+03      6.440      5.760  7.82e+03
##
##   Sum   of   elicited   marginal   means:   1.118
##
##   Beta   marginal   distributions   from   Dirichlet   fit:
##
##                    A              B          C          D              E
##   shape1       2.840       0.001230      1.100      2.030       0.001230
##   shape2       3.130       5.980000      4.870      3.950       5.980000
##  mean                 0.476       0.000206      0.185      0.339  0.000206
##  sd                        0.189       0.005430      0.147      0.179  0.005430
## sum     5.980 5.980000 5.980 5.980 5.980000
```

Chimaera monstrosa

16 December 2021, 11:04

Elicited judgements

quantiles	A	B	C	D	E
0.25	0.7787	1e-04	0.0128	1e-04	1e-04
0.50	0.9450	2e-04	0.0151	2e-04	2e-04
0.75	0.9950	3e-04	0.0176	3e-04	3e-04

Dirichlet density function and parameters

Define the vector of unknown population proportions as

$$\theta := (\theta_1, \ldots, \theta_k),$$

with $= 5$. We write

$$\theta \sim Dirichlet(a_1, \ldots, a_k),$$

with

$$f(\theta) = \frac{\Gamma(a_1 + \cdots + a_k)}{\Gamma(a_1) \ldots \Gamma(a_k)} \prod_{i=1}^{k} \theta_i^{a_i - 1}.$$

The fitted parameter values $a_1, \ldots, a_k$ are as follows:

```
##            A            B            C            D            E
## 2.6556297803 0.0007219776 0.0481334570 0.0007219776 0.0007219776
```

Comparing the elicited marginals with the marginals from the Dirichlet fit

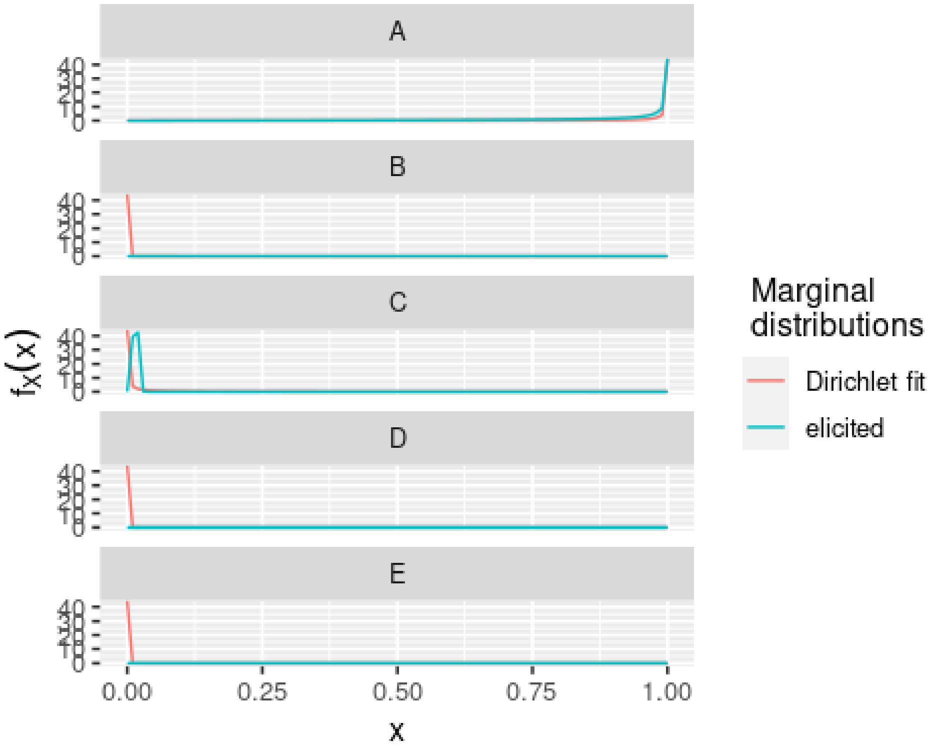

```
##
##        Directly        elicted        beta        marginal        distributions:
##
##                        A              B              C              D              E
##       shape1        1.680       1.80e+00       1.80e+01       1.80e+00       1.80e+00
##       shape2        0.302       7.82e+03       1.15e+03       7.82e+03       7.82e+03
##      mean                  0.847       2.30e-04       1.54e-02       2.30e-04       2.30e-04
##     sd                        0.208       1.72e-04       3.59e-03       1.72e-04       1.72e-04
##     sum                        1.980       7.82e+03       1.17e+03       7.82e+03       7.82e+03
##
##        Sum        of        elicited        marginal        means:        0.864
##
##       Beta        marginal        distributions        from        Dirichlet        fit:
##
##                          A              B              C              D              E
##       shape1        2.6600       0.000722       0.0481       0.000722       0.000722
##       shape2        0.0503       2.710000       2.6600       2.710000       2.710000
##      mean                  0.9810       0.000267       0.0178       0.000267       0.000267
##     sd                        0.0702       0.008480       0.0687       0.008480       0.008480
## sum    2.7100 2.710000 2.7100 2.710000 2.710000
```

Pristis pristis

16 December 2021, 11:08

Elicited judgements

quantiles	A	B	C	D	E
0.25	0.1336	1e-04	0.1900	0.2760	1e-04
0.50	0.1639	2e-04	0.3160	0.4210	2e-04
0.75	0.2002	3e-04	0.4363	0.5187	3e-04

Dirichlet density function and parameters

Define the vector of unknown population proportions as

$$\theta := (\theta_1, \ldots, \theta_k),$$

with $= 5$. We write

$$\theta \sim Dirichlet(a_1, \ldots, a_k),$$

with

$$f(\theta) = \frac{\Gamma(a_1 + \cdots + a_k)}{\Gamma(a_1) \ldots \Gamma(a_k)} \prod_{i=1}^{k} \theta_i^{a_i - 1}.$$

The fitted parameter values $a_1, \ldots, a_k$ are as follows:

```
##                  A                B                C                D                E
## 1.834741013 0.002509129 3.565815580 4.499536539 0.002509129
```

Comparing the elicited marginals with the marginals from the Dirichlet fit

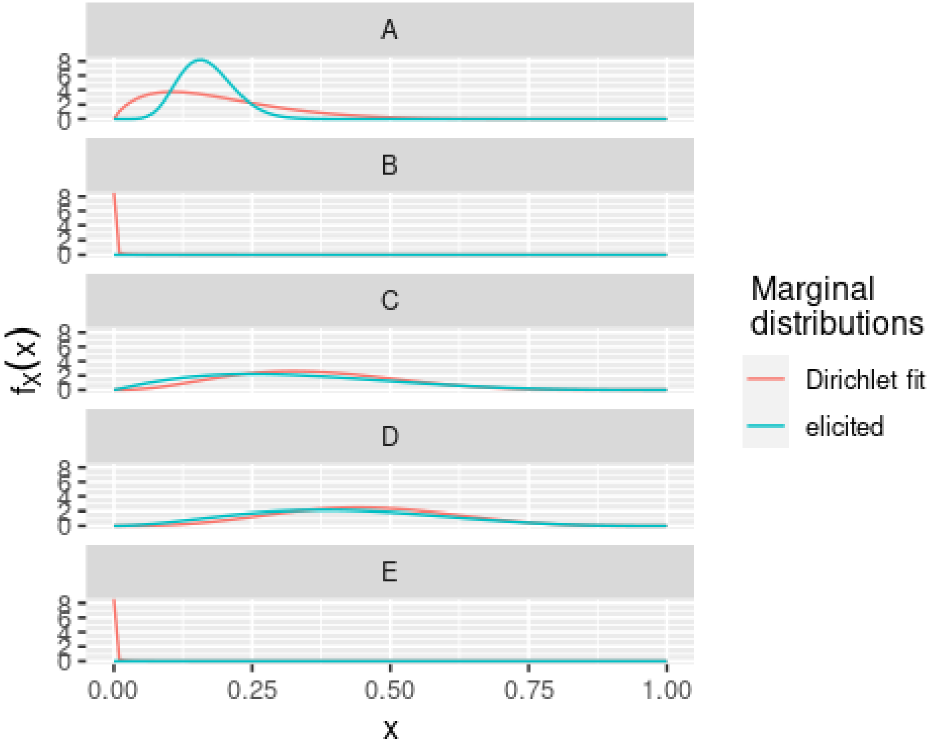


```
##
##      Directly       elicted       beta       marginal       distributions:
##
##                             A               B          C          D              E
##     shape1             9.5600       1.80e+00      2.130      3.040       1.80e+00
##      shape2        47.2000       7.82e+03      4.380      4.320       7.82e+03
##    mean                        0.1680      2.30e-04      0.327      0.413      2.30e-04
##   sd                             0.0493      1.72e-04      0.171      0.170      1.72e-04
##    sum                        56.7000      7.82e+03      6.510      7.360      7.82e+03
##
##      Sum        of        elicited        marginal        means:        0.91
##
##     Beta      marginal      distributions       from       Dirichlet       fit:
##
##                          A                    B          C          D              E
##      shape1      1.830         0.002510        3.570      4.500       0.002510
##      shape2      8.070         9.900000        6.340      5.410       9.900000
##     mean                   0.185      0.000253      0.360      0.454      0.000253
##    sd                          0.118      0.004820      0.145      0.151      0.004820
## sum     9.910 9.910000 9.910 9.910 9.910000
```

Squatina aculeata

16 December 2021, 11:12

Elicited judgements

quantiles	A	B	C	D	E
0.25	0.3907	1e-04	0.0853	0.2330	1e-04
0.50	0.5377	2e-04	0.1847	0.3663	2e-04
0.75	0.6743	3e-04	0.2842	0.5087	3e-04

Dirichlet density function and parameters

Define the vector of unknown population proportions as

$$\theta := (\theta_1, \ldots, \theta_k),$$

with $= 5$. We write

$$\theta \sim Dirichlet(a_1, \ldots, a_k),$$

with

$$f(\theta) = \frac{\Gamma(a_1 + \cdots + a_k)}{\Gamma(a_1) \ldots \Gamma(a_k)} \prod_{i=1}^{k} \theta_i^{a_i - 1}.$$

The fitted parameter values $a_1, \ldots, a_k$ are as follows:

```
##          A          B          C          D          E
## 2.84390949 0.00123169 1.10454725 2.02747180 0.00123169
```

Comparing the elicited marginals with the marginals from the Dirichlet fit

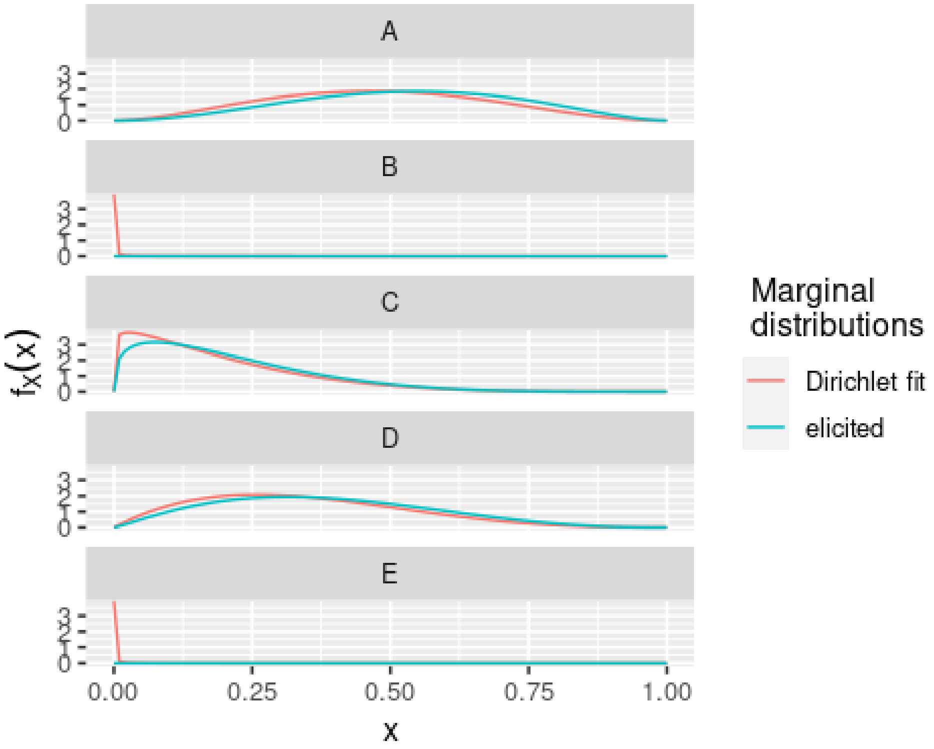

```
##
##         Directly         elicted         beta         marginal         distributions:
##
##                           A                   B            C             D              E
##         shape1         3.120         1.80e+00         1.330         2.190         1.80e+00
##         shape2         2.750         7.82e+03         5.110         3.580         7.82e+03
##       mean                    0.532       2.30e-04       0.207       0.379       2.30e-04
##      sd                           0.190       1.72e-04       0.148       0.187       1.72e-04
##      sum                            5.870       7.82e+03       6.440       5.760       7.82e+03
##
##         Sum         of         elicited         marginal         means:         1.118
##
##        Beta         marginal         distributions         from         Dirichlet         fit:
##
##                           A                   B            C             D              E
##         shape1         2.840         0.001230         1.100         2.030         0.001230
##         shape2         3.130         5.980000         4.870         3.950         5.980000
##       mean                    0.476       0.000206       0.185       0.339       0.000206
##      sd                           0.189       0.005430       0.147       0.179       0.005430
## sum    5.980 5.980000 5.980 5.980 5.980000
```

Dipturus oxyrinchus

16 December 2021, 11:17

Elicited judgements

quantiles	A	B	C	D	E
0.25	0.6497	1e-04	0.0783	0.0830	1e-04
0.50	0.7987	2e-04	0.0830	0.1097	2e-04
0.75	0.9043	3e-04	0.0878	0.1390	3e-04

Dirichlet density function and parameters

Define the vector of unknown population proportions as

$$\theta := (\theta_1, \dots, \theta_k),$$

with = 5 . We write

$$\theta \sim Dirichlet(a_1, \dots, a_k),$$

with

$$f(\theta) = \frac{\Gamma(a_1 + \dots + a_k)}{\Gamma(a_1) \dots \Gamma(a_k)} \prod_{i=1}^{k} \theta_i^{a_i - 1}.$$

The fitted parameter values $a_1, \dots, a_k$ are as follows:

```
##                    A                B                C                D                E
## 11.425181379   0.003464764   1.250570175   1.711536486   0.003464764
```

Comparing the elicited marginals with the marginals from the Dirichlet fit

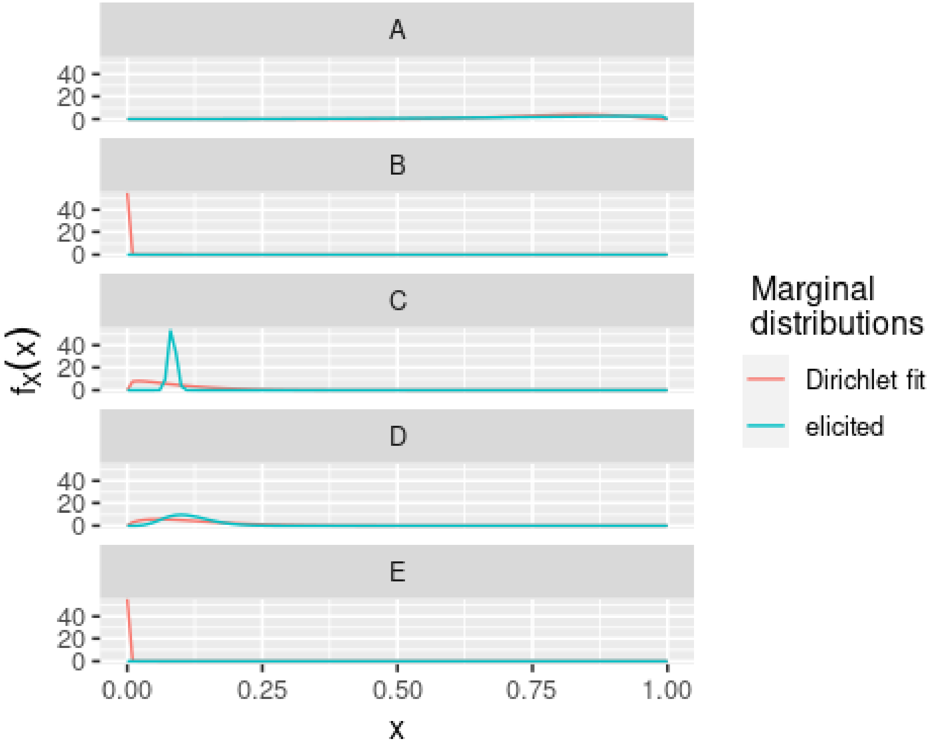


```
##
##      Directly       elicted       beta       marginal       distributions:
##
##                        A                 B                 C            D              E
##      shape1      3.570       1.80e+00       1.28e+02              6.410       1.80e+00
##       shape2       1.130        7.82e+03       1.41e+03       49.900       7.82e+03
##     mean                 0.760       2.30e-04       8.32e-02           0.114       2.30e-04
##     sd                        0.179      1.72e-04      7.05e-03           0.042       1.72e-04
##     sum                      4.690       7.82e+03      1.53e+03       56.300       7.82e+03
##
##      Sum         of        elicited          marginal          means:          0.957
##
##      Beta       marginal       distributions        from       Dirichlet        fit:
##
##                          A                 B                 C            D              E
##      shape1      11.400      3.46e-03            1.2500          1.7100       3.46e-03
##       shape2               2.970       1.44e+01       13.1000     12.7000       1.44e+01
##     mean                  0.794      2.41e-04        0.0869          0.1190       2.41e-04
##     sd                         0.103     3.95e-03          0.0718          0.0825       3.95e-03
## sum    14.400 1.44e+01 14.4000 14.4000 1.44e+01
```

Taeniura grabata

16 December 2021, 11:28

Elicited judgements

quantiles	A	B	C	D	E
0.25	0.522	1e-04	0.1013	0.1373	1e-04
0.50	0.638	2e-04	0.1363	0.1950	2e-04
0.75	0.743	3e-04	0.1865	0.2613	3e-04

Dirichlet density function and parameters

Define the vector of unknown population proportions as

$$\theta := (\theta_1, \ldots, \theta_k),$$

with $= 5$. We write

$$\theta \sim Dirichlet(a_1, \ldots, a_k),$$

with

$$f(\theta) = \frac{\Gamma(a_1 + \cdots + a_k)}{\Gamma(a_1) \ldots \Gamma(a_k)} \prod_{i=1}^{k} \theta_i^{a_i - 1}.$$

The fitted parameter values $a_1, \ldots, a_k$ are as follows:

```
##           A           B           C           D           E
## 9.157380923 0.003361376 2.134813999 2.993798721 0.003361376
```

Comparing the elicited marginals with the marginals from the Dirichlet fit

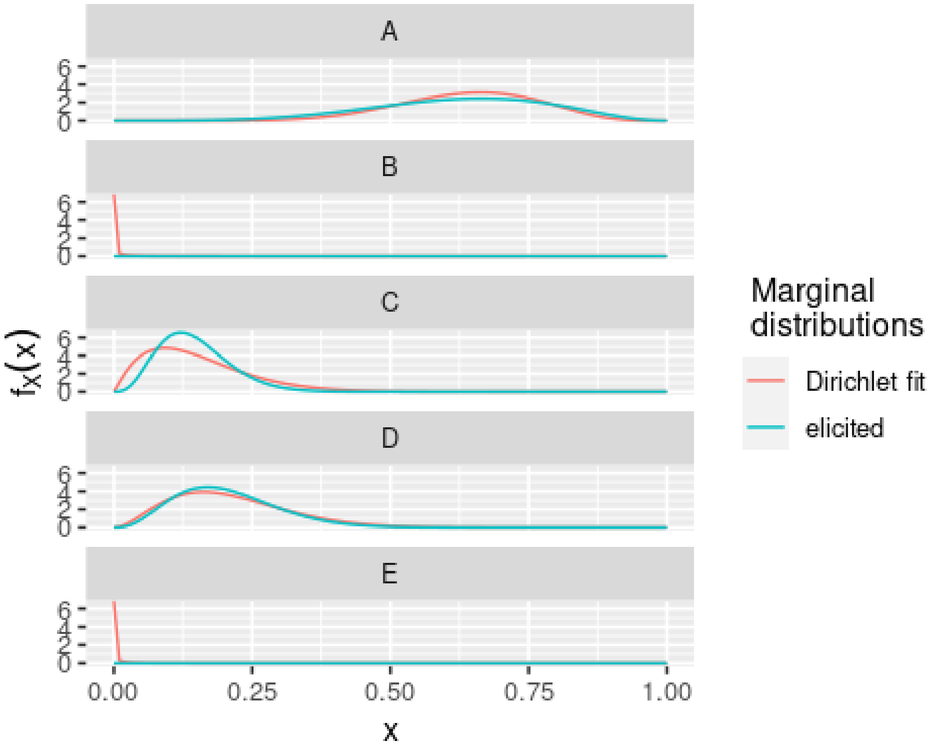

```
##
##        Directly         elicted         beta         marginal         distributions:
##
##                       A                  B              C             D               E
##       shape1      5.560       1.80e+00             4.4300            3.840      1.80e+00
##         shape2        3.300       7.82e+03          25.9000       14.900      7.82e+03
##      mean                  0.628      2.30e-04          0.1460           0.205      2.30e-04
##     sd                         0.154     1.72e-04           0.0632          0.091     1.72e-04
##      sum                          8.850       7.82e+03       30.3000       18.700      7.82e+03
##
##        Sum          of          elicited          marginal          means:           0.98
##
##       Beta        marginal        distributions          from          Dirichlet          fit:
##
##                          A                 B              C             D               E
##      shape1              9.160       3.36e-03             2.1300           2.990      3.36e-03
##        shape2                 5.140        1.43e+01        12.2000       11.300      1.43e+01
##     mean                         0.641      2.35e-04          0.1490           0.209      2.35e-04
##    sd                                0.123      3.92e-03          0.0911           0.104     3.92e-03
## sum    14.300 1.43e+01 14.3000 14.300 1.43e+01
```

Bathytoshia lata

16 December 2021, 11:32

Elicited judgements

quantiles	A	B	C	D	E
0.25	0.3977	1e-04	0.1937	0.1457	1e-04
0.50	0.4440	2e-04	0.3227	0.1960	2e-04
0.75	0.4963	3e-04	0.4287	0.2523	3e-04

Dirichlet density function and parameters

Define the vector of unknown population proportions as

$$\theta := (\theta_1, \ldots, \theta_k),$$

with $= 5$. We write

$$\theta \sim Dirichlet(a_1, \ldots, a_k),$$

with

$$f(\theta) = \frac{\Gamma(a_1 + \cdots + a_k)}{\Gamma(a_1) \ldots \Gamma(a_k)} \prod_{i=1}^{k} \theta_i^{a_i - 1}.$$

The fitted parameter values $a_1, \ldots, a_k$ are as follows:

```
##           A           B           C           D           E
## 8.123184755 0.004194047 5.973458043 3.703880238 0.004194047
```

Comparing the elicited marginals with the marginals from the Dirichlet fit

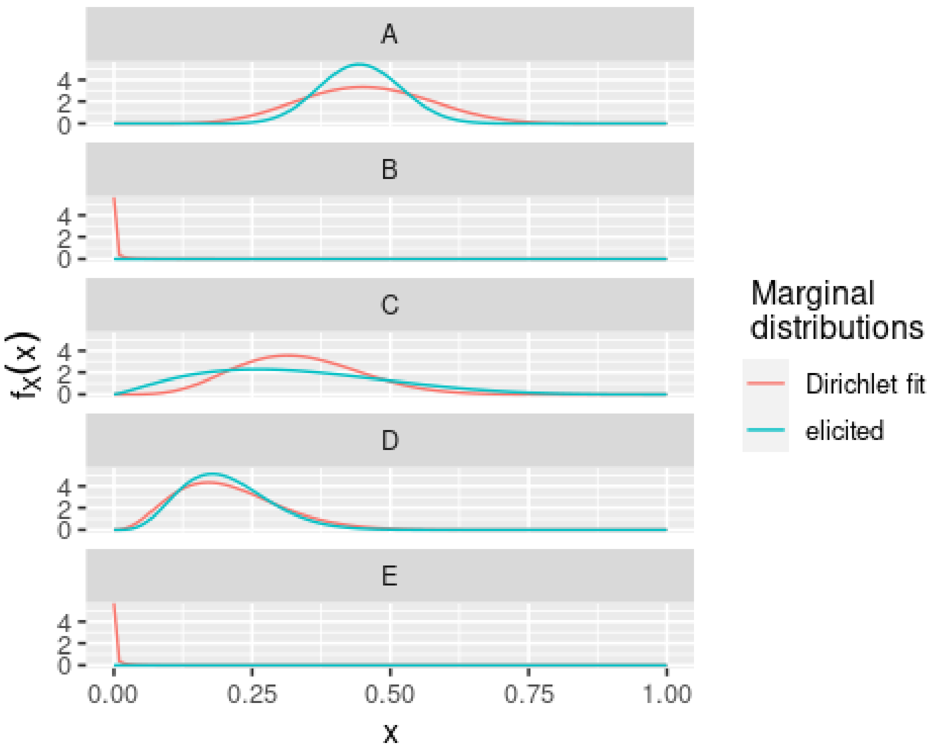


```
##
## Directly elicted beta marginal distributions:
##
##                 A        B      C       D        E
## shape1    20.7000 1.80e+00  2.300  5.1600 1.80e+00
## shape2    25.7000 7.82e+03  4.720 20.2000 7.82e+03
## mean       0.4460 2.30e-04  0.328  0.2030 2.30e-04
## sd         0.0722 1.72e-04  0.166  0.0784 1.72e-04
## sum       46.5000 7.82e+03  7.020 25.3000 7.82e+03
##
## Sum of elicited marginal means: 0.978
##
## Beta marginal distributions from Dirichlet fit:
##
##              A        B      C       D        E
## shape1   8.120 4.19e-03  5.970  3.7000 4.19e-03
## shape2   9.690 1.78e+01 11.800 14.1000 1.78e+01
## mean     0.456 2.36e-04  0.335  0.2080 2.36e-04
## sd       0.115 3.54e-03  0.109  0.0936 3.54e-03
## sum    17.800 1.78e+01 17.800 17.8000 1.78e+01
```

Dasyatis pastinaca

16 December 2021, 11:35

Elicited judgements

quantiles	A	B	C	D	E
0.25	0.4257	1e-04	0.1130	0.1597	1e-04
0.50	0.4913	2e-04	0.2523	0.2117	2e-04
0.75	0.5553	3e-04	0.4240	0.2693	3e-04

Dirichlet density function and parameters

Define the vector of unknown population proportions as

$$\theta := (\theta_1, \dots, \theta_k),$$

with $= 5$. We write

$$\theta \sim Dirichlet(a_1, \dots, a_k),$$

with

$$f(\theta) = \frac{\Gamma(a_1 + \dots + a_k)}{\Gamma(a_1) \dots \Gamma(a_k)} \prod_{i=1}^{k} \theta_i^{a_i - 1}.$$

The fitted parameter values $a_1, \dots, a_k$ are as follows:

```
##           A           B           C           D           E
## 5.771317637 0.002708178 3.399439636 2.571278482 0.002708178
```

Comparing the elicited marginals with the marginals from the Dirichlet fit

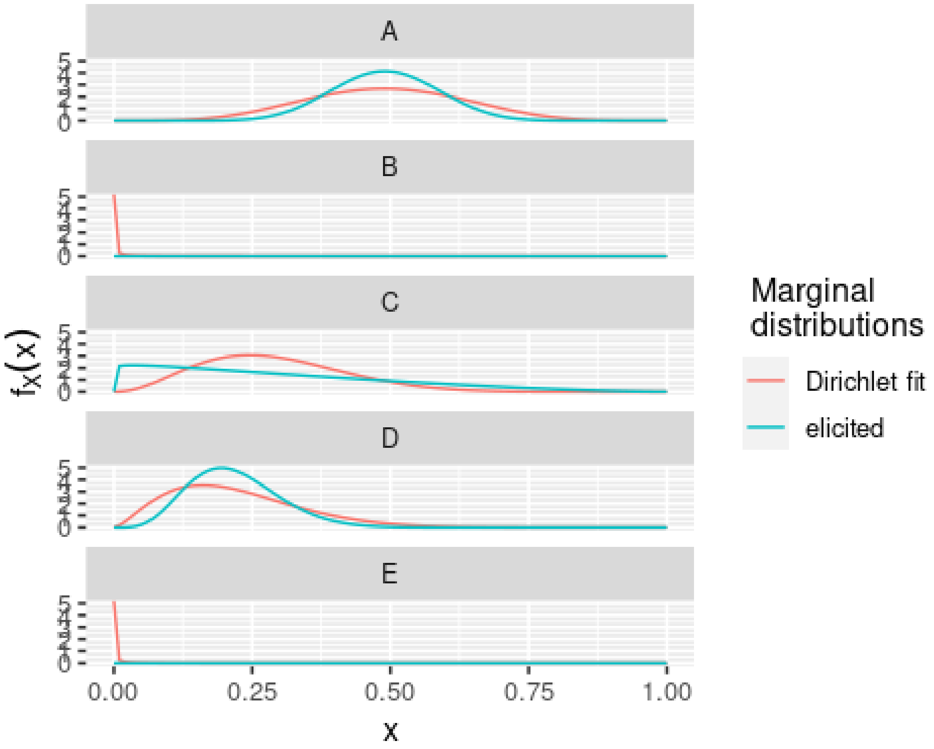

```
##
##        Directly        elicted        beta        marginal        distributions:
##
##                            A                 B         C             D            E
##      shape1       13.4000      1.80e+00      1.050              5.5600     1.80e+00
##       shape2        13.9000       7.82e+03      2.590       19.9000       7.82e+03
##     mean                    0.4910      2.30e-04     0.289          0.2190     2.30e-04
##     sd                        0.0939     1.72e-04     0.210          0.0804     1.72e-04
##      sum                    27.3000      7.82e+03      3.650       25.4000      7.82e+03
##
##        Sum         of         elicited         marginal         means:           0.999
##
##       Beta        marginal        distributions        from       Dirichlet          fit:
##
##                           A                 B          C           D            E
##      shape1             5.770      2.71e-03          3.400        2.570      2.71e-03
##      shape2             5.980      1.17e+01          8.350        9.180      1.17e+01
##     mean                    0.491      2.31e-04        0.289         0.219     2.31e-04
##    sd                          0.140     4.25e-03        0.127          0.116     4.25e-03
## sum    11.700 1.17e+01 11.700 11.700 1.17e+01
```

Rostroraja alba

16 December 2021, 12:01

Elicited judgements

quantiles	A	B	C	D	E
0.25	0.4647	1e-04	0.0553	0.2443	1e-04
0.50	0.5413	2e-04	0.1177	0.3477	2e-04
0.75	0.6000	3e-04	0.2500	0.4257	3e-04

Dirichlet density function and parameters

Define the vector of unknown population proportions as

$$\theta := (\theta_1, \dots, \theta_k),$$

with = 5 . We write

$$\theta \sim Dirichlet(a_1, \dots, a_k),$$

with

$$f(\theta) = \frac{\Gamma(a_1 + \dots + a_k)}{\Gamma(a_1) \dots \Gamma(a_k)} \prod_{i=1}^{k} \theta_i^{a_i - 1}.$$

The fitted parameter values $a_1, \dots, a_k$ are as follows:

```
##               A              B              C              D              E
## 6.530149818 0.002809544 2.020835884 4.227644247 0.002809544
```

Comparing the elicited marginals with the marginals from the Dirichlet fit

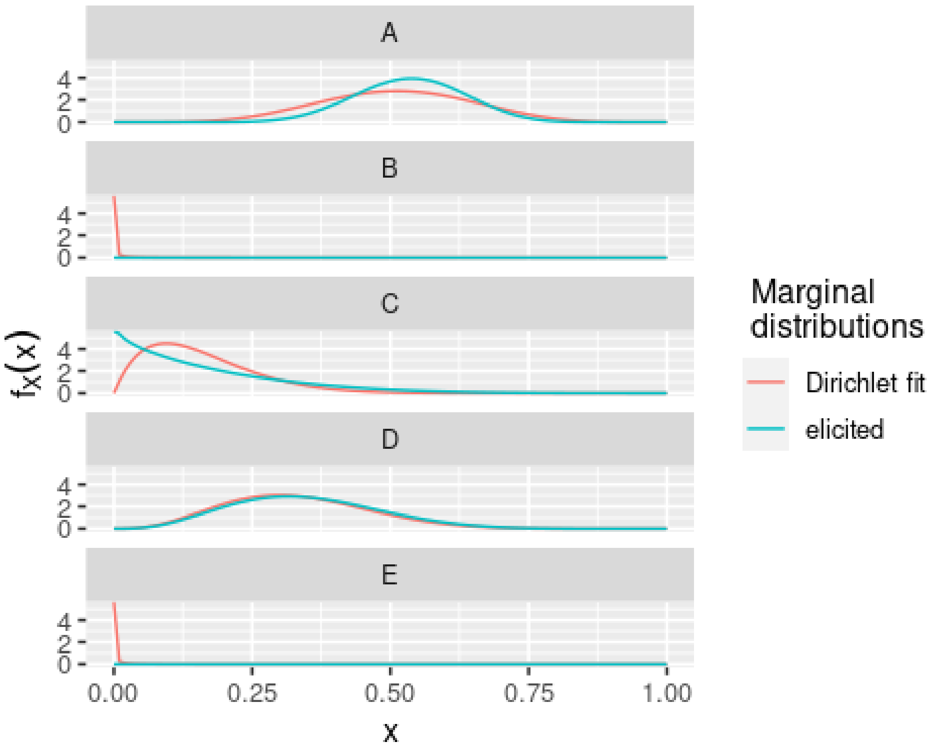


```
##
##        Directly        elicted        beta        marginal        distributions:
##
##                                  A                B          C            D                E
##       shape1      13.3000      1.80e+00      0.920            4.230      1.80e+00
##       shape2      11.5000      7.82e+03      4.630            7.960      7.82e+03
##     mean                      0.5360     2.30e-04     0.166         0.347     2.30e-04
##     sd                          0.0982     1.72e-04     0.145         0.131     1.72e-04
##      sum                      24.8000     7.82e+03      5.550      12.200      7.82e+03
##
##        Sum         of         elicited         marginal         means:         1.048
##
##       Beta        marginal        distributions        from        Dirichlet        fit:
##
##                               A                B              C            D                E
##     shape1         6.530         0.00281        2.0200         4.230         0.00281
##      shape2            6.250       12.80000      10.8000          8.560      12.80000
##    mean                0.511        0.00022        0.1580        0.331        0.00022
##    sd                        0.135        0.00399        0.0983        0.127        0.00399
## sum     12.800 12.80000 12.8000 12.800 12.80000
```

Dipturus nidaroidiensis

16 December 2021, 12:06

Elicited judgements

quantiles	A	B	C	D	E
0.25	0.6497	1e-04	0.0783	0.0830	1e-04
0.50	0.7987	2e-04	0.0830	0.1097	2e-04
0.75	0.9043	3e-04	0.0878	0.1390	3e-04

Dirichlet density function and parameters

Define the vector of unknown population proportions as

$$\theta := (\theta_1, \ldots, \theta_k),$$

with $= 5$. We write

$$\theta \sim Dirichlet(a_1, \ldots, a_k),$$

with

$$f(\theta) = \frac{\Gamma(a_1 + \cdots + a_k)}{\Gamma(a_1) \ldots \Gamma(a_k)} \prod_{i=1}^{k} \theta_i^{a_i - 1}.$$

The fitted parameter values $a_1, \ldots, a_k$ are as follows:

```
##              A            B            C            D            E
## 11.425181379  0.003464764  1.250570175  1.711536486  0.003464764
```

Comparing the elicited marginals with the marginals from the Dirichlet fit

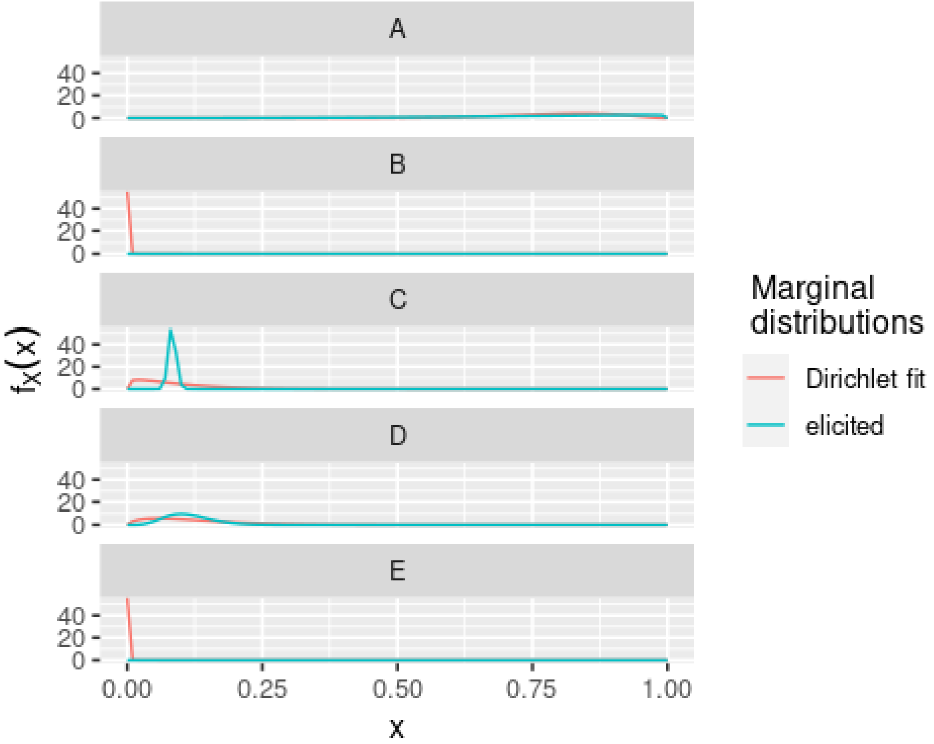

```
##
##         Directly        elicted         beta         marginal         distributions:
##
##                           A                  B                 C             D                 E
##       shape1        3.570        1.80e+00        1.28e+02                6.410        1.80e+00
##        shape2         1.130          7.82e+03        1.41e+03        49.900         7.82e+03
##      mean                       0.760       2.30e-04        8.32e-02           0.114        2.30e-04
##     sd                             0.179       1.72e-04      7.05e-03           0.042        1.72e-04
##      sum                           4.690        7.82e+03       1.53e+03       56.300        7.82e+03
##
##         Sum          of          elicited          marginal          means:            0.957
##
##       Beta        marginal        distributions         from        Dirichlet          fit:
##
##                             A                   B              C             D                 E
##      shape1       11.400       3.46e-03             1.2500             1.7100       3.46e-03
##       shape2                     2.970        1.44e+01        13.1000       12.7000       1.44e+01
##     mean                           0.794      2.41e-04          0.0869          0.1190       2.41e-04
##    sd                                0.103     3.95e-03          0.0718          0.0825       3.95e-03
## sum    14.400 1.44e+01 14.4000 14.4000 1.44e+01
```

Glaucostegus caemiculos

16 December 2021, 12:18

Elicited judgements

quantiles	A	B	C	D	E
0.25	0.2973	1e-04	0.142	0.2533	1e-04
0.50	0.3770	2e-04	0.227	0.3873	2e-04
0.75	0.4617	3e-04	0.384	0.5200	3e-04

Dirichlet density function and parameters

Define the vector of unknown population proportions as

$$\theta := (\theta_1, \ldots, \theta_k),$$

with $= 5$. We write

$$\theta \sim Dirichlet(a_1, \ldots, a_k),$$

with

$$f(\theta) = \frac{\Gamma(a_1 + \cdots + a_k)}{\Gamma(a_1) \ldots \Gamma(a_k)} \prod_{i=1}^{k} \theta_i^{a_i - 1}.$$

The fitted parameter values $a_1, \ldots, a_k$ are as follows:

```
##                  A                  B                  C                  D                  E
## 3.032340883 0.001828659 2.118675219 3.136779691 0.001828659
```

Comparing the elicited marginals with the marginals from the Dirichlet fit

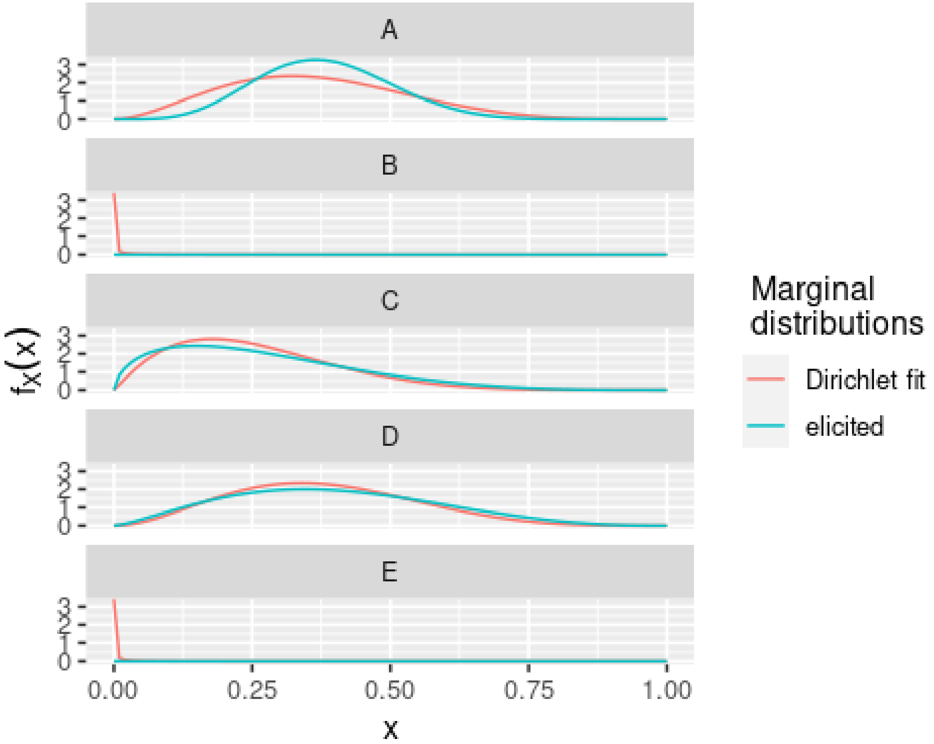


```
##
##        Directly        elicted        beta        marginal        distributions:
##
##                          A              B           C           D              E
##       shape1            6.140       1.80e+00       1.580       2.470       1.80e+00
##       shape2            9.930       7.82e+03       4.350       3.780       7.82e+03
##      mean                     0.382       2.30e-04       0.267       0.395       2.30e-04
##     sd                              0.118       1.72e-04       0.168       0.182       1.72e-04
##      sum                     16.100       7.82e+03       5.930       6.250       7.82e+03
##
##        Sum          of          elicited          marginal          means:          1.045
##
##       Beta        marginal        distributions        from        Dirichlet        fit:
##
##                         A                    B           C           D              E
##        shape1          3.030          0.001830          2.120          3.140          0.001830
##        shape2          5.260          8.290000          6.170          5.150          8.290000
##       mean                     0.366       0.000221       0.256       0.378       0.000221
##      sd                              0.158       0.004870       0.143       0.159       0.004870
## sum    8.290 8.290000 8.290 8.290 8.290000
```

Squatina squatina

16 December 2021, 12:21

Elicited judgements

quantiles	A	B	C	D	E
0.25	0.3907	1e-04	0.0629	0.2407	1e-04
0.50	0.5377	2e-04	0.1680	0.3743	2e-04
0.75	0.6743	3e-04	0.2848	0.5170	3e-04

Dirichlet density function and parameters

Define the vector of unknown population proportions as

$$\theta := (\theta_1, \dots, \theta_k),$$

with $= 5$. We write

$$\theta \sim Dirichlet(a_1, \dots, a_k),$$

with

$$f(\theta) = \frac{\Gamma(a_1 + \cdots + a_k)}{\Gamma(a_1) \dots \Gamma(a_k)} \prod_{i=1}^{k} \theta_i^{a_i - 1}.$$

The fitted parameter values $a_1, \dots, a_k$ are as follows:

```
##           A           B           C           D           E
## 2.632309159 0.001140046 0.994531352 1.912863515 0.001140046
```

Comparing the elicited marginals with the marginals from the Dirichlet fit

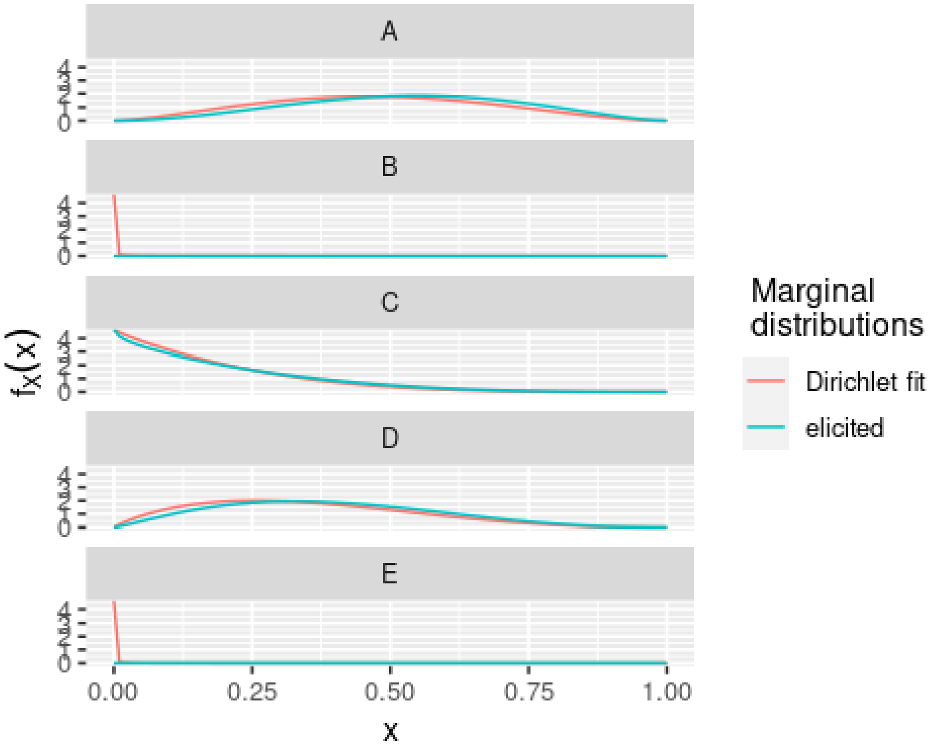

```
##
##        Directly        elicted        beta        marginal        distributions:
##
##                         A                B           C           D            E
##        shape1        3.120       1.80e+00       0.951       2.240     1.80e+00
##        shape2        2.750       7.82e+03       3.780       3.560     7.82e+03
##      mean                    0.532       2.30e-04       0.201       0.387       2.30e-04
##     sd                          0.190       1.72e-04       0.167       0.187      1.72e-04
##     sum                      5.870       7.82e+03       4.730       5.800      7.82e+03
##
##        Sum        of        elicited        marginal        means:        1.12
##
##       Beta       marginal       distributions       from       Dirichlet       fit:
##
##                         A                B           C           D            E
##        shape1        2.630       0.001140       0.995       1.910     0.001140
##        shape2        2.910       5.540000       4.550       3.630     5.540000
##      mean                    0.475       0.000206       0.179       0.345       0.000206
##     sd                          0.195       0.005610       0.150       0.186      0.005610
## sum    5.540 5.540000 5.540 5.540 5.540000
```

Dipturus batis

16 December 2021, 12:25

Elicited judgements

quantiles	A	B	C	D	E
0.25	0.6183	1e-04	0.1013	0.0830	1e-04
0.50	0.7410	2e-04	0.1363	0.1097	2e-04
0.75	0.8407	3e-04	0.1865	0.1390	3e-04

Dirichlet density function and parameters

Define the vector of unknown population proportions as

$$\theta := (\theta_1, \ldots, \theta_k),$$

with $= 5$. We write

$$\theta \sim Dirichlet(a_1, \ldots, a_k),$$

with

$$f(\theta) = \frac{\Gamma(a_1 + \cdots + a_k)}{\Gamma(a_1) \ldots \Gamma(a_k)} \prod_{i=1}^{k} \theta_i^{a_i - 1}.$$

The fitted parameter values $a_1, \ldots, a_k$ are as follows:

```
##                  A                B                C                D                E
## 11.291863752  0.003615452  2.296177735  1.785974133  0.003615452
```

Comparing the elicited marginals with the marginals from the Dirichlet fit

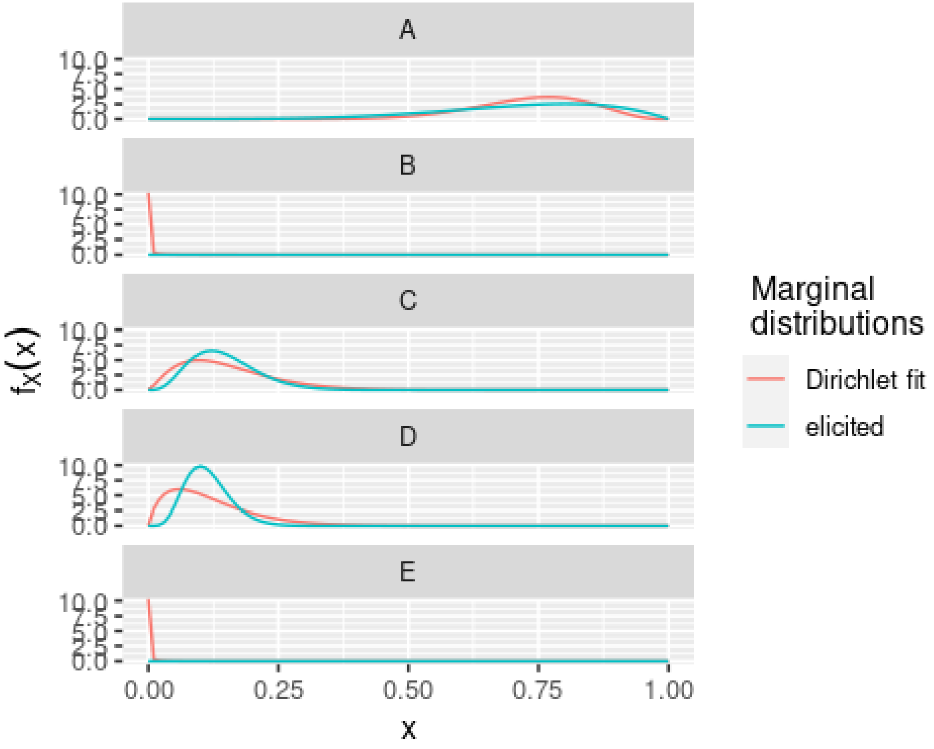


```
##
##        Directly       elicted       beta       marginal       distributions:
##
##                          A                B              C            D            E
##      shape1      5.240      1.80e+00            4.4300          6.410      1.80e+00
##        shape2        2.040       7.82e+03      25.9000      49.900      7.82e+03
##     mean                 0.720     2.30e-04        0.1460          0.114     2.30e-04
##     sd                       0.156     1.72e-04        0.0632          0.042     1.72e-04
##      sum                       7.290      7.82e+03      30.3000      56.300      7.82e+03
##
##        Sum         of         elicited         marginal         means:         0.98
##
##       Beta        marginal       distributions        from        Dirichlet        fit:
##
##                           A                B            C             D             E
##      shape1      11.300       3.62e-03            2.300          1.7900      3.62e-03
##       shape2              4.090       1.54e+01       13.100      13.6000      1.54e+01
##     mean                    0.734      2.35e-04        0.149          0.1160      2.35e-04
##     sd                          0.109      3.79e-03        0.088          0.0792      3.79e-03
## sum     15.400 1.54e+01 15.400 15.4000 1.54e+01
```

Pristis pectinata

December 2021, 12:28

Elicited judgements

quantiles	A	B	C	D	E
0.25	0.1336	1e-04	0.1900	0.2760	1e-04
0.50	0.1639	2e-04	0.3160	0.4210	2e-04
0.75	0.2002	3e-04	0.4363	0.5187	3e-04

Dirichlet density function and parameters

Define the vector of unknown population proportions as

$$\theta := (\theta_1, \ldots, \theta_k),$$

with $= 5$. We write

$$\theta \sim Dirichlet(a_1, \ldots, a_k),$$

with

$$f(\theta) = \frac{\Gamma(a_1 + \cdots + a_k)}{\Gamma(a_1) \ldots \Gamma(a_k)} \prod_{i=1}^{k} \theta_i^{a_i - 1}.$$

The fitted parameter values $a_1, \ldots, a_k$ are as follows:

```
##           A           B           C           D           E
## 1.834741013 0.002509129 3.565815580 4.499536539 0.002509129
```

Comparing the elicited marginals with the marginals from the Dirichlet fit

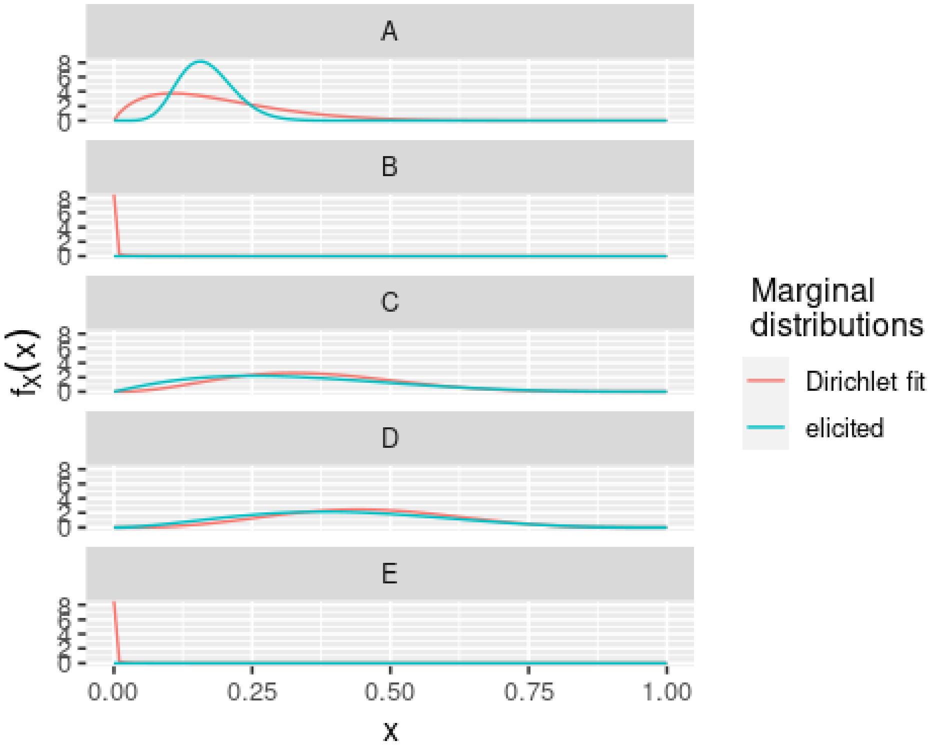

```
##
##        Directly          elicted          beta          marginal          distributions:
##
##                                A                 B           C           D               E
##      shape1               9.5600        1.80e+00      2.130       3.040        1.80e+00
##       shape2         47.2000        7.82e+03        4.380        4.320        7.82e+03
##     mean                          0.1680       2.30e-04       0.327       0.413        2.30e-04
##    sd                                0.0493       1.72e-04      0.171       0.170        1.72e-04
##     sum                          56.7000       7.82e+03       6.510       7.360        7.82e+03
##
##        Sum          of          elicited          marginal          means:          0.91
##
##       Beta        marginal        distributions          from        Dirichlet          fit:
##
##                             A                  B           C           D               E
##         shape1         1.830         0.002510        3.570        4.500         0.002510
##         shape2         8.070         9.900000        6.340        5.410         9.900000
##       mean                       0.185         0.000253        0.360       0.454         0.000253
##      sd                              0.118        0.004820       0.145       0.151         0.004820
## sum     9.910 9.910000 9.910 9.910 9.910000
```

Tetronarce nobiliana

16 December 2021, 12:32

Elicited judgements

quantiles	A	B	C	D	E
0.25	0.522	0.0490	0.0873	0.1185	1e-04
0.50	0.638	0.0517	0.1297	0.1570	2e-04
0.75	0.743	0.0547	0.1872	0.1995	3e-04

Dirichlet density function and parameters

Define the vector of unknown population proportions as

$$\theta := (\theta_1, \ldots, \theta_k),$$

with $= 5$. We write

$$\theta \sim Dirichlet(a_1, \ldots, a_k),$$

with

$$f(\theta) = \frac{\Gamma(a_1 + \cdots + a_k)}{\Gamma(a_1) \ldots \Gamma(a_k)} \prod_{i=1}^{k} \theta_i^{a_i - 1}.$$

The fitted parameter values $a_1, \ldots, a_k$ are as follows:

```
##                  A                B                C                D                E
## 11.589583503  0.957616427  2.627447003  3.003559735  0.004254159
```

Comparing the elicited marginals with the marginals from the Dirichlet fit

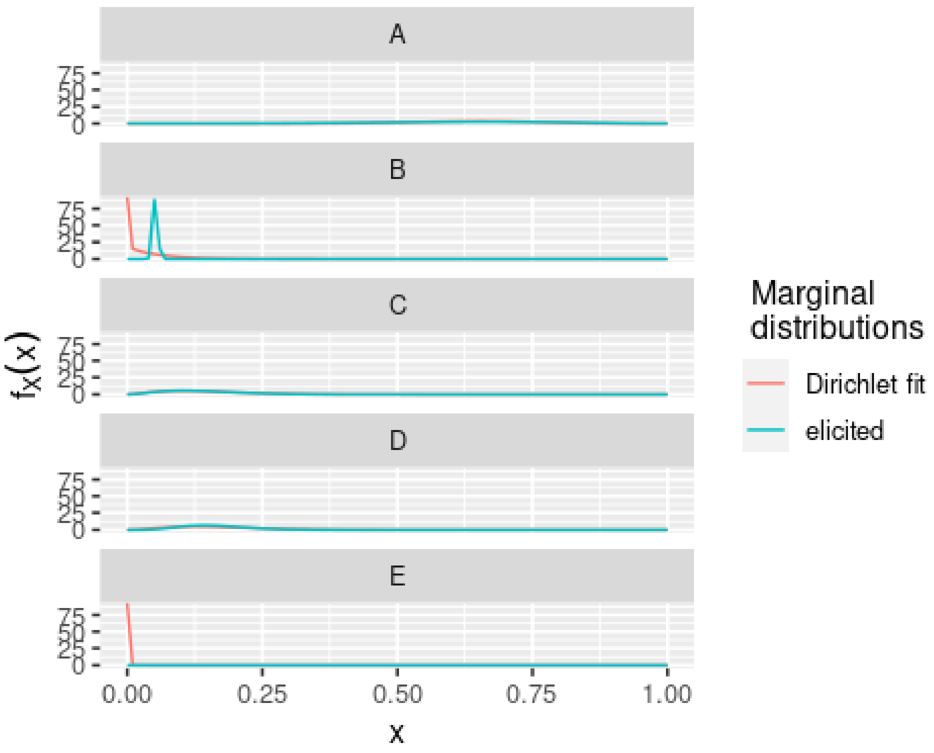


```
##
##        Directly        elicted        beta        marginal        distributions:
##
##                         A                B              C              D                E
##     shape1      5.560     1.43e+02           2.9900         5.9600     1.80e+00
##       shape2       3.300        2.61e+03      18.0000     30.7000     7.82e+03
##    mean                  0.628      5.19e-02        0.1420          0.1630     2.30e-04
##    sd                        0.154     4.23e-03        0.0745          0.0601     1.72e-04
##     sum                         8.850      2.75e+03      21.0000      36.7000     7.82e+03
##
##        Sum          of          elicited          marginal          means:          0.985
##
##       Beta        marginal        distributions        from        Dirichlet        fit:
##
##                            A                B              C              D                E
##     shape1     11.600            0.9580           2.6300          3.0000     4.25e-03
##       shape2                  6.590       17.2000      15.6000     15.2000     1.82e+01
##    mean                       0.637        0.0527         0.1450          0.1650     2.34e-04
##    sd                             0.110           0.0510         0.0803          0.0848     3.49e-03
## sum     18.200 18.2000 18.2000 18.2000 1.82e+01
```
