# Supplementary material for: Exploring fishing threat at fleet segment and subregional scale: Least expert knowledge and a resilience versus disturbance‐based approach as conservation's tools for cartilaginous fish
Source: Ecol Evol. 2023 Mar 19;13(3):e9881. doi: 10.1002/ece3.9881 (PMC10025082; doi:10.1002/ece3.9881)
Supplement: Supplementary file 4 — Data S4. [file ECE3-13-e9881-s004.pdf]

S4

S2. Basic data. Intrinsic vulnerability to Fishing (***IVF_X***, Cheung et al.2005; data acquisition www.fishbase.de, 2015-16) and corresponding estimates, as median (Med), upper (75) and lower (25) quartiles, of probability of fatal catch from aggregated judgments of three profiled and selected shark experts for ***X*** =76 cartilaginous species (alphabetically ordered) censed at Mediterranean scale and by five representative fishing fleet segments I (BT: bottom trawls; PL: pelagic longlines; PPG: passive polyvalent gears; SSF: small-scale fishery; PTP: pelagic pair trawl and purse seine). Corresponding qualitative species-specific risk scores ***QS_X*** were reported as well.

Fishing Segment		BT				PL				PPG				SSF				PTP			
**Species**	**IVF_X**	**Q_{25}**	**M**	**Q_{75}**	**QS_X**	**Q_{25}**	**M**	**Q_{75}**	**QS_X**	**Q_{25}**	**M**	**Q_{75}**	**QS_X**	**Q_{25}**	**M**	**Q_{75}**	**QS_X**	**Q_{25}**	**M**	**Q_{75}**	**QS_X**
Alopias superciliosus	79	0.0	0.0	0.0	0	33.1	78.4	91.8	1	3.2	3.6	4.0	0.75	1.6	1.8	2.0	0.75	1.8	8.9	25.3	0.75
Alopias vulpinus	68	0.3	1.1	2.6	0.75	32.4	74.9	91.0	0.5	3.2	3.6	4.0	0.75	1.6	1.8	2.0	0.25	2.0	10.1	28.7	0.25
Carcharhinus altimus	76	0.3	1.1	2.6	0.75	18.8	62.3	66.5	0.75	16.4	34.2	35.1	0.75	0.0	0.0	0.0	0	0.0	0.0	0.0	0
Carcharhinus brachyurus	87	1.9	2.9	4.7	0.75	18.3	50.9	66.3	0.75	17.2	27.0	42.4	1	9.6	13.5	18.3	0.75	0.3	3.6	22.3	0.75
Carcharhinus brevipinna	62	3.9	7.0	11.5	0.25	18.3	55.6	66.6	0.25	15.5	19.0	22.8	0.25	6.3	10.4	15.7	0.25	2.9	5.7	10.5	0.25
Carcharhinus falciformis	79	0.3	1.1	2.6	0.75	26.0	74.1	87.1	1	15.0	19.1	23.4	0.75	2.6	4.1	6.1	0.75	0.0	0.0	0.0	0
Carcharhinus limbatus	55	3.9	7.0	11.5	0.25	22.2	56.7	82.0	0.5	16.4	19.8	23.4	0.25	9.0	15.0	22.8	0.25	0.0	0.0	0.0	0
Carcharhinus obscurus	88	1.6	3.6	7.0	0.75	24.0	62.5	83.9	1	15.7	25.0	41.6	1	7.4	11.2	15.9	0.75	0.0	0.0	0.0	0
Carcharhinus plumbeus	88	20.4	27.5	35.8	0.75	7.0	27.2	55.5	0.75	14.8	28.3	43.1	1	15.0	26.8	39.8	0.75	0.0	0.0	0.0	0
Carcharias taurus	58	16.5	23.4	31.5	0.25	6.5	16.8	32.6	0.25	17.9	21.4	24.9	0.25	17.3	29.6	42.3	0.25	2.7	4.6	7.1	0.25
Carcharodon carcharias	86	12.0	15.9	20.4	0.75	25.5	60.9	86.3	1	5.3	7.3	9.9	0.75	3.7	5.2	7.3	0.75	1.3	1.5	1.8	0.75
Centrophorus uyato	87	63.0	77.3	88.6	1	0.0	0.0	0.0	0	14.0	19.7	28.3	0.75	1.6	1.8	2.0	0.75	0.0	0.0	0.0	0
Centrosymnus coelolepis	63	67.3	81.8	91.8	0.5	0.0	0.0	0.0	0	11.3	15.9	23.6	0.25	0.0	0.0	0.0	0	0.0	0.0	0.0	0
Cetorhinus maximus	86	10.3	15.4	21.9	0.75	0.0	0.0	0.0	0	11.6	20.0	34.2	0.75	34.8	53.4	69.5	1	1.6	8.3	28.2	0.75
Chimaera monstrosa	74	77.9	94.5	99.5	1	0.0	0.0	0.0	0	1.3	1.5	1.8	0.75	0.0	0.0	0.0	0	0.0	0.0	0.0	0
Dalatias licha	81	47.4	52.7	58.1	1	1.5	2.3	3.4	0.75	18.7	35.2	54.5	1	6.9	9.4	12.2	0.75	0.0	0.0	0.0	0
Bathytoshia lata	81	39.8	44.4	49.6	0.75	0.0	0.0	0.0	0	19.4	35.3	52.9	1	14.6	19.6	25.2	0.75	0.0	0.0	0.0	0
Dasyatis pastinaca	82	42.6	49.1	55.5	0.75	0.0	0.0	0.0	0	11.3	25.2	42.4	1	16.0	21.2	26.9	0.75	0.0	0.0	0.0	0
Dipturus batis	86	61.8	74.1	84.1	1	0.0	0.0	0.0	0	10.1	13.6	18.7	0.75	8.3	11.0	13.9	0.75	0.0	0.0	0.0	0
Dipturus nidaroidiensis	85	65.0	79.9	90.4	1	0.0	0.0	0.0	0	7.8	8.3	8.8	0.75	8.3	11.0	13.9	0.75	0.0	0.0	0.0	0
Dipturus oxyrinchus	77	65.0	79.9	90.4	1	0.0	0.0	0.0	0	7.8	8.3	8.8	0.75	8.3	11.0	13.9	0.75	0.0	0.0	0.0	0

Echinorhinus brucus	84	63.1	70.5	73.7	1	0.3	1.1	2.6	0.75	12.4	18.6	26.5	0.75	2.1	3.4	5.3	0.75	0.0	0.0	0.0	0
Etmopterus spinax	47	71.1	85.6	90.3	0.5	0.0	0.0	0.0	0	8.5	11.8	16.6	0.25	0.0	0.0	0.0	0	0.0	0.0	0.0	0
Galeorhinus galeus	74	42.5	53.1	63.3	1	4.5	11.3	19.9	0.75	18.0	30.4	51.5	1	8.1	12.0	16.8	0.75	0.0	0.0	0.0	0
Galeus melastomus	60	75.1	90.6	93.4	0.5	0.0	0.0	0.0	0	2.3	5.3	9.9	0.25	0.0	0.0	0.0	0	0.0	0.0	0.0	0
Gymnura altavela	51	23.8	35.6	47.1	0.25	0.0	0.0	0.0	0	5.5	11.8	25.0	0.25	14.8	20.2	26.3	0.25	13.3	26.2	37.0	0.50
Heptranchias perlo	73	58.2	64.6	69.9	1	4.5	11.3	19.9	0.75	14.9	21.6	29.5	0.75	0.0	0.0	0.0	0	0.0	0.0	0.0	0
Hexanchus griseus	84	30.8	38.1	45.8	0.75	4.9	5.2	5.5	0.75	23.7	31.4	39.8	1	5.0	7.9	11.4	0.75	2.7	4.6	7.1	0.75
Hexanchus nakamurrai	81	48.1	57.6	65.8	1	0.0	0.0	0.0	0	22.3	30.8	39.9	1	3.1	4.1	5.2	0.75	0.0	0.0	0.0	0
Isurus oxyrinchus	76	6.7	9.5	13.0	0.75	26.8	65.5	86.0	1	5.3	7.3	9.9	0.75	3.7	5.2	7.3	0.75	0.0	2.5	18.9	0.75
Lamna nasus	86	2.9	5.2	8.8	0.75	26.9	67.0	87.7	1	11.7	15.1	18.6	0.75	2.9	3.3	3.8	0.75	1.3	4.0	20.7	0.75
Leucoraja circularis	67	64.0	79.2	90.2	1	0.0	0.0	0.0	0	7.8	8.3	8.8	0.75	8.7	11.6	14.8	0.75	0.0	0.0	0.0	0
Leucoraja fullonica	67	60.8	73.5	83.8	1	0.0	0.0	0.0	0	3.2	3.6	4.0	0.75	13.0	18.0	23.9	0.75	0.0	0.0	0.0	0
Leucoraja melitensis	44	48.3	60.0	70.9	0.5	0.0	0.0	0.0	0	8.5	18.5	28.4	0.25	18.3	25.6	34.0	0.25	0.0	0.0	0.0	0
Leucoraja naevus	62	60.8	73.5	83.8	0.5	0.0	0.0	0.0	0	3.2	3.6	4.0	0.25	13.0	18.0	23.9	0.25	0.0	0.0	0.0	0
Mobula mobular	55	19.1	26.1	34.4	0.25	6.4	7.5	8.9	0.25	16.4	19.8	23.4	0.25	6.3	10.4	15.7	0.25	10.9	25.5	40.1	0.50
Mustelus asterias	70	20.8	27.7	35.7	0.75	4.9	5.2	5.5	0.25	14.9	17.4	20.2	0.75	18.7	32.0	47.0	0.25	5.3	14.9	28.9	0.25
Mustelus mustelus	74	20.8	27.7	35.7	0.75	4.9	5.2	5.5	0.75	14.9	17.4	20.2	0.75	18.7	32.0	47.0	0.75	5.3	14.9	28.9	0.75
Mustelus punctulatus	54	20.8	27.7	35.7	0.25	4.9	5.2	5.5	0.25	14.9	17.4	20.2	0.25	18.7	32.0	47.0	0.25	5.3	14.9	28.9	0.25
Myliobatis aquila	59	21.2	26.8	33.0	0.25	4.9	5.2	5.5	0.25	5.5	11.8	25.0	0.25	28.5	40.7	44.8	0.50	8.4	16.0	28.5	0.75
Odontaspis ferox	76	39.9	48.1	56.2	1	7.2	18.1	32.9	0.75	17.3	24.0	31.8	0.75	4.4	6.6	9.5	0.75	0.0	0.0	0.0	0
Oxynotus centrina	80	66.3	76.3	80.1	1	0.0	0.0	0.0	0	10.1	13.3	16.6	0.75	4.9	5.2	5.5	0.75	0.0	0.0	0.0	0
Prionace glauca	77	1.3	1.5	1.8	0.75	28.6	82.5	92.0	1	1.6	1.8	2.0	0.75	1.3	1.5	1.8	0.75	1.8	8.9	25.3	0.75
Pristis pectinata	86	13.4	16.4	20.0	0.75	0.0	0.0	0.0	0	19.0	31.6	43.6	1	27.6	42.1	51.9	1	0.0	0.0	0.0	0

Pristis pristis	75	13.4	16.4	20.0	0.75	0.0	0.0	0.0	0	19.0	31.6	43.6	1	27.6	42.1	51.9	1	0.0	0.0	0.0	0
Aetomylaeus bovinus	56	11.0	16.4	22.9	0.25	4.9	5.2	5.5	0.25	5.5	11.8	25.0	0.25	28.5	40.7	44.8	0.50	12.5	22.4	33.0	1.00
Pteroplatytrygon violacea	66	6.5	9.1	12.3	0.75	42.1	49.0	56.2	0.5	2.9	3.3	3.8	0.25	18.2	26.3	33.2	0.75	7.6	12.2	31.5	0.75
Rostroraja alba	84	46.5	54.1	60.0	1	0.0	0.0	0.0	0	5.5	11.8	25.0	0.75	24.4	34.8	42.6	0.75	0.0	0.0	0.0	0
Raja asterias	50	44.1	53.4	61.4	0.5	0.0	0.0	0.0	0	1.6	1.8	2.0	0.25	27.6	43.6	57.0	0.50	0.0	0.0	0.0	0
Raja brachyura	59	29.8	38.5	47.5	0.25	0.0	0.0	0.0	0	6.9	16.7	26.4	0.25	34.7	52.9	68.3	0.50	0.0	0.0	0.0	0
Raja clavata	72	63.2	78.2	89.1	1	0.0	0.0	0.0	0	4.8	5.8	6.8	0.75	21.5	30.2	36.2	0.75	0.0	0.0	0.0	0
Raja miralaetus	51	50.9	66.5	79.7	0.5	0.0	0.0	0.0	0	1.6	1.8	2.0	0.25	29.4	45.3	58.2	0.50	0.0	0.0	0.0	0
Raja montagui	59	53.3	59.6	64.8	0.5	0.0	0.0	0.0	0	1.6	1.8	2.0	0.25	22.6	32.5	40.1	0.25	0.0	0.0	0.0	0
Raja polystigma	45	33.1	41.5	50.0	0.25	0.0	0.0	0.0	0	6.9	16.7	26.4	0.25	31.1	49.4	66.4	0.50	0.0	0.0	0.0	0
Raja radula	50	23.0	31.1	39.9	0.25	0.0	0.0	0.0	0	6.9	16.7	26.4	0.25	36.2	54.7	69.8	0.50	0.0	0.0	0.0	0
Raja undulata	65	35.6	44.6	52.9	0.25	0.0	0.0	0.0	0	1.6	1.8	2.0	0.25	30.9	47.1	59.6	0.50	0.0	0.0	0.0	0
Glaucostegus caemiculos	85	29.7	37.7	46.2	0.75	0.0	0.0	0.0	0	14.2	22.7	38.4	0.75	25.3	38.7	52.0	1	0.0	0.0	0.0	0
Rhinobatos rhinobatos	68	26.8	33.8	41.3	0.75	0.0	0.0	0.0	0	14.2	22.7	38.4	0.75	27.8	42.6	55.3	1	0.0	0.0	0.0	0
Rhinoptera marginata	45	25.8	33.0	40.8	0.25	0.0	0.0	0.0	0	9.2	17.3	32.9	0.25	21.1	30.8	39.6	0.25	9.9	18.3	29.8	0.25
Rhizoprionodon acutus	61	43.7	54.0	60.4	0.5	0.8	10.0	27.7	0.25	10.1	13.3	16.6	0.25	14.6	25.0	36.1	0.25	0.0	0.0	0.0	0
Scyliorhinus canicula	62	52.5	67.8	80.8	0.5	0.0	0.0	0.0	0	5.5	8.9	15.0	0.25	24.2	34.8	43.3	0.25	0.0	0.0	0.0	0
Scyliorhinus stellaris	83	34.4	47.8	60.3	1	0.0	0.0	0.0	0	5.5	11.8	25.0	0.75	26.7	40.6	53.6	1	0.0	0.0	0.0	0
Somniosus rostratus	76	49.8	56.7	61.6	1	7.2	33.2	33.3	0.75	6.9	9.5	14.9	0.75	0.0	0.0	0.0	0.00	0.0	0.0	0.0	0
Sphyrna lewini	81	0.3	1.1	2.6	0.75	30.7	80.7	92.2	1	7.5	8.0	8.5	0.75	1.3	1.5	1.8	0.75	0.0	2.5	18.9	0.75
Sphyrna mokarran	86	0.3	1.1	2.6	0.75	30.7	80.7	92.2	1	7.5	8.0	8.5	0.75	1.3	1.5	1.8	0.75	0.0	2.5	18.9	0.75
Sphyrna tudes	59	0.3	1.1	2.6	0.25	30.7	80.7	92.2	0.5	7.5	8.0	8.5	0.25	1.3	1.5	1.8	0.25	0.0	2.5	18.9	0.25
Sphyrna zigaena	85	0.3	1.1	2.6	0.75	30.7	80.7	92.2	1	7.5	8.0	8.5	0.75	1.3	1.5	1.8	0.75	0.0	2.5	18.9	0.75

Squalus acanthias	68	19.9	27.0	35.1	0.75	7.2	18.1	32.9	0.25	9.1	11.2	13.8	0.75	12.1	23.2	36.3	0.25	10.2	20.0	34.4	0.50
Squalus blainvillei	67	19.9	27.0	35.1	0.25	6.5	16.8	32.6	0.25	14.9	17.4	20.2	0.25	15.7	26.9	40.0	0.25	5.3	14.9	28.9	0.25
Squatina aculeata	75	39.1	53.8	67.4	1	0.0	0.0	0.0	0	8.5	18.5	28.4	0.75	23.3	36.6	50.9	1	0.0	0.0	0.0	0
Squatina oculata	73	39.1	53.8	67.4	1	0.0	0.0	0.0	0	8.5	18.5	28.4	0.75	23.3	36.6	50.9	1	0.0	0.0	0.0	0
Squatina squatina	85	39.1	53.8	67.4	1	0.0	0.0	0.0	0	6.3	16.8	28.5	0.75	24.1	37.4	51.7	1	0.0	0.0	0.0	0
Taeniura grabata	79	52.2	63.8	74.3	1	0.0	0.0	0.0	0	10.1	13.6	18.7	0.75	13.7	19.5	26.1	0.75	0.0	0.0	0.0	0
Torpedo marmorata	69	50.9	62.2	72.6	1	0.0	0.0	0.0	0	7.1	13.6	27.0	0.75	17.5	24.8	33.4	0.75	0.0	0.0	0.0	0
Torpedo torpedo	65	43.3	57.6	70.7	0.5	2.1	3.4	5.3	0.25	6.4	7.6	8.9	0.25	19.5	33.4	49.3	0.25	0.0	0.0	0.0	0
Tetronarce nobiliana	87	52.2	63.8	74.3	1	4.9	5.2	5.5	0.75	8.7	13.0	18.7	0.75	11.9	15.7	19.9	0.75	0.0	0.0	0.0	0
