# Supplementary material for: Exploring fishing threat at fleet segment and subregional scale: Least expert knowledge and a resilience versus disturbance‐based approach as conservation's tools for cartilaginous fish
Source: Ecol Evol. 2023 Mar 19;13(3):e9881. doi: 10.1002/ece3.9881 (PMC10025082; doi:10.1002/ece3.9881)
Supplement: Supplementary file 5 — Data S5. [file ECE3-13-e9881-s007.pdf]

S5

The panels A and B below report the distributions of DRB_{IX}, $IERF_X$ and IFR_X grouped by IUCN extinction risk categories at Italian (panel A) and Mediterranean (B) scale, for X=76 Mediterranean cartilaginous species and I= five selected fishing fleet segments (BT: bottom trawls; PL: pelagic longlines; PPG: passive polyvalent gears; SSF: small-scale fishery; PTP: pelagic pair trawl and purse seine). DRB_{IX} is Disturbance Resilience Balance by fishing fleet segments I and species X; $IERF_X$ and IFR_X are Indexes of Extinction Risk to Fishing and Overfishing Response by species X.

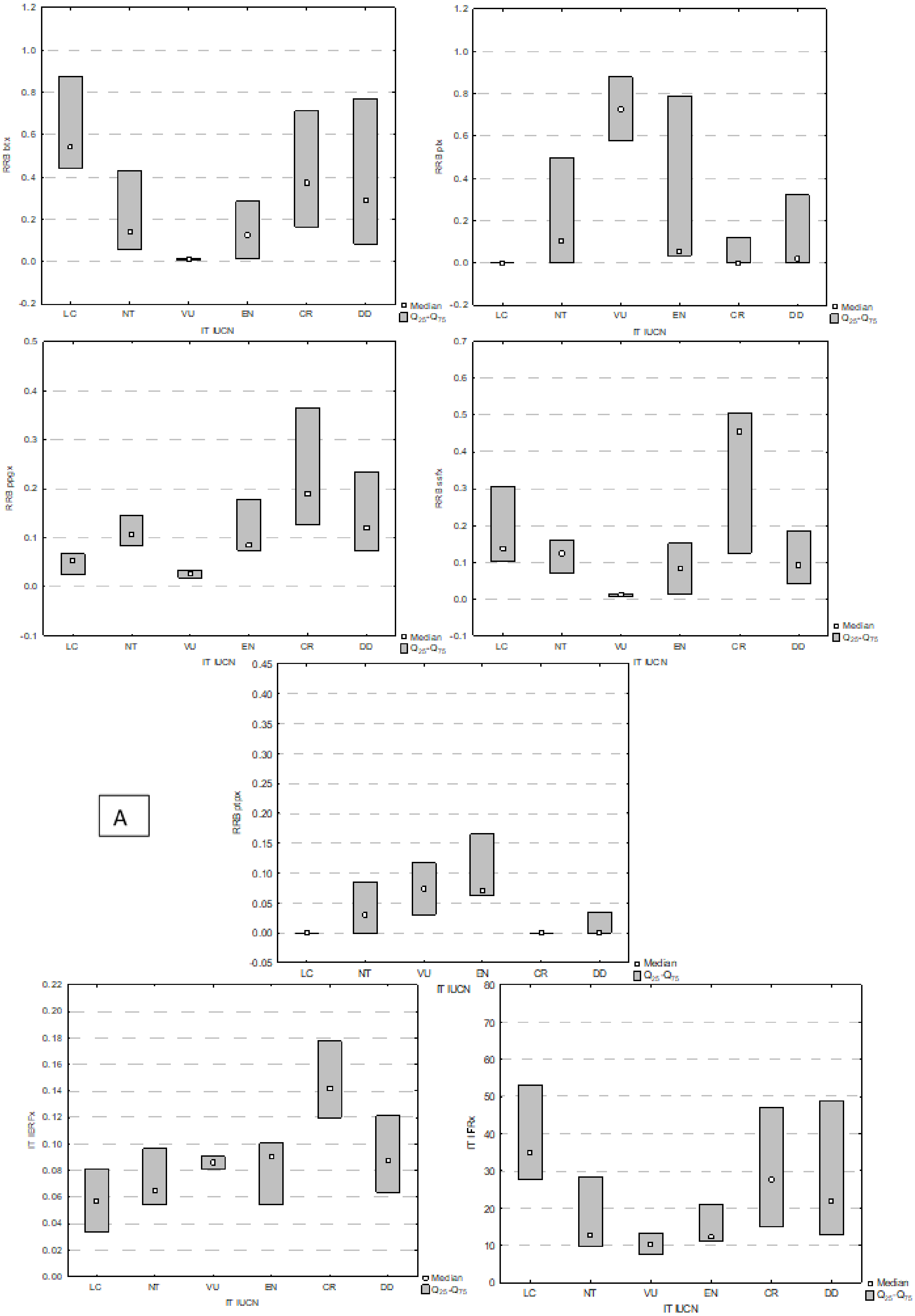

A
IT IUCN
LC
NT
VU
EN
CR
DD
Median
Q25-Q75

B

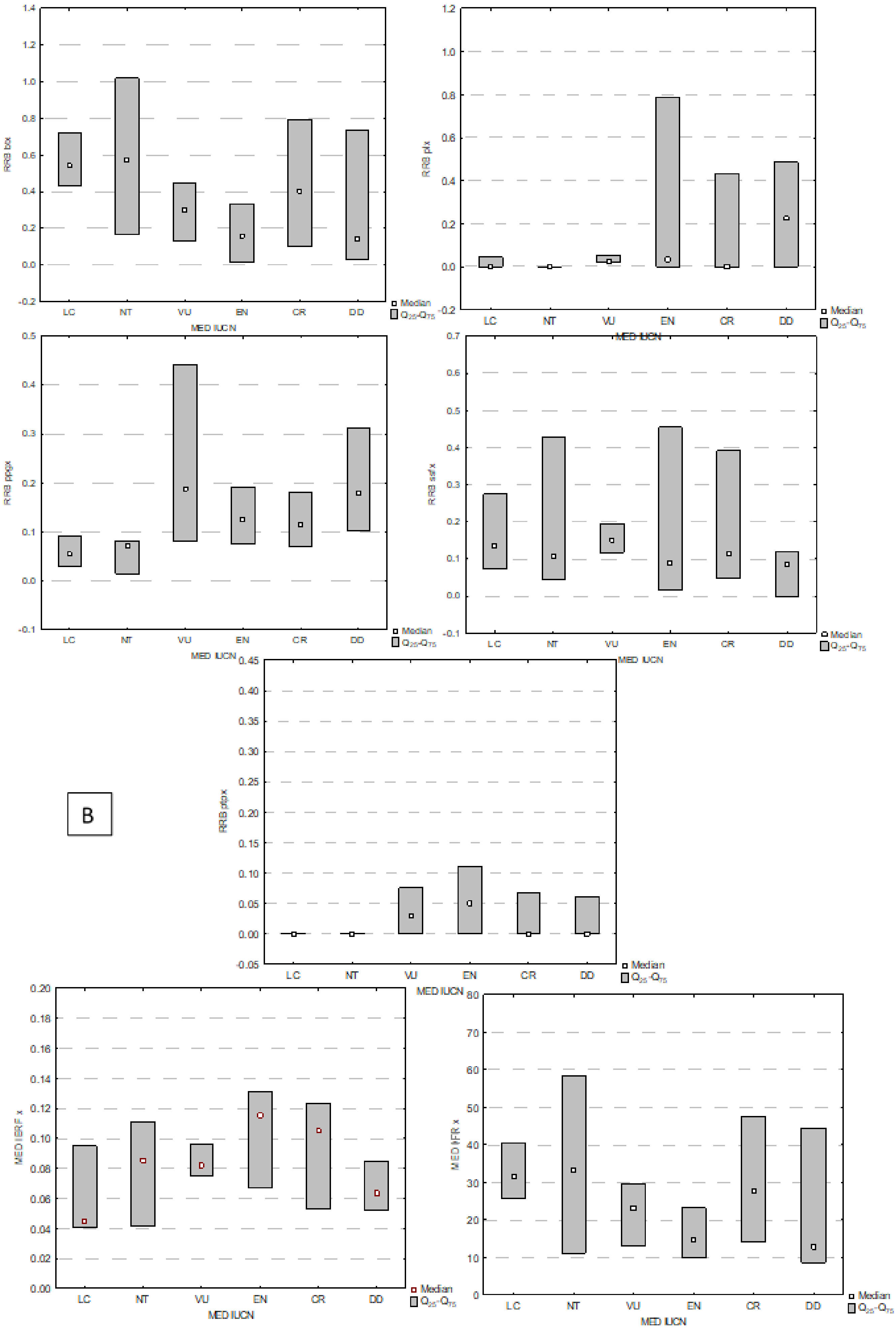
RRB box
RRB plx
RRB ppgx
RRB ssfx
RRB pfpx
MED IERF x
MED IFR x
LC
NT
VU
EN
CR
DD
MED IUCN
Median
Q_{25}-Q_{75}
