# Supplementary material for: Exploring fishing threat at fleet segment and subregional scale: Least expert knowledge and a resilience versus disturbance‐based approach as conservation's tools for cartilaginous fish
Source: Ecol Evol. 2023 Mar 19;13(3):e9881. doi: 10.1002/ece3.9881 (PMC10025082; doi:10.1002/ece3.9881)
Supplement: Supplementary file 6 — Appendix S1. [file ECE3-13-e9881-s003.docx]

**APPENDICES**

**APPENDIX A1. Suggested communication formats**

*Invitation e-mail*

Dear XX,

We are conducting an expert knowledge’s elicitation experiment, and you have been selected as one of three potential out of ten shark experts that we profiled, who could be randomly chosen to participate in the experiment....

The project consists briefly in making a risk assessment to fisheries for Mediterranean cartilaginous fauna through expert knowledge elicitation.

We would like to know if you are interested to participate anonymously*.

In case of affirmative response, we will let you know if you are selected for the experiment and confirm your participation.

Thank you,

Best

XX

* ^We will not share your identity and the data you eventually provide with anyone without your explicit permission. You will be acknowledged anonymously in any potential published work related to this experiment^.

*Participation e-mail*

Dear XX,

Thank you very much for joining us in this experiment! Attached to this message you will find the context, question for elicitation and information you need to perform your precious and anonymous* evaluations.

We kindly ask you to take an exact week to evaluate the question and provide us with your evaluations. Please communicate your starting time.

For any question, feel free to contact us at any time

Thank you very much

Best

XX

*We will not share your identity and the data you provide with anyone without your explicit permission. You will be acknowledged anonymously in any potential published work related to this experiment.

**APPENDIX A2**

**Contextualization and question to experts**

*Context*

Imagine that you are at sea on a boat, with no precise information about the geographic area (only cardinal points) where you are in the Mediterranean Sea, and consider that all the species studied (see att) are present in your observation area, distributed according to your knowledge about their general ecological attributes (shallow or deep species, benthic, demersal, pelagic). You are observing five boats belonging to five fishing segments and conducting their corresponding fisheries simultaneously. You only know that looking north of your position you will see boats belonging to fishing fleet segments authorized to fish in offshore waters (greater depth and distance from shore); looking south, you will see boats authorized to fish in inshore waters (less depth and distance from shore). You are informed about the fishing gear equipped on each boat according to the fishing fleet segment (see att) and you have to consider that boats with multiple gears fish simultaneously with all the gears they are equipped with.

*Question*

**According to your expert knowledge, which are the disaggregated average probabilities (please use 5% as scale unit in the range 0≤x≤100%) that an individual of each the 76 species considered can have to be fatally caught by each of the five boats, setting at 100 the sum of these probabilities?**

**APPENDIX A3.**

**General Scheme of the formal elicitation method used in the present experiment**


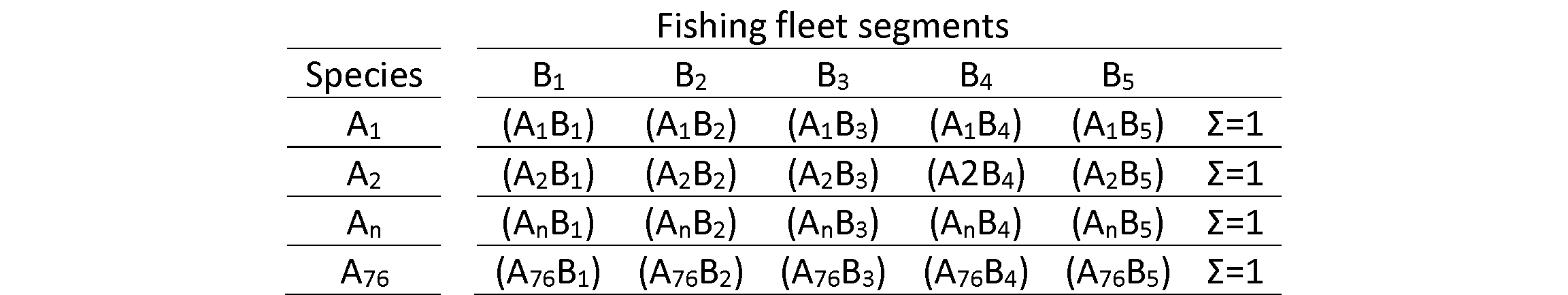


**(A_n_B_n_)** **ARE ESTIMATES BY AN EXPERT AND ASSUMED TO BE FIVE SINGLETONS WITHIN EACH ROW (SPECIES=A_n_) ALONG COLUMNS (SEGMENTS=B_n_)**

**QUARTILES**

**ASSIGNING LOWER AND UPPER BOUNDS TO BEST SINGLE ESTIMATES (A_n_B_n_), BASED ON FOUR QUARTILES OF THE CORRESPONDING BETA-FITTED MOTHER DISTRIBUTIONS B_n_(A_1►76_)**

**QUARTILES**

**OBTAINING EXPERT’S PRIOR BETA DISTRIBUTIONS FROM BOUNDED BEST ESTIMATES FOR EACH SPECIES WITHIN EACH OF THE CONSIDERED SEGMENTS**

**B_3_ (A_1►76_)**

**B_4_ (A_1►76_)**

**B_5_ (A_1►76_)**

**B_1_ (A_1►76_)**

**B_2_ (A_1►76_)**

**AGGREGATING EXPERTS’ PRIOR BETA DISTRIBUTIONS BY SPECIES AND SEGMENTS BY A LINEAR COMBINATION OF THE CORRESPONDING EXTRAPOLATED INTERQUARTILES (I, II AND III QUARTILES)**

**DIRICHLET FIT FOR THE COMBINED DISTRIBUTIONS BY SPECIES AND SEGMENT**

The figure above illustrates the overall workflow we followed to develop the elicitation method. In particular, information is provided as far the basic assumption made about singletons representing estimates by an expert when eliciting multiple uncertain quantities the sum to 1; secondly, about the method (using mother distributions) we elaborated to measure uncertainty around best estimates; thirdly, on the subsequent steps to finalize the elicitation framework according to methods already provided by leading protocols with some adjustments (see methods in the main text).

**APPENDIX B1.**

**Inter-experts agreement**

**A**ggregated results of Friedman ANOVAs on agreement between three experts about estimated probability of fatal catch of 76 Mediterranean cartilaginous species divided in two species groups: (A: Hexanchiformes, Lamniformes, Carcharhiniformes and Squaliformes; B: Squatiniformes, Rajiformes, Pristiformes and Chimaeriformes) and by five representative fishing fleet segments (BT: bottom trawls; PL: pelagic longlines; PPG: passive polyvalent gears; SSF: small-scale fishery; PTP: pelagic pair trawl and purse seine). Analyses were run by fishing segment and species group, separately. ***: p< 0.001; *: p< 00.5; ns: not significant.

.

| Fishing fleet segment | Species group (A=40; B=36) | Χ^2^ df 2 | p- level |  |
| --- | --- | --- | --- | --- |
|  |  |  |  |  |
| BT | A | 1.33 | ns |  |
|  | B | 23.92 | *** |  |
|  |  |  |  |  |
| PL | A | 2.67 | ns |  |
|  | B | 7.60 | * |  |
|  |  |  |  |  |
| PPG | A | 30.52 | *** |  |
|  | B | 43.01 | *** |  |

**APPENDIX B2 a-g.**

**Fishing risk charts by fishing segments**


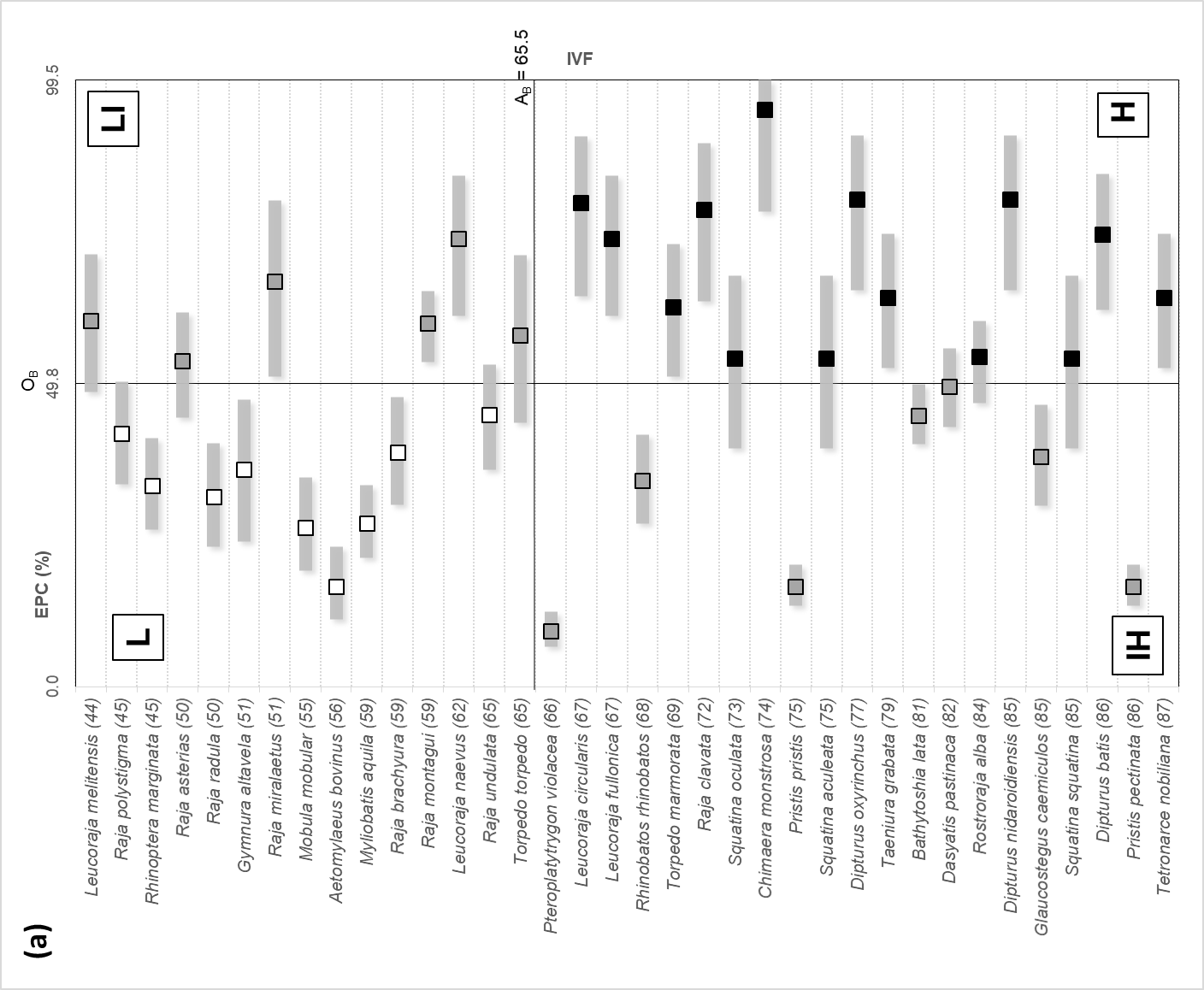


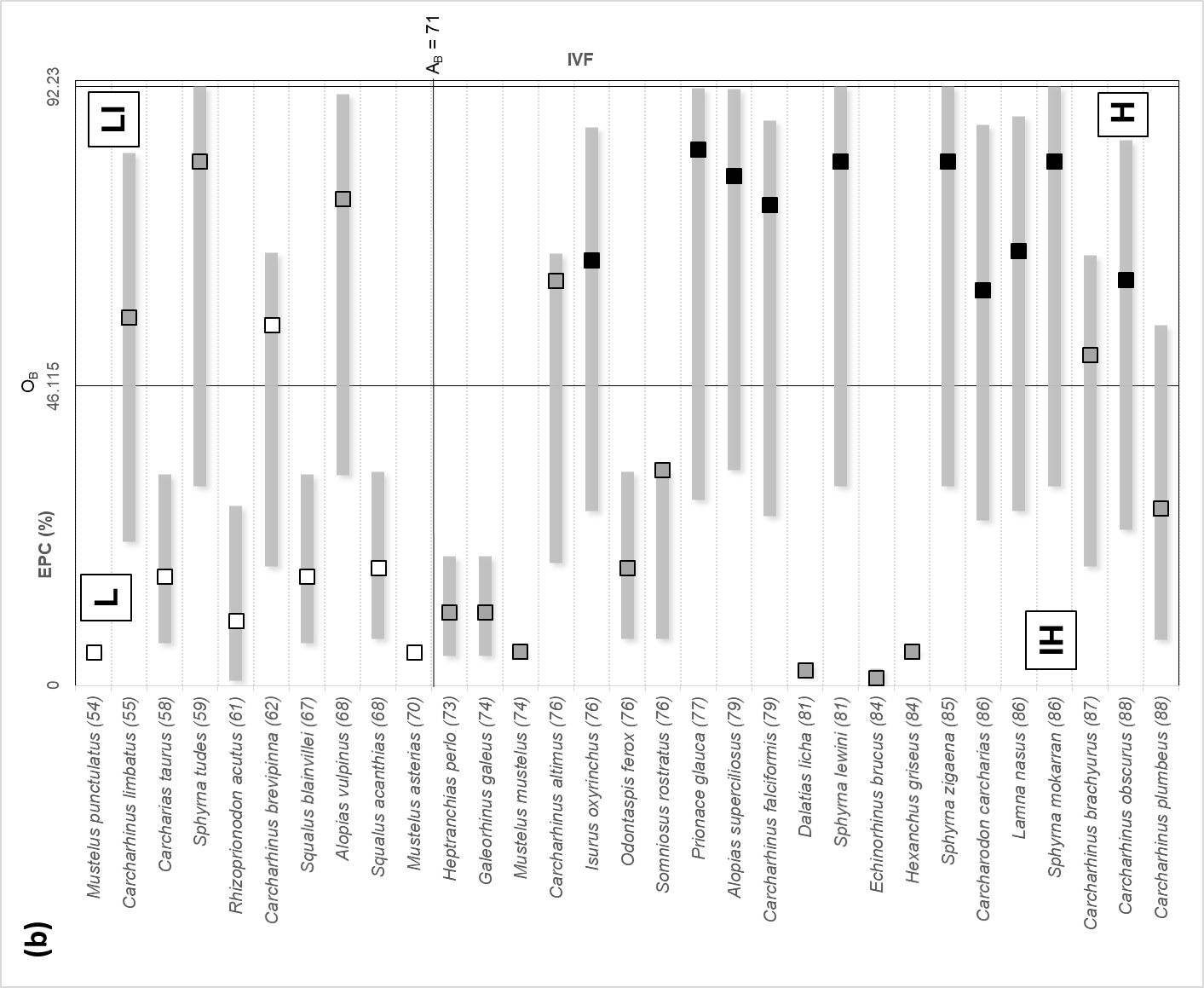


**APPENDIX B2** *a-b***.** Graphical representations of the qualitative risk from fishing for species of group B (Squatiniformes, Rajiformes, Rhinopristiformes and Chimaeriformes) in bottom trawls (**a**) and species of group A (Hexanchiformes, Lamniformes, Carcharhiniformes and Squaliformes,) in pelagic longlines fishing fleet segment (**b**). EPC (as median, 1st and 3rd quartiles) is the Estimated Probability of fatal Catch based on estimates by three independent, profiled and randomly selected sharks experts and plotted against Intrinsic Vulnerability to Fishing ***IVF*** () (values in brackets)**.** Based on half the horizontal (OB) and vertical (AB) axes, the quadrants identify four different levels of risk to fishing (L: low risk; LI: low to intermediate risk; IH: intermediate to high risk; H: high risk) where species may fall (L: white squares; LI and IH: grey squares; H: dark squares; shared IH-H: dark line) according to heuristic rules based on relative differences between species horizontal and vertical values and horizontal (O_B_) and vertical (A_B_) reference values, respectively.


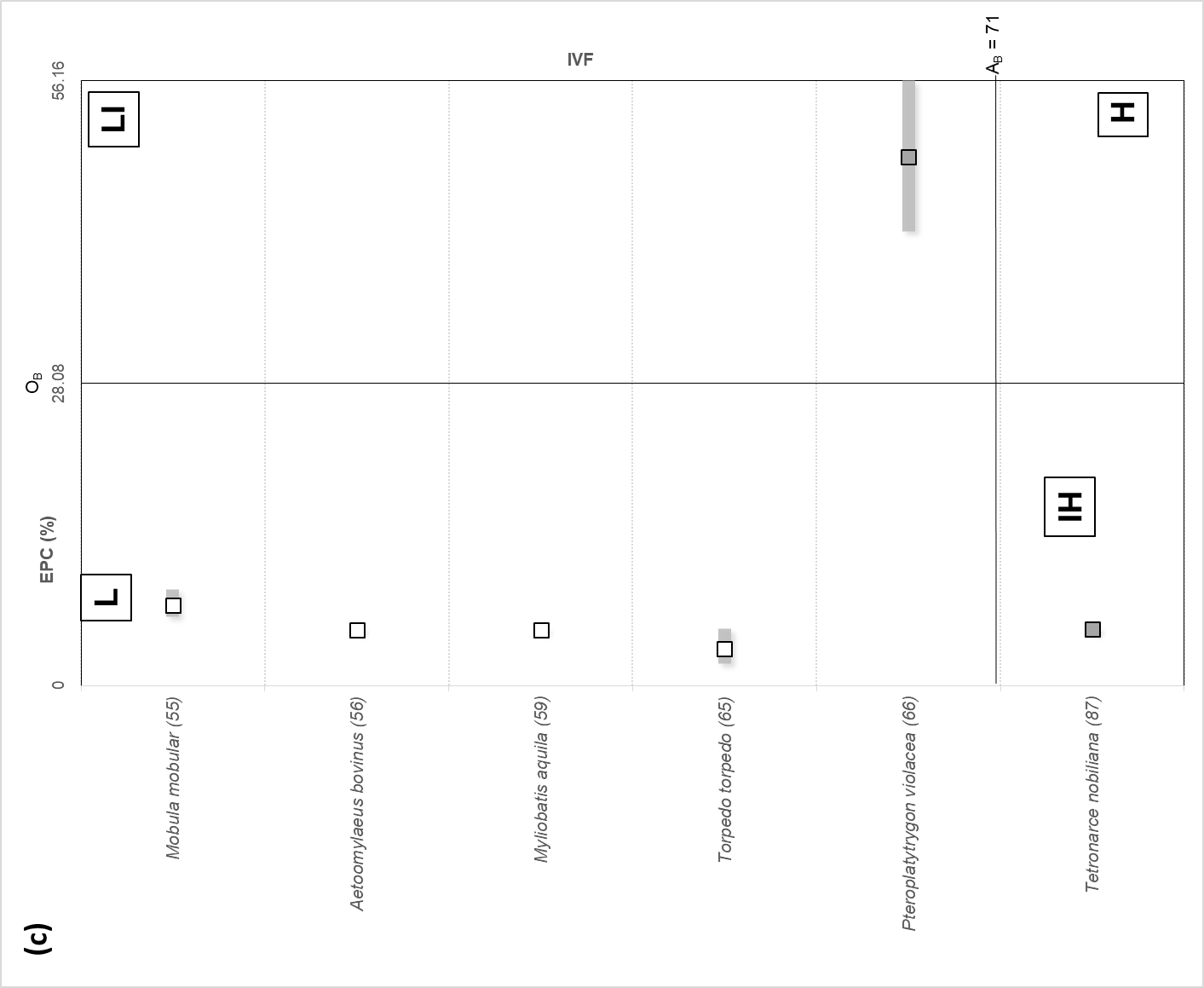

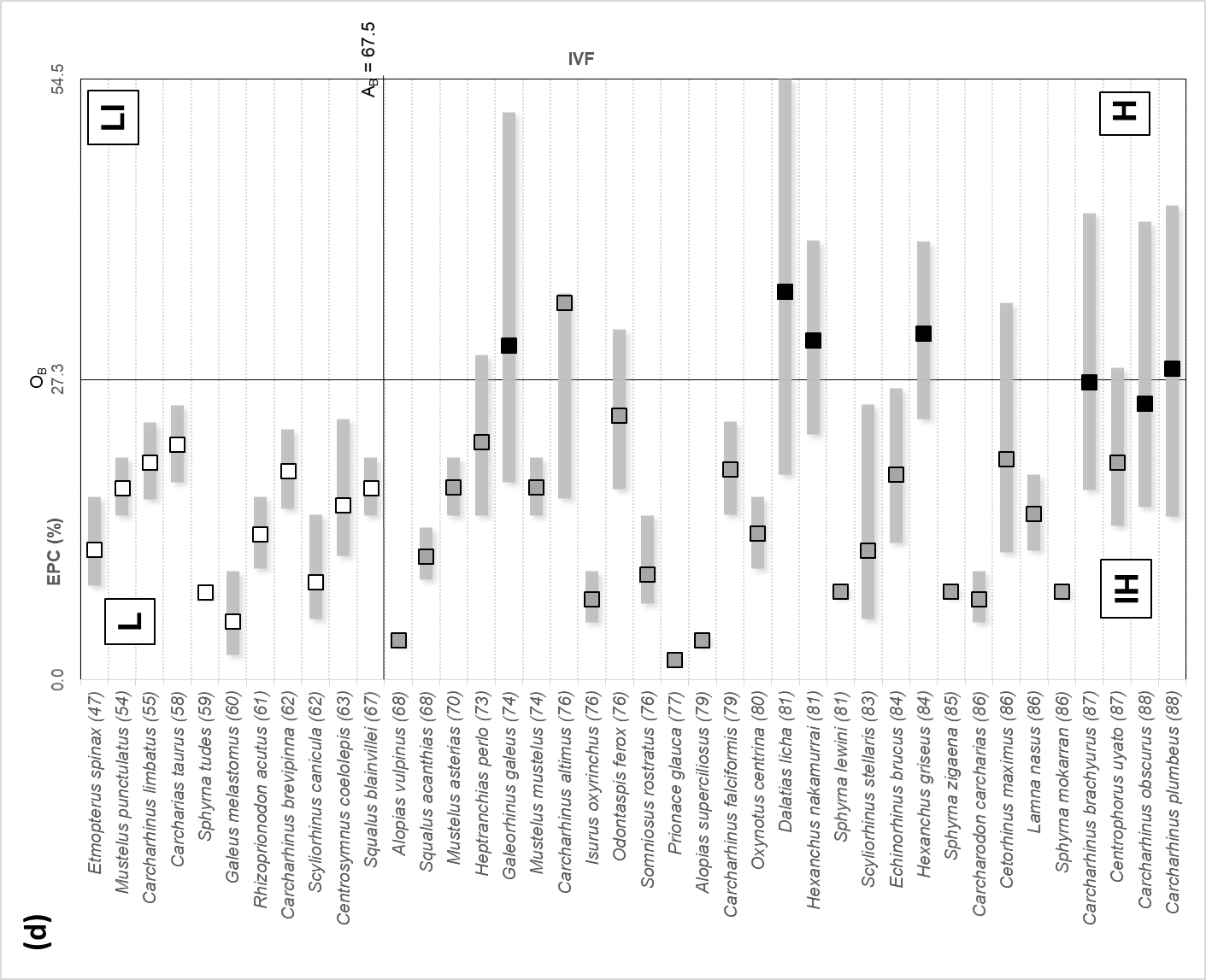


**APPENDIX B2** *c-d***.** Graphical representations of the qualitative risk from fishing for species of group B (Squatiniformes, Rajiformes, Rhinopristiformes and Chimaeriformes) in pelagic longlines (**c**) and species of group A (Hexanchiformes, Lamniformes, Carcharhiniformes and Squaliformes,) in Passive polyvalent gears fishing fleet segment (**d**). EPC (as median, 1st and 3rd quartiles) is the Estimated Probability of fatal Catch based on estimates by three independent, profiled and randomly selected sharks experts and plotted against Intrinsic Vulnerability to Fishing ***IVF*** () (values in brackets)**.** Based on half the horizontal (OB) and vertical (AB) axes, the quadrants identify four different levels of risk to fishing (L: low risk; LI: low to intermediate risk; IH: intermediate to high risk; H: high risk) where species may fall (L: white squares; LI and IH: grey squares; H: dark squares; shared IH-H: grey diamonds) according to heuristic rules based on relative differences between species horizontal and vertical values and horizontal (O_B_) and vertical (A_B_) reference values, respectively.


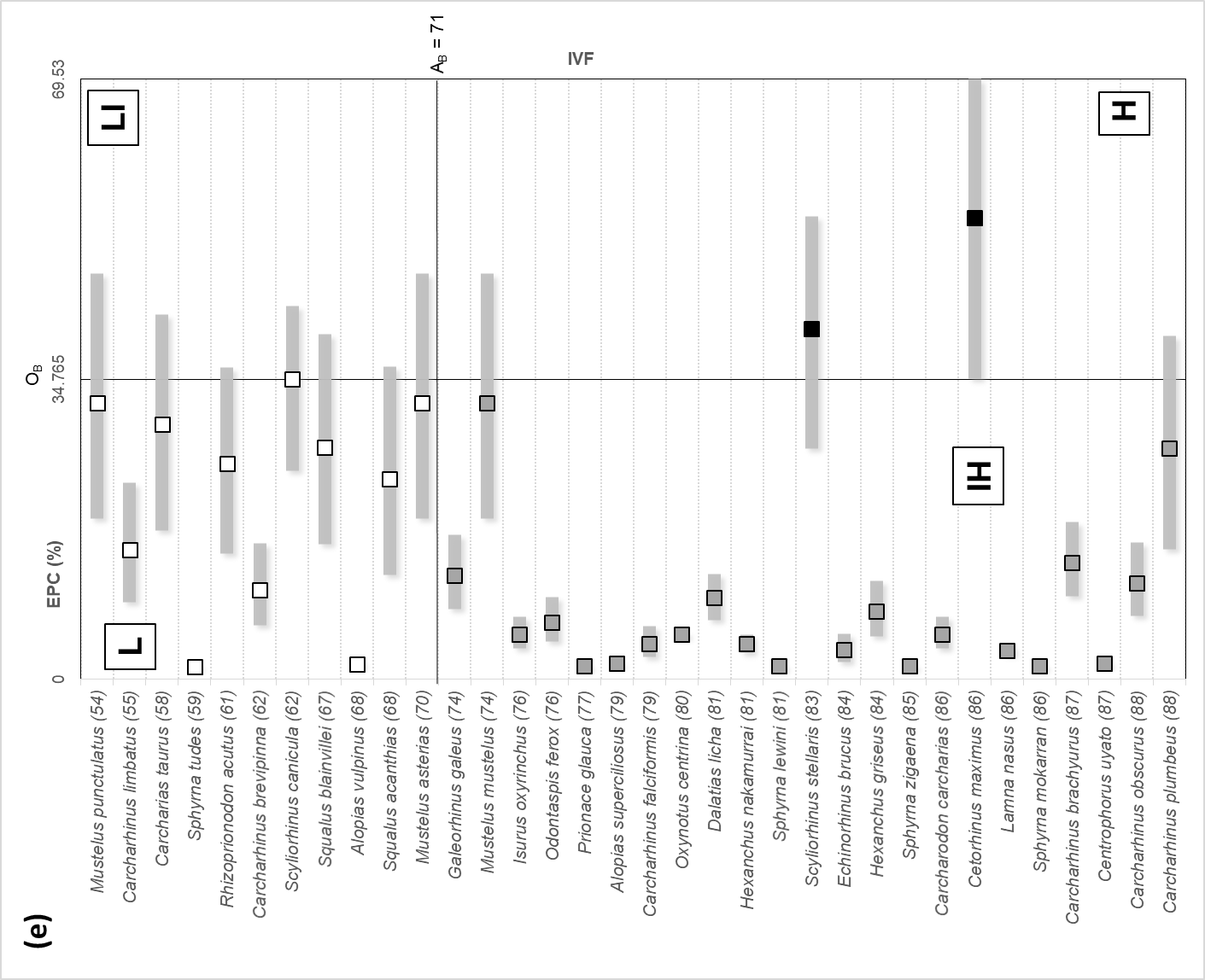


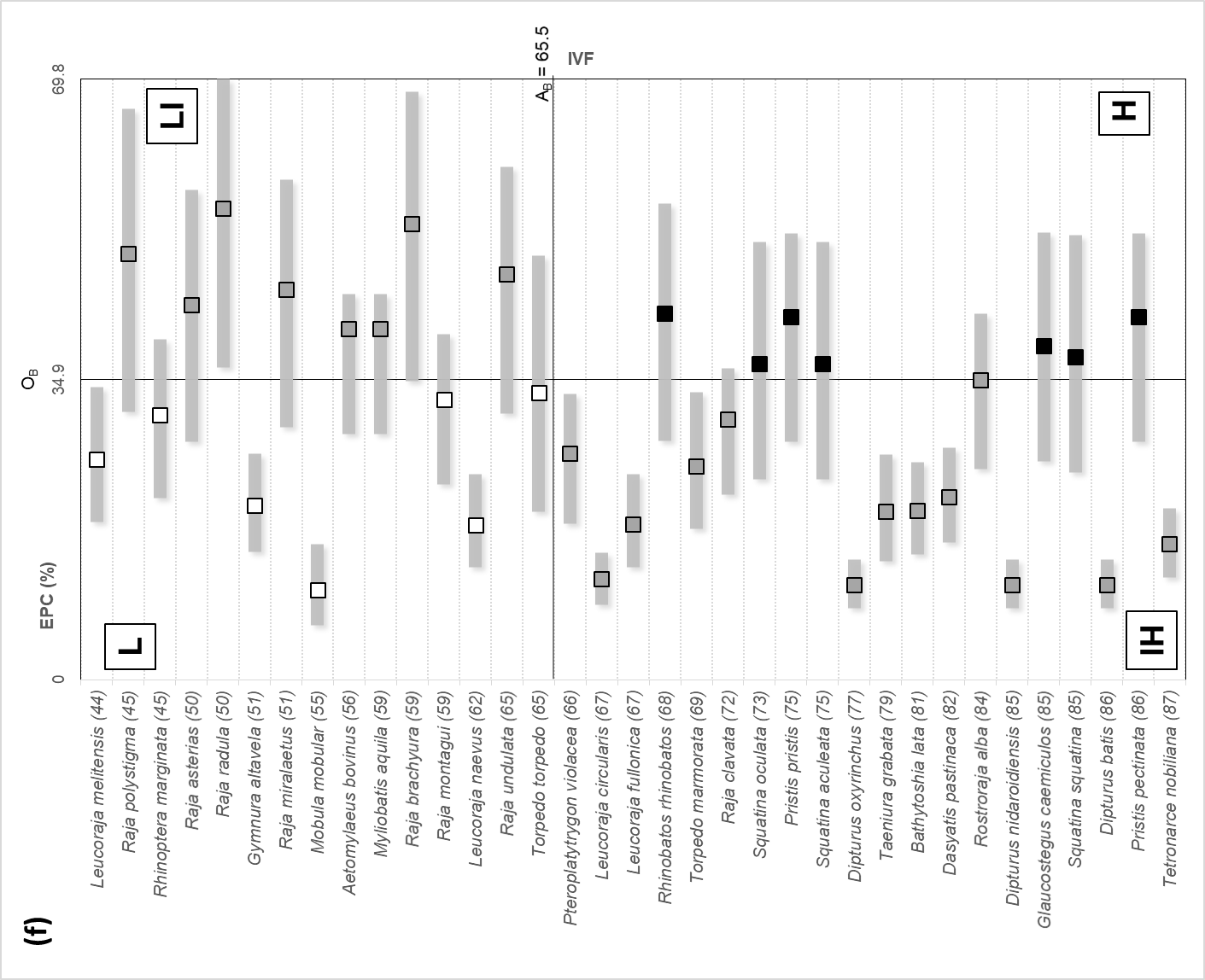


**APPENDIX B2** *e-f.* Graphical representations of the qualitative risk from fishing for species of group A (Hexanchiformes, Lamniformes, Carcharhiniformes and Squaliformes,) (**e**) and for species of group B (Squatiniformes, Rajiformes, Rhinopristiformes and Chimaeriformes) in small-scale fishery fishing fleet segment (**f**). EPC (as median, 1st and 3rd quartiles) is the Estimated Probability of fatal Catch based on estimates by three independent, profiled and randomly selected sharks experts and plotted against Intrinsic Vulnerability to Fishing ***IVF*** () (values in brackets)**.** Based on half the horizontal (OB) and vertical (AB) axes, the quadrants identify four different levels of risk to fishing (L: low risk; LI: low to intermediate risk; IH: intermediate to high risk; H: high risk) where species may fall (L: white squares; LI and IH: grey squares; H: dark squares; shared L-LI: grey diamonds) according to heuristic rules based on relative differences between species horizontal and vertical values and horizontal (O_B_) and vertical (A_B_) reference values, respectively.


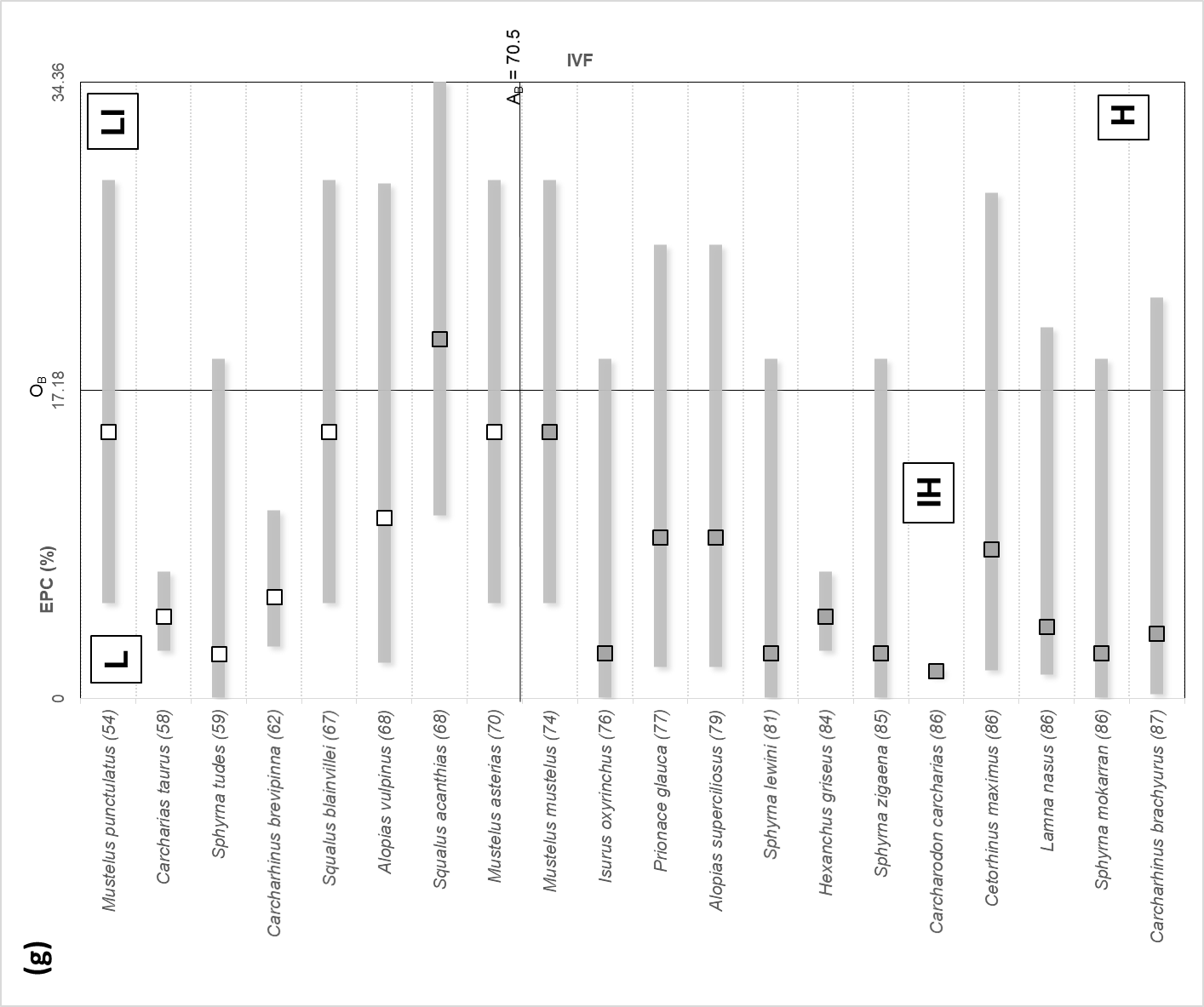

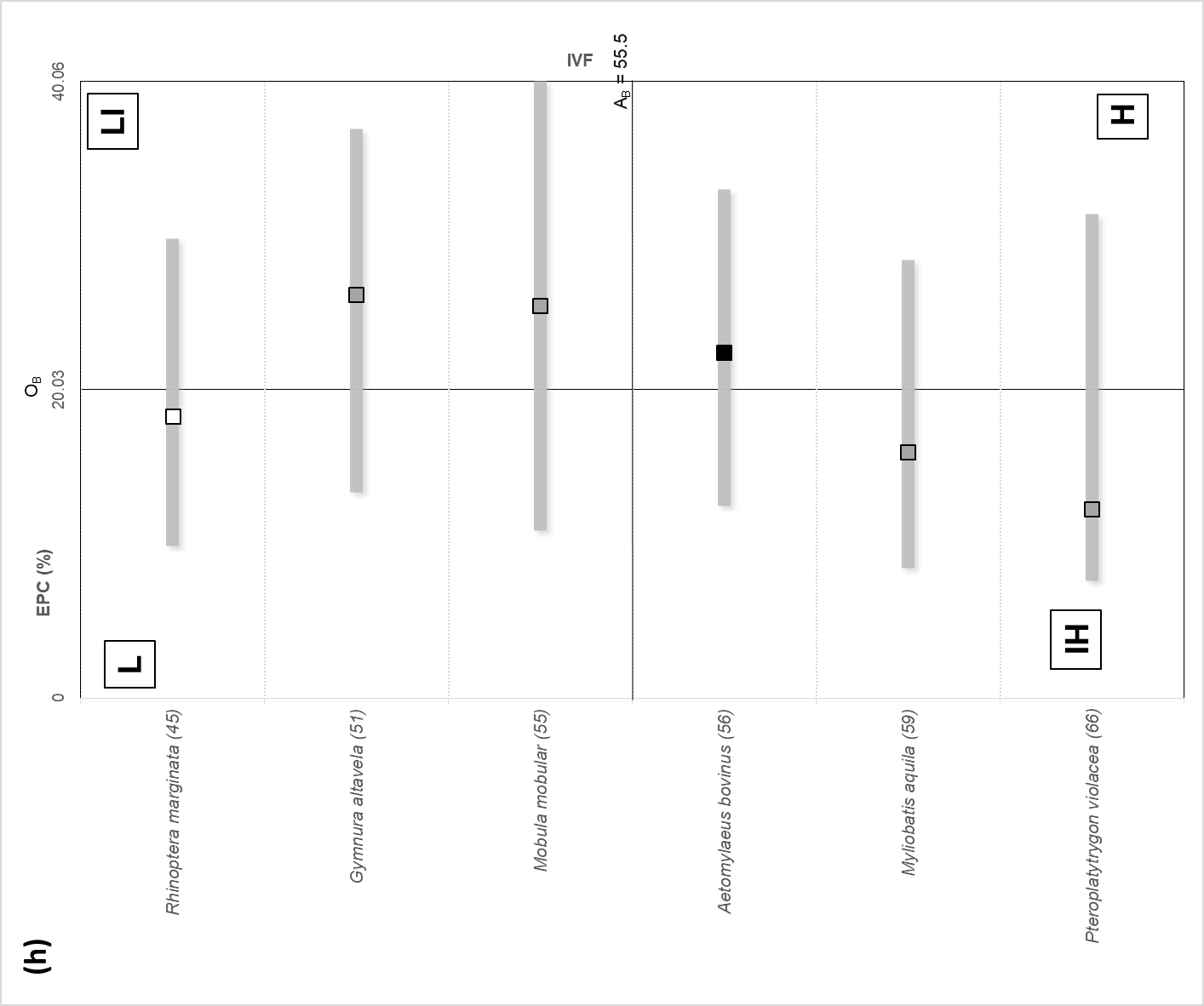


**APPENDIX B2** *g-h.* Graphical representations of the qualitative risk from fishing for species of group A (Hexanchiformes, Lamniformes, Carcharhiniformes and Squaliformes,) (**g**) and for species of group B (Squatiniformes, Rajiformes, Rhinopristiformes and Chimaeriformes) (**h**) in pelagic trawls + purse seines fishing fleet segment. EPC (as median, 1st and 3rd quartiles) is the Estimated Probability of fatal Catch based on estimates by three independent, profiled and randomly selected sharks experts and plotted against Intrinsic Vulnerability to Fishing ***IVF*** () (values in brackets)**.** Based on half the horizontal (OB) and vertical (AB) axes, the quadrants identify four different levels of risk to fishing (L: low risk; LI: low to intermediate risk; IH: intermediate to high risk; H: high risk) where species may fall (L: white squares; LI and IH: grey squares; H: dark squares L-IH: grey diamonds) according to heuristic rules based on relative differences between species horizontal and vertical values and horizontal (O_B_) and vertical (A_B_) reference values, respectively

**APPENDIX B3.**

**Breakdown of fishing threat within segments**

**A**ggregated results of chi-squared tests to check for randomness of species number among quadrants of the graphical representations of risk to fishing by fishing fleet segments. (BT: bottom trawls; PL: pelagic longlines; PPG: passive polyvalent gears; SSF: small-scale fishery; PTP: pelagic pair trawl and purse seine) and by species groups (species group A: Hexanchiformes, Lamniformes, Carcharhiniformes and Squaliformes; species group B: Squatiniformes, Rajiformes, Rhinopristiformes and Chimaeriformes). Species are aggregated into corresponding risk level (L: low; L-I: low to intermediate; I-H: intermediate to high; H: high) and are 76 cartilaginous species censed at the Mediterranean scale. Analyses were run by fishing segment and species group, separately. ***: p< 0.001; **: p< 0.01; ns: not significant

| Fishing fleet segments | N x species group | Count x quadrant | | | | Χ^2^ df 3 | p- level |
| --- | --- | --- | --- | --- | --- | --- | --- |
|  |  | L | LI | IH | H |  |  |
| BT | A=39 | 6 | 5 | 18 | 10 | 10.74 | * |
|  | B=36 | 9 | 6 | 7 | 14 | 4.22 | ns |
| PL | A=31 | 7 | 3 | 11 | 10 | 5.00 | ns |
|  | B=6 | 4 | 1 | 1 | 0 | 6.00 | ns |
| PPG | A=40 | 11 | 0 | 22 | 7 | 25.40 | *** |
|  | B=36 | 15 | 0 | 4 | 17 | 22.89 | *** |
| SSF | A=34 | 11 | 0 | 21 | 2 | 32.58 | *** |
|  | B=35 | 7 | 8 | 13 | 7 | 2.83 | ns |
| PTP | A=23 | 7 | 1 | 12 | 0 | 18.8 | *** |
|  | B=7 | 1 | 2 | 2 | 1 | 0.66 | ns |

**APPENDIX B4.**

**Breakdown of the fishing threat between segments**

Results of chi-squared test on 5 columns x 4 rows contingency table (X^2^= 29.15; df 12; p< 0.01) to check for randomness of species number between quadrants of graphical representations of risk to fishing with fishing fleet segments (BT: bottom trawls; PL: pelagic longlines; PPG: passive polyvalent gears; SSF: small-scale fishery; PTP: pelagic pair trawl and purse seine) as columns and corresponding species risk level (L: low; L-I: low to intermediate; I-H: intermediate to high; H: high) as rows. Species are 76 cartilaginous species censed at the Mediterranean scale and percent contribute to total chi-squared is displayed according to increasing order.

| Level of threat x FFL | Chi-squared | Percent contribute to total chi-squared | Deviance from expected as integer number of species |
| --- | --- | --- | --- |
| SSF L | 0.00 | 0.01 | ≈-0 |
| PL L | 0.04 | 0.15 | ≈+1 |
| PTP L | 0.08 | 0.26 | ≈+1 |
| PL LI | 0.11 | 0.36 | ≈+1 |
| PL H | 0.14 | 0.48 | ≈+1 |
| PTP LI | 0.16 | 0.54 | ≈+3 |
| PL IH | 0.39 | 1.35 | ≈-2 |
| PPG IH | 0.42 | 1.46 | ≈-4 |
| SSF LI | 0.44 | 1.49 | ≈+2 |
| BT IH | 0.59 | 2.03 | ≈-4 |
| PPG L | 1.08 | 3.70 | ≈+5 |
| SSF IH | 1.42 | 4.89 | ≈+6 |
| PTP IH | 1.50 | 5.15 | ≈+4 |
| BT L | 1.68 | 5.77 | ≈-6 |
| PPG H | 1.80 | 6.19 | ≈+6 |
| BT H | 1.98 | 6.80 | ≈+6 |
| BT LI | 2.45 | 8.41 | ≈+4 |
| SSF H | 3.47 | 11.89 | ≈-8 |
| PTP H | 4.41 | 15.12 | ≈-5 |
| PPG LI | 6.98 | 23.95 | ≈-7 |

**APPENDIX B5 a**

**PCA descriptive analysis: correlation matrix**

The table below reports the correlation matrix of active (DRB_IX_, IT or MED IERF_X_, IT or MED IFR_X_ and IVF_X_) and supplementary (IT IUCN_RX_, MED IUCN_RX_ and GLO IUCN_RX_) variables used in PCA analysis to explore data at Italian (IT) and Mediterranean (MED) scales. Based on values of autocorrelation of individual segments (RBB_IX_) with the cumulative metrics IFR_X_ and IERF_X_, (S2.3.1B a, light dotted border areas), we differentiated three groups of segments at both experimental geographical scales: two segments (PPG and SSF) were strongly and directly related to IERF_X_ whereas having no relation with IFR_X_; two others (PL and PTP) were inversely related to IFR_X_ and had weak or no relation with IERF_X_. One segment (BT) was strongly and directly related to IFR_X_ while having a lower and inverse relation with IERF_X_.

|  | | **IVF_X_** | **RRB_BTX_** | **RRB_PLX_** | **RRB_PPGX_** | **RRB_SSFX_** | **RRB_PTPX_** | **IT**  **IERF_X_** | **IT**  **IFR_X_** | **MED**  **IERF_X_** | **MED**  **IFR_X_** | ***IT**  **IUCN_RX_** | ***MED**  **IUCN_RX_** | ***GLO**  **IUCN_RX_** |
| --- | --- | --- | --- | --- | --- | --- | --- | --- | --- | --- | --- | --- | --- | --- |
| **IVF_X_** | 1.00 | | -0.10 | 0.30 | 0.43 | 0.09 | -0.05 | 0.49 | -0.04 | 0.42 | -0.03 | 0.55 | 0.50 | 0.45 |
| **RRB_BTX_** | -0.10 | | 1.00 | -0.64 | -0.09 | 0.07 | -0.50 | -0.28 | 0.99 | -0.32 | 0.99 | -0.36 | -0.28 | -0.17 |
| **RRB_PLX_** | 0.30 | | -0.64 | 1.00 | -0.19 | -0.46 | 0.35 | 0.05 | -0.57 | 0.15 | -0.60 | 0.17 | 0.21 | 0.03 |
| **RRB_PPGX_** | 0.43 | | -0.09 | -0.19 | 1.00 | 0.47 | -0.19 | 0.84 | -0.09 | 0.54 | -0.04 | 0.66 | 0.51 | 0.58 |
| **RRB_SSFX_** | 0.09 | | 0.07 | -0.46 | 0.47 | 1.00 | -0.29 | 0.65 | 0.07 | 0.77 | 0.09 | 0.30 | 0.28 | 0.40 |
| **RRB_PTPX_** | -0.05 | | -0.50 | 0.35 | -0.19 | -0.29 | 1.00 | -0.06 | -0.47 | -0.09 | -0.47 | 0.17 | 0.16 | 0.00 |
| **IT IERF_X_** | 0.49 | | -0.28 | 0.05 | 0.84 | 0.65 | -0.06 | 1.00 | -0.24 | 0.86 | -0.21 | 0.71 | 0.63 | 0.65 |
| **IT IFR_X_** | -0.04 | | 0.99 | -0.57 | -0.09 | 0.07 | -0.47 | -0.24 | 1.00 | -0.26 | 1.00 | -0.34 | -0.24 | -0.14 |
| **MED IERF_X_** | 0.42 | | -0.32 | 0.15 | 0.54 | 0.77 | -0.09 | 0.86 | -0.26 | 1.00 | -0.25 | 0.50 | 0.52 | 0.53 |
| **MED IFR_X_** | -0.03 | | 0.99 | -0.60 | -0.04 | 0.09 | -0.47 | -0.21 | 1.00 | -0.25 | 1.00 | -0.31 | -0.22 | -0.12 |
| ***IT IUCN_RX_** | 0.55 | | -0.36 | 0.17 | 0.66 | 0.30 | 0.17 | 0.71 | -0.34 | 0.50 | -0.31 | 1.00 | 0.86 | 0.80 |
| ***MED IUCN_RX_** | 0.50 | | -0.28 | 0.21 | 0.51 | 0.28 | 0.16 | 0.63 | -0.24 | 0.52 | -0.22 | 0.86 | 1.00 | 0.89 |
| ***GLO IUCN_RX_** | 0.45 | | -0.17 | 0.03 | 0.58 | 0.40 | 0.00 | 0.65 | -0.14 | 0.53 | -0.12 | 0.80 | 0.89 | 1.00 |

**Footnote to table**

DRB_IX_ is Disturbance-Resilience Balance by fishing fleet segments I (BT: bottom trawls; PL: pelagic longlines; PPG: passive polyvalent gears; SSF: small-scale fishery; PTP: pelagic pair trawl and purse seine) and species X (76 cartilaginous species censed at Mediterranean scale); IT or MED IERF_X_ is Index of Extinction Risk to Fishing and IT or MED IFR_X_ is Index of Fishing Response by species X. IVF_X_ is Intrinsic Vulnerability to Fishing by species X used as control variable. GLO, MED and IT IUCN_RX_ are ranked IUCN extinction risk categories by species with GLO IUCN_RX_ used as control variable. Asterisked variables are supplementary and shadowed green cells contain correlations used to discuss the model: light dotted border areas represent values of autocorrelation between active experimental variables (individual vs cumulative); hard dotted border areas represent values of correlation between active experimental (cumulative) and supplementary variables, comprised of control (underlined values); continuous line bordered areas represent values of correlation between supplementary variables; open shadowed green cells show correlation between control active variable and supplementary variable

**APPENDIX B5 b.**

**PCA Biplot**

The bi-plot below shows that 72.60% of the total variance is represented by two main orthogonal factors at Italian (IT) and Mediterranean (MED) scales. Correlation vectors of active (DRB_IX_, IT or MED IERF_X_, IT or MED IFR_X_ and IVF_X_ solid dark line) and supplementary (IT IUCN_RX_, MED IUCN_RX_ and GLO IUCN_RX_, asterisked, dotted dark line) variables differ in length and angle to factors (the bigger is the length, the higher the average correlation between variables; the smaller is the angle, the higher the correlation with the factor forming that angle). Based on disposition (vector length and angle) of correlation vectors and values from the correlation matrix (*Table)*, we interpreted the two principal factors extracted as informing on impact of mono-gear vs multi-gear segment’s characteristics (Fact. 2) and fishing effort (Fact. 1), with Fact. 1 increasing from the left to the right, and Fact. 2 from the bottom to the top, respectively, at both scales. BT and IFR_X_ was more associated to factor 1 than 2; PL and PTP to both factors; PPG, SSF, and IERF_X_ were more associated to factor 2 than 1.
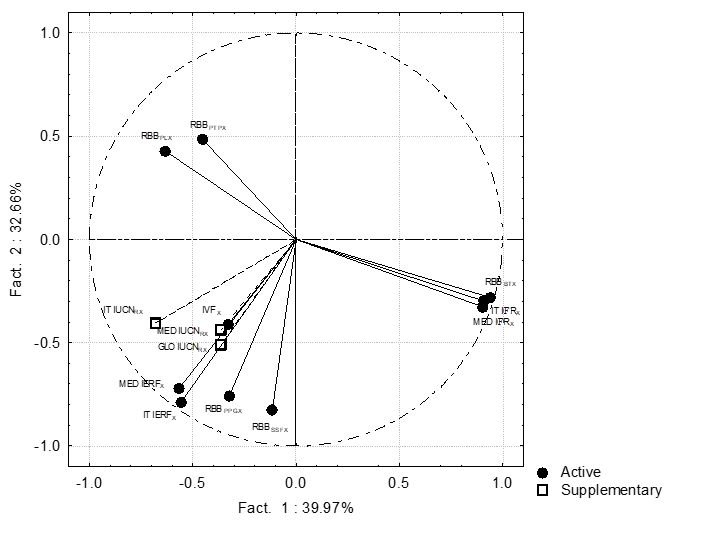


**Footnote to bi-plot**

DRB_IX_ is Resistance Resilience Balance by fishing fleet segments I (BT: bottom trawls; PL: pelagic longlines; PPG: passive polyvalent gears; SSF: small-scale fishery; PTP: pelagic pair trawl and purse seine) and species X (76 cartilaginous species censed at Mediterranean scale); IT or MED IERF_X_ and IT or MED IFR_X_ are Indexes of Extinction Risk to Fishing and of Overfishing Response by species. IVF_X_ is Intrinsic Vulnerability to Fishing by species X and used as control variable. GLO, MED and IT IUCN_RX_ are IUCN ranked extinction risk categories by species with GLO IUCN_RX_ and used as control variable.

**APPENDIX B6.**

**Kruskal-Wallis ANOVA and pairwise comparisons**

The table below reports aggregated results from Kruskal Wallis Anovas on differences in RRB_IX_, IERF_X_ and IFR_X_ between X= 76 Mediterranean cartilaginous species grouped according to IUCN extinction risk categories at Italian (IT) and Mediterranean (MED) scales, within I=five representative fishing fleet segments (BT: bottom trawls; PL: pelagic longlines; PPG: passive polyvalent gears; SSF: small-scale fishery; PTP: pelagic pair trawl and purse seine). Data showed much more significant results at the Italian scale compared to Mediterranean scale; in particular, non-threatened species (LC and NT) showed significant lower values than threatened (VU, EN and CR) in RRB_PLX_, RRB_PPGX_, RRB_PTPX_ and IERF_X_. Threatened species showed lower values than non-threatened in the cases of RRB_BTX_ and IFR_X_; some differences resulted within threatened species in RRB_SSFX_ (VU<CR) and in RRB_PTPX_ (EN>CR). DD had higher values than LC species in RRB_PPGX_; lower values than CR in RRB_SSFX_ and IERF_X_; lower than EN species in RRB_PTPX_. Significant differences limited to RRB_PPGX_ and IERF_X_ at the Mediterranean scale. The former showed VU and DD species to have higher values than LC and NT species; the latter showed LC species to have lower values than EN and CR species.

| IT | DRB _BT X_  H (5;76) =14.69  * | DRB _PL X_  H (5;76) =16.50  ** | DRB _PPG X_  H (5;76) =24.06  *** | DRB _SSF X_  H (5;76) =12.98  * | DRB _PTP X_  H (5;76) =18.96  ** | IT IERF_X_  H (5;76) =20.38  ** | IT IFR_X_  H (5;76) =13.78  * |
| --- | --- | --- | --- | --- | --- | --- | --- |
| LC | a*b* | c* | d***e** | - | i* | l*** | n* |
| NT | - | - | - | - | - | - | - |
| VU | b* | - | f* | h* | - | - | - |
| EN | a* | c* | - | - | i*j*k* | - | n* |
| CR | - | - | d***f* | g*h* | j* | l***m* | - |
| DD | - | - | e** | g* | k* | m* | - |
| MED | DRB _BT X_  H (5;76) =8.07  ns | DRB _PL X_  H (5;76) =9.18  ns | DRB _PPG X_  H (5;76) =18.46  ** | DRB _SSF X_  H (5;76) =3.76  ns | DRB _PTP X_  H (5;76) =10.15  ns | MED IERF_X_  H (5;76) =15.59  * | MED IFR_X_  H (5;76) =6.93  ns |
| LC | na | na | p*q* | na | na | r**s* | na |
| NT | na | na | o*q* | na | na | - | na |
| VU | na | na | q* | na | na | - | na |
| EN | na | na | - | na | na | s* | na |
| CR | na | na | - | na | na | r** | na |
| DD | na | na | o*p* | na | na | - | na |
|  |  |  |  |  |  |  |  |

Footnote to table

RRB_IX_ is Resistance Resilience Balance by fishing fleet segments I and species X; IT or MED IERF_X_ and IT or MED IFR_X_ are Indexes of Extinction Risk to Fishing and of Overfishing Response by species. Significance codes: *P<0.05, **P<0.01, ***P<0.001; same letter denote significantly different groups. na and ns refer to not applicable and not significant, respectively.

**APPENDIX B7 a**.

**Percentile regression of IERF*_X_***

The table below reports estimated parameters for the slope of the linear 10^th^ stepped percentile regression on IT (upper half) or MED (lower half) IERF_X_ as dependent and IT or MED IUCN_RX_ as independent variables. IERF_X_ is Index of Extinction Risk to Fishing by X species. Significance codes: *p<0.05. **p<0.01, ***p<0.001.

| **Model: IT IERF_X_=a*(IT IUCN_RX_)** | | **Slope parameters** | | | | | |  |
| --- | --- | --- | --- | --- | --- | --- | --- | --- |
|  |  | Est | Std. Error | t (4) | p | Lower conf. | Upper conf. |  |
| Percentiles | R^2^ |  |  |  |  |  |  |  |
| 10^TH^ | 0.46 | 0.09 | 0.01 | 7.46 | ** | 0.06 | 0.13 |  |
| 20^TH^ | 0.62 | 0.11 | 0.03 | 7.76 | ** | 0.07 | 0.15 |  |
| 30^TH^ | 0.70 | 0.12 | 0.01 | 9.47 | *** | 0.09 | 0.16 |  |
| 40^TH^ | 0.80 | 0.13 | 0.01 | 12.23 | *** | 0.10 | 0.16 |  |
| 50^TH^ | 0.70 | 0.14 | 0.01 | 11.10 | *** | 0.10 | 0.17 |  |
| 60^TH^ | 0.30 | 0.14 | 0.02 | 8.38 | ** | 0.10 | 0.19 |  |
| 70^TH^ | 0.46 | 0.16 | 0.02 | 8.03 | ** | 0.10 | 0.21 |  |
| 80^TH^ | 0.13 | 0.16 | 0.02 | 6.89 | ** | 0.10 | 0.23 |  |
| 90^TH^ | 0.00 | 0.17 | 0.03 | 5.80 | ** | 0.09 | 0.26 |  |
|  |  |  |  |  |  |  |  |  |
| **Model: MED IERF_X_=a*(MED IUCN_RX_)** | |  |  |  |  |  |  |  |
|  |  |  |  |  |  |  |  |  |
| 10^TH^ | 0.18 | 0.06 | 0.01 | 6.70 | ** | 0.03 | 0.08 |  |
| 20^TH^ | 0.00 | 0.07 | 0.01 | 5.74 | ** | 0.04 | 0.11 |  |
| 30^TH^ | 0.00 | 0.09 | 0.01 | 5.60 | ** | 0.04 | 0.13 |  |
| 40^TH^ | 0.03 | 0.10 | 0.02 | 6.41 | ** | 0.06 | 0.15 |  |
| 50^TH^ | 0.23 | 0.13 | 0.01 | 8.24 | ** | 0.09 | 0.17 |  |
| 60^TH^ | 0.00 | 0.14 | 0.02 | 6.86 | ** | 0.08 | 0.19 |  |
| 70^TH^ | 0.00 | 0.15 | 0.02 | 5.98 | ** | 0.08 | 0.22 |  |
| 80^TH^ | 0.00 | 0.16 | 0.03 | 4.99 | ** | 0.07 | 0.25 |  |
| 90^TH^ | 0.00 | 0.18 | 0.04 | 5.07 | ** | 0.08 | 0.28 |  |

**APPENDIX B7** **b**.

**Percentile regression of IFR_X_**

The table below reports the estimated parameters (a, b and c) of the quadratic 10^th^ stepped percentile regression on IT (upper half) or MED (lower half) IFR_X_ as dependent and IT or MED IUCN_RX_ as independent variables. IFR_X_ is Index of Overfishing Response by X species. Significance codes:*p<0.05. **p<0.01, ***p<0.001; borderline significances are in brackets

| **Model IT IFR_X_= a*(IT IUCN_RX_)^2^ – b*(IT IUCN_RX_) + c** | | | | | | | | | | | | | | | | | | | |
| --- | --- | --- | --- | --- | --- | --- | --- | --- | --- | --- | --- | --- | --- | --- | --- | --- | --- | --- | --- |
|  |  | **a** | | | | | | **b** | | | | | | **c** | | | | | |
| Percentiles | R^2^ | Est | Std. Error | t (2) | p | Lower conf. | Upper conf. | Est | Std. Error | t (2) | p | Lower conf. | Upper conf. | Est | Std. Error | t (2) | p | Lower conf. | Upper  conf. |
|  |  |  |  |  |  |  |  |  |  |  |  |  |  |  |  |  |  |  |  |
| 10^TH^ | 0.90 | 42.49 | 10.61 | 4.00 | (0.06) | -3.15 | 88.13 | 54.28 | 12.97 | 4.18 | (0.05) | -1.54 | 110.10 | 25.69 | 3.40 | 7.55 | * | 11.04 | 40.34 |
| 20^TH^ | 0.90 | 82.93 | 22.94 | 3.61 | (0.07) | -15.78 | 181.64 | 111.38 | 28.06 | 3.97 | (0.06) | -9.36 | 232.11 | 44.87 | 7.36 | 6.09 | * | 13.18 | 76.56 |
| 30^TH^ | 0.96 | 112.72 | 16.52 | 6.82 | * | 41.64 | 183.80 | 141.01 | 20.21 | 6.98 | * | 54.07 | 227.95 | 51.70 | 5.30 | 9.75 | * | 28.88 | 74.52 |
| 40^TH^ | 0.96 | 123.26 | 18.24 | 6.76 | * | 44.76 | 201.75 | 155.89 | 22.31 | 6.99 | * | 59.89 | 251.89 | 57.20 | 5.85 | 9.77 | ** | 32.00 | 82.40 |
| 50^TH^ | 0.97 | 141.46 | 17.27 | 8.19 | * | 67.149 | 215.78 | 177.11 | 21.12 | 8.38 | * | 86.22 | 268.01 | 63.59 | 5.54 | 11.47 | ** | 39.74 | 87.45 |
| 60^TH^ | 0.97 | 204.37 | 24.34 | 8.40 | * | 99.65 | 309.08 | 245.53 | 29.77 | 8.25 | * | 117.45 | 373.61 | 81.14 | 7.81 | 10.39 | ** | 47.53 | 114.75 |
| 70^TH^ | 0.99 | 207.77 | 12.07 | 17.21 | ** | 155.82 | 259.71 | 253.64 | 14.76 | 17.18 | ** | 190.11 | 317.17 | 87.23 | 3.87 | 22.51 | ** | 70.56 | 103.90 |
| 80^TH^ | 0.99 | 240.46 | 5.91 | 40.68 | *** | 215.02 | 265.89 | 299.90 | 7.23 | 41.48 | *** | 268.79 | 331.01 | 106.40 | 1.90 | 56.07 | *** | 98.23 | 114.56 |
| 90^TH^ | 0.98 | 250.73 | 23.17 | 10.82 | ** | 151.04 | 350.42 | 323.89 | 28.34 | 11.43 | ** | 201.95 | 445.82 | 119.97 | 7.44 | 16.13 | ** | 87.97 | 151.97 |
|  |  |  |  |  |  |  |  |  |  |  |  |  |  |  |  |  |  |  |  |
| **Model MED IFR_X_= a*(MED IUCN_RX_)^2^ – b*(MED IUCN_RX_) + c** | | | | | | | | | | | | | | | | | | | |
|  |  | **a** | | | | | | **b** | | | | | | **c** | | | | | |
|  |  |  | | | | | |  | | | | | |  | | | | | |
| 10^TH^ | 0.87 | 34.08 | 12.81 | 2.66 | 0.12 | -21.06 | 89.22 | 48.44 | 15.67 | 3.09 | (0.09) | -18.99 | 115.89 | 25.48 | 4.11 | 6.19 | * | 7.78 | 43.18 |
| 20^TH^ | 0.88 | 58.34 | 20.47 | 2.85 | 0.10 | -29.73 | 146.41 | 82.21 | 25.04 | 3.28 | (0.08) | -25.51 | 189.94 | 38.07 | 6.57 | 5.79 | * | 9.80 | 66.34 |
| 30^TH^ | 0.71 | 44.65 | 29.54 | 1.51 | 0.27 | -82.46 | 171.76 | 65.16 | 36.13 | 1.80 | 0.21 | -90.31 | 220.63 | 35.34 | 9.48 | 3.73 | (0.06) | -5.46 | 76.15 |
| 40^TH^ | 0.76 | 0.89 | 37.65 | 0.02 | 0.98 | -161.12 | 162.89 | 23.26 | 46.05 | 0.50 | 0.66 | -174.89 | 221.41 | 35.44 | 12.09 | 2.93 | 0.10 | -16.56 | 87.45 |
| 50^TH^ | 0.49 | 42.00 | 50.11 | 0.84 | 0.49 | -173.59 | 257.59 | 63.47 | 61.29 | 1.03 | 0.41 | -200.2 | 327.17 | 45.66 | 16.08 | 2.84 | 0.10 | -23.54 | 114.87 |
| 60^TH^ | 0.53 | 74.82 | 50.81 | 1.47 | 0.28 | -143.80 | 293.45 | 94.20 | 62.15 | 1.51 | 0.27 | -173.20 | 361.61 | 55.76 | 16.31 | 3.42 | (0.07) | -14.42 | 125.94 |
| 70^TH^ | 0.20 | 58.0 | 101.52 | 0.58 | 0.62 | -378.13 | 495.53 | 80.16 | 124.18 | 0.64 | 0.58 | -454.13 | 614.46 | 60.04 | 32.59 | 1.84 | 0.21 | -80.18 | 200.26 |
| 80^TH^ | 0.15 | 55.72 | 112.68 | 0.49 | 0.67 | -429.08 | 540.53 | 75.66 | 137.82 | 0.55 | 0.64 | -517.32 | 668.63 | 63.01 | 36.17 | 1.74 | 0.22 | -92.61 | 218.63 |
| 90^TH^ | 0.13 | 39.43 | 87.60 | 0.45 | 0.70 | -337.47 | 416.34 | 54.20 | 107.14 | 0.50 | 0.66 | -406.79 | 515.20 | 64.00 | 28.12 | 2.28 | 0.15 | -56.98 | 184.99 |
|  |  |  |  |  |  |  |  |  |  |  |  |  |  |  |  |  |  |  |  |

**APPENDIX B8**.

**General Linear Models**

The table below reports aggregated results of the General Linear Model analyses used to check for the best models among nine (reference number in the intercept heading) including: Intrinsic Vulnerability to fishing (IVF_X_), Indices of Extinction Risk to Fishing (IERF_X_) and Overfishing Response (IFR_X_), Sum of Weighted Qualitative Scores (effort divided SW(/)QS_X_ and effort multiplied SW(*)QS_X_) at Italian and Mediterranean scale as response variables, and IUCN ranked categories at Italian (IT IUCN_RX_), Mediterranean (MED IUCN_RX_) and global (GLO IUCN_RX_) scales as continuous predictors. Significance codes:*p<0.05. **p<0.01, ***p<0.001. A Beta error distribution and a logit link function are assumed in the analyses. Type of estimator: maximum likelihood. Significance codes: *p<0.05, ***p<0.001.

|  | *Estimate* | *St. err* | *z value* | *Pr(>\|t\|)* | *Number of iterations* | *Pseudo-R² (McFadden)* | *Phi coefficients* | *Log-likelihood* |
| --- | --- | --- | --- | --- | --- | --- | --- | --- |
| 1 Intercept (IVF_X_) | -3.65 | 0.06 | -56.0.06-60.09 | *** | 2265 (BFGS) + 4 (Fisher scoring) | 0.30 | Estimate 1321.5  Std. Error 325.8  z value  4.056  Pr(>\|z\|) *** | 130.3  (Df:5) |
| IT IUCN_RX_ | 0.28 | 0.15 | 1.88 0.15 1.89 0.06 | 0.06 |  |  |  |  |
| GLO IUCN_RX_ | -0.07 | 0.20 | -0.33 | 0.74 |  |  |  |  |
| MED IUCN_RX_ | 0.09 | 0.23 | 0.37 | 0.71 |  |  |  |  |
|  |  |  |  |  |  |  |  |  |
| 2 Intercept (IT IERF_X_) | -4.29 | 0.18 | -24.20 | *** | 138 (BFGS) + 2 (Fisher scoring) | 0.48 | Estimate 218.51  Std. Error 54.49  z value  4.01  Pr(>\|z\|) *** | 103.4  (Df:5) |
| IT IUCN_RX_ | 0.87 | 0.36 | 2.40 | * |  |  |  |  |
| GLO IUCN_RX_ | 0.43 | 0.51 | 0.86 | 0.39 |  |  |  |  |
| MED IUCN_RX_ | -0.01 | 0.59 | -0.02 | 0.98 |  |  |  |  |
|  |  |  |  |  |  |  |  |  |
| 3 Intercept (IT IFR_X_) | -3.21 | 0.19 | -16.66 | *** | 62 (BFGS) + 6 (Fisher scoring) | 0.14 | Estimate 107.04  Std. Error 27.04  z value  3.96  Pr(>\|z\|) *** | 92.57  (Df:5) |
| IT IUCN_RX_ | -0.99 | 0.49 | 2.00 | * |  |  |  |  |
| GLO IUCN_RX_ | 0.67 | 0.71 | 0.94 | 0.35 |  |  |  |  |
| MED IUCN_RX_ | -0.11 | 0.78 | -0.14 | 0.88 |  |  |  |  |
|  |  |  |  |  |  |  |  |  |
| 4 Intercept (MED IERF_X_) | -4.18 | 0.21 | -19.85 | *** | 105 (BFGS) + 5 (Fisher scoring) | 0.28 | Estimate 139.79  Std. Error 35.12  z value  3.98  Pr(>\|z\|) *** | 96.66  (Df:5) |
| IT IUCN_RX_ | 0.38 | 0.43 | 0.89 | 0.37 |  |  |  |  |
| GLO IUCN_RX_ | 0.36 | 0.60 | 0.60 | 0.55 |  |  |  |  |
| MED IUCN_RX_ | 0.39 | 0.69 | 0.56 | 0.57 |  |  |  |  |
|  |  |  |  |  |  |  |  |  |
| 5 Intercept (MED IFR_X_) | -3.24 | 0.20 | -16.20 | *** | 28 (BFGS) + 6 (Fisher scoring) | 0.12 | Estimate 99.69  Std. Error 25.23  z value  3.95  Pr(>\|z\|) *** | 91.64  (Df:5) |
| IT IUCN_RX_ | -0.91 | 0.51 | -1.78 | 0.07 |  |  |  |  |
| GLO IUCN_RX_ | 0.77 | 0.74 | 1.03 | 0.30 |  |  |  |  |
| MED IUCN_RX_ | -0.23 | 0.81 | -0.28 | 0.78 |  |  |  |  |
|  |  |  |  |  |  |  |  |  |
| 6 Intercept (IT SW_(/)_QS_X_) | -4.22 | 0.17 | -24.93 | *** | 107 (BFGS) + 3 (Fisher scoring) | 0.49 | Estimate 238.59  Std. Error 59.42  z value  4.02  Pr(>\|z\|) *** | 104.6  (Df:5) |
| IT IUCN_RX_ | 0.73 | 0.34 | 2.17 | * |  |  |  |  |
| GLO IUCN_RX_ | -0.50 | 0.53 | -1.16 | 0.24 |  |  |  |  |
| MED IUCN_RX_ | 0.88 | 0.50 | 1.76 | 0.08 |  |  |  |  |
|  |  |  |  |  |  |  |  |  |
| 7 Intercept (IT SW_(*)_QS_X_) | -3.84 | 0.13 | -28.68 | *** | 359 (BFGS) + 4 (Fisher scoring) | 0.30 | Estimate 323.50  Std. Error 80.20  z value  4.03  Pr(>\|z\|) *** | 108.20  (Df:5) |
| IT IUCN_RX_ | 0.31 | 0.29 | 1.06 | 0.29 |  |  |  |  |
| GLO IUCN_RX_ | -0.56 | 0.37 | -1.51 | 0.13 |  |  |  |  |
| MED IUCN_RX_ | 0.82 | 0.42 | 1.93 | 0.05 |  |  |  |  |
|  |  |  |  |  |  |  |  |  |
| 8 Intercept (MED SW_(/)_QS_X_) | -4.30 | 0.21 | -20.45 | *** | 84 (BFGS) + 3 (Fisher scoring) | 0.41 | Estimate 328.61  Std. Error 81.47  z value  4.03  Pr(>\|z\|) *** | 108.50  (Df:5) |
| IT IUCN_RX_ | 0.66 | 0.41 | 1.61 | 0.11 |  |  |  |  |
| GLO IUCN_RX_ | -0.38 | 0.53 | -0.72 | 0.47 |  |  |  |  |
| MED IUCN_RX_ | 0.98 | 0.61 | 1.59 | 0.11 |  |  |  |  |
|  |  |  |  |  |  |  |  |  |
| 9 Intercept (MED SW_(*)_QS_X_) | -3.87 | 0.13 | -28.90 | *** | 293 (BFGS) + 4 (Fisher scoring) | 0.33 | Estimate 153.20  Std. Error 38.46  z value  3.98  Pr(>\|z\|) *** |  |
| IT IUCN_RX_ | 0.35 | 0.29 | 1.21 | 0.23 |  |  |  |  |
| GLO IUCN_RX_ | -0.58 | 0.37 | -1.57 | 0.11 |  |  |  |  |
| MED IUCN_RX_ | 0.83 | 0.42 | 1.96 | * |  |  |  |  |
|  |  |  |  |  |  |  |  |  |

**APPENDIX B9**

**GLOSSARY**

**AB_I_**: Abscissa Benchmark by fishing fleet segment I

**BT**: Bottom Trawls

**EPC_IX_**: Estimates of Probability of fatal Catch by fishing fleet segment I and species X

**FFL**: Fishing Fleet segment.

**f_I_**: fishing effort as average gross tonnage x fishing days by I fishing fleet segments

**GLMA**: General Linear Model Analysis

**KWA**: Kruskal Wallis Anova

**IT** or **MED** **SW_(/)_QS_X_**=Σ_I_ (QS_IX_)/f_I_. Sum of fishing effort Weighted (divided) Qualitative Scores species X at Italian or Mediterranean scale, respectively, with I as fishing fleet segments.

**IT** or **MED** **SW_(*)_QS_X_**=Σ_I_ (QS_IX_)*f_I_. Sum of fishing effort Weighted (multiplied) Qualitative Scores by species X at Italian or Mediterranean scale, respectively, with I as fishing fleet segments.

**IT** or **MED** **f_I_**: mean relative percentage of fishing effort (as gross tonnage x fishing days) by fishing fleet segment I at Italian or Mediterranean scale.

**IT** or **MED** **IFR_X_**=Σ_I_ (RRB_IX_)*f_I_. Index of Fishing Response by species X at Italian or Mediterranean scale, respectively, with I as fishing fleet segments.

**IT IOR_X_**=’(IT IFR_X_). Index of Overfishing Response by species X at Italian scale.

**IT** or **MED** **IERF_X_**=ΣI (RRB_IX_)/f_I_ Index of Extinction Risk to Fishing by species X at Italian or Mediterranean scale, respectively, with I as fishing fleet segments.

**IT** or **MED** or **GLO** **IUCN_RX_**: ranked IUCN extinction risk categories at Italian or Mediterranean or Global scale by species X, respectively.

**IT** or **MED** or **GLO** **IUCN**: IUCN extinction risk categories at Italian or Mediterranean or Global scale by species X, respectively.

**IVF_X_**: Intrinsic Vulnerability to Fishing (Cheung et al, 2005) by species X

**OB_I_**: Ordinate Benchmark by fishing fleet segment I

**PCA**: Principal Component Analysis

**PL**: Pelagic Longlines

**PPG**: Passive Polyvalent Gears

**PR**: Percentile linear non linear Regression

**PTP**: Pelagic Trawls + Purse Seines

**QS_IX_**: Qualitative Scores by species X and fishing fleet segment I

**RRB_IX_**=((mean EPC_IX_)/(IVF_X_))*(QS_IX_). Disturbance Resilience Balance by species X and fishing fleet segment I

**SSF**: Small Scale Fisher
