# Supplementary figures and images for: Exploring fishing threat at fleet segment and subregional scale: Least expert knowledge and a resilience versus disturbance‐based approach as conservation's tools for cartilaginous fish
Source: Ecol Evol. 2023 Mar 19;13(3):e9881. doi: 10.1002/ece3.9881 (PMC10025082; doi:10.1002/ece3.9881)

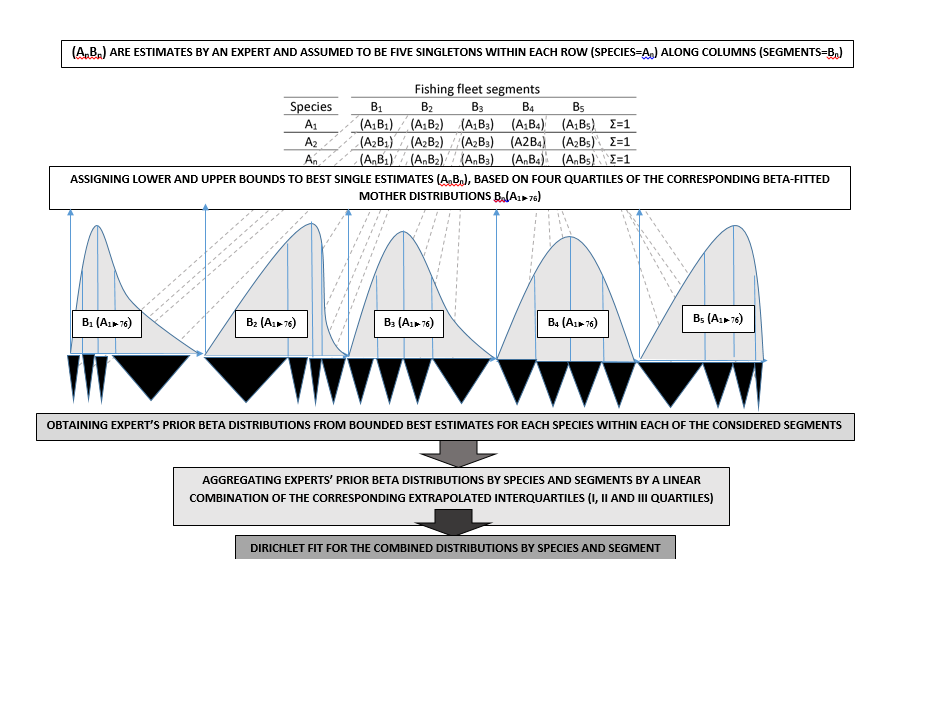

Supplement: Supplementary file 7 — Images S1. [file ECE3-13-e9881-s005.zip › ECE3_9881_Appendi A3.png]

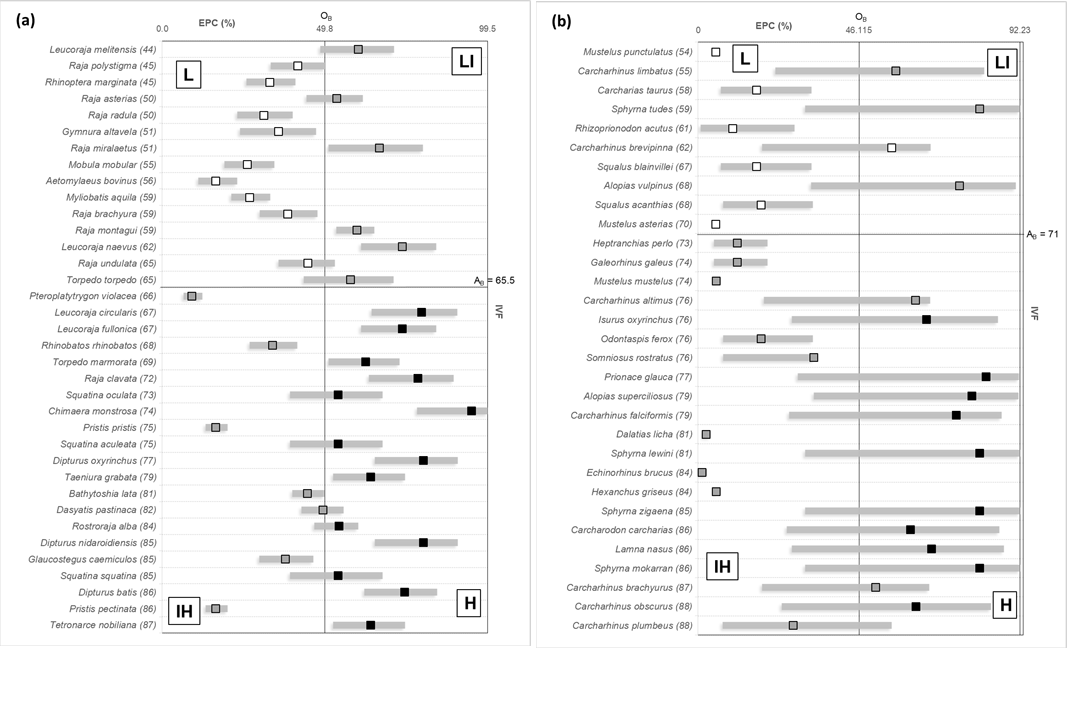

Supplement: Supplementary file 7 — Images S1. [file ECE3-13-e9881-s005.zip › ECE3_9881_Appendix B2 a-b.png]

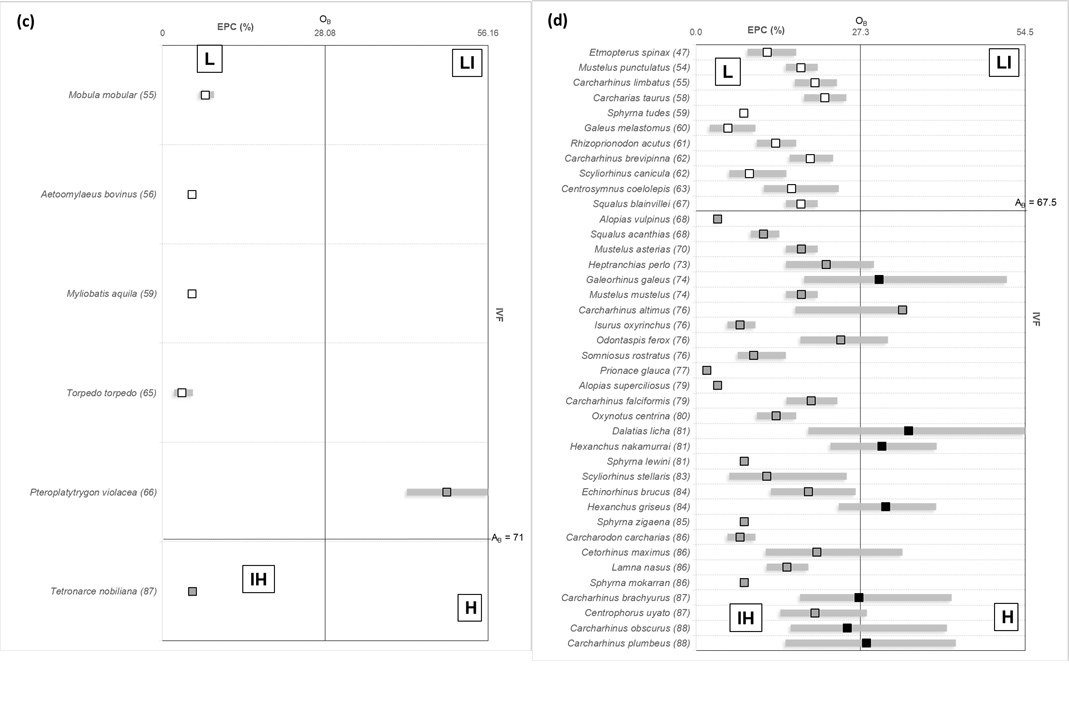

Supplement: Supplementary file 7 — Images S1. [file ECE3-13-e9881-s005.zip › ECE3_9881_Appendix B2 c-d.png]

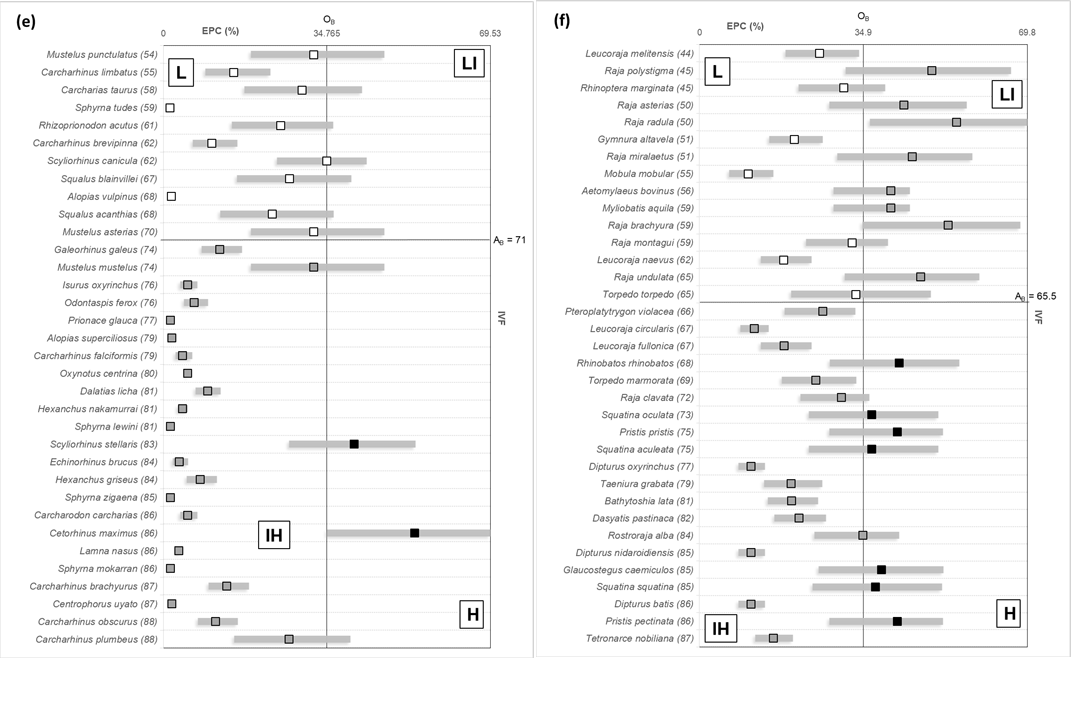

Supplement: Supplementary file 7 — Images S1. [file ECE3-13-e9881-s005.zip › ECE3_9881_Appendix B2 e-f.png]

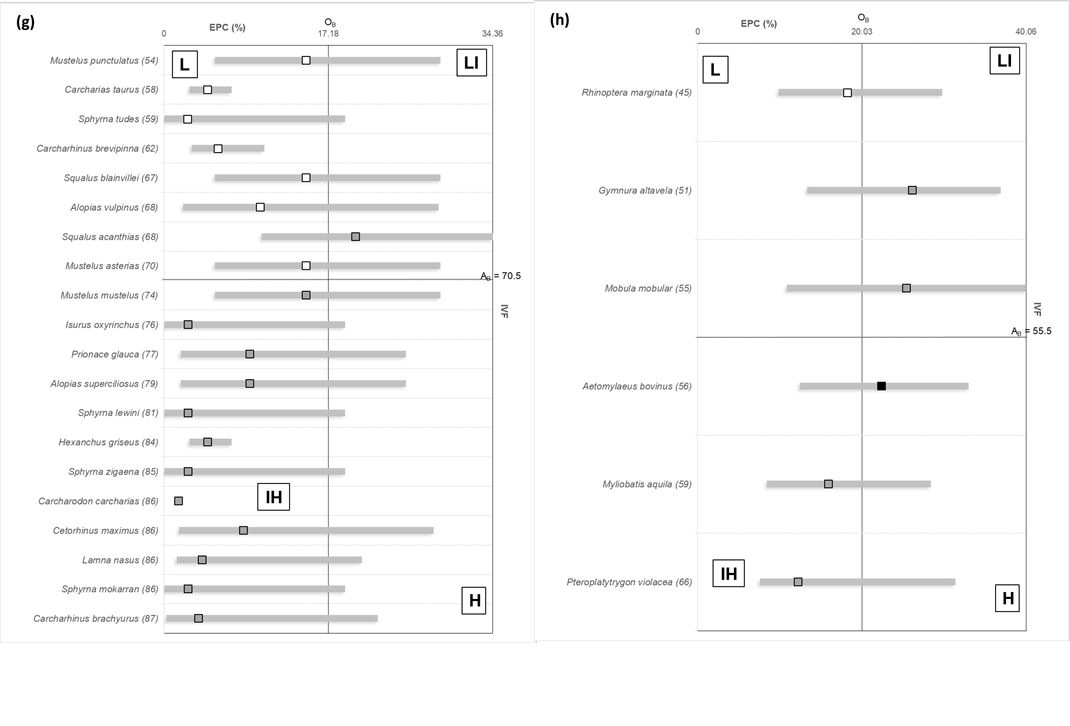

Supplement: Supplementary file 7 — Images S1. [file ECE3-13-e9881-s005.zip › ECE3_9881_Appendix B2 g-h.png]

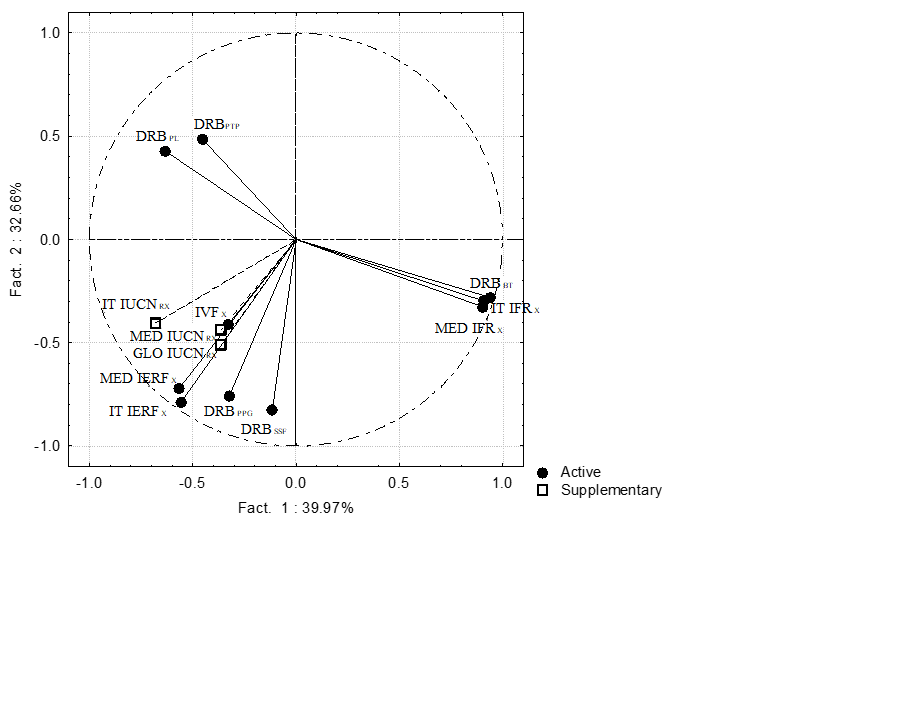

Supplement: Supplementary file 7 — Images S1. [file ECE3-13-e9881-s005.zip › ECE3_9881_Appendix B5 b.png]
